# Deconstruction of Desacetamidocolchicine’s B Ring Reveals a Class 3 Atropisomeric AC Ring with Tubulin Binding Properties

**DOI:** 10.1021/acs.joc.5c00284

**Published:** 2025-05-27

**Authors:** Lauren P. Bejcek, Orugbani S. Eli, Diana M. Kapkayeva, Jordan Nafie, John A. Beutler, Emilio Gallicchio, Dan L. Sackett, Ryan P. Murelli

**Affiliations:** † PhD Program in Chemistry, The Graduate Center of the City University of New York, New York, New York 10016, United States; ‡ Department of Chemistry and Biochemistry, 2037 Brooklyn College, The City University of New York, Brooklyn, New York 11210, United States; § 3147 Biotools, Inc., 17546 Bee Line Highway, Jupiter, Florida 33478, United States; ∥ Molecular Targets Program, National Cancer Institute, National Institutes of Health. 1050 Boyles Street, Frederick, Maryland 21702, United States; ⊥ The Graduate Center of the City University of New York, PhD Program in Biochemistry, New York, New York 10016, United States; # Eunice Kennedy Shriver National Institute of Child Health and Human Development, 2511 National Institutes of Health, 1 Center Dr, Bethesda, Maryland 20892, United States

## Abstract

Colchicine is one of the oldest known microtubule-targeting agents and also represents a classic example of axial chirality and atropisomerism in medicine. This is because colchicine’s axially chiral methoxytropone-trimethoxybenzene (called the AC ring) is directly responsible for tubulin binding and is thermodynamically set into the requisite *aR* form by a point chiral acetamido group on its B ring. Indeed, desacetamidocolchicine (DAAC), a colchicine analogue without the acetamido group, racemizes within minutes. Herein, we describe the synthesis as well as physical and biological characterization of a series of AC ring-containing molecules that represent B-ring further deconstructed variants of DAAC. These studies revealed a novel analogue with an AC ring that is highly stable to epimerization based not on thermodynamic stabilization but rather a high rotational barrier energy. Profiling and characterization of the dihedral angles were carried out computationally and experimentally using vibrational circular dichroism, demonstrating that the ground state dihedral angles of the new molecules differ significantly from those of colchicine. However, despite this difference, the molecule retained antiproliferative, tubulin-binding, and tubulin polymerization inhibitory activity.

## Graphical abstract

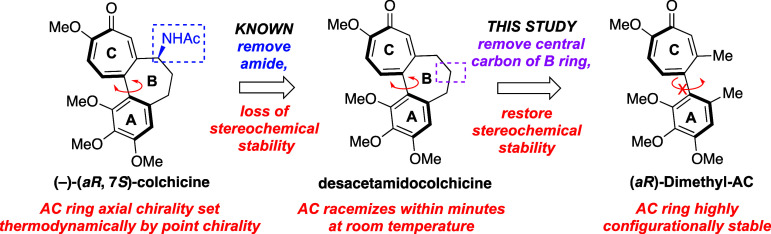

## Introduction

(−)-Colchicine (**1**, Figure 1A) is one of the world’s oldest known medicines, dating back to at least ancient Egypt, when its natural product source, the autumn crocus, was used for treatment of inflammation.^1^ In more recent times, the pure form of colchicine has been used as an FDA-approved drug most closely associated with gout, but also used for prevention of pericarditis and familial Mediterranean fever.^2^ There is also growing interest in other applications.^3^ For example, the FDA very recently approved colchicine for use in the prevention of cardiovascular disease.^4^ Colchicine’s broad value is generally tied to its microtubule destabilizing activity,^5^ which is the mechanism through which many of the most important and widely used chemotherapeutics work. While colchicine is not currently used clinically as a chemotherapeutic due to its high toxicity, there is high active interest in the development of antiproliferative agents that target the colchicine-binding site of tubulin.^6^

**Figure 1:**
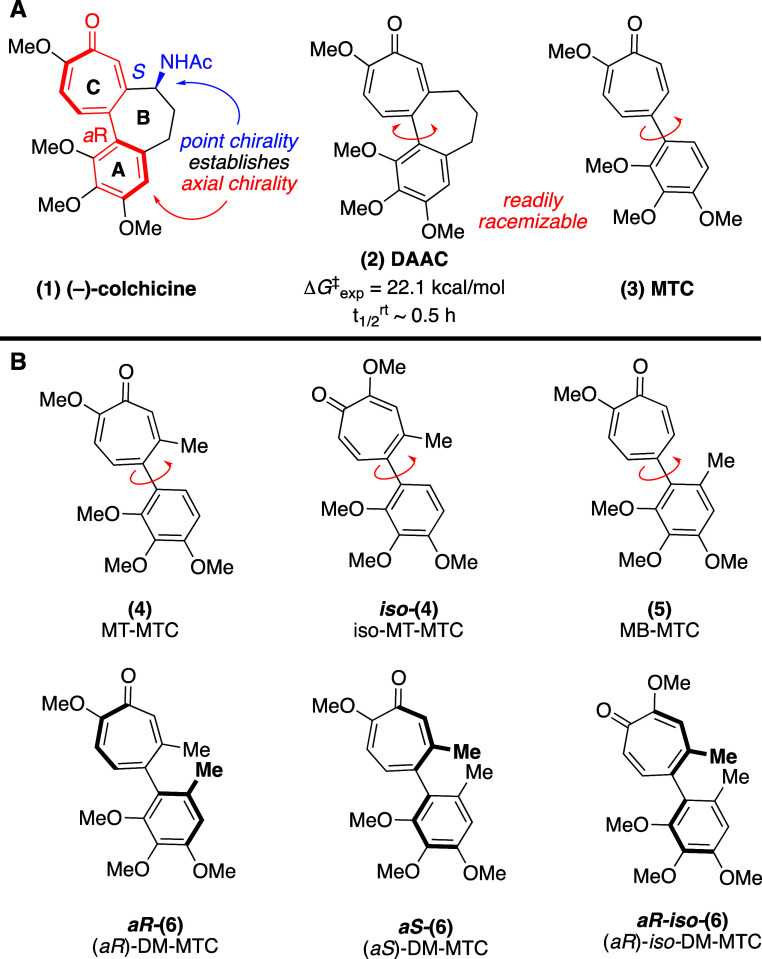
Tubulin-binding AC ring containing molecules. (A) Natural product (−)-colchicine and widely studied, simplified variants. (B) New AC ring analogs studied in present manuscript. *aS*-iso-(6) is also described herein, but omitted for space considerations.

(−)-Colchicine’s tubulin-binding is directly related to its axially chiral AC ring, which is thermodynamically set into the preferred tubulin-binding *aR* (axial *R*) form by nearby *S* point chirality on its 7-membered B ring.^7^ While (+)-colchicine, which favors the *aS* form of the AC ring due to its alternative *R* point chirality, is a substantially less potent tubulin inhibitor,^8^ the readily racemizable AC ring-bearing desacetamidocolchicine (**2**, DAAC, Figure 1A)^9^ and [2-methoxy-5-(2,3,4-trimethoxyphenyl)-2,4,6-cycloheptatrien-1-one] (**3**, MTC, Figure 1A)^10^ each maintain potent tubulin-based antiproliferative activity in line with (−)-colchicine. Given the importance of the AC ring, there has been considerable interest in understanding how its configuration influences both conformation and binding.^11^ However, with few exceptions,^12^ biological studies on AC ring-containing analogs have relied on semisynthetic derivatives of the natural product source.^13^

Herein, we describe the de novo construction of several structural intermediates between DAAC and MTC. These are named Methyl on Tropone of MTC (MT-MTC, **4**, Figure 1B), Methyl on Benzene of MTC (MB-MTC, **5**), and DiMethyl MTC (DM-MTC, **6**). These studies were enabled by a recently described oxidopyrylium cycloaddition/reductive ring-opening approach to methoxytropones.^14^ Through the course of these studies, we identified a highly configurationally stable AC ring analogue, (*a*R)**-6**, that has tubulin-inhibition and antiproliferative activity despite a ground-state dihedral angle and a steep energetic well that varies substantially from AC dihedral angle binding of known colchicine analogs.^15^

## Results and Discussion

### Computational Modeling and Profiling of Torsional Angles

The dynamics of the torsional angles of methylated AC ring analogs **4**–**6** were studied computationally by carrying out a relaxed coordinate scan with Schrödinger Suite’s Jaguar software.^16^ In these studies, an optimized geometry was generated, and the dihedral angle χ was varied in an iterative nature by 10° (Scheme 1A). A full 360° beyond the first coplanar orientation was scanned in order to establish a full and iterative 0–360° window (Scheme 1B). Of note, the three methoxy groups on the A ring did not reorganize during the simulations due to their proximity, and this caused enantiomers with different energies near the ground-states. Thus, for ground-state profiling, the lowest energy of each enantiomer was chosen as the most accurate ground-state for each pose. These values are plotted from 0–180° in Scheme 1C.^17^

**Scheme 1:**
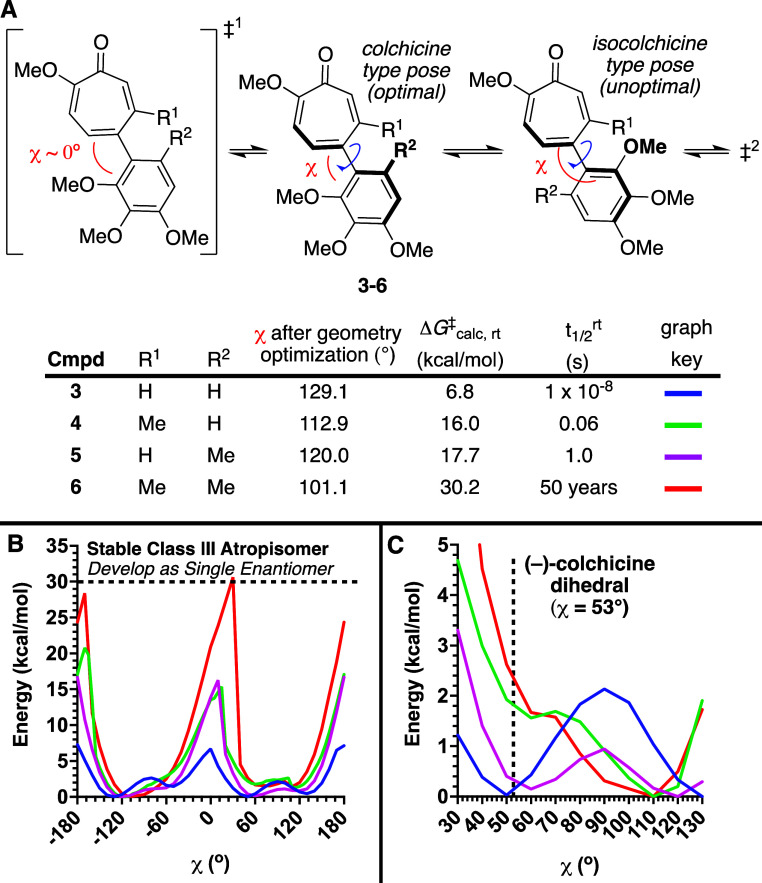
Dihedral Angle Modeling of **3–6**, (A) Illustration of Torsional Angle of Interest, along with Table Describing Key Data Points from Computational Optimization and Torsional Scan Studies (M06-2X/6-311G**), (B) 360° Torsional Profile of **3**–**6** for Approximating Chiral Axis Stability 30 kcal/mol is Viewed as a Threshold for the Rotational Barrier About a Chiral Axis above which Molecules can be Developed as Single Enantiomers, (C) Ground-State Dihedral Profiling of **3**–**6**, along with Known Tubulin-Bound (−)-Colchicine Dihedral Angle (pdb 1SAO)^15^

The dihedral angle of the minimized structures for MTC (**3**) was 129.1°, although profiling of its dihedral angle revealed an energetically comparable pose at ∼50° (blue, Scheme 1C). This observation, as well as local energetic maxima at its planar and perpendicular orientations, have been observed previously.^18^ A similar set of two near energetically equivalent local minima were also observed for MB-MTC (**5**) at ∼120° and 60°, shifted toward the perpendicular orientation (magenta, Scheme 1C). We consider these two energetic minima to represent a “colchicine-type” pose (∼60°) and an “isocolchicine-type” pose (∼120°), as they closely match the dihedral angles of the AC ring in colchicine and isocolchicine, respectively (see Scheme 1A for reference and Scheme 3E for structure of isocolchicine). MT-MTC (**4**), meanwhile, has a more defined energetic minimum at 112.9°, and its colchicine-type pose (∼60°) is roughly 2 kcal/mol uphill in energy (green, Scheme 1C). While **4** and **5** differ significantly near their ground state, they share a similar barrier to enantiomerization (∼17 kcal/mol), which is significantly higher than that of MTC (**3**, Δ*G* ^‡^ = 6.8 kcal/mol), but below that of DAAC (**2**, Δ*G* ^‡^ = 22.1 kcal/mol).^19^ DM-MTC (**6**) has a ground-state profile similar to MT-MTC (**4**) (red vs green, Scheme 1C), but it possesses a sharper energetic well and a ground-state closer to a coplanar orientation (χ = 101.1°). It also possesses a very high rotational barrier (Δ*G* ^‡^_calc_ = 30.2 kcal/mol), which would place it at the frontier of class 3 atropisomer designation. The term “class 3 atropisomer” describes molecules with rotational barriers of chiral axes above 30 kcal/mol, and medicinal chemists generally view such molecules as candidates for single atropisomer drug development.^20^ This distinguishes them from compounds with moderately stable chiral axes that are not good candidates for single atropisomer drug development (Δ*G* ^‡^ = 22–30 kcal/mol, “class 2”), and unstable chiral axes (Δ*G* ^‡^ < 22 kcal/mol, “class 1’). The implications to this are that if (*aR*)**-6** is active, it would have a narrow range of accessible dihedral angles that are mostly distinct from those accessible for (−)-colchicine^18^ and could thereby alter its selectivity and physiochemical properties. Given that the broad medicinal value of (−)-colchicine is directly tied to this AC ring, the synthesis, physical, and biochemical evaluation of **6** was warranted.

### Synthesis of AC Ring Analogs

Established methods for synthesizing the AC ring of colchicine are almost exclusively related to the natural product total synthesis^21^ and are not easily adaptable to analogs without a B ring. The only established AC ring strategy that seemed viable for the synthesis of **6** was an 11-step synthesis of a chloro-AC analog reported over 30 years ago by Banwell.^12a^ We recently described a convenient oxidopyrylium (5 + 2) cycloaddition/reductive ring-opening approach to access methoxytropones that could be used to generate the AC ring system.^14,22^ Using this strategy, cycloadduct **18** was generated from alkyne **14** and oxidopyrylium ylide dimer **12** and treated with samarium iodide with an acidic aqueous workup (pH 3 phosphate buffer).^23^ Excitingly, the only product observed was the AC ring analogue *iso* **-6** (Scheme 2). *iso* **-6** was viewed as a valuable control molecule, as it has accessible torsional angles of the AC ring more consistent with isocolchicine.^24^ It could also be converted to **6** through demethylation followed by a nonselective methylation,^25^ providing a separable mixture of *iso* **-6** and **6**. By substituting alternative oxidopyrylium dimer **11** and/or alkyne (**13**), this route also provided additional analogs **3**, *iso* **-4**, **4** and **5**. Of note, cycloaddition reactions with dimer **11** was sluggish and low yielding, and required higher temperatures that appears to produce a reaction-compromising dimer rearrangement.^26^ Fortunately for the present studies, cycloadducts derived from **14** were only a single step from the target molecules. It is worth noting that this lower reactivity presently prevents a more direct route to **6** from oxidopyrylium dimer **11** and an even less-reactive alkyne-methylated variant of **14**, but efforts are underway to address this limitation.

**Scheme 2:**
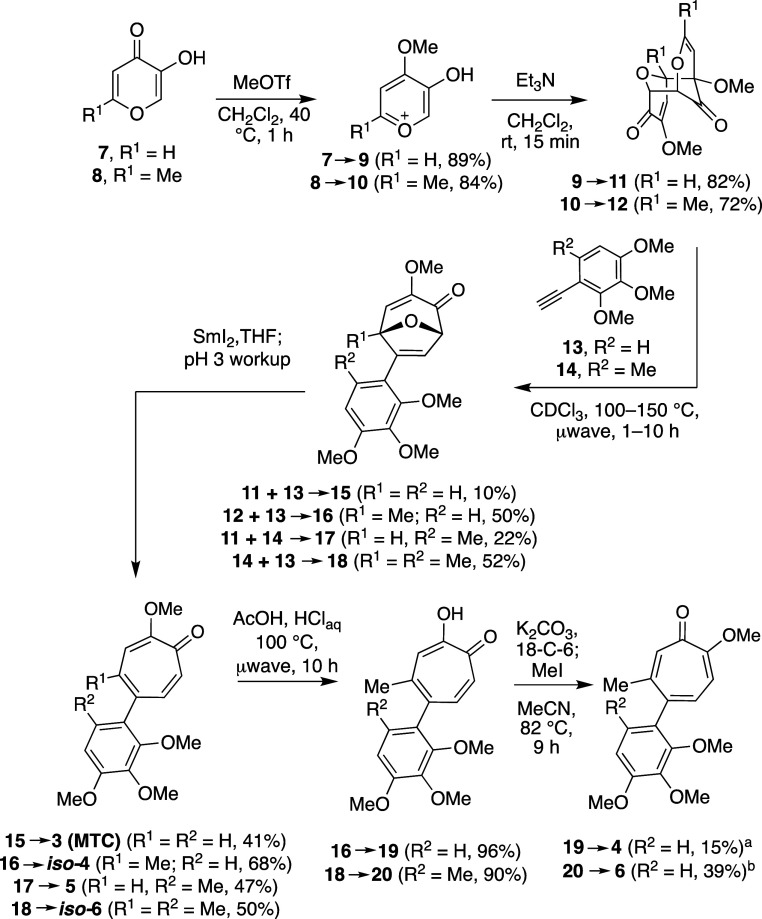
Oxidopyrylium Cycloaddition/Reductive Ring-Opening Approach to AC Ring Analogs

a iso**-4** was also isolated in 19% yield.
b Represents combined yield of (*aR*)**-6** (20%) and (*aS*)**-6** (19%), following preparatory chiral resolution via Diacel AI polysaccharide-based column. **(±)-iso-6** was also isolated in 25% yield.

### Chiral Resolution, Characterization, and Configurational Stability Studies

(*aR*)**-6** and (*aS*)**-6** were readily separable from one another and racemic *iso* **-6** through the use of a polysaccharide-based chiral column (Diacel IA column)^27^ fitted to an automated medium-pressure liquid chromatography unit (Biotage Isolera Prime). Racemization was observable after heating a solution of either enantiopure (*aR*)- or (*aS*)**-6** at 135 °C in xylenes over a few hours,^16^ and the rate of enantiomerization was extrapolated using the Eyring equation (Δ*G* ^‡^ = 31.2 ± 0.3 kcal/mol).^28^ (*aR*)- and (*aS* **)-iso-6** were separable in an analogous fashion using a chiral IC polysaccharide column. However, no racemization was observed, even after heating to 145 °C for 10 h, at which point decomposition was observed. Thus, we can project that the rotational energy barrier is only 37 kcal/mol. However, a computationally determined rotational barrier for *iso* **-6** was 33.5 kcal/mol, which is roughly 3 kcal/mol more than that calculated for **6**. One potential explanation for this notable difference in configurational stability is the greater double bond character of the methyl-bearing C^4^–C^5^ carbon of *iso* **-6** in comparison to the C^5^–C^6^ in **6**, as this would both shorten and rigidify this steric-bearing bond (see Scheme 3A/B for details). Evaluation of the lower-in-energy local maxima from the torsional scan (χ = 170°) reveals that the smaller bond distance of *iso-* **6** (1.39 Å vs 1.48 Å) leads to a larger torsional angle between the methyl and methoxy-bearing oxygens (blue, χ^2^ = 9.9° vs 6.9°) and smaller distance between the appendages (2.60 vs 2.68 Å) (Scheme 3C). Studies have previously described differences in rotational barriers of DAAC (**2**) and its analogous isomer that, while less dramatic, are consistent with this trend (Δ*G* ^‡^ = 22.1 vs 23.4 kcal/mol).^18^

**Scheme 3:**
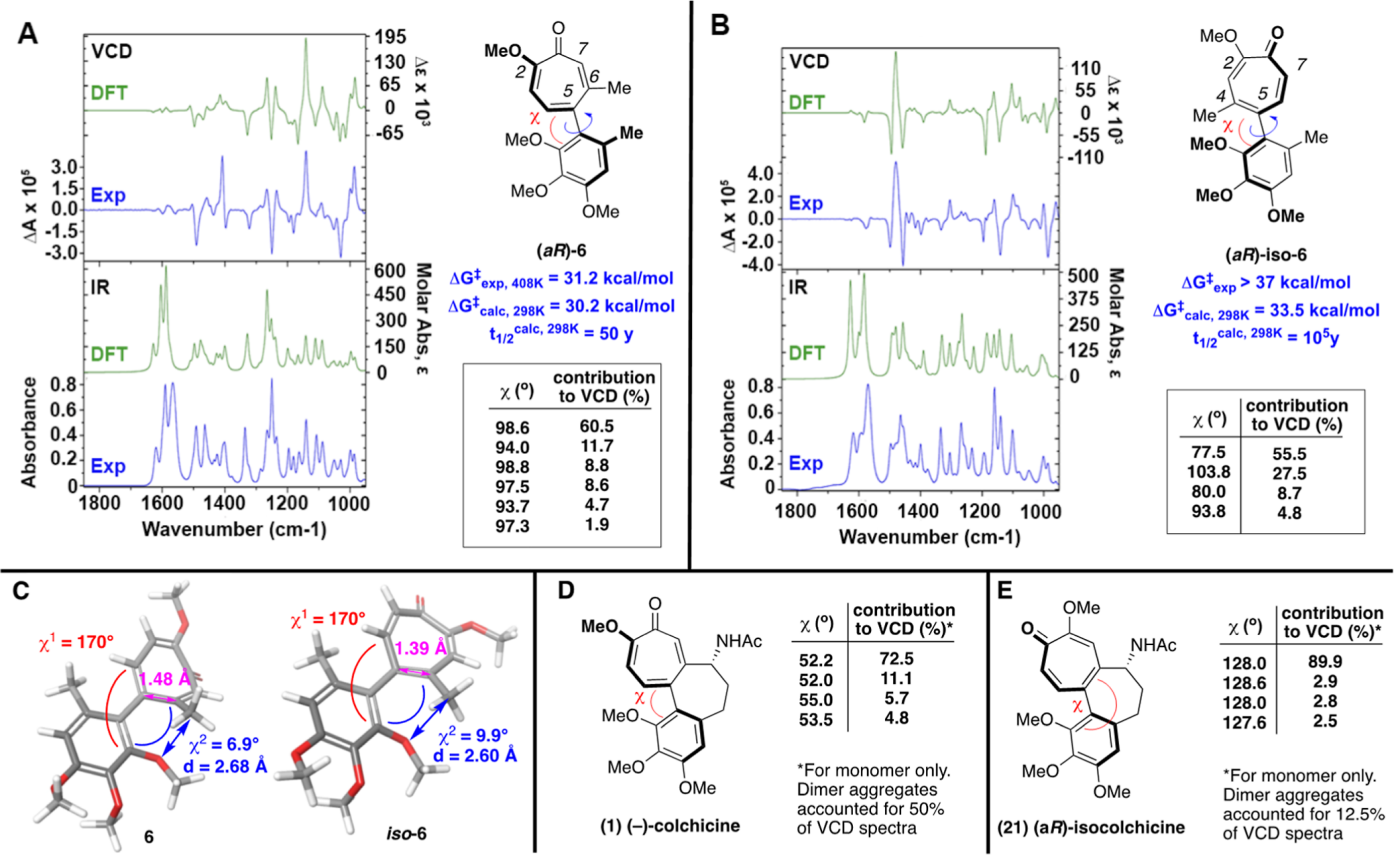
Characterization of AC Ring Chirality and Stability, (A/B) Experimental (EXP) and Calculated (DFT) Vibrational Circular Dichroism (VCD) Spectra of (*aR*)**-6** and (*aR*)-*iso* **-6**, along with Contributions to VCD. Also Shown are Free Energy Values Determined Experimentally and Computationally, (C) (*aR*)**-6** and (*aR* **)-iso-6** at Local Maxima (χ = 170°), Representing Lowest Path to Enantiomerization, Illustrating Impact of Double Versus Single-Bond Character on Torsion and Methyl-Methoxy Bond Distance. (D/E) Torsional Angle Contributions to VCD for (−)-Colchicine and (aR)-Isocolchicine, Excluding Contributions of Aggregates

Absolute stereochemistry of (*aR*)**-6**, (*aS*)**-6**, (*aR*)-*iso* **-6**, and (*aS*)-*iso* **-6** were determined using VCD (Scheme 3).^29^ VCD is a technique which evaluates CD spectra in the IR range and can be used in combination with density functional theory-based modeling to determine absolute stereochemistry and to provide insight into conformations in solution. Molecular modeling of both (*aR*) and (*aS*)**-6** yielded 20 unique conformations within 7 kcal/mol of one-another, each of which underwent further DFT optimization at several levels of theory to calculate VCD.^30^ Although many of the methods gave computed VCD spectra that closely matched the experimental spectra, optimization at B3LYP/cc-pVTZ provided the best match, and in these studies, 6 conformers were identified that contributed 1% or greater to the Boltzmann weighted average. While most of the conformational flexibility was coming from the methoxy groups, there was some small variation of the dihedral angle defining the chiral axis (93–100°), with 98.6° contributing to 60.5% of the spectra (Scheme 3A). A similar treatment was given to *iso* **-6**, with the B3PW91/cc-pVTZ combination, this time yielding slightly better results than the other methods. Unexpectedly, the VCD spectra were considerably different from that of **6** on account of the greater variation of dihedral angles of contributing molecules (77–104°, Scheme 3B). Most significantly, the major contributor has a dihedral angle of 77.5° (55.5% contribution share), whereas the second greatest contributor has a vastly different dihedral angle of 103.8° (27.5% contribution share). In DFT-based dihedral profiling (M06-2X/6-311G**), *iso* **-6** had two ground state minima—one at 70° and one at 100°—consistent with these findings.^17^ These two conformations are roughly equidistant from the perpendicular conformation, and consequently, many of their VCD peaks are equal to and opposite in signal strength, weakening the observed signal.

Given this unexpected difference, we wanted to gauge whether the VCD spectra of colchicine and isocolchicine may also provide evidence of larger differences in their conformers. While a few VCD spectra of colchicine have been reported,^31^ we are unaware of any published VCD studies of isocolchicine, and thus carried out studies on each (Scheme 3D/E), as well as their respective enantiomers.^17^ In these studies, both colchicine and isocolchicine appeared to have very similar ranges of dihedral angles in their VCD-contributing conformers. The only notable, major difference seemed to be in the molecules’ ability to aggregate into dimers. The presence of these aggregates made it necessary to model them and combine these with the spectra from monomeric forms to get consistent data.^17^ This aggregation is well-known with colchicine,^32^ and in our studies the dimer contributed to roughly 50% of the VCD spectra. On the other hand, isocolchicine dimer aggregate only accounted for roughly 12.5% of the VCD spectra, which appears to be due to differences in ability to generate appropriate hydrogen bond network.^17^ Regardless, the differences that were observed for **6** and *iso* **-6** did not translate in any notable manner to the B-ring containing AC rings.

### Biological Testing

The four atropisomeric compounds—(*aR*)**-6**, (*aS*)**-6**, (*aR* **)-iso-6**, and (*aS*)-iso-6)—were each evaluated in the NCI 60 5-point screen.^33^ (*aR*)**-6** showed substantial growth suppression across various cell-lines at 10 μM (Figure 2A), with a mean GI_50_ value of 2.4 μM, a selectivity range of 2.39 log units, and total growth inhibition (TGI) value of 63 μM. A distinct plateau at the TGI level of analysis was also observed for (*aR*)**-6**, which is characteristic of tubulin interactive agents.^34^ Meanwhile, (*aS*)**-6** and (*aR*)-*iso* **-6** were not cytotoxic at the highest concentration tested (100 μM), and (a*S*)-*iso* **-6** was approximately 10-fold less potent in the NCI-60 screen than (*aR*)**-6** (GI_50_, _mean_ = 27 μM), with a smaller selectivity range (1.46 log units) and TGI mean value (91 μM). Growth inhibition of (*aR*)**-6** was 10 to 50-fold less potent than colchicine.

**Figure 2:**
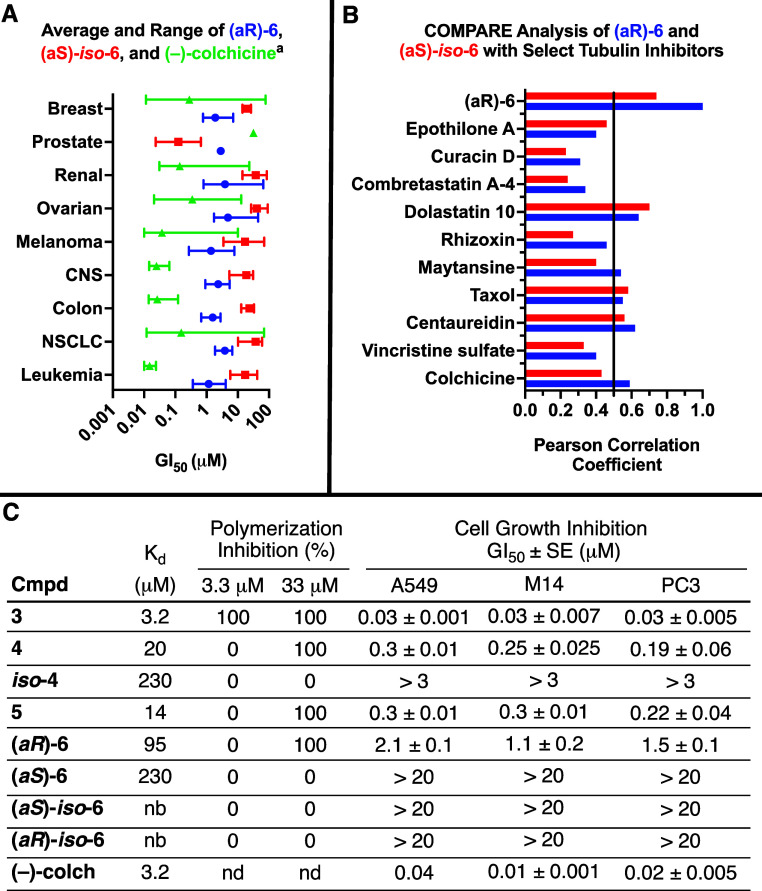
Representative Biological Results. (A) Summary of growth inhibition data in NCI-60 cell screen data of (*aR*)**-6** and (*aS*)-*iso* **-6**, along with prior data on colchicine obtained from the NCI-60 database of screening results, averaged by cell type. ^a^For a complete list of results, see the Supporting Information. (B) Pearson correlation coefficient between (*aR*)**-6**, (*aS*)-*iso* **-6**, and known tubulin interactive compounds in NCI-60 assay. A coefficient of >0.5 is considered significant. (C) Tubulin binding constants (*K*_d_) and polymerization inhibition at select concentrations at 25 °C, as well as 50% growth inhibition concentration (GI_50_) of select cell lines, reported as average of 3 trials ±standard error for all experiments except colchicine with A549, which is based on 2 experiments. “nd” denotes that an experiment was not performed, and nb denotes that the no binding was observed within the detection limit of the assay.

The TGI level of responses for (*aR*)**-6** and (*aS*)-*iso* **-6** were subsequently compared to known tubulin interactors using a pattern recognition algorithm (COMPARE analysis, Figure 2B).^35^ Many of these known compounds had been tested at multiple different high concentrations, and a single set of high concentration tests was chosen for each compound, where it displayed the most robust selectivity pattern. Notably, (*aR*)**-6** showed a meaningful correlation (Pearson correlation >0.5) to (−)-colchicine, while (*aS*)-*iso* **-6** fell short. However, a few tubulin-targeting molecules had a significant correlation with both molecules: the colchicine site binder centaureidin, the vinca site binder dolastatin 10, and taxol. However, the closest correlation was in fact between (*aR*)**-6** and (a*S*)-*iso* **-6**, supporting a shared mechanism of action. While several well-established tubulin inhibitors did not have meaningful correlations with either molecule, enough positive correlations supported the testing of the compounds in biochemical assays.

All of the AC ring-containing molecules synthesized were subsequently tested for their ability to bind to tubulin (*K*_d_) and inhibit microtubule formation (Figure 2C). MTC (**3**) had the lowest binding constant of all compounds tested, and was the only compound to inhibit microtubule polymerization at 3 μM. Compounds **4**, **5**, and (*aR*)**-6** had binding constants 5–30 fold less potent than **3**, but were each capable of inhibiting microtubule polymerization at 33 μM. While *iso* **-4** and (*aS*)**-6** each appeared to bind to tubulin near the limits of the assays, neither compound nor either enantiomer of *iso* **-6** inhibited microtubule polymerization at the concentrations tested.

Growth inhibition studies were carried out with the library against select cancer cell-lines, and this activity was tracked closely with biochemical assays. Specifically, molecules that did not inhibit microtubule polymerization did not have any significant growth inhibition in cells, and the differences in binding constants of active molecules tracked closely with differences in growth inhibition (Figure 2C). (−)-colchicine was also tested as a control in cell-based assays, and showed comparable activity to MTC (**3**), consistent with literature precedence.^10a^ This data support that the cytotoxicity of (*aR*)**-6** is directly related to microtubule disruption, and the lower cytotoxicity is because of its lower affinity for tubulin. Furthermore, the activity differences between active compounds tracks with prior energetic well profiling of **3**, **4**, and (*aR*)**-6**, which suggested that **4** and **5** prefer an isocolchicine-type pose when unbound, and require uphill energetic climbs to achieve a dihedral angle near that of colchicine (see Scheme 1C).^36^ The lower activity of **5** is not as clear based on these data, but one possibility is that its broad energy well could lead to entropic penalties when bound.

### Computational Studies on AC Analog–Tubulin Interaction

To glean additional insight into these differences in activity, we turned to computational modeling of the library in tubulin. Binding constants were calculated using the alchemical Relative Binding Free Energy method (RBFE) with the AToM-OpenMM software version v3.5.0 ^37^ (https://github.com/Gallicchio-Lab/AToM-OpenMM), and MTC (**3**) was used as a reference. MTC and the other compounds were aligned to the crystal structure of colchicine bound to tubulin (PDB 5ITZ).^38^ Hamiltonian replica exchange alchemical molecular dynamics calculations were performed with 22 replicas for 20 ns per replica, and 100 ns molecular dynamics simulations for the dihedral angle analysis were conducted for each compound bound to tubulin and free in solution. From this data, we were able to extract a series of poses, through which we could profile AC ring dihedral angles bound and unbound (ie, Figure 3A), and obtain computational binding constants (Figure 3B).

**Figure 3:**
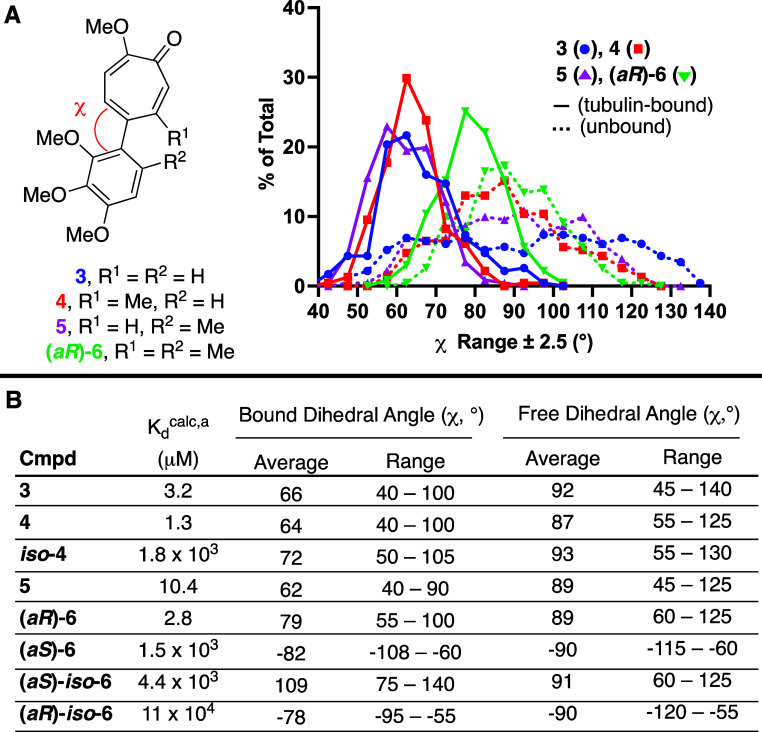
Representative Results from Modeling of AC Ring Analog Interactions with Tubulin in silico. (A) Distribution of dihedral angles for tubulin active compounds **3**, **4**, **5**, and (*aR*)**-6** when bound (solid line) and unbound (dotted line). Data points represent the total number of poses within 5° windows, shown at the midpoint. (B) Summary computational data in tabular form, including average and range of dihedral angles. ^a^Binding constants were calculated using relative binding free energies and the experimentally determined binding constant of **3** in Figure 2C.

What was immediately apparent from these studies was that for tubulin-active molecules, modeling gave binding constants within 5-fold of **3** (**4**, **5**, and (*aR*)**-6**), while those that were mostly inactive differed by at least 100-fold (Figure 3B). Compounds **4** and **5** both bound to tubulin in silico with dihedral angle profiles that were comparable to those of **3**, whereas (*aR*)**-6** bound with much larger dihedral angles. When unbound, each of the compounds had comparable average dihedral angles that was near coplanar, but differed noticeably in their distribution. Specifically, compound **3** had dihedral angle distributions nearly uniformly from 50° to 130°, while (*aR*)**-6** was mostly in the 80° to 100° range (Figure 3A). **4** profiled more similarly to (*aR*)**-6**, and **5** more similar to **3**, consistent with the energetic profiling results (see Scheme 1C). Thus, (a*R*)**-6** has the smallest change in structure to binding, illustrative of its high conformational rigidity.

Looking more closely at the structures generated in silico, two main hydrogen bond contacts are observed (Figure 4). The most stable of these contacts is that between the oxygen of the tropone carbonyl and the hydrogen of the amide backbone of valine 181 (not shown). A second hydrogen bond is observed between the thiol hydrogen of a highly flexible cysteine 241 and the trimethoxybenzene-associated oxygen atoms, and more specifically those of the methoxy groups at the meta and/or para positions (illustrated in Figure 4). MTC (**3**) shows consistent hydrogen bonding between this thiol and at least one of these two trimethoxybenzene-associated methoxy groups, whereas it is less frequent in the case of (*aR*)**-6**. Given that the para methoxy groups are in line with the AC chiral axes, the methoxy at the para position is not influenced as much by the differences in dihedral angles as the meta methoxy groups would be. This could help explain the diminished potency of (*aR*)**-6** as compared to **3** observed experimentally. Regardless, the drastically different binding pose of (*aR*)**-6** to tubulin as compared to colchicine could provide intriguing opportunities for studying dihedral influence on tubulin inhibition, potentially even isotype selectivity, as well as off-target effects.

**Figure 4:**
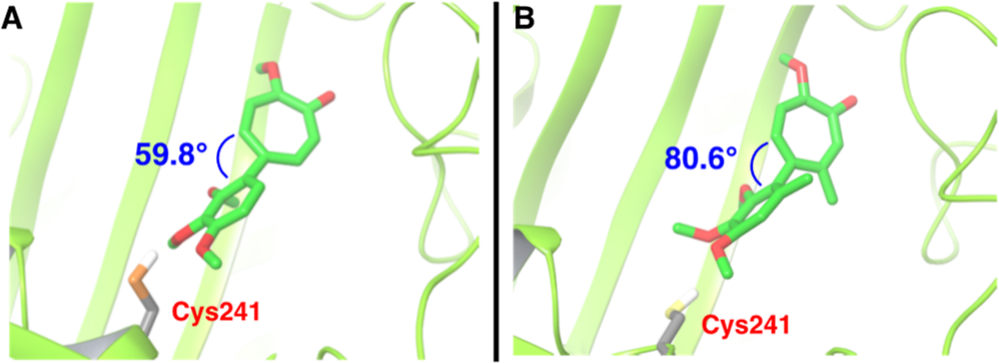
Representative Images of AC ring Analogues from Simulations. (A) is **3** (MTC) and (B) is (*aR*)**-6**, chosen from molecular dynamics structures with dihedral angles that are representative of major poses.

## Conclusions

A simple, deconstructed variant of DAAC (**2**), DM-MTC ((*aR*)**-6**), is highly stable to epimerization with an experimental energy barrier to inversion of 31.2 ± 0.3 kcal/mol, nearly 10 kcal/mol higher than that of the B-ring containing desacetomidocolchicine (**2**). Experimental and computational studies demonstrate that (*aR*)**-6** exists with a torsional angle of its pharmacoactive AC ring of approximately 100°, which is nearly 50° from how it exists in colchicine both in the solution phase and when bound to tubulin. Despite this difference, (*aR*)**-6** still possesses cytotoxicity against many cell-lines at low micromolar concentrations and is capable of binding to tubulin and inhibiting microtubule polymerization. Molecular dynamics studies suggest that (*aR*)**-6** may prefer to bind to tubulin with a dihedral angle at or around 80°, which differentiates it from the 60° dihedral angle preferred **3**, **4**, and **5** in similar modeling experiments. Thus, (*aR*)**-6** represents a new and novel tubulin binding analogue that could complement existing microtubule destabilizing agents that bind to the colchicine-binding site.

## Experimental Section

### General Information

All starting materials and reagents were purchased from commercially available sources and used without further purification except for CH_2_Cl_2_ and benzene, which were purified on a solvent purification system prior to the reaction. ^1^H NMR shifts are measured using the solvent residual peak as the internal standard (CHCl_3_ δ 7.26, D_2_O δ 4.79) and reported as follows: chemical shift, multiplicity (s = singlet, bs = broad singlet, d = doublet, t = triplet, dd = doublet of doublet, q = quartet, m = multiplet), coupling constant (Hz), and integration. ^13^C NMR shifts are measured by using the solvent residual peak as the internal standard (CDCl_3_ δ 77.20) and reported as chemical shifts. Infrared (IR) spectral bands are characterized as broad (br), strong (s), medium (m), and weak (w). Microwave reactions were performed via the Biotage Initiator 2.5 in a sealed vessel. Purification via reverse phase column chromatography was performed on the Biotage Isolera Prime, with Biotage SNAP 12 g cartridges, in a solvent system of acetonitrile in water, each solvent containing 0.05% trifluoroacetic acid (TFA). Oxidopyrilium dimers **(11**–**12)** ^39^ alkyne **13**, cycloadducts **15** and 16, and methoxytropones **3, 4** and *iso* **-4** were synthesized as previously reported.^14^

### DFT Calculations

#### General Information for DFT Calculations

Computational modeling was carried out using Schrödinger Suite’s Jaguar software. Unless otherwise noted, all calculations were performed using the M06-2X functional and the 6–311G** basis set in the gas phase at room temperature (298.15 K). This functional and basis set was chosen for its cited accuracy for both conformational and energetic accuracy.^40^

#### Torsional Angle Profiling

AC ring structures (**3**–**6**) were submitted to a geometry optimization to provide a preliminary ground-state conformation. These structures were used to determine ground state dihedral angles provided in Scheme 1A. These were then submitted to a relaxed coordination scan of dihedral χ in increments of 10°, scanned in an iterative rather than simultaneous fashion by selecting “previously optimized geometry”, to a final dihedral angle of ˈ540°′. This was done in order to obtain a full and iterative 360° window for each compound (180–540°), and this full window is provided in Scheme 1B, adjusted to −180° to 180°, and plotted against change in energy (kcal/mol). Additional details can be found in the Supporting Information file.

#### Free Energy Barrier Determination

Free energy barriers were determined as follows. Single point energy, including vibrational frequency measurements, were obtained for each molecules’ global minima and 2 local maxima. In each case, none of the minima (ie, GS) had a vibrational frequency, while all of the local maxima (ie, TS) had a single negative frequency, and these values are included in the Supporting Information. A folder of compiled text files of Cartesian coordinates (*xyz*) from these computations is provided in a separate zip file as a unique Supporting Information file. Expected “observed” free energies were extrapolated from these values, and additional details on these computations can be found in the main Supporting Information document.

### Synthesis and Characterization

#### 2-Ethynyl-2,3,4-trimethoxy-1-methylbenzene (**14**)

To a flame-dried round-bottom flask equipped with a stir bar was added 2-iodo-3,4,5-trimethoxy-1-methylbenzene (5 g, 16 mmol) followed by bis­(triphenylphosphine)palladium chloride (570 mg, 0.81 mmol) and copper iodide (155 mg, 0.81 mmol). The reaction vessel was sealed and purged with argon. Toluene (15 mL) and triethylamine (20 mL) were then added to the reaction, and argon bubbled through the resulting solution for approximately 10 min. Trimethylsilyl (TMS) acetylene (3.47 mL, 24.3 mmol) was added via syringe, and then the reaction was heated at 90 °C for 16 h. The solution was then cooled to room temperature, filtered through Celite, and concentrated under reduced pressure. The resulting oil was then dissolved in methanol (20 mL), and potassium carbonate (15 g) was added. The mixture was stirred at room temp for approximately 24 h, diluted with CH_2_Cl_2_ (20 mL), and washed with water (2 × 20 mL). The organic layer was dried over Na_2_SO_4_, filtered and concentrated under reduced pressure. The resulting oil was purified by chromatography (Biotage Isolera Prime, SiliCycle Silia*Sep* 40 g silica gel, 40–63 μm 60 Å, solvent gradient: 0–100% EtOAc in hexanes (500 mL) and concentrated to reveal **14** as a pale yellow solid (3.2 g, 96% yield). m.p. = 52–53 °C. *R*_f_ = 0.94, 60% EtOAc/pentane. IR (thin film, KBr): 3273 (br), 2937 (m), 1596 (m), 1493 (m), 1463 (w), 1400 (w), 1335 (s), 1246 (m), 1124 (s), 1077 (w), 1034 (w) cm^–1^. ^1^H NMR (400 MHz, CDCl_3_): δ 6.50 (s, 1H), 3.94 (s, 3H), 3.83 (s, 3H), 3.82 (s, 3H), 3.40 (s, 1H), 2.37 (s, 3H). ^13^C­{^1^H} NMR (101 MHz, CDCl_3_): δ 155.3 (s), 153.8 (s), 139.8 (s), 137.5 (s), 109.1 (s), 108.6 (s), 83.7 (s), 78.6 (s), 61.1 (s), 61.0 (s), 55.9 (s), 20.7 (s). HRMS (ESI + TOF)*m*/*z*: (M + H)^+^ calcd for C_12_H_15_O_3_^+^: 207.1017; found, 207.1016.

#### General Procedure for Synthesis of 8-oxabicyclo[3.2.1]­octa-3,6-dienones (**17, 18**)

A solution of oxidopyrylium dimer (1 equiv) in CDCl_3_ (0.2 M) (10–17 equiv) was added in a microwave vial. The reaction was subjected to microwave irradiation at 120 °C–150 °C for 1 to 10 h. The resulting solution was immediately subjected to purification via column chromatography (Biotage Isolera Prime, SiliCycle SiliaSep 10 g silica gel, 40–63 μm 60 Å, solvent gradient: 0–100% EtOAc in hexanes [500 mL]).

#### (±)-(1*S*,5*S*)-3-methoxy-6-(2,3,4-trimethoxy-6-methylphenyl)-8-oxabicyclo­[3.2.1]­octa-3,6-dien-2-one (**17**)

Yellow oil **17** (73 mg, 42% yield) obtained from oxidopyrylium dimer **11** (132 mg, 0.52 mmol, 1 equiv) alkyne **14** (2 g, 9.70 mmol, 17 equiv) after 150 °C for 40 min. *R*_f_ = 0.83 in 60% ethyl acetate/pentane. IR (thin film, KBr): 2937 (w), 2838 (w), 1712 (s), 1610 (m), 1494 (m), 1463 (m), 1397 (m), 1334 (w), 1195 (s), 1136 (s), 1097 (s), 1043 (w), 992 (m), 918 (w), 896 (w), 839 (w) cm^–1^. ^1^H NMR (400 MHz, CD_3_CN): δ 6.68 (s, 1H), 6.29–6.20 (m, 2H), 5.36 (d, *J* = 4.9 Hz, 1H), 4.98 (d, *J* = 2.5 Hz, 1H), 3.82 (s, 3H), 3.78 (s, 3H), 3.76 (s, 3H), 3.50 (s, 3H), 2.16 (s, 3H). ^13^C­{^1^H} NMR (101 MHz, CD_3_CN): δ 191.0 (s), 154.4 (s), 153.6 (s), 152.7 (s), 147.3 (s), 141.0 (s), 133.2 (s), 126.0 (s), 120.1 (s), 117.9 (s), 110.8 (s), 88.6 (s), 82.36 (s), 61.7 (s), 61.2 (s), 56.6 (s), 55.2 (s), 20.8 (s). HRMS (ESI + TOF)*m*/*z*: (M + H)^+^ calcd for C_18_H_20_O_6_^+^: 333.1338; found, 333.1341.

#### (±)-(1*S*,5*S*)-3-methoxy-5-methyl-6-(2,3,4-trimethoxy-6-methylphenyl)-8-oxabicyclo­[3.2.1]­octa-3,6-dien-2-one (**18**)

Yellow oil **18** (456 mg, 52% yield) obtained from oxidopyrylium dimer **12** (362 mg, 1.28 mmol, 1 equiv) and alkyne **14** (3.2 g, 15.4 mmol, 12 equiv) afer 120 °C for 2 h. *R*_f_ = 0.87 in 60% ethyl acetate/pentane. IR (thin film, KBr): 2937 (br), 1709 (s), 1608 (m), 1493 (m), 1463 (m), 1397 (m), 1333 (m), 1195 (w), 1175 (w), 1137 (s), 1106 (s), 1037 (w), 993 (m), 914 (w), 864 (w) cm^–1^. ^1^H NMR (400 MHz, CDCl_3_): δ 6.58 (s, 1H), 6.26 (s, 1H), 6.20 (m, 1H), 4.98 (m, 1H), 3.85 (s, 3H), 3.85 (s, 3H), 3.63 (s, 3H), 3.60 (s, 3H), 2.20 (s, 3H), 1.38 (s, 3H). ^13^C­{^1^H} NMR (101 MHz, CDCl_3_): δ 191.0 (s), 155.3 (s), 153.2 (s), 151.2 (s), 144.3 (s), 140.2 (s), 132.6 (s), 127.3 (s), 124.0 (s), 120.1 (s), 110.0 (s), 88.0 (s), 86.5 (s), 61.3 (s), 60.8 (s), 56.1 (s), 54.7 (s), 21.3 (s), 21.0 (s). HRMS (ESI + TOF)*m*/*z*: (M + H)+ calcd for C_19_H_23_O_6_^+^: 347.1489; found, 347.1490.

#### General Procedure for Synthesis of 2-methoxy­(2,3,4-trimethoxyphenyl)­cyclohepta-2,4,6-trien-1-ones (**5, iso-6**)

To a flame-dried microwave vial equipped with a stir bar was added the 8-oxabicyclo[3.2.1]­octa-3,6-dienone cycloadduct (1 equiv) in THF (0.2 M). The reaction vessel was purged for 5 min with argon, and a 0.1 M solution of samarium iodide in THF was added via syringe (5–6 equiv). The resulting solution was allowed to stir at room temperature for 2 min before being quenched with an equivalent volume of pH 3 phosphate buffer. The cloudy mixture was then stirred at room temperature for 3 h after which the THF was removed en vacuo. The mixture was then diluted with deionized water, extracted with Et_2_O (5×), and the combined organics washed with Rochelle’s salt (3×), water (1×), and brine (1×), then dried with Na_2_SO_4_, filtered, concentrated, and then purified via chromatography (Biotage Isolera Prime, SiliCycle SiliaSep 40 g silica gel, 40–63 μm 60 Å, solvent gradient: 0–100% acetonitrile in dichloromethane).

#### 2-Methoxy-5-(2,3,4-trimethoxy-6-methylphenyl)­cyclohepta-2,4,6-trien-1-one (**5**)

Yellow oil MB-MTC **(5)** was obtained from cycloadduct **17** (61 mg, 0.18 mmol, 1 equiv) and samarium iodide (11.0 mL, 6 equiv). *R*_f_ = 0.12 in 60% ethyl acetate/pentane. IR (thin film, KBr): 2936 (br), 2838 (w), 1624 (w), 1578 (s), 1492 (m), 1461 (m), 1399 (m), 1361 (w), 1333 (m), 1282 (w), 1241 (s), 1195 (w), 1174 (w), 1137 (m), 1114 (m), 1094 (m), 1049 (w), 1004 (w), 981 (w), 922 (w), 861 (w), 817 (w) cm^–1^. ^1^H NMR (400 MHz, CD_3_CN): δ 7.14–7.02 (m, 2H), 6.98–6.87 (m, 2H), 6.71 (s, 1H), 3.90 (s, 3H), 3.84 (s, 3H), 3.79 (s, 3H), 3.63 (s, 3H), 2.08 (s, 3H). ^*13^C­{^1^H} NMR (101 MHz, CDCl_3_): δ 180.4 (s), 164.6 (s), 153.1 (s), 151.1 (s), 140.9 (s), 140.3 (s), 138.3 (s), 136.2 (s), 134.1 (s), 131.4 (s), 129.2 (s), 112.9 (s), 109.3 (s), 61.1 (s), 56.7 (s), 56.2 (s), 20.5 (s). ^13^C­{^1^H} NMR (101 MHz, CD_3_CN): δ 180.5 (s), 165.5 (s), 154.0 (s), 151.9 (s), 141.1 (s), 138.2 (s), 136.3 (s), 134.8 (s), 132.3 (s), 130.2 (s), 113.4 (s), 110.5 (s), 61.4 (s), 61.2 (s), 56.8 (s), 56.6 (s), 20.3 (s). HRMS (ESI + TOF)*m*/*z*: (M + H)^+^ calcd for C_18_H_20_O_5_^+^: 317.1385; found, 317.1389.

#### 2-Methoxy-4-methyl-5-(2,3,4-trimethoxy-6-methylphenyl)­cyclohepta-2,4,6-trien-1-one (*iso* **-6**)

Pale yellow oil *iso* **-6** (209 mg, 50% yield) obtained from cycloadduct **18** (147 mg, 0.42 mmol, 1 equiv) and samarium iodide (25.4 mL each, 6 equiv). A Daicel IC chiral column in DCM/Acetonitrile (0–100%) was used to separate the enantiomers. *R*_f_ = 0.10 in 60% ethyl acetate/pentane. IR (thin film, KBr): 2937 (br), 1621 (w), 1575 (s), 1460 (m), 1398 (m), 1333 (s), 1266 (m), 1194 (w), 1158 (s), 1138 (s), 1099 (s), 1049 (w), 1001 (w), 921 (w), 834 (w), 729 (w) cm^–1^. ^1^H NMR (400 MHz, CDCl_3_): δ 7.11 (d, *J* = 12.7 Hz, 1H), 7.05 (d, *J* = 12.7 Hz, 1H), 6.79 (s, 1H), 6.58 (s, 1H), 3.97 (s, 3H), 3.89 (s, 3H), 3.87 (s, 3H), 3.68 (s, 3H), 2.13 (s, 3H), 1.99 (s, 3H). ^13^C­{^1^H} NMR (101 MHz, CDCl_3_): δ 179.5 (s), 162.9 (s), 153.0 (s), 150.4 (s), 142.4 (s), 141.1 (s), 140.4 (s), 136.4 (s), 133.7 (s), 130.8 (s), 129.1 (s), 117.7 (s), 109.2 (s), 61.1 (s), 61.0 (s), 56.1 (s), 56.1 (s), 26.0 (s), 19.9 (s). HRMS (ESI + TOF)*m*/*z*: (M + H)^+^ calcd for C_19_H_23_O_5_^+^: 331.1546; found, 331.1540.

#### 2-Hydroxy-4-methyl-5-(2,3,4-trimethoxyphenyl)­cyclohepta-2,4,6-trien-1-one (**19**)

In a microwave vial, *iso-* **4** (18 mg, 0.06 mmol) was dissolved in a 1:1 mixture of MeOH: HCl (2N) (3.2 mL) and the mixture was allowed to stir under reflux for 16 h. The reaction mixture was cooled to room temperature and diluted with CH_2_Cl_2_ (10 mL) and water (5 mL). The aqueous layer neutralized with 5% NaHCO_3_ (aq), and the layers were separated. The aqueous layer was further extracted with CH_2_Cl_2_ (4 × 10 mL) and the combined organic extract was dried with anhydrous Na_2_SO_4_ to give **19** as a dark brown oil (16.5 mg, 96% yield). *R*_f_ = 0.35 in 80% ethyl acetate: hexane. IR (ATR, ZnSe): 3203 (br), 2939 (w), 1598 (m), 1546 (m), 1493 (m), 1456 (m), 1441 (s), 1408 (s), 1330 (w), 1290 (m), 1259 (s), 1229 (m), 1165 (m), 1106 (m), 1088 (s), 997 (s), 815 (m) cm^–1^. ^1^H NMR (400 MHz; CDCl_3_): δ 7.42 (s, 1H), 7.29 (d, *J* = 11.5 Hz, 1H), 7.19 (d, *J* = 11.5 Hz, 1H), 6.78 (d, *J* = 8.5 Hz, 1H), 6.72 (d, *J* = 8.5 Hz, 1H), 3.91 (s, 3H), 3.90 (s, 3H), 3.66 (s, 3H), 2.22 (s, 3H). ^13^C­{^1^H} NMR (101 MHz; CDCl_3_): δ 171.2, 168.8, 153.8, 150.8, 149.0, 142.5, 140.4, 139.5, 130.3, 126.0, 124.1, 122.6, 107.6, 61.23, 61.17, 56.3, 27.0. HRMS (ESI + TOF)*m*/*z*: (M + H)^+^ calcd for C_17_H_19_O_5_^+^: 303.1233; found, 303.1233.

#### 2-Hydroxy-4-methyl-5-(2,3,4-trimethoxy-6-methylphenyl)­cyclohepta-2,4,6-trien-1-one (**20**)

In a microwave vial equipped with a stir bar was added *iso* **-6** (209 mg, 0.63 mmol) dissolved in AcOH (3 mL, 0.2 M). 12 N HCl solution (8 mL) was added, and the reaction was subjected to microwave irradiation to 100 °C for 10 h. Upon completion, the reaction was quenched with sodium carbonate (5 mL) and extracted with DCM (5 × 10 mL). The organics were combined, dried with Na_2_SO_4_, filtered, and concentrated en vacuo. The resulting oil was dissolved in 10 mL of toluene and concentrated en vacuo again. This process was repeated 5 times to remove residual acetic acid and afforded **20** as a dark brown oil (178 mg, 90% yield). *R*_f_ = 0.25 in 60% ethyl acetate/pentane. IR (thin film, KBr): 2926 (br), 2849 (w), 1591 (s), 1518 (w), 1492 (w), 1438 (s), 1399 (w), 1365 (m), 1333 (m), 1302 (m), 1270 (w), 1235 (w), 1141 (w), 1103 (w), 1083 (w) cm^–1^. ^1^H NMR (400 MHz, CDCl_3_): δ 7.44 (s, 1H), 7.22 (d, *J* = 11.6 Hz, 1H), 7.18 (d, *J* = 11.6 Hz, 1H), 6.58 (s, 1H), 3.88 (s, 3H), 3.86 (s, 3H), 3.66 (s, 3H), 2.12 (s, 3H), 1.97 (s, 3H). ^13^C­{^1^H} NMR (101 MHz, CDCl_3_): δ 171.6 (s), 168.5 (s), 153.1 (s), 150.4 (s), 149.0 (s), 140.4 (s), 140.4 (s), 138.6 (s), 130.7 (s), 129.4 (s), 125.7 (s), 123.2 (s), 109.2 (s), 61.1 (s), 60.9 (s), 56.1 (s), 26.4 (s), 20.0 (s). HRMS (ESI + TOF)*m*/*z*: (M + H)^+^ calcd for C_18_H_21_O_5_^+^: 317.1384; found, 317.1387.

#### General Procedure for Methylation of Tropolones (**4, 6**)

To a flame-dried microwave vial equipped with a stir bar was added **19** or **20** (1 equiv) CH_3_CN (0.2 M), K_2_CO_3_ (3 equiv), and dicylohexyl-18-crown-6 (0.1 equiv). The reaction vessel was sealed and purged with argon. Under argon, iodomethane was added via a syringe (5 equiv). The reaction mixture was heated at 82 °C for 24 h in an oil bath. Upon completion, the reaction mixture was diluted with DCM, washed with sodium hydroxide (2×), sodium carbonate (1×), water (1×), and brine (1×). The combined organics were dried with Na_2_SO_4_, filtered, and concentrated en vacuo. The resulting oil was then purified by chromatography (Biotage Isolera Prime, SiliCycle SiliaSep 10 g silica gel, 40–63 μm 60 Å, solvent gradient: 0–100% acetonitrile in dichloromethane [500 mL]). Product fractions were concentrated en vacuo to yield a mixture of *iso* **-4** and MT-MTC (**4**); or a mixture of *iso* **-6** and DM-MTC. A Daicel IA chiral column in 2-propanol/hexanes (10–100%) was used for the resolution of the enantiomers.

#### 2-Methoxy-6-methyl-5-(2,3,4-trimethoxyphenyl)­cyclohepta-2,4,6-trien-1-one (MT-MTC, **4**)

Off-white solid MT-MTC **(4)** (2.6 mg, 15% yield) was obtained from tropolone **19** (16 mg, 0.06 mmol, 1 equiv), K_2_CO_3_ (22.8 mg, 0.165 mmol, 3 equiv), and dicylohexyl-18-crown-6 (2 mg, 0.01 mmol, 0.10 equiv) and MeI (17.1 μL, 0.28 mmol, 5 equiv) in CH_3_CN (291 μL, 0.2 M) at 82 °C for 24 h to yield a mixture of *iso* **-4** and **4** as oil (5.9 mg, 34% combined yield). A chiral Daicel IA column in Hex/2-propanol (10–100%) was used to separate the isomers to yield **4** as a solid (2.6 mg, 15% yield). m.p. = 196–198 °C. *R*_f_ = 0.20 in 60% ethyl acetate: hexane. IR (ATR, ZnSe): 2941 (w), 2837 (w), 1659 (w), 1618 (w), 1589 (m), 1567 (s), 1491 (m), 1461 (m), 1432 (m), 1409 (s), 1301 (m), 1250 (s), 1205 (m), 1159 (m), 1093 (s), 1068 (s), 1028 (w) 1011 (m), 996 (m), 977 (m), 916 (m), 850 (m), 808 (s), 795 (m) cm^–1^. ^1^H NMR (400 MHz, CDCl_3_): δ 7.34 (d, *J* = 0.2 Hz, 1H), 6.93 (d, *J* = 10.4 Hz, 1H), 6.80 (d, *J* = 8.5 Hz, 1H), 6.71 (d, *J* = 8.6 Hz, 1H), 6.65 (d, *J* = 10.5 Hz, 1H), 3.95 (s, 3H), 3.90 (s, 6H), 3.69 (s, 3H), 2.09 (d, *J* = 0.8 Hz, 3H). ^13^C­{^1^H} NMR (101 MHz, CDCl_3_): δ 179.4 (s), 163.7 (s), 153.9 (s), 150.9 (s), 148.7 (s), 142.3 (s), 141.1 (s), 137.8 (s), 132.7 (s), 130.2 (s), 124.1 (s), 111.3 (s), 107.4 (s), 61.2 (s), 61.1 (s), 56.3 (s), 56.2 (s), 26.8 (s). HRMS (ESI + TOF)*m*/*z*: (M + H)^+^ calcd for C_18_H_20_O_5_^+^: 317.1389; found, 317.1386.

#### 2-Methoxy-6-methyl-5-(2,3,4-trimethoxy-6-methylphenyl)­cyclohepta-2,4,6-trien-1-one (DM-MTC, **6**)

DM-MTC **(6)** was obtained from a reaction between **20** (170 mg, 0.54 mmol, 1 equiv), K_2_CO_3_ (223 mg, 1.62 mmol, 3 equiv), and dicylohexyl-18-crown-6 (20 mg, 0.05 mmol, 0.10 equiv), and MeI (166 μL, 2.7 mmol, 5 equiv) in CH_3_CN (3 mL, 0.2 M), heated at 82 °C for 9 h to give a mixture of DM-MTC **(6)** and *iso* **-6** a pale yellow oil (131 mg, 74% combined yield). A chiral Diacel IA column in Hex/2-propanol (10–100%) was used to separate the DM-MTC (6) enantiomers, and *iso* **-6**. *R*_f_ = 0.10 in 60% ethyl acetate: pentane. IR (thin film, KBr): 2936 (w), 2840 (w), 1752 (w), 1620 (m), 1589 (s), 1492 (m), 1462 (m), 1400 (m), 1335 (m), 1265 (m), 1248 (s), 1195 (w), 1139 (m), 1106 (m), 1088 (m), 1028 (w), 1000 (w) cm^–1^. ^1^H NMR (400 MHz, CDCl_3_): δ 7.40 (s, 1H), 6.85 (d, *J* = 10.5 Hz, 1H), 6.68 (d, *J* = 10.5 Hz, 1H), 6.60 (s, 1H), 3.98 (s, 3H), 3.91 (s, 3H), 3.89 (s, 3H), 3.73 (s, 3H), 2.03 (s, 3H), 2.01 (s, 3H). ^13^C­{^1^H} NMR (101 MHz, CDCl_3_): δ 179.4 (s), 163.8 (s), 153.0 (s), 150.6 (s), 148.7 (s), 140.3 (s), 139.9 (s), 138.1 (s), 132.7 (s), 130.9 (s), 129.2 (s), 111.4 (s), 109.1 (s), 61.1 (s), 60.9 (s), 56.2 (s), 56.1 (s), 26.5 (s), 20.0 (s). HRMS (ESI + TOF)*m*/*z*: (M + H)^+^ calcd for C_19_H_23_O_5_^+^: 331.1546; found, 331.1540.

### Vibrational Circular Dichroism (VCD)

#### VCD Measurements

CDCl_3_ (Cambridge Isotope Laboratories Silver Foil) was run through a small plug of activated basic alumina immediately before use. To a small vial containing ∼ 7 mg of chiral molecule ((*aR*)**-6**, (*aS*)**-6**, (*aR*)-*iso* **-6**, (*aS*)-*iso*-6, (−)-colchicine or (*aR*)-isocolchicine) was added 150 μL of CDCl_3_. The resulting solution was transferred to a liquid IR cell (BaF_2_, 100 μm cell path) and placed in the measurement chamber. Experimental spectra were acquired on a BioTools, Inc. (Jupiter, FL) ChiralIR 2X Dual PEM FT-VCD spectrometer, set to 4 cm^–1^ resolution, with PEM (both 1 and 2) maximum frequency set to 1400 cm^–1^. The sample was then measured for 6 to 8 h in 1 h blocks. The IR data from the first block was solvent and water vapor subtracted, then offset to zero at 2000 cm^–1^. The VCD data blocks were averaged, and enantiomer subtracted ((E1–E2)/2). Finally, the VCD spectrum was offset to zero at 2000 cm^–1^. The VCD noise data were block averaged and used without further processing.

#### VCD Calculations

The *aR* enantiomer of each compound (**6**, *iso*-6, colchicine and isocolchicine) were constructed (separately) using ComputeVOA (BioTools, Jupiter, FL). A thorough conformational search was performed for each at the molecular mechanics level using the MMF94 force field in a 7 kcal/mol energy window. All conformers were subjected to DFT level optimization and frequency calculation with Gaussian ’09 (Wallingford, CT)^41^ at the B3LYP/6-31G­(d) and B3PW91/6-31G­(d) levels.^42^ The resulting lowest energy unique conformations were reoptimized using the cc-pVTZ/B3LYP and cc-pVTZ/B3PW91 method,^42c^ and the IR and VCD frequencies recalculated at these levels. The resulting spectra from all methods were Boltzmann averaged (using both free energy and electronic energy), plotted at 5 cm^–1^ resolution, and then *x*-axis scaled (range of 0.968 to 0.985-values obtained using CompareVOA (BioTools, Jupiter, FL) and varied with basis set and functional) for comparison to the experimental IR and VCD spectra. Two different weighing methods (free energy and electronic energy) gave consistent results for stereochemistry, with the larger cc-pVTZ basis set and electronic energy weighting giving results most similar to those of the experimental IR and VCD. These were ultimately used for similarity values and plots. The excellent visual agreement and high similarity values (IR and VCD) as well as confidence level^30^ for both **6** and *iso* **-6** leaves no doubt that the absolute configuration has been correctly assigned.

### Bioactivity Methods for Colchicine AC Analogs

#### Binding Affinity

Binding affinity to the colchicine site of bovine brain tubulin (PurSolutions, LLC (puresoluble.com)) was assayed by competition of test compounds with MDL ((E)-1-(2,5-dimethoxyphenyl)-3-[4-(dimethylamino)­phenyl]-2-methylprop-2-en-1-one) as described previously.^43^ MDL fluorescence increases many-fold upon binding to the colchicine site of tubulin. Inhibition of this fluorescence increase was measured and converted to *K*_d_ for the test compound as described.

#### Tubulin Polymerization Inhibition

Inhibition of tubulin polymerization was determined as described previously.^44^ In brief, 10 μM bovine brain tubulin (PurSolutions, LLC (puresoluble.com)) was incubated under conditions strongly promoting polymerization of tubulin (1 M NaGlutamate, 0.1 M Mes (Morpholinoethanesulfonic acid), 1 mM MgCl_2_, 0.5 mM GTP, pH 6.9), in the absence or presence of 3.3 or 33 μM test compound, incubated for 30 min at 37 °C, then centrifuged at 100,000*g* for 8 min, and the top 3/4 of the supernatant removed. Protein concentration was measured, and polymerization was determined as pelletable protein lost from the supernatant.

#### Cell-Growth Inhibition Studies

Inhibition of cell growth was determined by using standard procedures. Cell lines were obtained from the NCI anticancer drug screen and maintained in DMEM medium supplemented with 10% fetal bovine serum. Growing cells were exposed to serial dilutions of each compound for 3 days. Cell growth was determined with CellTiter Assay Reagent (Promega), and growth parameters, including inhibition of growth, were measured by using the methods specified by the manufacturer.

#### 60-Cell Screen

(a*R*)**-6**, (a*S*)**-6**, (a*S*)-*iso* **-6**, and (a*R*)-*iso* **-6** were submitted to the 60 Cell Screen panel and evaluated at 5 doses (100 μM, 10 μM, 1 μM, 0.1 μM, and 0.01 μM). A summary of the data for all molecules is in Table S5 in the Supporting Information document, and a compilation of GI_50_ values of active compounds determined from these studies along with prior experiment of colchicine is listed in Table S6. The complete report from this submission is available at the publisher’s Web site as a separate Supporting Information file. For COMPARE analysis, TGI levels of response for (*aR*)-DM-MTC (6) and (*aS*)-*iso*-DM-MTC were compared to those of several established tubulin inhibitors previously tested in the NCI-60 screen. A complete list of these data can be viewed in Table S7 in the Supporting Information document.

### Molecular Dynamics Simulations

The molecular dynamics relative binding free energy calculations^37^ were conducted using the AToM-OpenMM package version 3.5.0 ^45^ and the ATM MetaForce OpenMM plugin version 0.3.5 ^46^ and the OpenMM MD engine version 8.0.^47^ We used the 5ITZ PDB structure of the tubulin dimer bound to colchicine.^38^ The AMBER’s FF14SB force field^48^ was used for the protein receptor, and the TIP3P model was used for the water solvent. The GAFF force field was used for parametrizing ligands. Starting with the protein receptor and each ligand pair aligned in the protein binding site, the second ligand in the pair was translated by the displacement vector. The system was then solvated within a rectangle box with a 10 Å TIP3P water buffer by tleap from AmberTools. Potassium and chloride ions were added to neutralize the system, if needed. Relative binding free energy calculations employed 22 alchemical replicas, each simulated for 40 ns. Additional molecular dynamics runs of the complexes to collect the dihedral angle probability distributions were run for 100 ns.^49^

## Supplementary Material









## Data Availability

The data underlying this study are available in the published article and its Supporting Information.

## Notes

^1^H and ^13^C NMR spectra of all newly synthesized compounds; data on experimental rotational barrier determination; computational data associated with rotational barrier; details on biological, biochemical, and alchemical studies,are included in primary supporting information file named jo5c00284_si_001.pdf; (PDF)
A full listing of reports of NCI data is included in a pdf file named jo5c00284_si_002.pdf (PDF)
A full list of VCD-associated data, including the complete report as provided by Biotools, and mol files, are included in a zip file named jo5c00284_si_003.zip­(ZIP)
A full listing of cartesian coordinates for key structures (ground state and transition states) are provided as .xyz files in a zip file named jo5c00284_si_004.zip­(ZIP)
