# Supplementary material for: Deconstruction of Desacetamidocolchicine’s B Ring Reveals a Class 3 Atropisomeric AC Ring with Tubulin Binding Properties
Source: J Org Chem. 2025 May 27;90(22):7246–58. doi: 10.1021/acs.joc.5c00284 (PMC12150326; doi:10.1021/acs.joc.5c00284)
Supplement: Supplementary file 1 [file jo5c00284_si_001.pdf]

Deconstruction of Desacetamidocolchicine's B Ring Reveals a Class 3 Atropisomeric AC Ring with Tubulin Binding Properties

Lauren P. Bejcek,[a,b,‡] Orugbani S. Eli,[a,b,‡] Diana M. Kapkayeva,[a] Jordan Nafie,[c] John A. Beutler,[d] Emilio Gallicchio,[a,b,e] Dan L. Sackett,[f] Ryan P. Murelli *[a,b,e]

[a]*PhD Program in Chemistry, The Graduate Center, The City University of New York, New York, NY, USA*

[b]*Department of Chemistry and Biochemistry, Brooklyn College, The City University of New York, Brooklyn, New York, USA*

[c]*Biotools, Inc., 17546 Bee Line Highway, Jupiter, FL, USA*

[d]*Molecular Targets Program, National Cancer Institute, National Institute of Health, Frederick, MD, USA*

[e]*PhD Program in Biochemistry, The Graduate Center, The City University of New York, New York, NY, USA*

[f]*Division of Basic and Translational Biophysics, Eunice Kennedy Shriver National Institute of Child Health and Human Development, National Institutes of Health, Bethesda, MD, USA*

Supporting Information

Table of Contents

I. General Information……………………………………………………………..s-3

II. Synthesis and Characterization…………………………………………………...s-3

Oxidopyrylium Triflate Salts **(9 – 10)**……………………………………………………s-3

2-ethynyl-2,3,4-trimethoxy-1-methylbenzene **(14)**………...…………………...………………...…s-3

8-oxabicyclo[3.2.1]octa-3,6-dienones **(15 – 18)**…………………………………...………...……s-4

2-methoxy(2,3,4-trimethoxyphenyl)cyclohepta-2,4,6-trien-1-ones (**5** & ***iso*-6**)……………………..…s-5

2-hydroxy(2,3,4-trimethoxyphenyl)cyclohepta-2,4,6-trienones **(19 – 20)** …………………………...…..s-7

MT-MTC **(4)** and DM-MTC **(6)**……………..………………………………………...…..s-8

III. DFT Calculations…………………...…………………………...……...……….s-10

Torsional Angle Profiling……………………………………..……….…………..………...s-10

Table S1……………………………………..………………………..…………..s-11

Free Energy Barrier Determination……………………………………………………………s-11

Table S2……………………………………...…………….…..……………….s-12

Table S3……………………………………...…………….…..……………….s-12

Ground State Profiling…………………………………………………………………………...s-12

Figure S1…………………………………………………………………...…….s-13

Table S4……………………………………...………..……………………….s-13

Molecular Dynamics Simulation……………………………………………………………………s-13

IV. Experimental Profiling of Dihedral Angles……………………......……......………….s-14

Rotational Energy Barrier Determination……………………………………………………..s-14

Table S5……………………………………………………………………………………..s-15
Vibrational Circular Dichroism…………………………………………………………………..s-16
V. Bioactivity Methods for Colchicine AC Analogs…………………......……......…………s-16
60 Cell Screen…………………………..………………………………………..……..……………s-16
Table S6………………………………………………………...…………….…………………s-17
Table S7………………………………………………………...………………..……………s-17
Table S8………………………………………………………...…………..…………………s-18
VI. HPLC Chromatograms…...……………………………………………..………………....s-19
VII. NMR Spectra……………..……………………………………………..………………....s-23
References……………………………………………………………………………………..s-49

I. General Information

All starting materials and reagents were purchased from commercially available sources and used without further purification, except for CH_2Cl_2 and benzene, which were purified on a solvent purification system prior to the reaction. ^{1}H NMR shifts are measured using the solvent residual peak as the internal standard ($CHCl_3$ δ 7.26, D_2O δ 4.79), and reported as follows: chemical shift, multiplicity (s = singlet, bs = broad singlet, d = doublet, t = triplet, dd = doublet of doublet, q = quartet, m = multiplet), coupling constant (Hz), and integration. ^{13}C NMR shifts are measured using the solvent residual peak as the internal standard ($CDCl_3$ δ 77.20) and reported as chemical shifts. Infrared (IR) spectral bands are characterized as broad (br), strong (s), medium (m), and weak (w). Microwave reactions were performed via Biotage Initiator 2.5. Purification via reverse phase column chromatography was performed on the Biotage Isolera Prime, with Biotage SNAP 12g cartridges, in a solvent system of acetonitrile in water, each solvent containing 0.05% trifluoroacetic acid (TFA). Oxidopyrilium dimers **(11-12)**[39] alkyne **13**, cycloadducts **15** and **16,** and methoxytropones **3, 4,** and ***iso*-4** were synthesized as previously reported.[14]

II. Synthesis and Characterization

Oxidopyrilium Triflate Salts (9 – 10)[39]

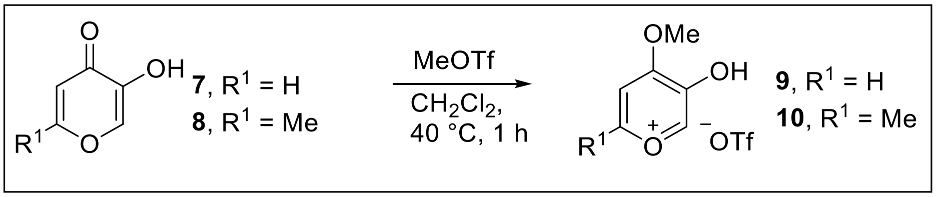

General Procedure: The oxidopyrylium salts were prepared as described previously.[14,39] To a solution of 3-hydroxy-4-pyrone derivative **7** or **8** in CH_2Cl_2 was added methyl trifluoromethanesulfonate (MeOTf). The mixture was stirred under reflux for an hour, cooled to room temperature, and then evaporated under reduced pressure to yield the crude oil. Crystallization from ethyl acetate (EtOAc) yielded pure solids **9** and **10**. Characterization data for triflate salts **9** and **10** have been previously reported.[14]

2-ethynyl-2,3,4-trimethoxy-1-methylbenzene (14)

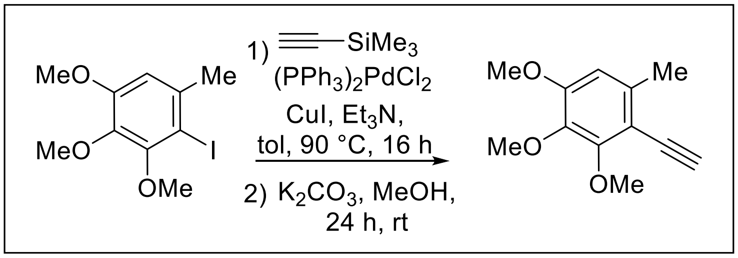

To a flame-dried round bottom flask equipped with a stir bar was added 2-iodo-3,4,5-trimethoxy-1-methylbenzene (5g, 16 mmol) followed by bis(triphenylphosphine)palladium chloride (570 mg, 0.81 mmol) and copper iodide (155 mg, 0.81 mmol). The reaction vessel was sealed and purged with argon. Toluene (15 mL) and triethylamine (20 mL) were then added to the reaction and argon bubbled through the resulting solution for approximately 10 minutes. Trimethylsilyl (TMS) acetylene (3.47 mL, 24.3 mmol) was added via syringe and then the reaction was heated at 90 °C for 16 hours. The solution was then cooled to room temperature, filtered through celite, and concentrated under reduced pressure. The resulting 6.37 grams of oil was then dissolved in 20 mL of methanol and 15 grams potassium carbonate was added. The mixture was stirred at room temp for approximately 24 hours, diluted with CH_2Cl_2 (20 mL), and washed with water (2 x 20 mL). The organic layer was dried over Na_2SO_4, filtered, and then concentrated under reduced pressure. The resulting oil was purified by chromatography (Biotage Isolera Prime, SiliCycle Silia*Sep* 40 g silica gel, 40-63 μm 60 Å, solvent gradient: 0-100% EtOAc in hexanes (500 mL), and concentrated to reveal **14** as a pale yellow solid (3.2 g, 96 % yield). **m.p.** = 52 – 53 °C. $\mathbf{R_f}$ = 0.94, 60% EtOAc/pentane. **IR (thin film, KBr):** 3273 (br), 2937 (m), 1596 (m), 1493 (m), 1463 (w), 1400 (w), 1335 (s), 1246 (m), 1124 (s), 1077 (w), 1034 (w) cm^{-1}. **^{1}H NMR (400 MHz, $CDCl_3$)** δ 6.50 (s, 1H), 3.94 (s, 3H), 3.83 (s, 3H), 3.82 (s, 3H), 3.40 (s, 1H), 2.37 (s, 3H). **^{13}C{^{1}H} NMR (101 MHz, $CDCl_3$)** δ 155.3 (s), 153.8 (s), 139.8 (s), 137.5 (s), 109.1 (s), 108.6 (s), 83.7 (s), 78.6 (s), 61.1 (s), 61.0 (s), 55.9 (s), 20.7 (s). **HRMS (ESI+ TOF) *m/z*:** $(M+H)^+$ Calc'd for $C_{12}H_{15}O_3^+$: 207.1017. Found: 207.1016.

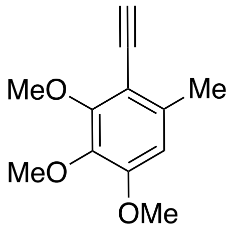

8-oxabicyclo[3.2.1]octa-3,6-dienones (15 – 18)

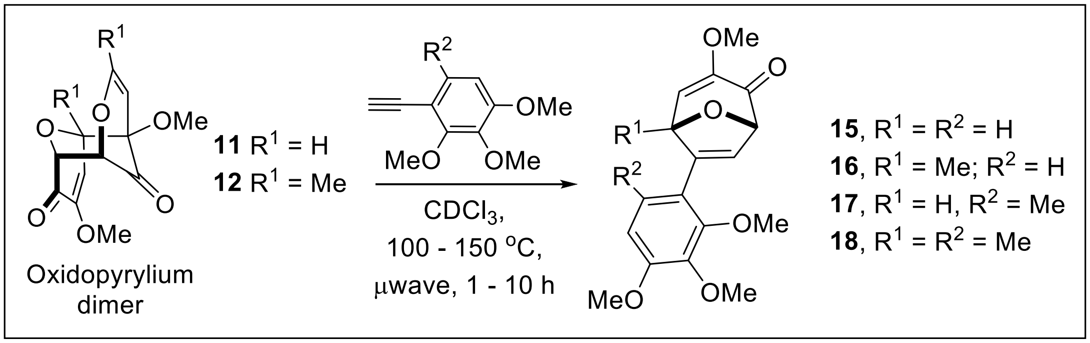

General Procedure: To a solution of oxidopyrylium dimer (1 eq.) in $CDCl_3$ (0.2 M) in a microwave vial was added (10 – 17 eq.). The reaction was subjected to microwave irradiation at 120 °C – 150 °C for 1 to 10 hours. The resulting solution was immediately subjected to purification via column chromatography (Biotage Isolera Prime, SiliCycle SiliaSep 10 g silica gel, 40-63 μm 60 Å, solvent gradient: 0-100% EtOAc in hexanes (500 mL).

Cycloadducts **15** and **16** were synthesized according to the general procedure, and the characterization data reported in reference.[14]

(±)-(1S,5S)-3-methoxy-6-(2,3,4-trimethoxy-6-methylphenyl)-8-oxabicyclo[3.2.1]octa-3,6-dien-2-one (17)

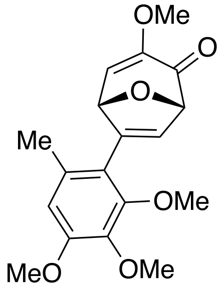

Yellow oil **17** (73 mg, 42% yield) obtained from oxidopyrylium dimer **11** (132 mg, 0.52 mmol, 1 eq.) alkyne **14** (2 g, 9.70 mmol, 17 eq.) at 150 °C for 40 minutes. **Rf** = 0.83 in 60 % ethyl acetate/pentane. **IR (thin film, KBr):** 2937 (w), 2838 (w), 1712 (s), 1610 (m), 1494 (m), 1463 (m), 1397 (m), 1334 (w), 1195 (s), 1136 (s), 1097 (s), 1043 (w), 992 (m), 918 (w), 896 (w), 839 (w) cm^{-1}. **^{1}H NMR (400 MHz, CD_3CN)** δ 6.68 (s, 1H), 6.29 – 6.20 (m, 2H), 5.36 (d, J = 4.9 Hz, 1H), 4.98 (d, J = 2.5 Hz, 1H), 3.82 (s, 3H), 3.78 (s, 3H), 3.76 (s, 3H), 3.50 (s, 3H), 2.16 (s, 3H). **$^{13}C\{^1H\}$ NMR (101 MHz, CD_3CN)** δ 191.0 (s), 154.4 (s), 153.6 (s), 152.7 (s), 147.3 (s), 141.0 (s), 133.2 (s), 126.0 (s), 120.1 (s), 117.9 (s), 110.8 (s), 88.6 (s), 82.36 (s), 61.7 (s), 61.2 (s), 56.6 (s), 55.2 (s), 20.8 (s). **HRMS (ESI+ TOF) *m/z*:** $(M+H)^+$ Calc'd for $C_{18}H_{20}O_6^+$: 333.1338. Found: 333.1341.

(±)-(1*S*,5*S*)-3-methoxy-5-methyl-6-(2,3,4-trimethoxy-6-methylphenyl)-8-oxabicyclo[3.2.1]octa-3,6-dien-2-one (18)

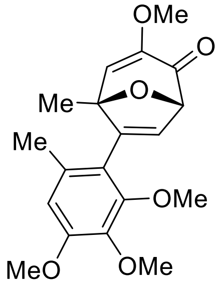

Yellow oil **18** (456 mg, 52% yield) obtained from oxidopyrylium dimer **12** (362 mg, 1.28 mmol, 1 eq.) and alkyne **14** (3.2 g, 15.4 mmol, 12 eq.) at 120 °C for 2 hours. **R_f** = 0.87 in 60 % ethyl acetate/pentane. **IR (thin film, KBr):** 2937 (br), 1709 (s), 1608 (m), 1493 (m), 1463 (m), 1397 (m), 1333 (m), 1195 (w), 1175 (w), 1137 (s), 1106 (s), 1037 (w), 993 (m), 914 (w), 864 (w) cm^{-1}. **^{1}H NMR (400 MHz, $CDCl_3$)** δ 6.58 (s, 1H), 6.26 (s, 1H), 6.20 (m, 1H), 4.98 (m, 1H), 3.85 (s, 3H), 3.85 (s, 3H), 3.63 (s, 3H), 3.60 (s, 3H), 2.20 (s, 3H), 1.38 (s, 3H). **$^{13}C\{^1H\}$ NMR (101 MHz, $CDCl_3$)** δ 191.0 (s), 155.3 (s), 153.2 (s), 151.2 (s), 144.3 (s), 140.2 (s), 132.6 (s), 127.3 (s), 124.0 (s), 120.1 (s), 110.0 (s), 88.0 (s), 86.5 (s), 61.3 (s), 60.8 (s), 56.1 (s), 54.7 (s), 21.3 (s), 21.0 (s). **HRMS (ESI+ TOF) *m/z*:** (M+H)+ Calc'd for $C_{19}H_{23}O_6^+$: 347.1489. Found: 347.1490.

2-methoxy(2,3,4-trimethoxyphenyl)cyclohepta-2,4,6-trien-1-ones (5 & *iso*-6)

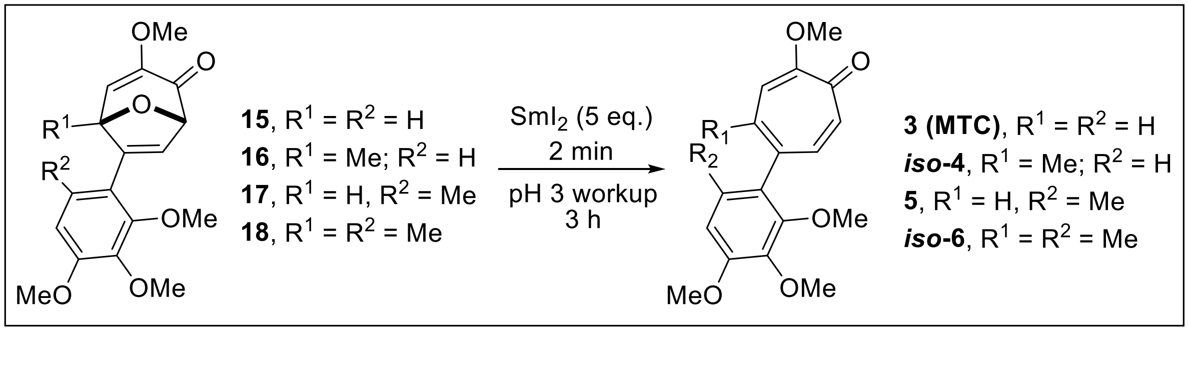

General Procedure: To a flame-dried microwave vial equipped with a stir bar, was added the 8-oxabicyclo[3.2.1]octa-3,6-dienone cycloadduct (1 eq) in THF (0.2 M). The reaction vessel was purged for 5 minutes with argon, and a 0.1 M solution of samarium iodide in THF was added via syringe (5 - 6 eq.). The resulting solution was allowed to stir at room temperature for 2 minutes before quenched with an equivalent volume of pH 3 phosphate buffer. The cloudy mixture was then stirred at room temperature for 3 hours after which the THF was removed *en vacuo*. The mixture was then diluted with deionized water, extracted with Et_2O (5x), and the combined organics washed with Rochelle's salt (3x), water (1x), and brine (1x), then dried with Na_2SO_4, filtered, and concentrated, and then purified via chromatography. (Biotage Isolera Prime, SiliCycle SiliaSep 40 g silica gel, 40-63 μm 60 Å, solvent gradient: 0-100% acetonitrile in dichloromethane (500 mL).

Cycloheptatrienones **3 (MTC)** and ***iso*-4** were synthesized according to the general procedure, and characterization data reported in reference.[14]

2-methoxy-5-(2,3,4-trimethoxy-6-methylphenyl)cyclohepta-2,4,6-trien-1-one (5)

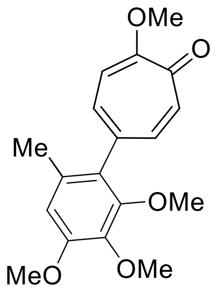

Yellow oil MB-MTC **(5)** was obtained from cycloadduct **17** (61 mg, 0.18 mmol, 1 eq.) and samarium iodide (11.0 mL, 6 eq.). **R_f** = 0.12 in 60 % ethyl acetate/pentane. **IR (thin film, KBr):** 2936 (br), 2838 (w), 1624 (w), 1578 (s), 1492 (m), 1461 (m), 1399 (m), 1361 (w), 1333 (m), 1282 (w), 1241 (s), 1195 (w), 1174 (w), 1137 (m), 1114 (m), 1094 (m), 1049 (w), 1004 (w), 981 (w), 922 (w), 861 (w), 817 (w) cm^{-1}. **^{1}H NMR (400 MHz, CD_3CN)** δ 7.14 – 7.02 (m, 2H), 6.98 – 6.87 (m, 2H), 6.71 (s, 1H), 3.90 (s, 3H), 3.84 (s, 3H), 3.79 (s, 3H), 3.63 (s, 3H), 2.08 (s, 3H). ***^{13}C{^{1}H} NMR (101 MHz, $CDCl_3$)** δ 180.4 (s), 164.6 (s), 153.1 (s), 151.1 (s), 140.9 (s), 140.3 (s), 138.3 (s), 136.2 (s), 134.1 (s), 131.4 (s), 129.2 (s), 112.9 (s), 109.3 (s), 61.1 (s), 56.7 (s), 56.2 (s), 20.5 (s). **^{13}C{^{1}H} NMR (101 MHz, CD_3CN)** δ 180.5 (s), 165.5 (s), 154.0 (s), 151.9 (s), 141.1 (s), 138.2 (s), 136.3 (s), 134.8 (s), 132.3 (s), 130.2 (s), 113.4 (s), 110.5 (s), 61.4 (s), 61.2 (s), 56.8 (s), 56.6 (s), 20.3 (s). **HRMS (ESI+ TOF) *m/z*:** $(M+H)^+$ Calc'd for $C_{18}H_{20}O_5^+$: 317.1385. Found: 317.1389.

2-methoxy-4-methyl-5-(2,3,4-trimethoxy-6-methylphenyl)cyclohepta-2,4,6-trien-1-one (*iso*-6)

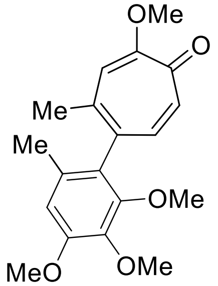

Pale yellow oil ***iso*-6** (209 mg, 50% yield) obtained from cycloadduct **18** (147 mg, 0.42 mmol, 1 eq.) and samarium iodide (25.4 mL each, 6 eq.). A Daicel IC chiral column in DCM/Acetonitrile (0 – 100%) was used to separate the enantiomers. **R_f** = 0.10 in 60 % ethyl acetate/pentane. **IR (thin film, KBr):** 2937 (br), 1621 (w), 1575 (s), 1460 (m), 1398 (m), 1333 (s), 1266 (m), 1194 (w), 1158 (s), 1138 (s), 1099 (s), 1049 (w), 1001 (w), 921 (w), 834 (w), 729 (w) cm^{-1}. **^{1}H NMR (400 MHz, $CDCl_3$)** δ 7.11 (d, J = 12.7 Hz, 1H), 7.05 (d, J = 12.7 Hz, 1H), 6.79 (s, 1H), 6.58 (s, 1H), 3.97 (s, 3H), 3.89 (s, 3H), 3.87 (s, 3H), 3.68 (s, 3H), 2.13 (s, 3H), 1.99 (s, 3H). **^{13}C{^{1}H} NMR (101 MHz, $CDCl_3$)** δ 179.5 (s), 162.9 (s), 153.0 (s), 150.4 (s), 142.4 (s), 141.1

(s), 140.4 (s), 136.4 (s), 133.7 (s), 130.8 (s), 129.1 (s), 117.7 (s), 109.2 (s), 61.1 (s), 61.0 (s), 56.1 (s), 56.1 (s), 26.0 (s), 19.9 (s). **HRMS (ESI+TOF) *m/z*:** $(M+H)^+$ Calc'd for $C_{19}H_{23}O_5^+$: 331.1546. Found: 331.1540.

2-hydroxy(2,3,4-trimethoxyphenyl)cyclohepta-2,4,6-trienones (19 – 20)

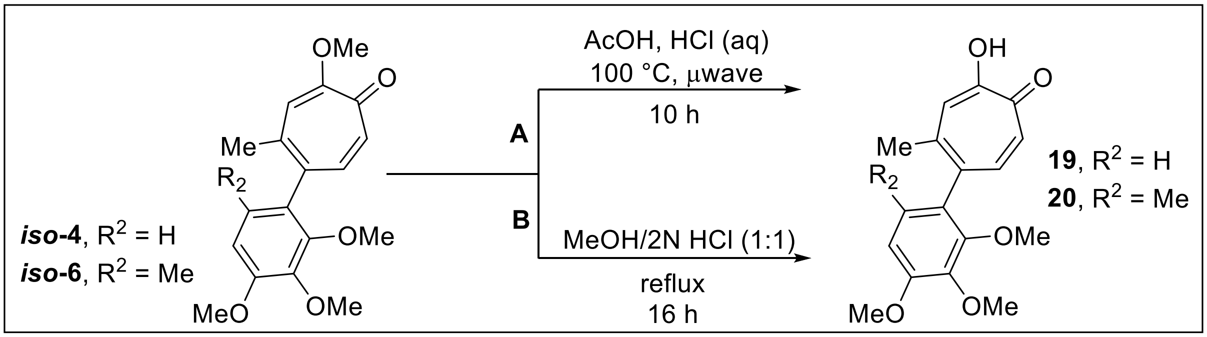

2-hydroxy-4-methyl-5-(2,3,4-trimethoxyphenyl)cyclohepta-2,4,6-trien-1-one (19)

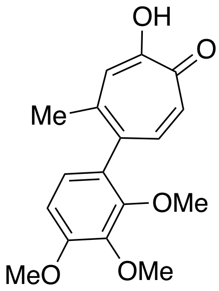

In a microwave vial, ***iso*-4** (18 mg, 0.06 mmol) was dissolved in a 1:1 mixture of MeOH:HCl (2N) (3.2 mL) and the mixture stirred under reflux for 16 hours. The reaction was let to cool to room temperature and diluted with CH_2Cl_2 (10 mL) and water (5 mL). The aqueous layer neutralized with 5% $NaHCO_3$ (aq.) and the layers were separated. The aqueous layer was further extracted four times with CH_2Cl_2 (4 x 10 mL), and the combined organic extract was dried with anhydrous Na_2SO_4 to give **19** as a dark brown oil (16.5 mg, 96% yield). $\mathbf{R_f}$ = 0.35 in 80 % ethyl acetate: hexane. **IR (ATR, ZnSe):** 3203 (br), 2939 (w), 1598 (m), 1546 (m), 1493 (m), 1456 (m), 1441 (s), 1408 (s), 1330 (w), 1290 (m), 1259 (s), 1229 (m), 1165 (m), 1106 (m), 1088 (s), 997 (s), 815 (m) cm^{-1}. **^{1}H-NMR (400 MHz; $CDCl_3$)** δ 7.42 (s, 1H), 7.29 (d, J = 11.5 Hz, 1H), 7.19 (d, J = 11.5 Hz, 1H), 6.78 (d, J = 8.5 Hz, 1H), 6.72 (d, J = 8.5 Hz, 1H), 3.91 (s, 3H), 3.90 (s, 3H), 3.66 (s, 3H), 2.22 (s, 3H). **^{13}C{^{1}H} NMR (101 MHz; $CDCl_3$)** δ 171.2, 168.8, 153.8, 150.8, 149.0, 142.5, 140.4, 139.5, 130.3, 126.0, 124.1, 122.6, 107.6, 61.23, 61.17, 56.3, 27.0. **HRMS (ESI+ TOF) *m/z*:** $(M+H)^+$ Calc'd for $C_{17}H_{19}O_5^+$: 303.1233. Found: 303.1233.

2-hydroxy-4-methyl-5-(2,3,4-trimethoxy-6-methylphenyl)cyclohepta-2,4,6-trien-1-one (20)

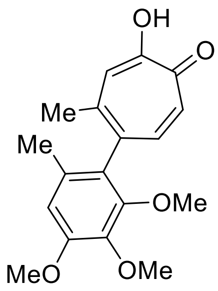

In a microwave vial equipped with a stir bar was added ***iso*-6** (209 mg, 0.63 mmol) dissolved in AcOH (3 mL, 0.2 M). 8 mL of 12N HCl solution was added, and the reaction was subjected to microwave irradiation to 100 °C for 10 hours. Upon completion, the reaction was quenched with 5 mL sodium carbonate and extracted with DCM (5 x 10 mL). The organics were combined, dried with Na_2SO_4, filtered, and concentrated *en vacuo*. The resulting oil was dissolved in 10 mL of toluene and concentrated *en vacuo* again. This process was repeated 5 times to remove residual acetic acid, and afforded **20** as a dark brown oil (178 mg, 90% yield). $\mathbf{R_f}$ = 0.25 in 60 % ethyl acetate/pentane. **IR (thin film, KBr):** 2926 (br), 2849 (w), 1591 (s), 1518

(w), 1492 (w), 1438 (s), 1399 (w), 1365 (m), 1333 (m), 1302 (m), 1270 (w), 1235 (w), 1141 (w), 1103 (w), 1083 (w) cm^{-1}. **^{1}H NMR (400 MHz, $CDCl_3$)** δ 7.44 (s, 1H), 7.22 (d, *J* = 11.6 Hz, 1H), 7.18 (d, *J* = 11.6 Hz, 1H), 6.58 (s, 1H), 3.88 (s, 3H), 3.86 (s, 3H), 3.66 (s, 3H), 2.12 (s, 3H), 1.97 (s, 3H). **$^{13}C\{^1H\}$ NMR (101 MHz, $CDCl_3$)** δ 171.6 (s), 168.5 (s), 153.1 (s), 150.4 (s), 149.0 (s), 140.4 (s), 140.4 (s), 138.6 (s), 130.7 (s), 129.4 (s), 125.7 (s), 123.2 (s), 109.2 (s), 61.1 (s), 60.9 (s), 56.1 (s), 26.4 (s), 20.0 (s). **HRMS (ESI+ TOF) *m/z*:** $(M+H)^+$ Calc'd for $C_{18}H_{21}O_5^+$: 317.1384. Found: 317.1387.

MT-MTC (4) and DM-MTC (6)

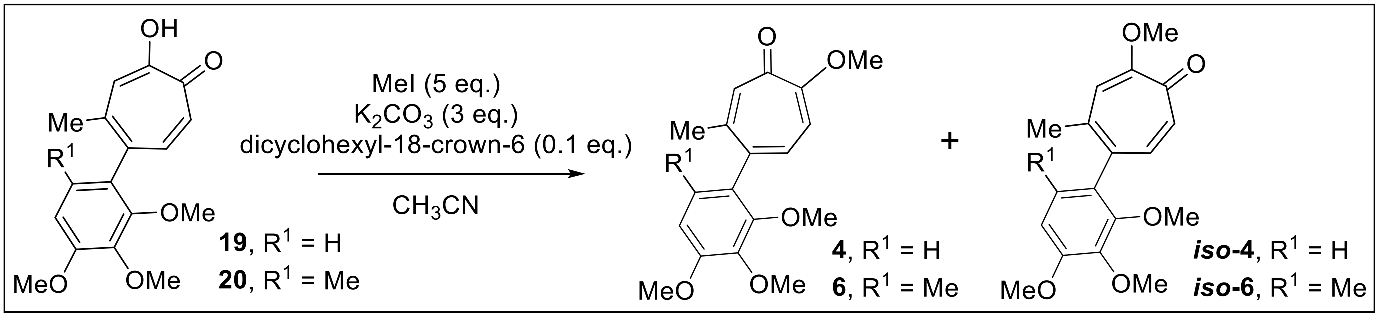

General Procedure: To a flame-dried microwave vial equipped with a stir bar was added **19** or **20** (1 eq.) in CH_3CN (0.2 M), K_2CO_3 (3 eq.), and dicylohexyl-18-crown-6 (0.1 eq.). The reaction vessel is sealed and purged with argon. Under argon, iodomethane is added via a syringe (5 eq.). The reaction mixture is heated at 82 °C for 24 hours in an oil bath. Upon completion, the reaction mixture is diluted with DCM, washed with sodium hydroxide (2x), sodium carbonate (1x), water (1x), and brine (1x). The combined organics are dried with Na_2SO_4, filtered, and concentrated *en vacuo*. The resulting oil is then purified by chromotagraphy (Biotage Isolera Prime, SiliCycle SiliaSep 10 g silica gel, 40-63 μm 60 Å, solvent gradient: 0-100% acetonitrile in dichloromethane (500 mL). Product fractions were concentrated *en vacuo* to yield a mixture of ***iso*-4** and **MT-MTC (4)**; or a mixture of ***iso*-6** and **DM-MTC**. A Daicel IA chiral column in 2-propanol/hexanes (10-100%) was used for resolution of the enantiomers.

2-methoxy-6-methyl-5-(2,3,4-trimethoxyphenyl)cyclohepta-2,4,6-trien-1-one (MT-MTC (4))

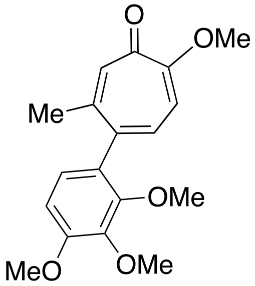

Off-white solid MT-MTC **(4)** (2.6 mg, 15% yield) was obtained from tropolone **19** (16 mg, 0.06 mmol, 1 eq.), K_2CO_3 (22.8 mg, 0.165 mmol, 3 eq.), and dicylohexyl-18-crown-6 (2 mg, 0.01 mmol, 0.10 eq.) and MeI (17.1 μL, 0.28 mmol, 5 eq.) in CH_3CN (291 μL, 0.2 M) at 82 °C for 24 hours to yield a mixture of ***iso*-4** and **4** as oil (5.9 mg, 34% combined yield). A chiral Daicel IA column in Hex/2-propanol (10 – 100%) was used to separate the isomers to yield **4** as a solid (2.6 mg, 15% yield). **m.p.** = 196 – 198 °C. **R_f** = 0.20 in 60 % ethyl acetate: hexane. **IR (ATR, ZnSe):** 2941 (w), 2837 (w), 1659 (w), 1618 (w), 1589 (m), 1567 (s), 1491 (m), 1461 (m), 1432 (m), 1409 (s), 1301 (m), 1250 (s), 1205 (m), 1159 (m), 1093 (s), 1068 (s), 1028 (w) 1011 (m), 996 (m), 977 (m), 916 (m), 850 (m), 808 (s), 795 (m) cm^{-1}. **^{1}H NMR (400 MHz, $CDCl_3$)**

δ 7.34 (d, *J* = 0.2 Hz, 1H), 6.93 (d, *J* = 10.4 Hz, 1H), 6.80 (d, *J* = 8.5 Hz, 1H), 6.71 (d, *J* = 8.6 Hz, 1H), 6.65 (d, *J* = 10.5 Hz, 1H), 3.95 (s, 3H), 3.90 (s, 6H), 3.69 (s, 3H), 2.09 (d, *J* = 0.8 Hz, 3H). **^{13}C{^{1}H} NMR (101 MHz, $CDCl_3$)** δ 179.4 (s), 163.7 (s), 153.9 (s), 150.9 (s), 148.7 (s), 142.3 (s), 141.1 (s), 137.8 (s), 132.7 (s), 130.2 (s), 124.1 (s), 111.3 (s), 107.4 (s), 61.2 (s), 61.1 (s), 56.3 (s), 56.2 (s), 26.8 (s). **HRMS (ESI+ TOF) *m/z*:** $(M+H)^+$ Calc'd for $C_{18}H_{20}O_5^+$: 317.1389. Found: 317.1386.

2-methoxy-6-methyl-5-(2,3,4-trimethoxy-6-methylphenyl)cyclohepta-2,4,6-trien-1-one (DM-MTC, (6))

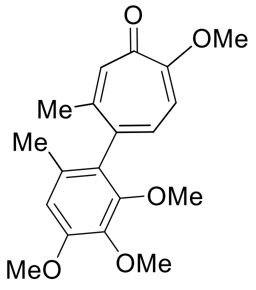

DM-MTC **(6)** was obtained from a reaction between **20** (170 mg, 0.54 mmol, 1 eq), K_2CO_3 (223 mg, 1.62 mmol, 3 eq), and dicylohexyl-18-crown-6 (20 mg, 0.05 mmol, 0.10 eq), and MeI (166 µL, 2.7 mmol, 5 eq) in CH_3CN (3mL, 0.2 M), heated at 82 °C for 9 hours to give a mixture of DM-MTC **(6)** and ***iso*-6** a pale yellow oil (131 mg, 74% combined yield). A chiral Diacel IA column in Hex/2-propanol (10-100%) was used to separate the **DM-MTC (6)** enantiomers, and ***iso*-6**. **R_f** = 0.10 in 60 % ethyl acetate: pentane. **IR (thin film, KBr):** 2936 (w), 2840 (w), 1752 (w), 1620 (m), 1589 (s), 1492 (m), 1462 (m), 1400 (m), 1335 (m), 1265 (m), 1248 (s), 1195 (w), 1139 (m), 1106 (m), 1088 (m), 1028 (w), 1000 (w) cm^{-1}. **^{1}H NMR (400 MHz, $CDCl_3$)** δ 7.40 (s, 1H), 6.85 (d, *J* = 10.5 Hz, 1H), 6.68 (d, *J* = 10.5 Hz, 1H), 6.60 (s, 1H), 3.98 (s, 3H), 3.91 (s, 3H), 3.89 (s, 3H), 3.73 (s, 3H), 2.03 (s, 3H), 2.01 (s, 3H). **^{13}C{^{1}H} NMR (101 MHz, $CDCl_3$)** δ 179.4 (s), 163.8 (s), 153.0 (s), 150.6 (s), 148.7 (s), 140.3 (s), 139.9 (s), 138.1 (s), 132.7 (s), 130.9 (s), 129.2 (s), 111.4 (s), 109.1 (s), 61.1 (s), 60.9 (s), 56.2 (s), 56.1 (s), 26.5 (s), 20.0 (s). **HRMS (ESI+TOF) *m/z*:** $(M+H)^+$ Calc'd for $C_{19}H_{23}O_5^+$: 331.1546. Found: 331.1540.

III. DFT Calculations

General Information for DFT Calculations. Computational modeling was carried out using Schrödinger Suite's Jaguar software. Unless otherwise noted, all calculations were performed using the M06-2X functional and the 6-311G** basis set in the gas phase at room temperature (298.15 K). This functional and basis set was chosen for its cited accuracy for both conformational and energetic accuracy.[41]

Torsional Angle Profiling. AC rings structures (**3-6**) were submitted to a geometry optimization to provide a preliminary ground-state conformation. These structures were used to determine ground state dihedral angles provided in **Scheme 1A**. These were then submitted to a relaxed coordination scan of dihedral χ in increments of 10º - scanned in an iterative rather than simultaneous fashion by selecting 'previously optimized geometry' - to a final dihedral angle of '540º'. This was done in order to obtain a full and iterative 360º window for each compound (180º to 540º) and this full window is provided in **Scheme 1B**, adjusted to -180º to 180º, and plotted against change in energy (kcal/mol).

These numbers are provided in the following table (**Table S1**):

dihedral angle (°)	Absolute Energy (au) (M06-2X/6-311G**)					ΔE (kcal/mol)				
	3 (MTC)	5	MT-MTC (4)	DM-MTC (6)	*iso*-6	MTC	5	MTMTC	DMMTC	*iso*-6
129.1	-1034.512288					0.6965				
120		-1073.817566					0.78814			
112.9			-1073.816705					1.74069		
101.1				-1113.123975					1.728762	
108.5					-1113.1233					1.379245
110				-1113.124397	-1113.1233				1.463957	1.37046
120		-1073.817563	-1073.817607	-1113.123534	-1113.1226		0.79002	1.17468	2.00549	1.7952775
130	-1034.512291	-1073.817069	-1073.816442	-1113.121129	-1113.1202	0.6946	1.10001	1.90572	3.514627	3.33579
140	-1034.51127	-1073.81529	-1073.813899	-1113.117299	-1113.1155	1.3353	2.21633	3.50145	5.917952	6.246135
150	-1034.509741	-1073.811903	-1073.810584	-1113.111629	-1113.1085	2.2948	4.34167	5.58161	9.475877	10.658715
160	-1034.506764	-1073.806811	-1073.805642	-1113.10505	-1113.099	4.1628	7.5369	8.68272	13.6042	16.64632
170	-1034.502641	-1073.800022	-1073.799474	-1113.096755	-1113.0873	6.75	11.797	12.5531	18.80931	23.959205
180	-1034.501783	-1073.792288	-1073.792729	-1113.087939	-1113.0734	7.2884	16.6501	16.7856	24.34135	32.661375
190	-1034.505201	-1073.801624	-1073.786825	-1113.081474	-1113.0725	5.1436	10.7917	20.4904	28.39814	33.2405575
200	-1034.508543	-1073.808306	-1073.791846	-1113.108145	-1113.1036	3.0465	6.59879	17.3397	11.66209	13.72719
210	-1034.511427	-1073.813331	-1073.805756	-1113.115423	-1113.1119	1.2368	3.4456	8.61118	7.095142	8.540275
220	-1034.512972	-1073.816628	-1073.814343	-1113.120605	-1113.1184	0.2673	1.37673	3.22284	3.843437	4.45776
230	-1034.513398	-1073.818352	-1073.813244	-1113.123976	-1113.1224	0	0.29492	3.91246	1.728135	1.90258
240	-1034.512867	-1073.818822	-1073.819162	-1113.12594	-1113.1245	0.3332	0	0.19892	0.495725	0.5867125
250	-1034.511745	-1073.818554	-1073.819479	-1113.12673	-1113.1253	1.0373	0.16817	0	0	0.116715
260	-1034.510427	-1073.817905	-1073.818895	-1113.126487	-1113.1254	1.8643	0.57542	0.36646	0.152482	0.0784375
270	-1034.509471	-1073.817319	-1073.818028	-1113.126234	-1113.1244	2.4642	0.94313	0.9105	0.31124	0.70782
280	-1034.509175	-1073.817052	-1073.817113	-1113.12537	-1113.1233	2.6499	1.11067	1.48466	0.8534	1.359165
290	-1034.509683	-1073.816955	-1073.81612	-1113.124212	-1113.1241	2.3312	1.17154	2.10777	1.580045	0.8640675
300	-1034.510796	-1073.816816	-1073.815333	-1113.122767	-1113.1233	1.6328	1.25876	2.60161	2.486782	1.3648125
310	-1034.511097	-1073.8159	-1073.814189	-1113.120291	-1113.1216	1.4439	1.83355	3.31948	4.040473	2.4378375
320	-1034.510161	-1073.813837	-1073.812149	-1113.116828	-1113.1179	2.0312	3.12809	4.59957	6.213505	4.7332325
330	-1034.508679	-1073.810588	-1073.809208	-1113.112045	-1113.1124	2.9612	5.16683	6.44505	9.214837	8.18511
340	-1034.506521	-1073.806258	-1073.805635	-1113.106199	-1113.1045	4.3153	7.88391	8.68711	12.8832	13.1379675
350	-1034.504417	-1073.801375	-1073.801857	-1113.099906	-1113.0951	5.6356	10.948	11.0578	16.83206	19.06847
360	-1034.502741	-1073.796743	-1073.798363	-1113.093433	-1113.0877	6.6873	13.8546	13.2503	20.89387	23.70695
370	-1034.506583	-1073.79289	-1073.79659	-1113.088749	-1113.0805	4.2764	16.2723	14.3628	23.83308	28.2556975
380	-1034.509318	-1073.80932	-1073.808511	-1113.083392	-1113.0752	2.5602	5.9625	6.88242	27.19459	31.5325025
390	-1034.511449	-1073.813553	-1073.812004	-1113.077958	-1113.0686	1.223	3.3063	4.69056	30.60443	35.67212
400	-1034.512787	-1073.816576	-1073.814712	-1113.119521	-1113.1192	0.3834	1.40937	2.99129	4.523647	3.9137175
410	-1034.513355	-1073.81818	-1073.816407	-1113.122548	-1113.123	0.027	0.40285	1.92768	2.624205	1.544905
420	-1034.512716	-1073.818585	-1073.816992	-1113.124068	-1113.1245	0.428	0.14872	1.56059	1.670405	0.6381675
430	-1034.511544	-1073.818278	-1073.816785	-1113.124216	-1113.1255	1.1634	0.34136	1.69049	1.577535	0
440	-1034.510477	-1073.817643	-1073.816262	-1113.124258	-1113.1251	1.8329	0.73982	2.01867	1.55118	0.24347
450	-1034.509995	-1073.817177	-1073.816251	-1113.123835	-1113.1242	2.1354	1.03224	2.02557	1.816612	0.786885
460	-1034.510315	-1073.817043	-1073.815913	-1113.123282	-1113.123	1.9346	1.11632	2.23767	2.16362	1.527335
470	-1034.511371	-1073.817368	-1073.817733	-1113.124444	-1113.124	1.2719	0.91238	1.09561	1.434465	0.9531725
480	-1034.51229	-1073.817469	-1073.817613	-1113.123526	-1113.1232	0.6953	0.84901	1.17092	2.01051	1.439485
490	-1034.512646	-1073.817051	-1073.816413	-1113.121125	-1113.1207	0.4719	1.1113	1.92391	3.517138	3.0214125
500	-1034.51208	-1073.815271	-1073.813883	-1113.117292	-1113.1158	0.827	2.22825	3.51149	5.922345	6.065415
510	-1034.510345	-1073.811873	-1073.810583	-1113.111617	-1113.1087	1.9158	4.3605	5.58224	9.483407	10.513135
520	-1034.507267	-1073.80675	-1073.805664	-1113.104108	-1113.0992	3.8472	7.57518	8.66891	14.1953	16.507015
530	-1034.503032	-1073.799923	-1073.79947	-1113.096745	-1113.0875	6.5047	11.8591	12.5556	18.81559	23.8293125
540	-1034.502039	-1073.792228	-1073.792727	-1113.087945	-1113.0735	7.1278	16.6877	16.7869	24.33759	32.6481975
minima	-1034.513398	-1073.818822	-1073.819479	-1113.12673	-1113.1255	0	0	0	0	0

Table S1. Absolute energy values (in au) obtained from AC analog optimization (top 6 entries) and dihedral angle-based relaxed coordinate scans (110-540º) at M06-2X/6-311G**. DE values were computed from these numbers by subtracting the lowest value of the individual series and converting to kcal/mol by multiplying by 627.5.

Free Energy Barrier Determination. Free energy barriers were determined as follows. Single point energy – including vibrational frequency measurements – were obtained for each molecules' global minima and 2 local maxima. In each case, none of the minima (*ie*, GS) had a vibrational frequency, while all the local maxima (*ie*, TS) had a single negative frequency. A folder of compiled text files of cartesian coordinates (.xyz) from these computations are provided in a separate zip file.

ΔG for each transition state was determined by subtracting the maxima from the minima, and converted to kcal/mol by multiplying by 627.5.

These are included in the following table:

	Free Energy (au)			Imaginary Frequency (cm-1)			ΔG(kcal/mol)		
	GS	TS1	TS2	GS	TS1	TS2	GS	TS1	TS2
MTC (**3**)	-1034.2362	-1034.22433	-1034.224952	-	-77.916263	-54.5426	-	7.44654	7.0562
MB-MTC (**5**)	-1073.51614	-1073.48642	-1073.487672	-	-29.069197	-40.2997	-	18.6537	17.866
MT-MTC (**4**)	-1073.51682	-1073.48222	-1073.491252	-	-37.350552	-31.4093	-	21.7102	16.045
DM-MTC (**6**)	-1112.79758	-1112.74931	-1112.74722	-	-31.470897	-14.2954	-	30.2863	31.598
**iso-(6)**	-1112.79694	-1112.73896	-1112.734017	-	-11.19128	-10.5131	-	36.3787	39.482

Table S2. Free energy and associated data obtained from a set of follow-up single-point energy experiments with calculated vibrational frequencies (M06-2X/6-311G**).

Rate constants were obtained for each transition state (k_1 and k_2) by using the Arrhenius equation,

$k = (k_bT/h)e^{-\Delta G/kbT}$

$T = 298.15$ K, $k_B = 1.38 \times 10^{-23}$ m^2kg/s^2K, $h = 6.6 \times 10^{-34}$ m^2kg/s^2, $R = 1.99 \times 10^{-3}$ kcal/molK.

For each molecule, the pair of transition states were added to one-another to obtain k_{obs}, which could then be converted to half-life of enantiomerization through the equation $t_{1/2} = \ln(2)/k_{obs}$.

Meanwhile, $\Delta G^{‡}_{obs}$ was obtained by submitting k_{obs} to the following equation.

$\Delta G^{‡}_{obs} = -RT*\ln(k_BT/hk_{obs})$

Data from these studies are included in the following table (**Table S3**):

	Rate constants			Free Energy	Half-life to enantiomerization		
	k(1) (s-1)	k(2) (s-1)	k(obs) (s-1)	ΔG‡ obs (kcal/mol)	t(1/2) (s)	t(1/2) (d)	t(1/2) (y)
MTC (**3**)	2.4E+07	42891829.2	66670261.32	6.78830047	1.03966E-08	1.20332E-13	3.29676E-16
MB-MTC (**5**)	0.15068	0.52447123	0.675152734	17.71015838	1.026652409	1.18826E-05	3.25549E-08
MT-MTC (**4**)	0.00081	11.2698127	11.27062354	16.03995731	0.06150034	7.11809E-07	1.95016E-09
DM-MTC (**6**)	4.11E-10	4.596E-11	4.57062E-10	30.23712124	1516529006	17552.41905	48.08881932
**iso-(6)**	1.46E-14	7.84E-17	1.46784E-14	36.4	4.72223E+13	546553903.2	1497407.954

Table S3. Rate constants, observed free energy, and half-life to enantiomerization as determined from the above equations.

Ground State Profiling. Evaluating the ground-state dihedral angle for profiling, it was noted that the minimal energy profiles were non-symmetric. While this is not unreasonable at the transition states where ortho groups may not have enough energy to pass one-another until past the coplanar orientation of the dihedral, near the ground states the enantiomers should be consistent. It was found that the reason behind this was that the clustered nature of the trimethoxy groups limited their rotation during optimizations. The impact of this was

that the 'enantiomers' were conformationally different and effectively atropdiastereotopic (see **Figure S1** for example). The nature of this intriguing C-O rigidity – at least computationally – is currently unclear and under investigation. However, for the present studies we chose to make the simplifying assumption that the lowest energy enantiomer for each was the most accurate for a given dihedral. Numbers from this data are observed in **Table S4** below, and were used to generate **Scheme 1C** in the manuscript.

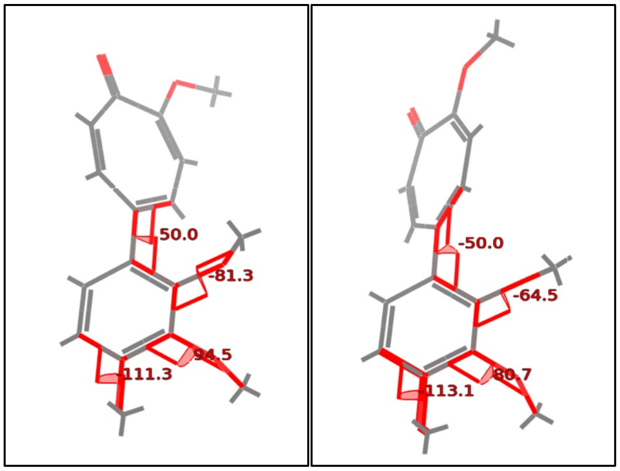

Figure S1. Example of mirror AC ring plane from torsional scan (50º vs. -50º), rendered energetically different based on lack of rotation of trimethoxy groups (-81.3º, 94.5º and -111.3º vs. -64.5º, 80.7º and -113.1º). Structure A and B are MTC at 310º and 410º in the previously described **Table S1**, respectively. As B (-50 or 410º) is lower in energy than A (50 or 310º), its energy value was chosen to represent 50º in **Table S4**, below.

180°-based dihedral	ΔE (kcal/mol)				
	3 (MTC)	5	MT-MTC (4)	6	*iso*-6
0	6.6872675	13.8545725	13.25029	20.893867	23.70695
10	4.2764125	10.9479925	11.057805	16.83206	19.06847
20	2.5602	5.962505	6.88242	12.883202	13.1379675
30	1.2229975	3.3062975	4.6905625	9.2148375	8.18511
40	0.3834025	1.409365	2.9912925	4.5236475	3.9137175
50	0.0269825	0.402855	1.92768	2.624205	1.544905
60	0.427955	0.1487175	1.5605925	1.670405	0.6381675
70	1.163385	0.34136	1.690485	1.577535	0
80	1.8329275	0.7398225	1.484665	0.8534	0.24347
90	2.1353825	0.9431325	0.9105025	0.31124	0.70782
100	1.8643025	0.5754175	0.36646	0.1524825	0.0784375
110	1.0372575	0.16817	0	0	0.116715
120	0.3332025	0	0.1989175	0.495725	0.5867125
130	0	0.294925	1.9057175	1.728135	1.90258
140	0.267315	1.376735	3.22284	3.8434375	4.45776
150	1.2368025	3.4456025	5.5816125	7.0951425	8.540275
160	3.0465125	6.59879	8.6689125	11.662088	13.72719
170	5.1436175	10.791745	12.5531375	18.809312	23.8293125
180	7.1277725	16.650085	16.785625	24.337587	32.6481975

Table S4. Compilation of lowest energy values based on 180º rotation, used to profile energetics versus dihedrals of molecules near the ground-state in the manuscript.

Molecular Dynamics Simulations. The molecular dynamics relative binding free energy calculations[37] were conducted using the AToM-OpenMM package version 3.5.0[45] and the ATM MetaForce OpenMM plugin

version 0.3.5[46] and the OpenMM MD engine version 8.0.[47] We used the 5ITZ PDB structure of the tubulin dimer bound to colchicine.[38] The AMBER's FF14SB force field[48] was used for the protein receptor, and the TIP3P model was used for the water solvent. The GAFF force field was used for parameterizing ligands. Starting with the protein receptor and each ligand pair aligned in the protein binding site, the second ligand in the pair was translated by the displacement vector. The system was then solvated within a rectangle box with a 10 Å TIP3P water buffer by tleap from AmberTools. Potassium and chloride ions were added to neutralize the system if needed. Relative binding free energy calculations employed 22 alchemical replicas each simulated for 40 ns. Additional molecular dynamics runs of the complexes to collect the dihedral angle probability distributions were run for 100 ns.[45]

IV. Experimental Profiling of Dihedral Angles

Rotational Energy Barrier Determination. A small spatula tip (~0.2 mg) of either **(*aR*)-6** (*aka*, (aR)-MT-MTC) or **(*aS*)-6 (***aka,* (*aS*)-MT-MTC**)** was diluted in 2 mL o-xylenes in a large vial containing a stir bar. The solution was heated to 135 ºC open to air (temperature set to 135 ºC, but fluxuated between 134 and 138 °C). ~100 µL aliquots were extracted from solution using a syringe fitted with a long disposable needle at various time intervals and diluted into automated sample vials containing 500 µL CH_2Cl_2 to rapidly cool. These samples were monitored on Agilent with an analytical HPLC IA column, using 0 – 40% *i*PrOH in Hexanes gradient over 30 minutes at a flow rate of 0.5 mL/min, and a pressure of roughly 5 bar). Samples eluted approximately 20 minutes into run, with **(*aR*)-6** eluting first, followed by **(*aS*)-6**. Three separate runs were carried out, two of which monitored racemization of **(*aS*)-6**, and one of which monitored racemization of **(*aR*)-6**.

Integration of the HPLC traces were used to determine enantiomeric excess by calculating the difference between the two enantiomers and dividing that by the sum of the two enantiomers, and then multiplying that value by 100. From this data was plotted ln/(ee) vs. time (seconds) using Microsoft Excel, and a line-fit provided a slope (corresponding to $k_{racemization}$), and dividing this number by half providing $k_{enantiomerization}$ (or k_{ent} in equation below), with units inverse seconds. A summary of data follows (**Table S5**), and representative chromatograms illustrated in **section VI**.

Experiment 1				
time (s)	area (aR-6)	area (aS-6)	ee	ln(1/ee)
0	0	1.285	100	0
300	301	9154	93.6329984	0.06578732
600	1020	11740	84.0125392	0.17420412
1200	1495	7022	64.8937419	0.43241899
3600	2425	4284	27.7090475	1.2834112
5400	4735	5514	7.60074154	2.57692437
7200	4287	4302	0.17464198	6.35018739

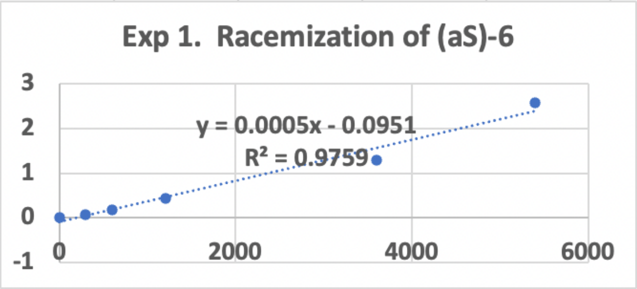

Experiment 2				
time (s)	area (aR-6)	area (aS-6)	ee	ln(1/ee)
0	100	0	100	0
180	100	0	100	0
600	1845	91.5	90.5499613	0.09926843
1860	1816	436	61.2788632	0.48973521
3540	651	294	37.7777778	0.97344915
5400	799	447	28.2504013	1.26406252
7200	747	478	21.9591837	1.51598474

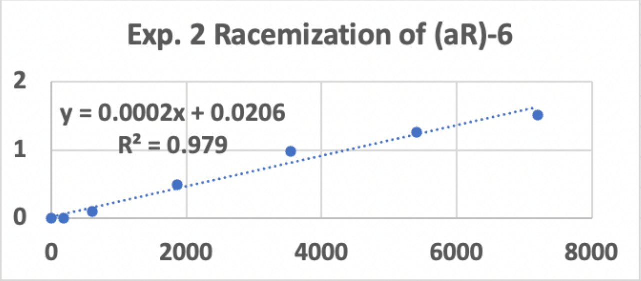

Experiment 3				
time (s)	area (aR-6)	area (aS-6)	ee	ln(1/ee)
0	163	12216	97.3665078	0.0266879
120	78	6601	97.664321	0.02363388
900	907	8843	81.3948718	0.20585792
1800	2363	11026	64.7023676	0.43537239
3600	2258	5060	38.28915	0.96000362
6120	2806	4106	18.8078704	1.67089477

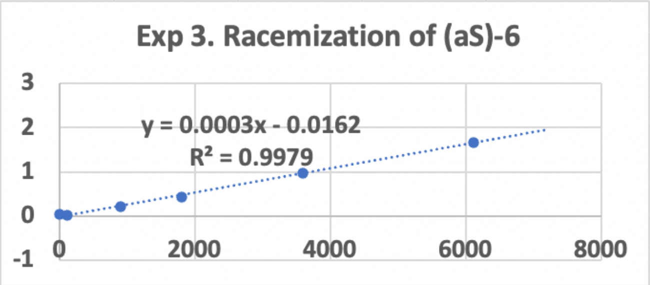

Table S5. Data obtained from racemization studies (left), along with plots of ln(1/ee) (y axis) vs. time in seconds (x axis) (right). X and Y labels on these graphs are omitted for clarity and text size considerations.

Free energy to enantiomerization was calculated with the following equation:

$$\Delta G^{\ddagger} = -(R*T)*(\ln((k_{ent}*h)/(k_B*T)))$$

Wherein $R = 8.31$ J*mol^{-1}*K^{-1}; $k_B = 1.38 \times 10^{-23}$ J*K^{-1}; $h = 6.63 \times 10^{-34}$ J*s, T = 408 K. This value was converted from J to kcal/mol through the conversion factor of 4184.

$\Delta G^{\ddagger}_{trial\ 1(B)}$ = 30.9 kcal/mol

$\Delta G^{\ddagger}_{trial\ 2(A)}$ = 31.6 kcal/mol

$\Delta G^{\ddagger}_{trial\ 3(B)}$ = 31.3 kcal/mol

$\Delta G^{\ddagger}_{average}$= 31.3 +/- 0.4 kcal/mol

Vibrational Circular Dichroism

Computational (*via* Gaussian '09) and Experimental (*via* ChiralIR w/ Dual PEM) VCD and IR spectra were obtained on $CDCl_3$ solutions of each enantiomer of DT-MTC (**6**), iso-DT-MTC (***iso*-6**), colchicine, and isocolchicine. The complete reports – as provided by BioTools - are available online at the publishers website as a part of a supporting information zip file. This zip file also contains a word file containing cartesian coordinates, as well as .mol files of key conformations. Note, in the report, **(aR)-6**, **(aS)-6**, **(aS)-*iso*-6** and **(aR)-*iso*-6** are referred to as bejcecineA, bejcecineB, isobejcecineC and isobejcecineD, respectively, in line with our internal naming system.

V. Bioactivity Methods for Colchicine AC Analogs

1. Binding affinity to the colchicine site of bovine brain tubulin (PurSolutions, LLC (puresoluble.com)) was assayed by competition of test compounds with MDL ((E)-1-(2,5-dimethoxyphenyl)-3-[4-(dimethylamino)phenyl]-2-methylprop-2-en-1-one) as described previously.[43] MDL fluorescence increases many-fold upon binding to the colchicine site of tubulin. Inhibition of this fluorescence increase was measured and converted to a K_d for the test compound as described.

2. Inhibition of tubulin polymerization was determined as described in [PMID 23973075]. In brief, 10 μM bovine brain tubulin (PurSolutions, LLC (puresoluble.com)) was incubated under conditions strongly promoting polymerization of tubulin (1M NaGlutamate, 0.1 M Mes (Morpholinoethanesulfonic acid), 1 mM $MgCl_2$, 0.5 mM GTP, pH 6.9), in the absence or presence of 3.3 or 33 μM test compound, incubated for 30 min at 37 °C, then centrifuged at 100,000 x g for 8', and the top ¾ of the supernatant removed. Protein concentration was measured, and polymerization was determined as pelletable protein lost from the supernatant.

3. Inhibition of cell growth was determined using standard procedures. Cell lines were obtained from the NCI anticancer drug screen, and maintained in DMEM medium supplemented with 10% fetal bovine serum. Growing cells were exposed to serial dilutions of each compound for 3 days. Cell growth was determined with CellTiter Assay Reagent (Promega), and growth parameters, including inhibition of growth, were measured using the methods specified by the manufacturer.

60-Cell Screen

(aR)-6, **(aS)-6**, **(aS)-*iso*-6**, and **(aR)-*iso*-6** were submitted to the 60 Cell Screen panel, and evaluated at 5 doses (100 mM, 10 mM, 1 mM, 0.1 mM, and 0.01 mM). A summary of the data for all molecules is in **Table S6,** and a compilation of GI_{50} values of active compounds determined from these studies along with prior

experiment of colchicine is listed in **Table S7.** The complete report from this submission is available at the publisher's website as a separate supplementary information file.

	NSC#	Mean GI_{50} (μM)	Log_{10} Range at GI_{50}	Mean TGI (μM)	Log_{10} Range at TGI
**(aR)-DM-MTC (6)**	830095	2.4	2.39	63	2.13
**(aS)-DM-MTC (6)**	830096	>100	0.65	>100	0
**(aS)-*iso*-6**	830097	27	1.46	91	0.81
**(aR)-*iso*-6**	830098	>100	0.40	>100	0

Table S6. Potency and cell line selectivity of Atropisomers A-D in NCI-60 assay.

Cell Type	(aR)-DM-MTC		Colchicine		(aS)-isoDM-MTC	
**Leukemia**	**log**	**µM**	**log**	**µM**	**log**	**µM**
CCRF-CEM	-5.71	1.95	-7.82	0.02	-4.53	29.512
HL-60(TB)	-6.16	0.69	-7.89	0.01	-4.68	20.893
K-562	-6.31	0.49	-7.79	0.02	-5.3	5.0119
MOLT-4	-5.58	2.63	-7.45	0.04	-4.44	36.308
RPMI-8226	-5.39	4.07	-8	0.01	-4.38	41.687
SR	-6.44	0.36	-7.49	0.03	-5.24	5.7544
**NSCLC**	**log**	**µM**	**log**	**µM**	**log**	**µM**
A549/ATCC	-5.42	3.8	-6.58	0.26	-4.45	35.481
EKVX	-5.18	6.61	-4	100	-4.18	66.069
HOP-62	-5.42	3.8	—	—	-4.46	34.674
HOP-92	-5.45	3.55	—	—	-4.99	10.233
NCI-H226	-5.41	3.89	-7.56	0.03	-4.06	87.096
NCI-H23	-5.39	4.07	-7.54	0.03	-4.33	46.774
NCI-H322M	-5.18	6.61	-7.21	0.06	-4.22	60.256
NCI-H460	-5.55	2.82	-7.65	0.02	-4.5	31.623
NCI-H522	-5.73	1.86	-7.84	0.01	-4.69	20.417
**Colon Cancer**	**log**	**µM**	**log**	**µM**	**log**	**µM**
COLO-205	-5.76	1.74	-7.83	0.01	-4.66	21.878
HCC-2998	-5.55	2.82	-7.51	0.03	-4.48	33.113
HCT-116	-5.71	1.95	-7.49	0.03	-4.6	25.119
HCT-15	-6.17	0.68	-6.68	0.21	-4.89	12.882
HT29	-5.73	1.86	-7.55	0.03	-4.6	25.119
KM29	-5.79	1.62	-7.35	0.04	—	—
SW-620	-5.91	1.23	-7.66	0.02	-4.51	30.903
**CNS Cancer**	**log**	**µM**	**log**	**µM**	**log**	**µM**
SF-268	-5.26	5.5	-5.33	4.68	—	—
SF-295	-5.76	1.74	—	—	-4.59	25.704
SF-539	-5.65	2.24	-7.54	0.03	-4.53	29.512
SNB-19	-5.53	2.95	-7.43	0.04	-5.27	5.3703
SNB-75	-6.04	0.91	-6.04	0.91	-4.51	30.903
U251	-5.53	2.95	-7.53	0.03	-4.725	18.836

Cell Type	(aR)-DM-MTC		Colchicine		(aS)-isoDM-MTC	
**Melanoma**	**log**	**µM**	**log**	**µM**	**log**	**µM**
LOX IMVI	-5.69	2.04	-7.71	0.019	-5.18	6.6069
M14	-6.07	0.85	-7.26	0.055	-4.76	17.378
MDA-MB-435	-6.57	0.27	-8	0.01	-5.46	3.4674
SK-MEL-2	-6.05	0.89	-8	0.01	-4.69	20.417
SK-MEL-5	-5.5	3.16	-8	0.01	-4.34	45.709
UACC-257	-5.11	7.76	-4	100	-4.15	70.795
UACC-62	-6.03	0.93	-7.67	0.021	-4.78	16.596
**Ovarian Cancer**	**log**	**µM**	**log**	**µM**	**log**	**µM**
IGROV1	-5.37	4.27	-7.28	0.052	-4.32	47.863
OVCAR-4	-4.35	44.7	-4.03	93.33	-4.04	91.201
OVCAR-5	-5.36	4.37	-7.12	0.076	-4.38	41.687
OVCAR-8	-5.47	3.39	—	—	-4.45	35.481
NCI/ADR-RES	-5.76	1.74	—	—	-4.55	28.184
SK-OV-3	-5.59	2.57	—	—	-4.57	26.915
**Renal**	**log**	**µM**	**log**	**µM**	**log**	**µM**
786-0	-5.35	4.47	-7.11	0.078	-4.33	46.774
A498	-6.1	0.79	—	—	-4.85	14.125
ACHN	-5.27	5.37	-6.43	0.372	-4.07	85.114
CAKI-1	-6.03	0.93	—	—	-4.71	19.498
RXF 393	-5.71	1.95	-8	0.01	-4.68	20.893
SN 12C	-5.46	3.47	-7.27	0.054	-4.42	38.019
TK-10	-4.18	66.1	-4	100	-4	100
UO-31	-5.16	6.92	-6.29	0.513	-4.34	45.709
**Prostate**	**log**	**µM**	**log**	**µM**	**log**	**µM**
PC-3	-5.64	2.29	-7.62	0.024	-4.5	31.623
DU-145	-5.45	3.55	-5.08	8.318	—	—
**Breast Cancer**	**log**	**µM**	**log**	**µM**	**log**	**µM**
MCF7	-6.11	0.78	-7.94	0.011	-4.6	25.119
MDA-MB-231/ATCC	-5.59	2.57	-5.15	7.079	-4.63	23.442
HS 578T	-5.81	1.55	—	—	-4.8	15.849
BT-549	-5.69	2.04	-7.37	0.043	-4.82	15.136
T-47D	-5.15	7.08	-4.12	75.68	-4.58	26.303
MDA-MB-468	-5.97	1.07	—	—	-4.85	14.125

Table S7. GI_{50} values from 5-dose experiments obtained through the National Cancer Institute Developmental Therapeutic Program. Values listed as log -4.00 are above the detection limit (ie, < -4.00), values listed as log -8.00 have values below the detection (ie, > - 8.00), and values listed as — are not available. Data from (–)-colchicine obtained from the NCI, experiment ID# 9212EC88, taken on December 14, 1992.

For COMPARE analysis, TGI level of response for **(aR)-DM-MTC (6)** and **(aS)-*iso*-DM-MTC** were compared to those of several established tubulin inhibitors previously tested in the NCI-60 screen. A complete list of this data can be viewed in **Table S8.**

**Compound**	NSC#	Top Concentration	Number of tests	**(aR)-DM-MTC (6)**	**(aS)-*iso*-DM-MTC**	Tubulin Site
**Colchicine**	757	10^{-4} M	7	0.59	0.43	Colchicine
**Centaureidin**	106969	10^{-4} M	4	0.62	0.56	Colchicine
**Combretastatin A-4**	613729	10^{-4} M	7	0.34	0.24	Colchicine
**Curacin D**	669360	10^{-4} M	3	0.31	0.23	Colchicine
**Maytansine**	153858	10^{-4} M	12	0.54	0.4	Vinca
**Rhizoxin**	332598	10^{-4} M	8	0.46	0.27	Vinca
**Dolastatin 10**	376128	10^{-8} M	5	0.64	0.7	Vinca
**Vincristine sulfate**	67574	10^{-5} M	9	0.4	0.33	Vinca
**Epothilone A**	684362	10^{-6} M	2	0.4	0.46	Taxane
**Taxol**	125973	$10^{-4.6}$ M	10	0.55	0.58	Taxane
**(aR)-DM-MTC (6)**	830095	10^{-4} M	1	1	0.74	
**(aS)-*iso*-6**	830097	10^{-4} M	1		1	

Table S8. Pearson Correlation Coefficient at TGI level between **(aR)-DM-MTC (6)** and **(aS)-*iso*-DM-MTC** with known tubulin interacting as determined in the NCI-60 assay. A coefficient of >0.50 is considered significant.

VI. HPLC Chromatograms

DM-MTC, (aR)-6

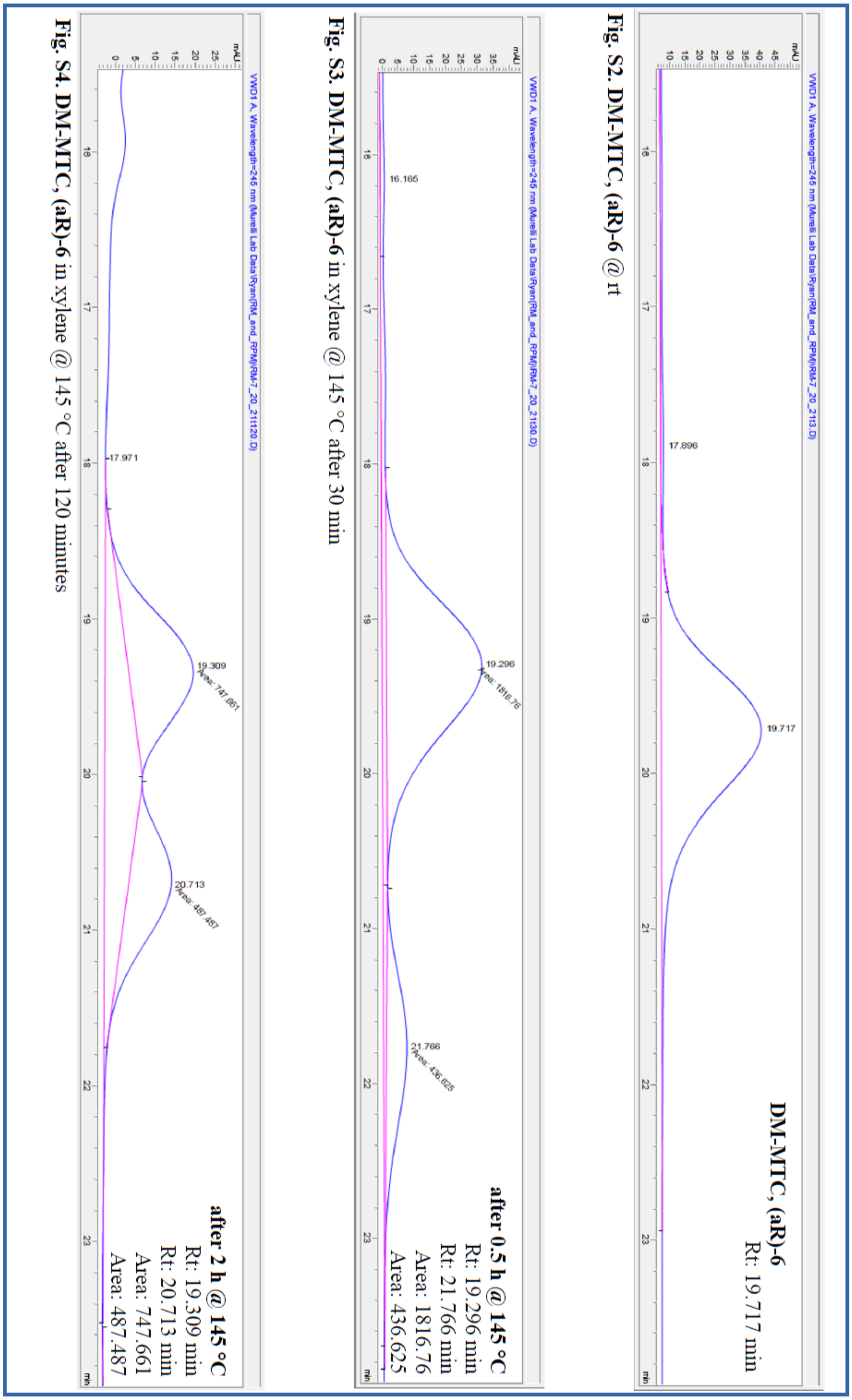

Fig. S2. DM-MTC, (aR)-6 @ rt

Fig. S3. DM-MTC, (aR)-6 in xylene @ 145 °C after 30 min

Fig. S4. DM-MTC, (aR)-6 in xylene @ 145 °C after 120 minutes

DM-MTC, (aS)-6

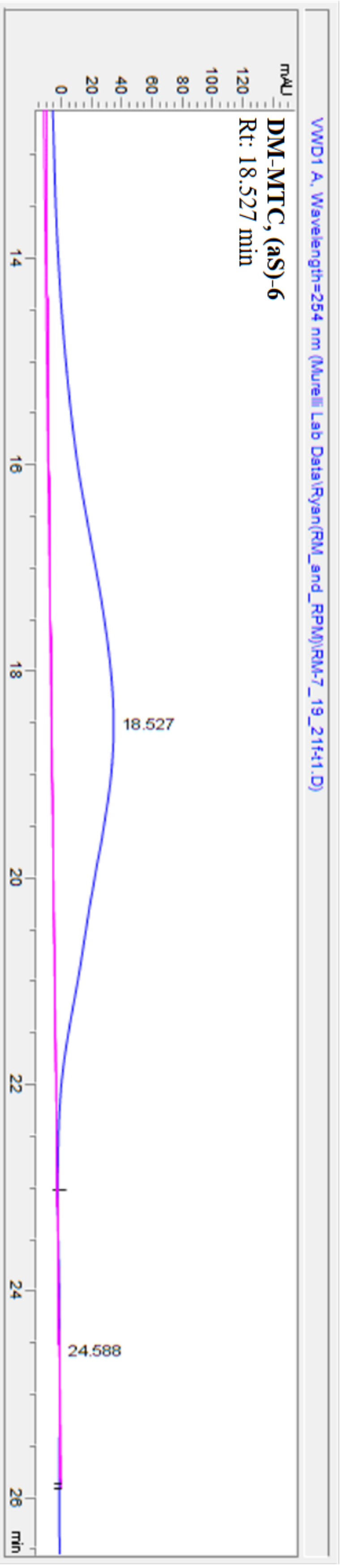

Fig. S5. DM-MTC, (aS)-6 @ rt

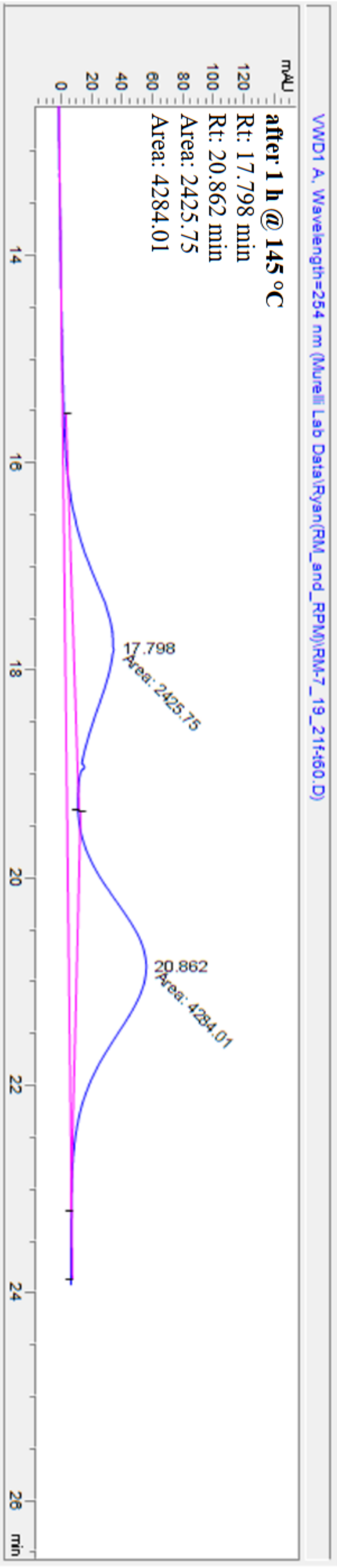

Fig. S6. DM-MTC, (aS)-6 in xylene @ 145 °C after 60 minutes

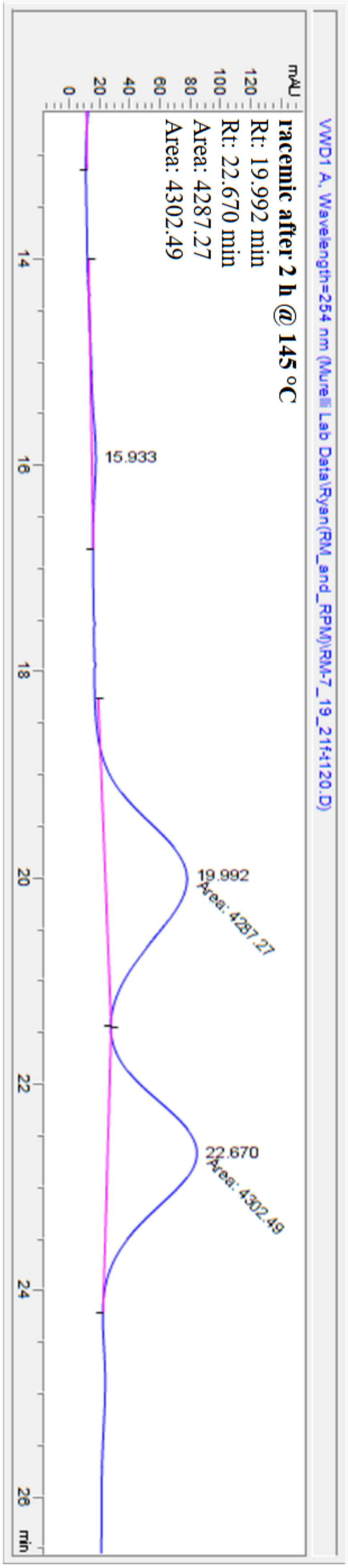

Fig. S7. DM-MTC, 6 racemic @ 145 °C after 120 minutes

Iso-6

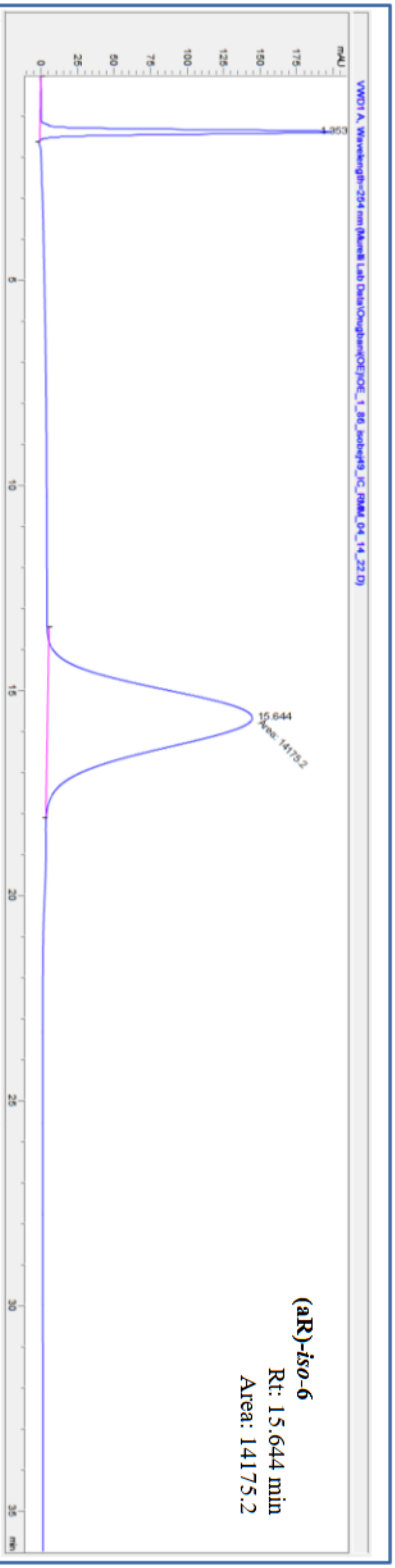

Fig. S8. **(aR)-*iso-6*** @ rt

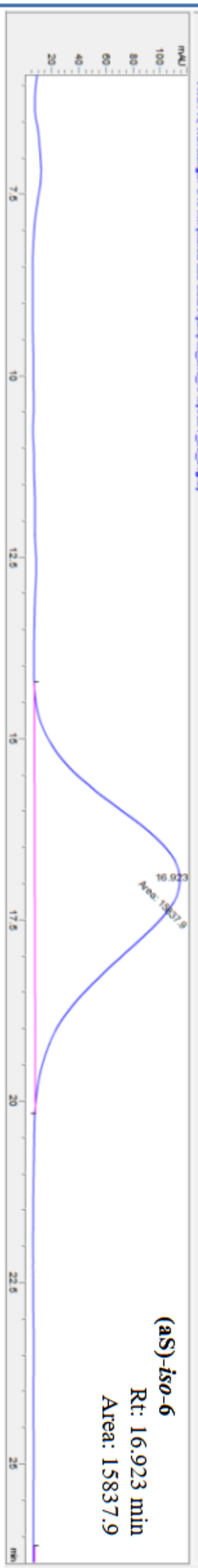

Fig. S9. **(aR)-*iso-6*** @ rt

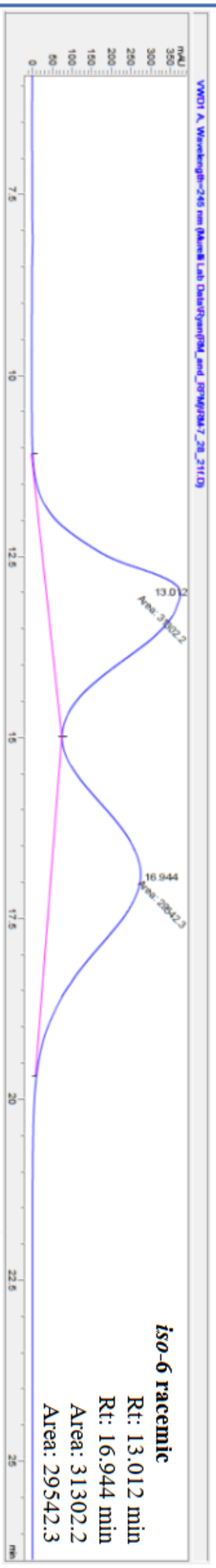

Fig. S10. Racemic **(aR)-*iso-6*** @ rt

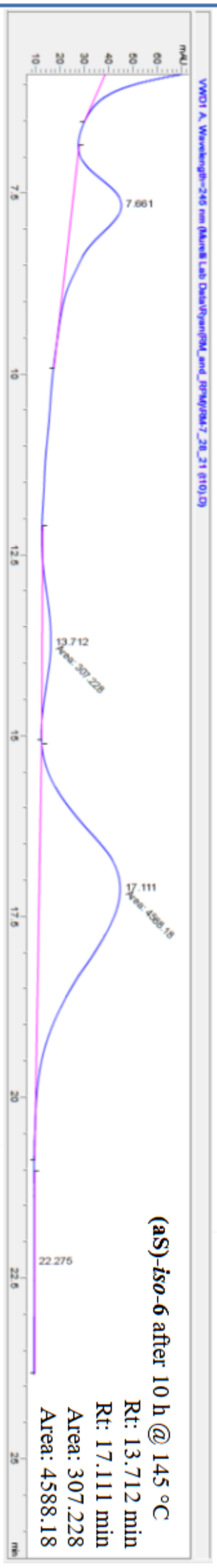

Fig. S11. **(aS)-*iso-6*** racemization experiment after 10 h @ 145 °C

VII. NMR Spectra

^{1}H NMR (400 MHz, $CDCl_3$) of 14

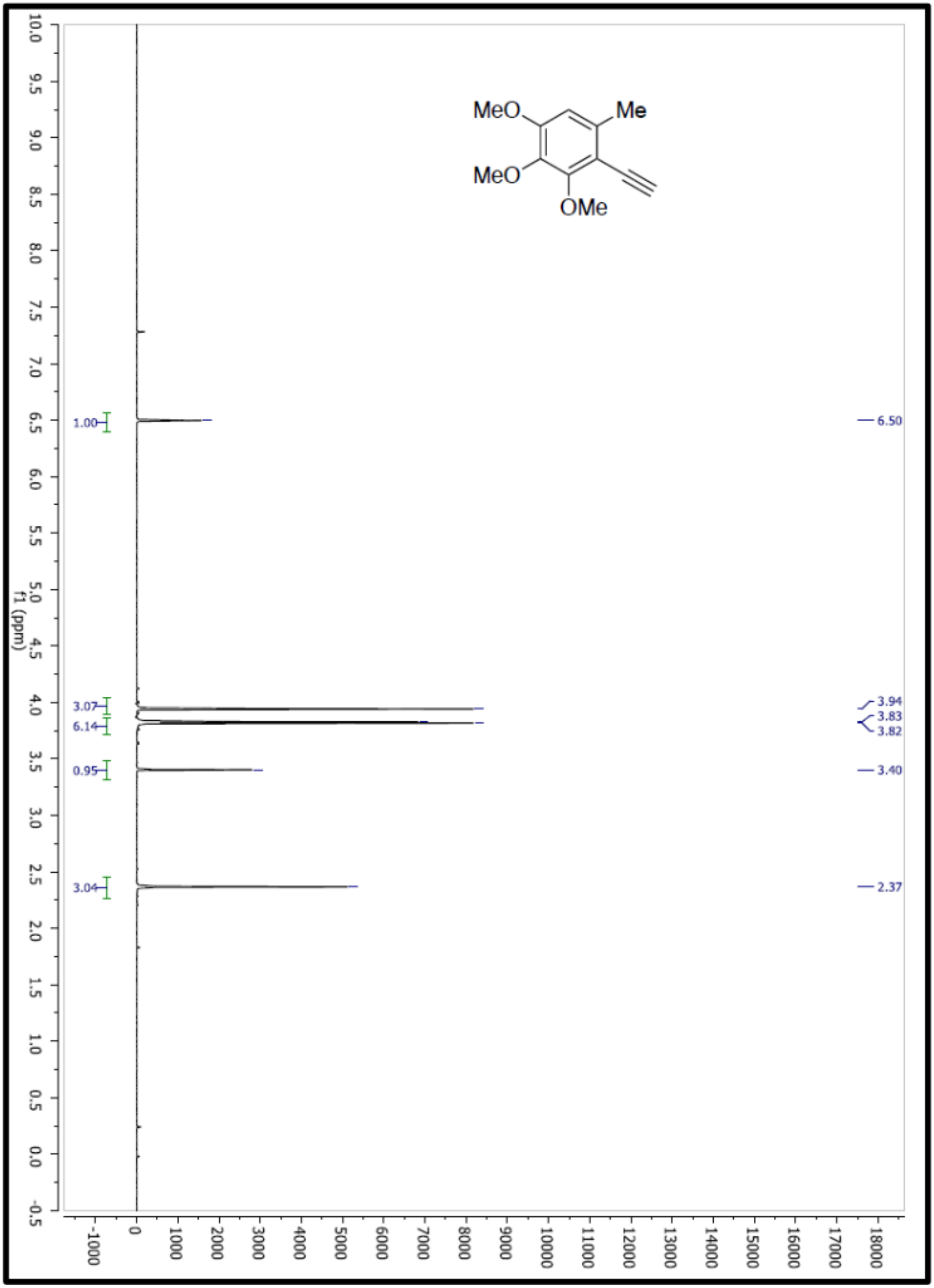

$^{13}C\{^{1}H\}$ NMR (101 MHz, $CDCl_3$) of 14

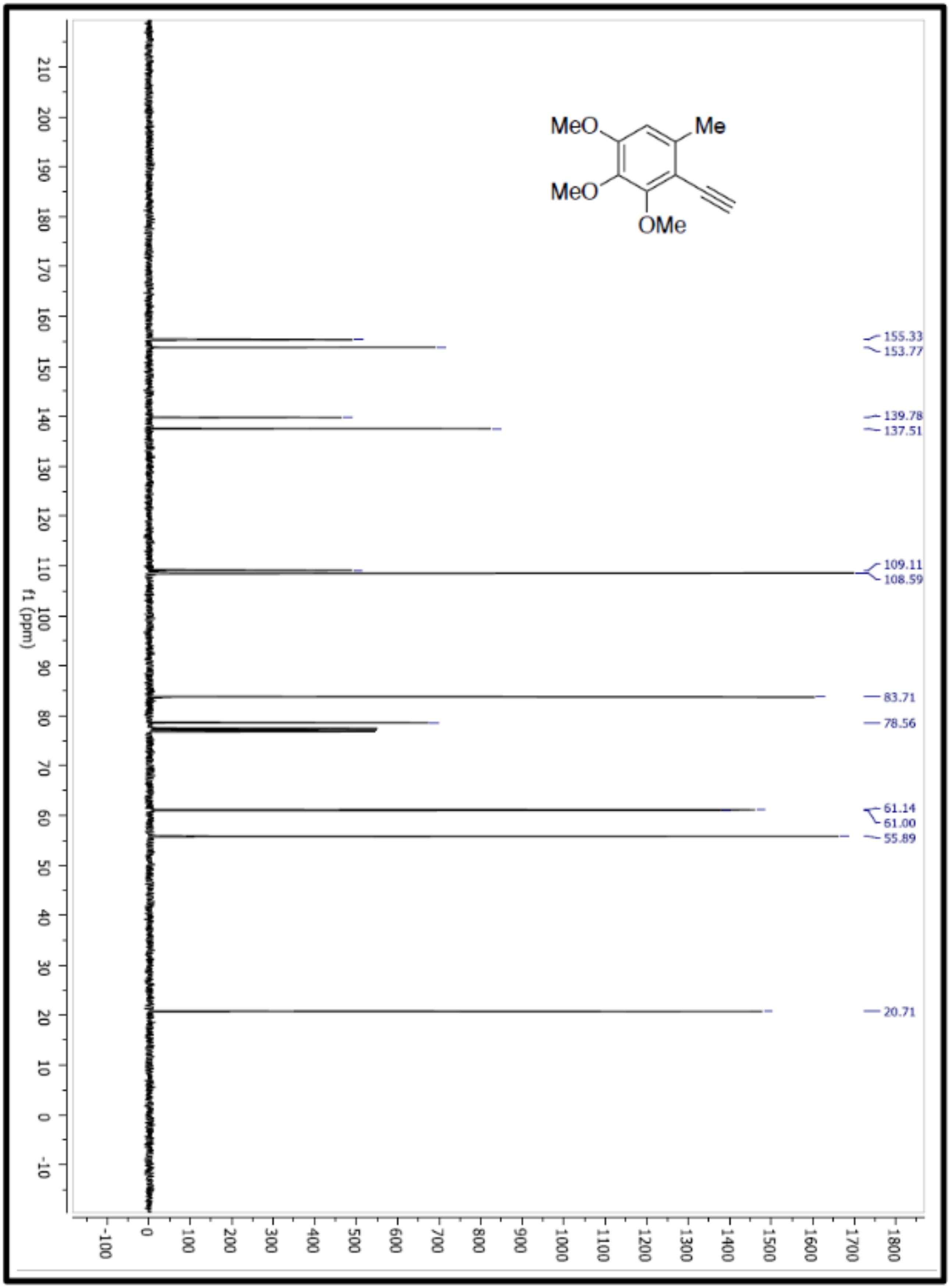

^{1}H NMR (400 MHz, $CDCl_3$) of 15

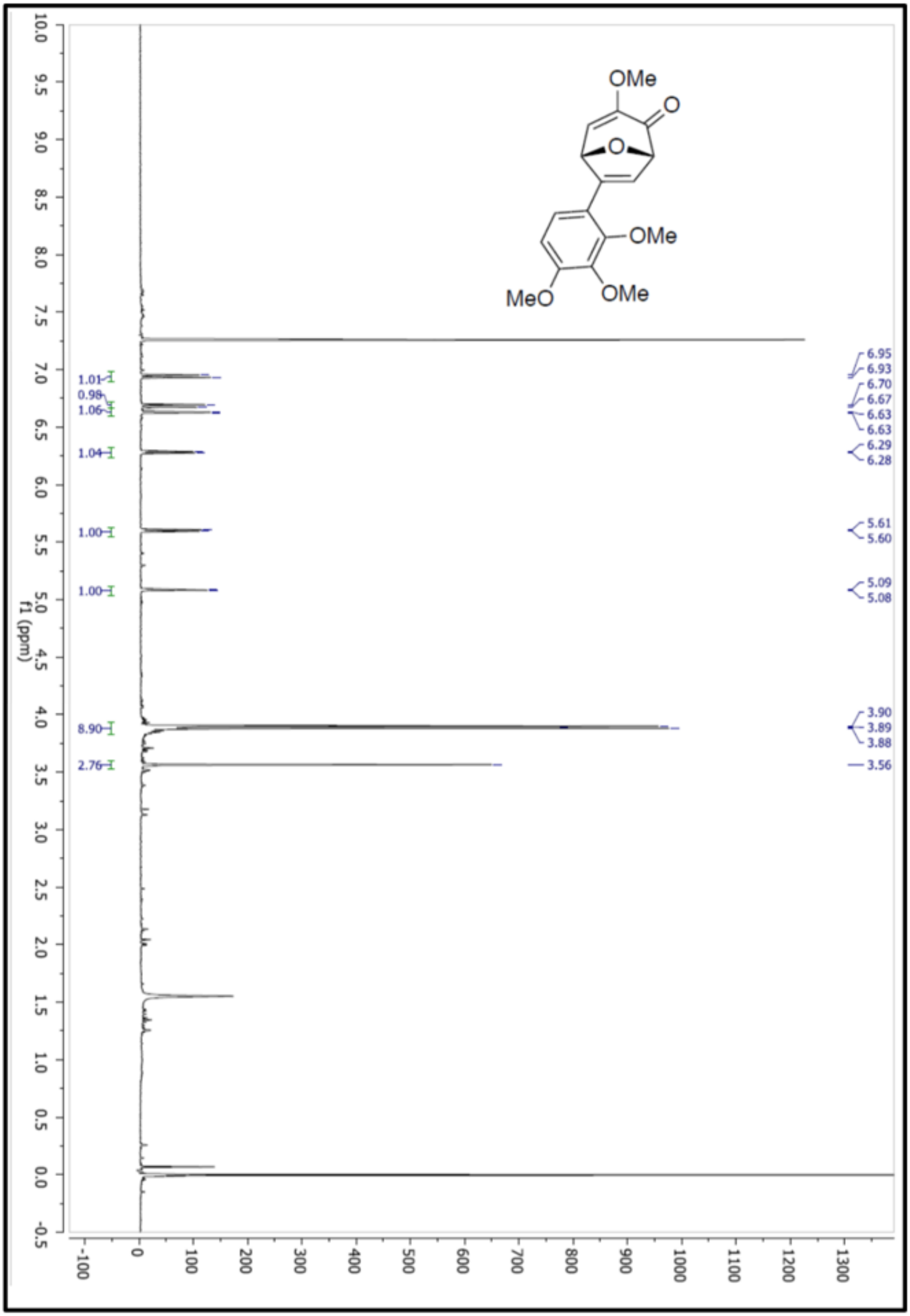

$^{13}C\{^1H\}$ NMR (101 MHz, $CDCl_3$) of 15

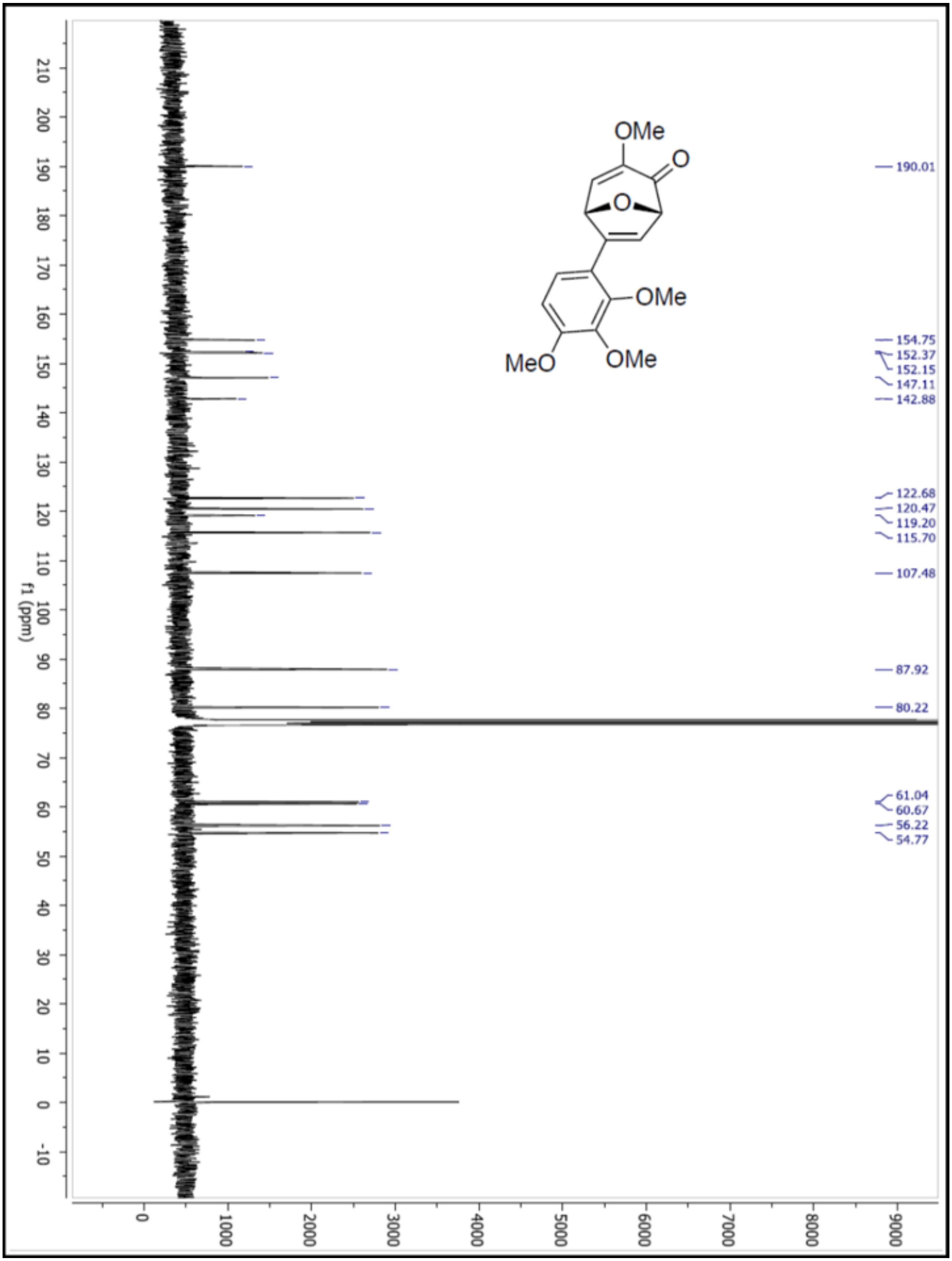

^{1}H NMR (400 MHz, $CDCl_3$) of 16

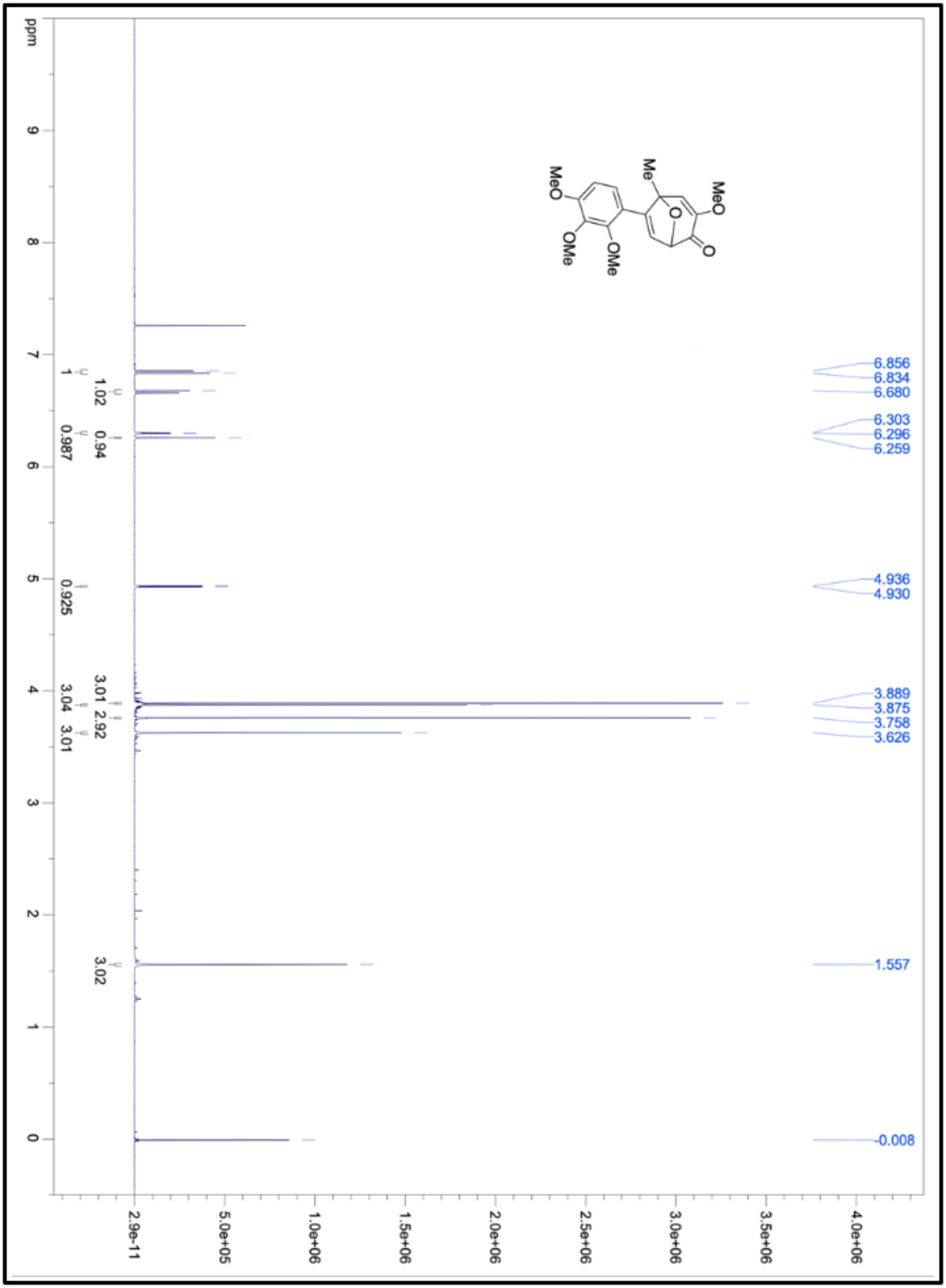

$^{13}C\{^1H\}$ NMR (101 MHz, $CDCl_3$) of 16

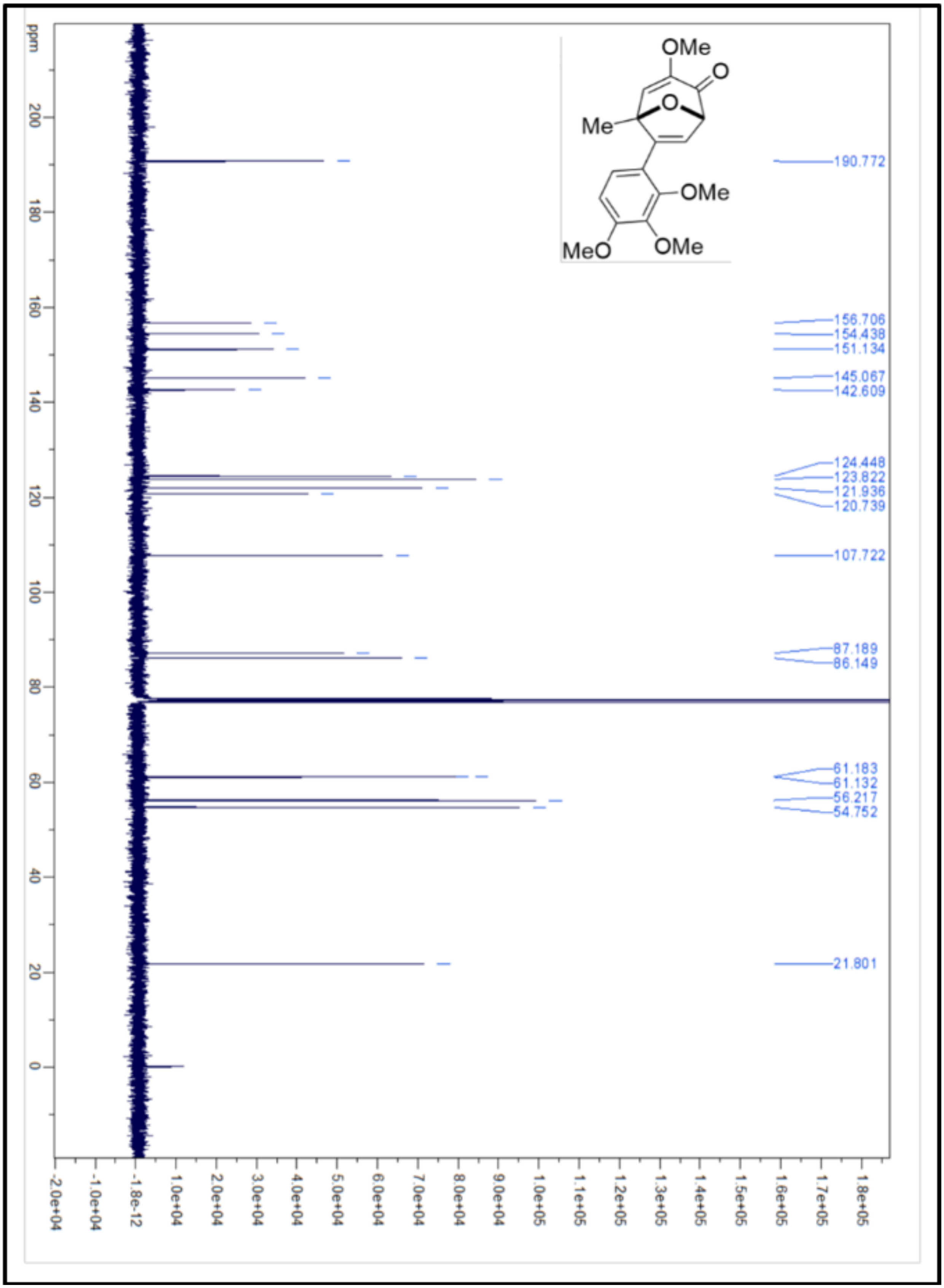

^{1}H NMR (400 MHz, CDCl3) of 17

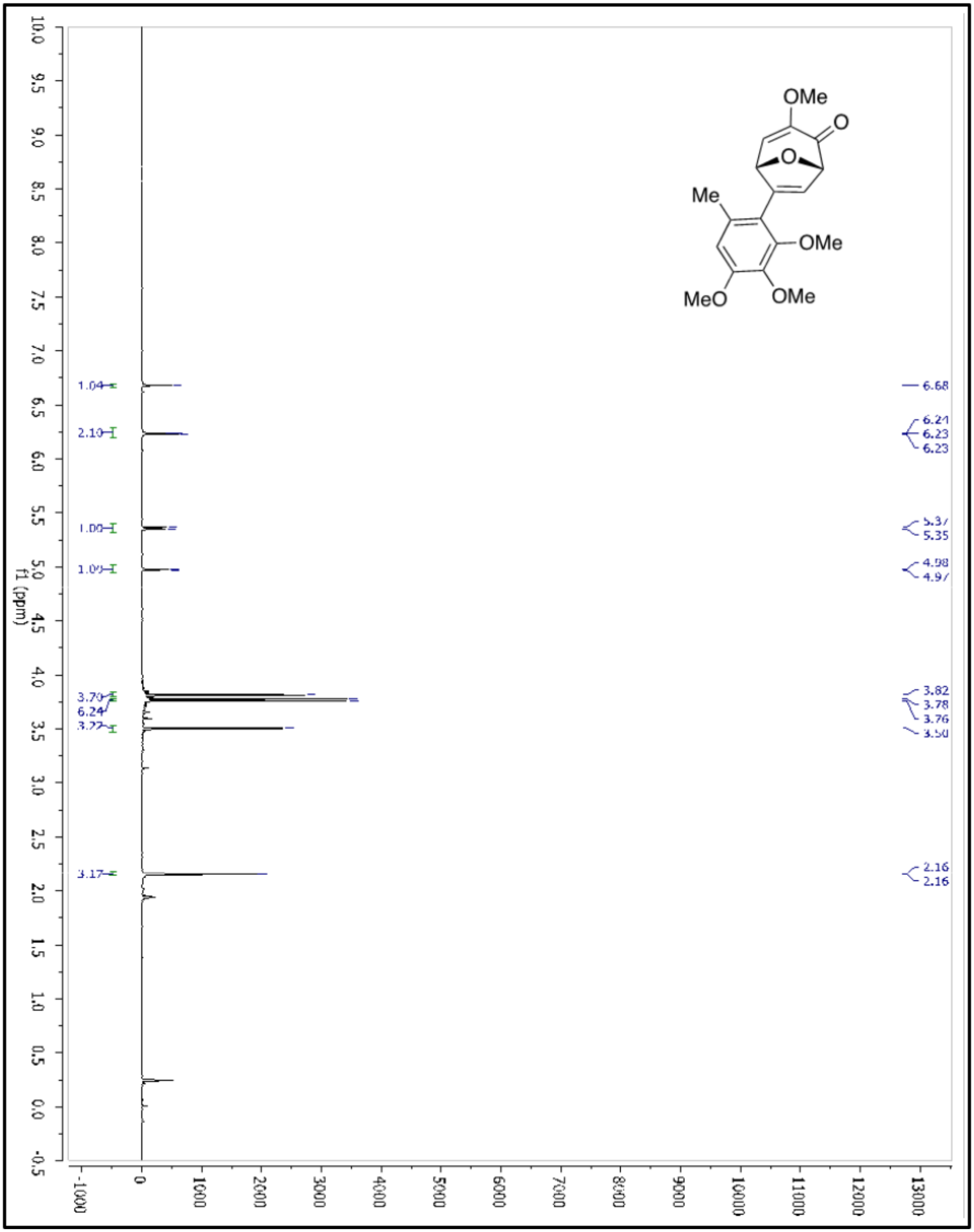

$^{13}C\{^{1}H\}$ NMR (101 MHz, CDCl$_3$) of 17

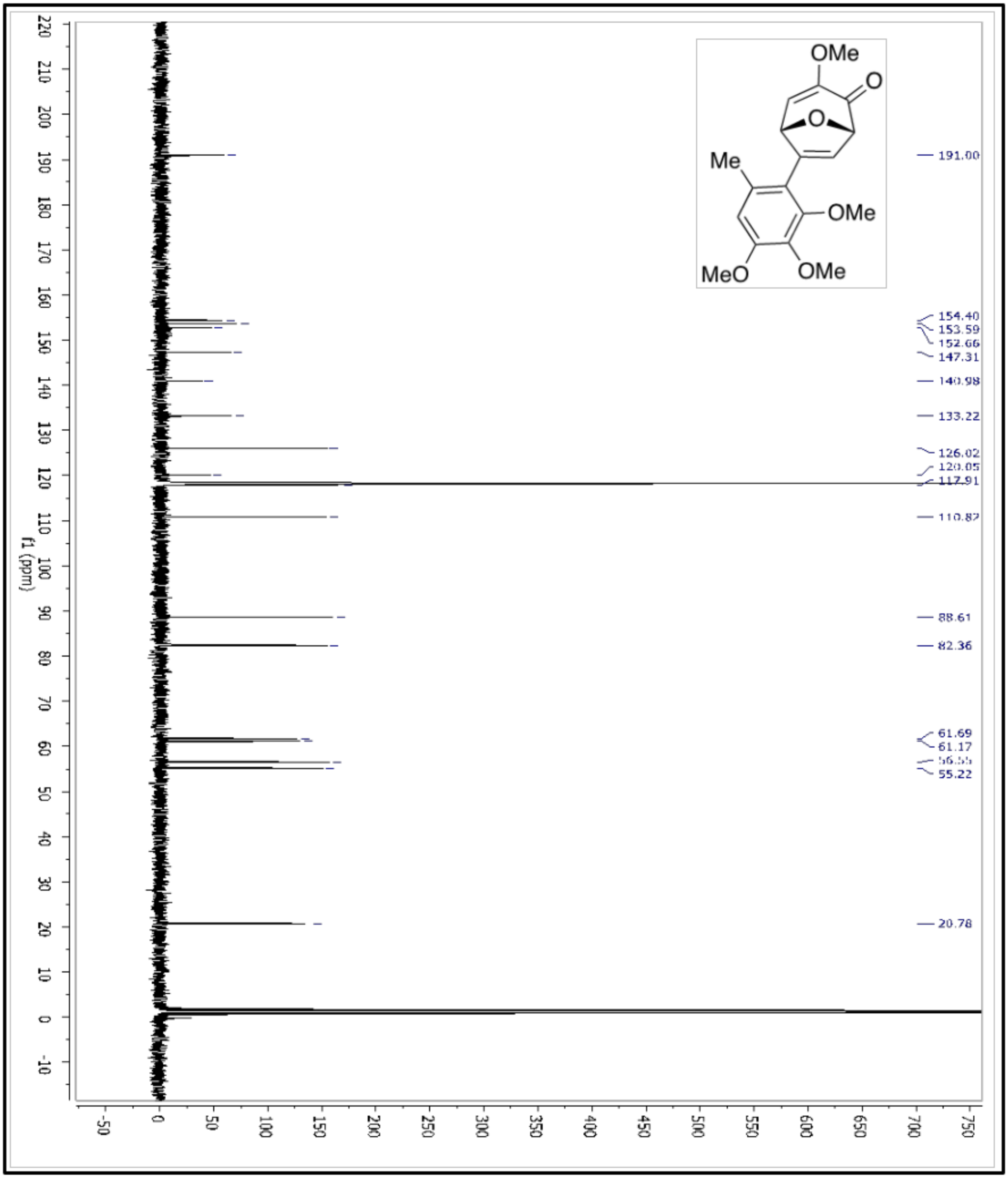

^{1}H NMR (400 MHz, $CDCl_3$) of 18

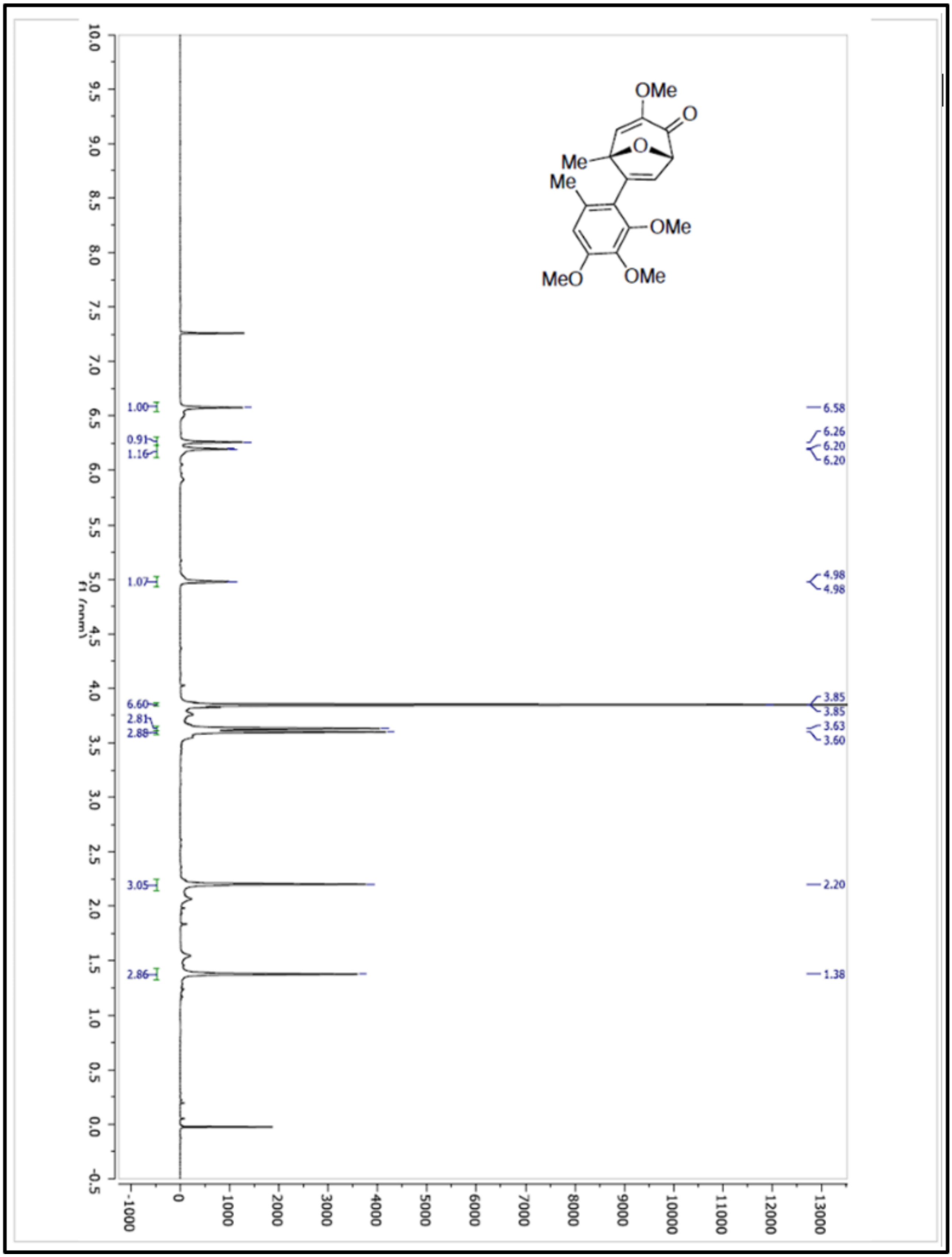

$^{13}C\{^{1}H\}$ NMR (101 MHz, $CDCl_3$) of 18

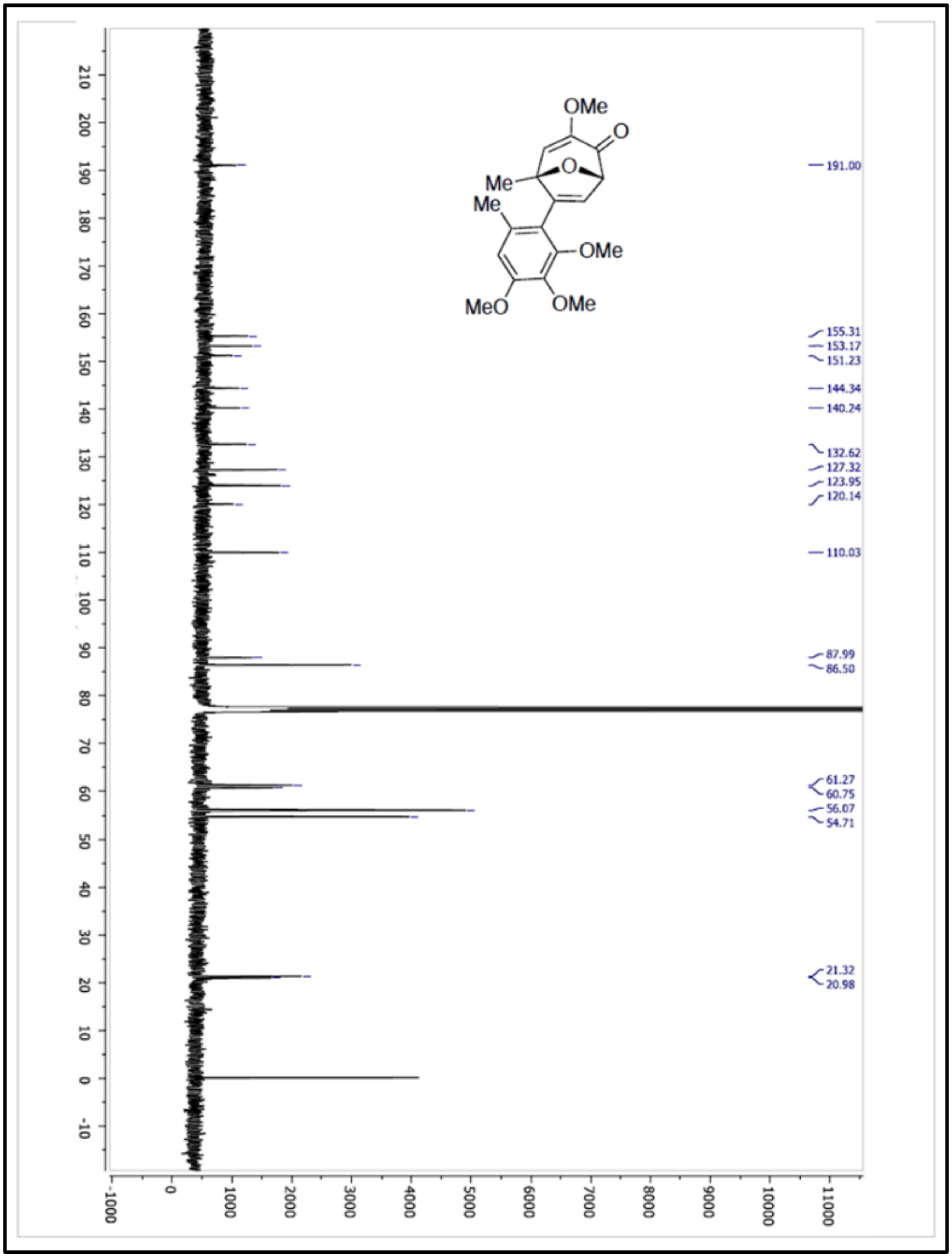

^{1}H NMR (400 MHz, $CDCl_3$) of *iso*-4

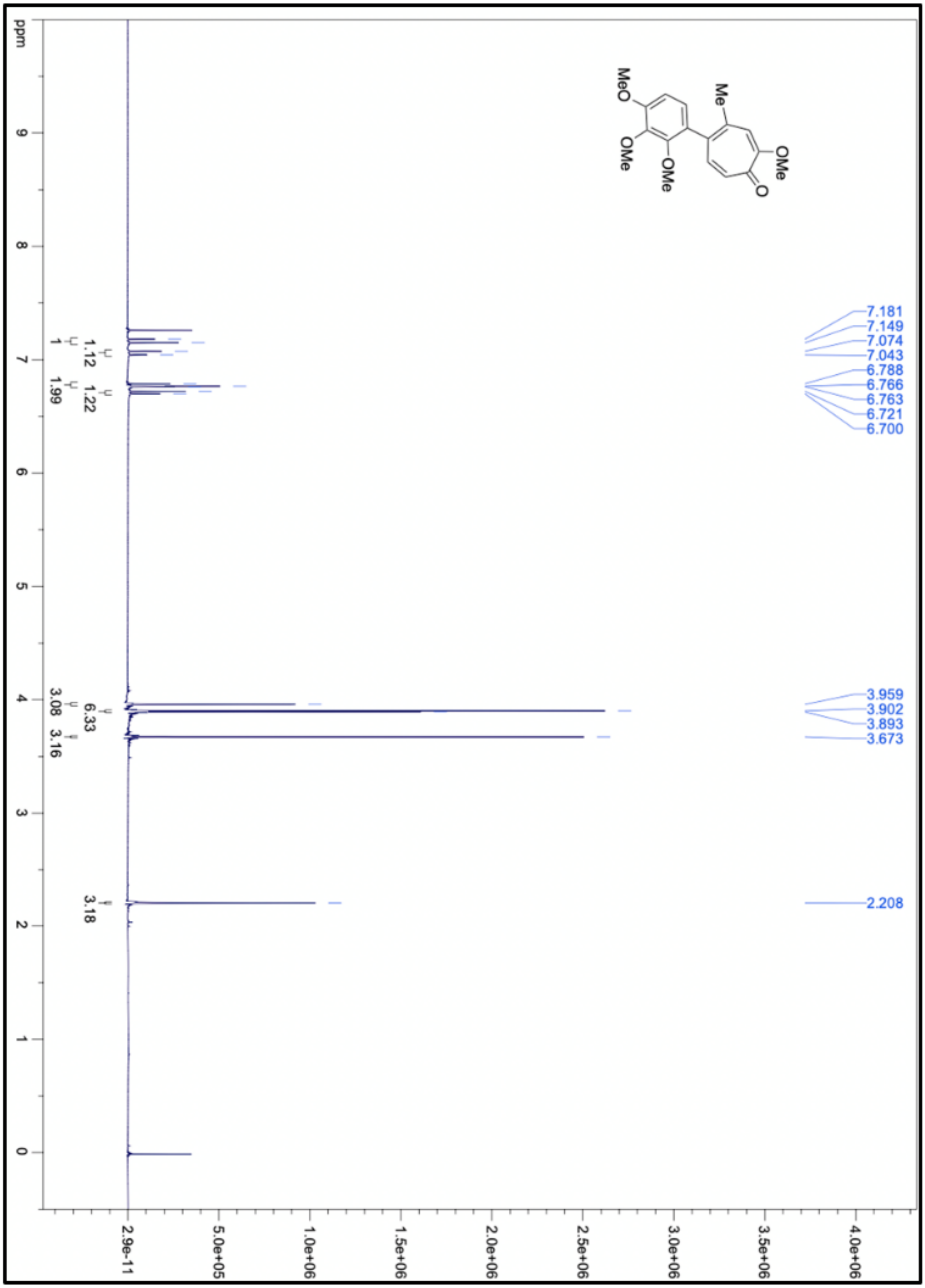

$^{13}C\{^1H\}$ **NMR (101 MHz, CDCl$_3$) of *iso*-4**

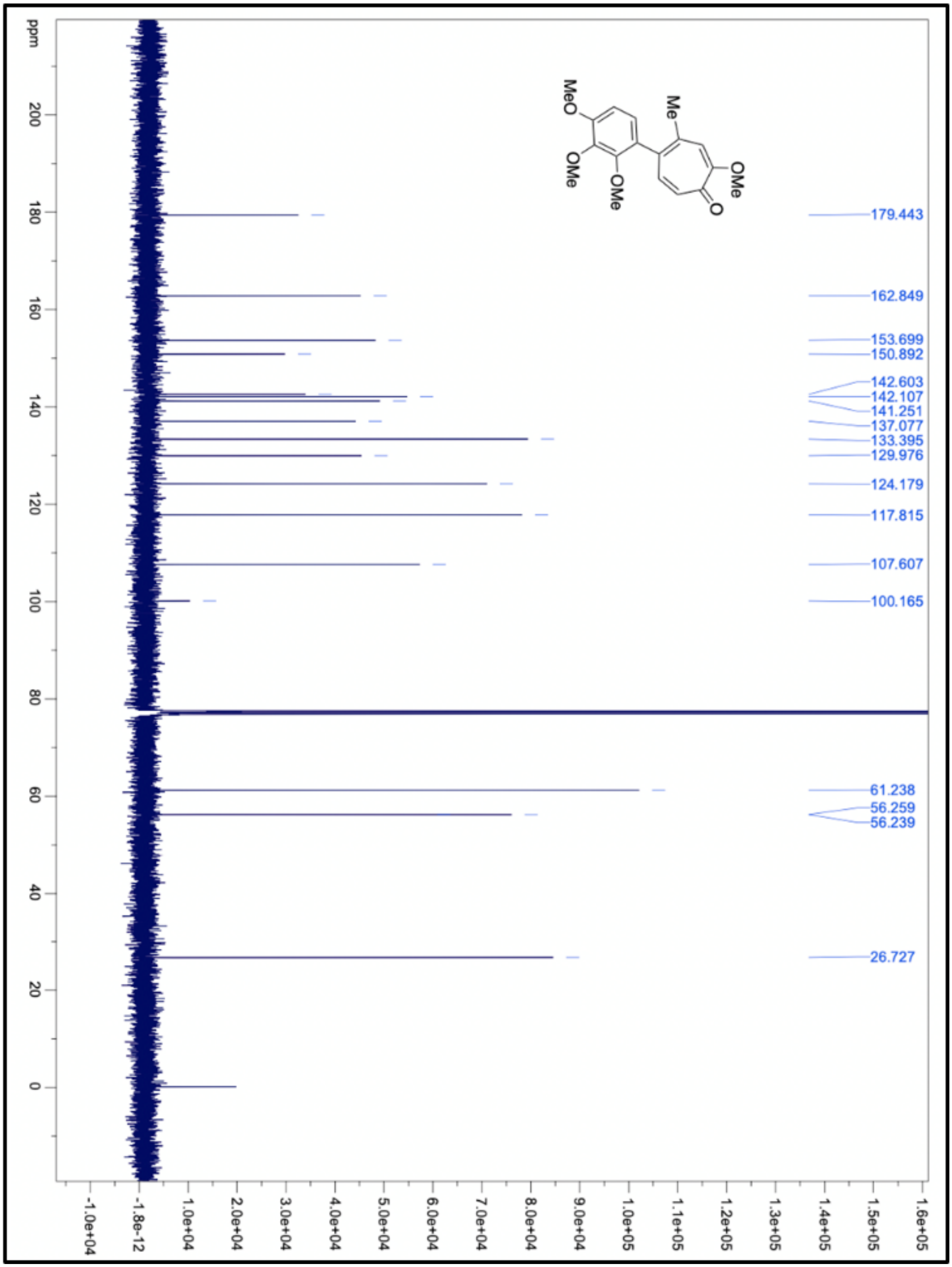

^{1}H NMR (400 MHz, $CDCl_3$) of 5

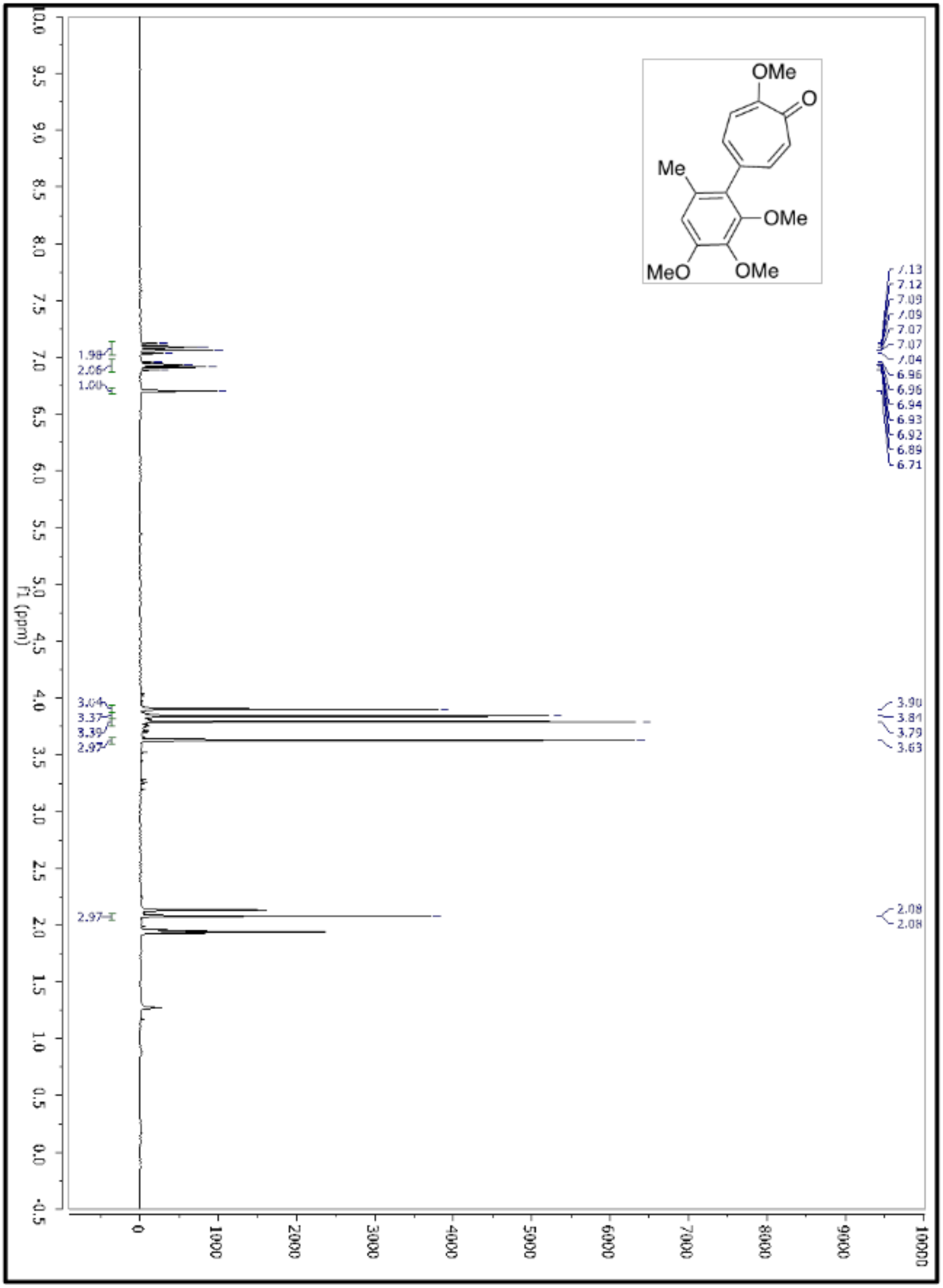

$^{13}C\{^1H\}$ NMR (101 MHz, $CDCl_3$) of 5

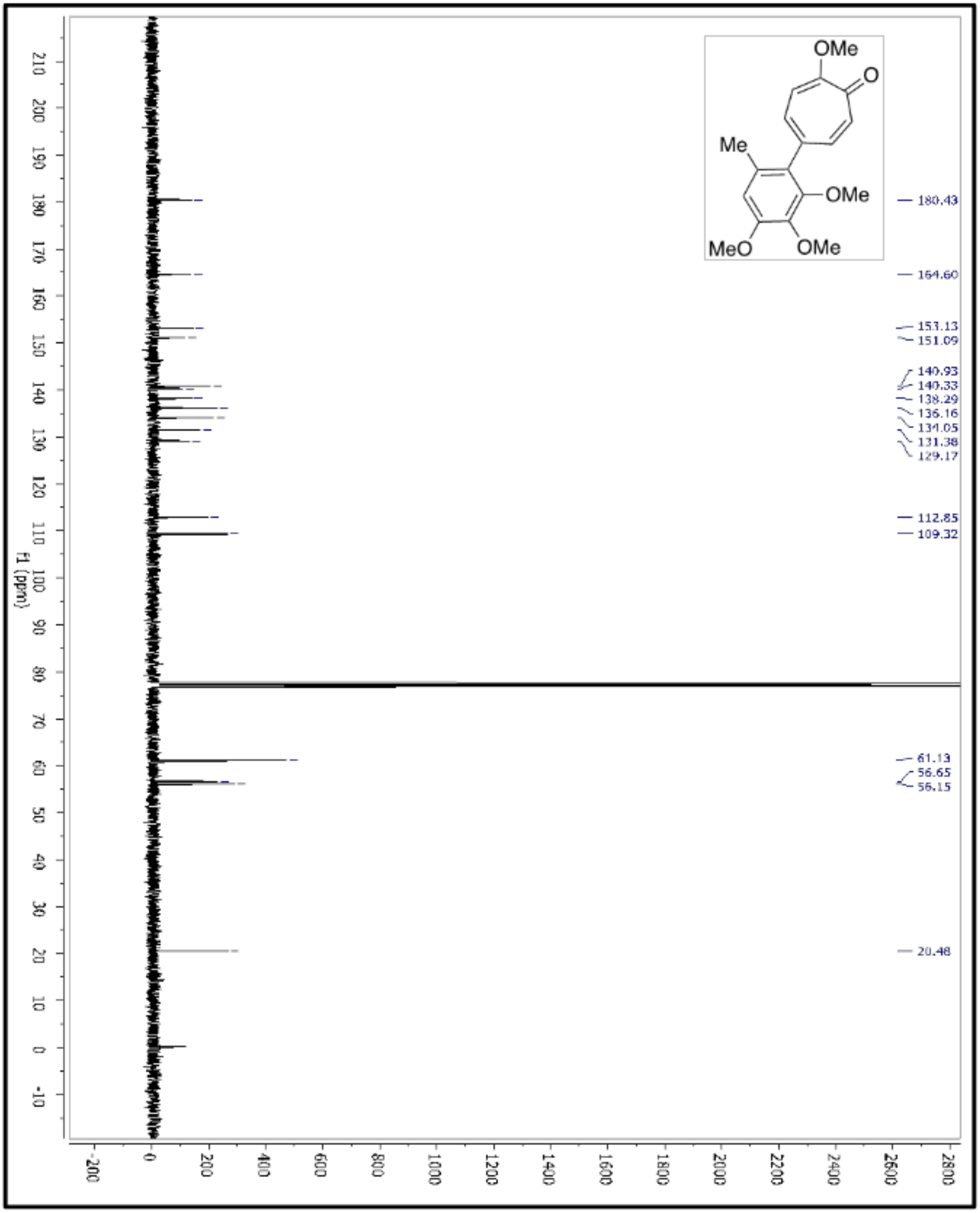

$^{13}C\{^{1}H\}$ NMR (101 MHz, CD_3CN) of 5

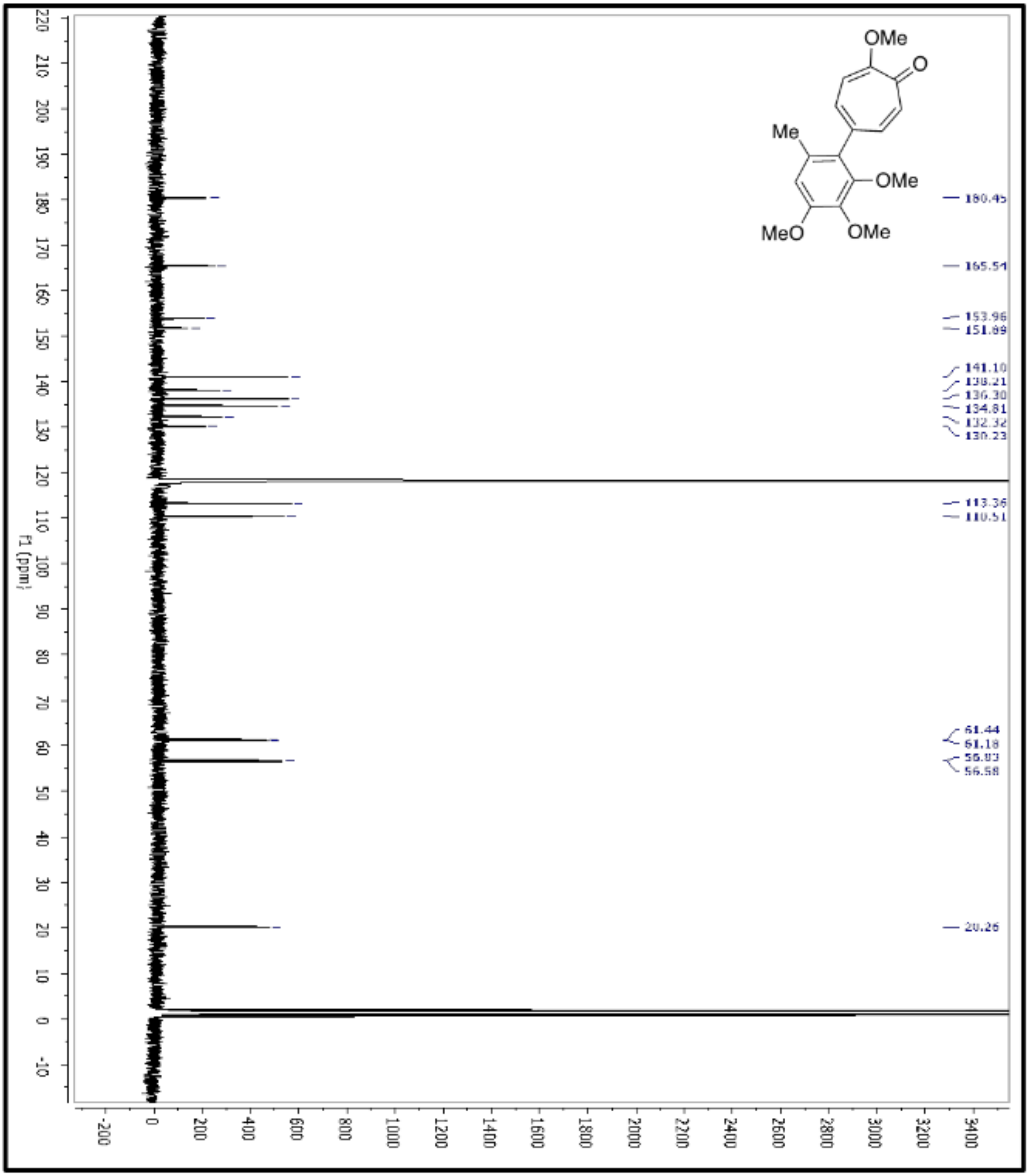

^{1}H NMR (400 MHz, $CDCl_3$) of *iso*-**6**

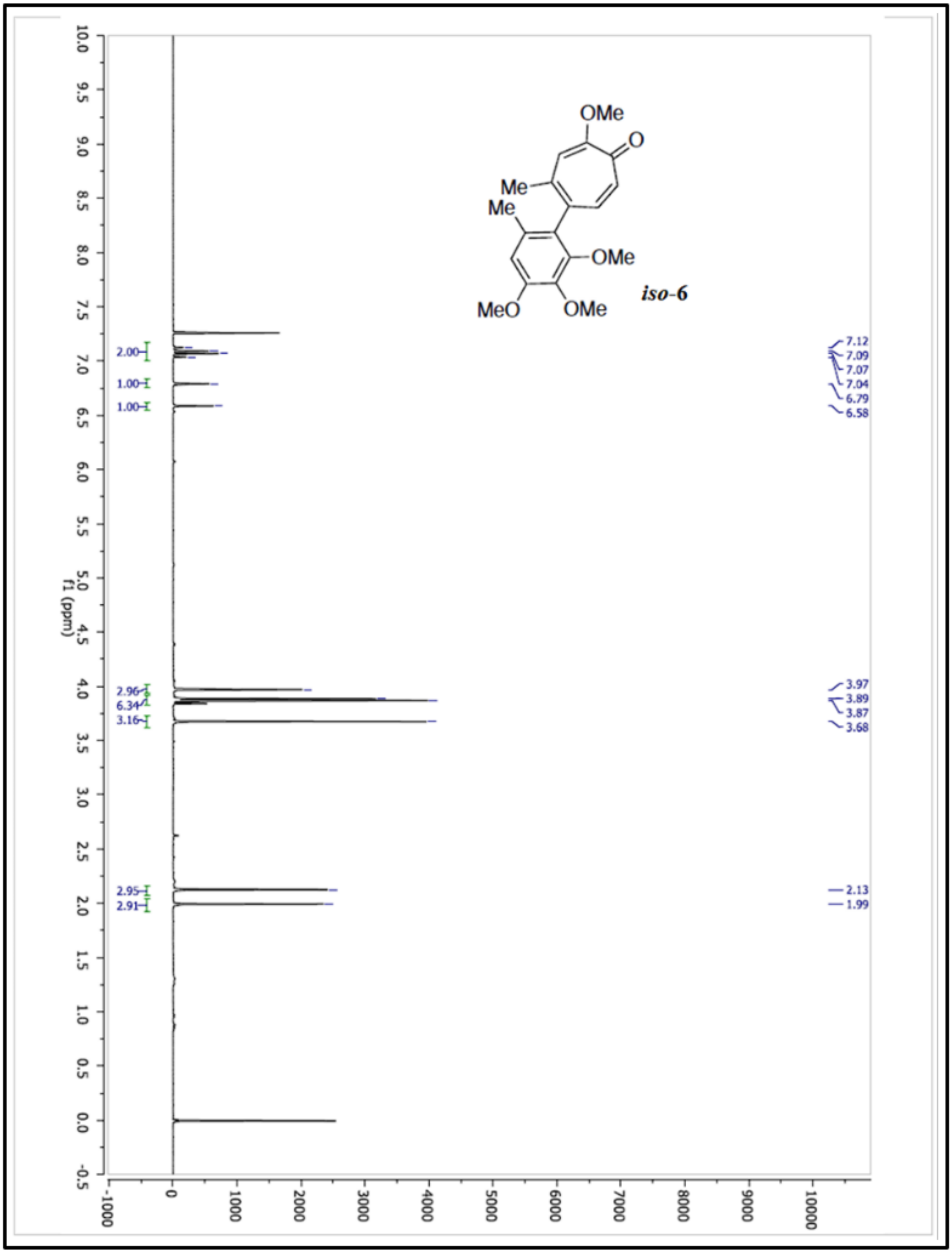

$^{13}C\{^{1}H\}$ NMR (101 MHz, $CDCl_3$) of *iso*-6

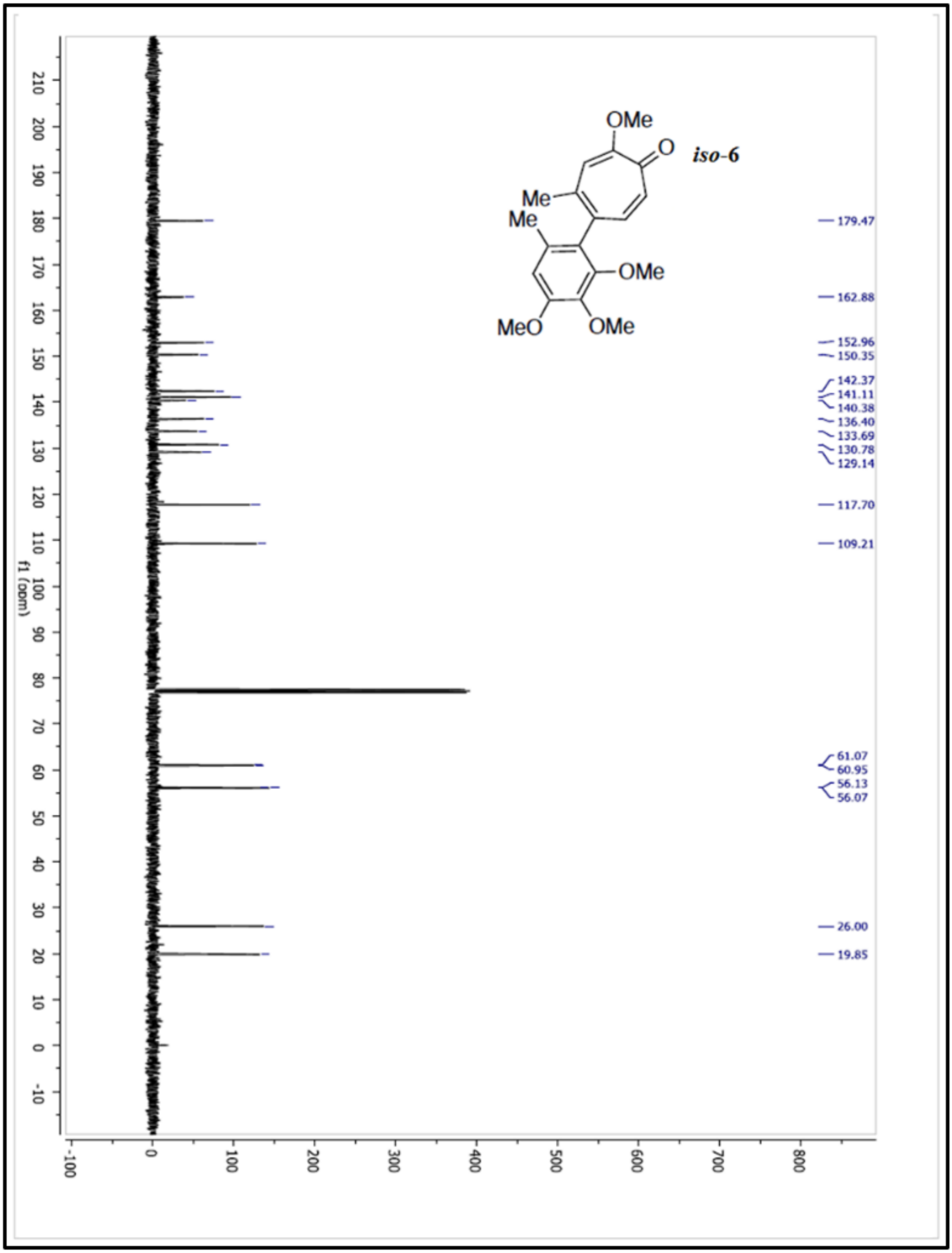

^{1}H NMR (400 MHz, $CDCl_3$) of 19

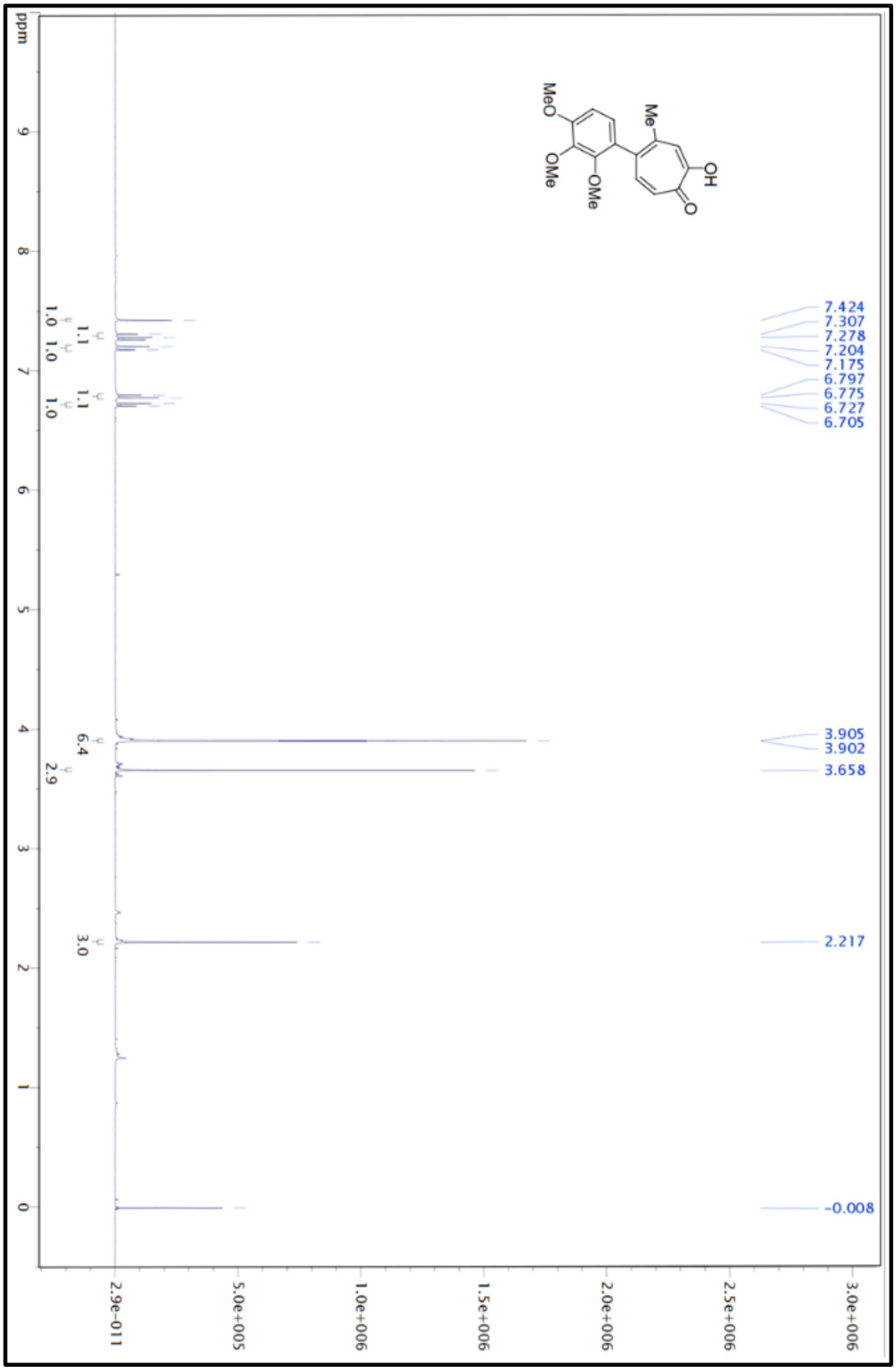

$^{13}C\{^1H\}$ NMR (101 MHz, $CDCl_3$) of 19

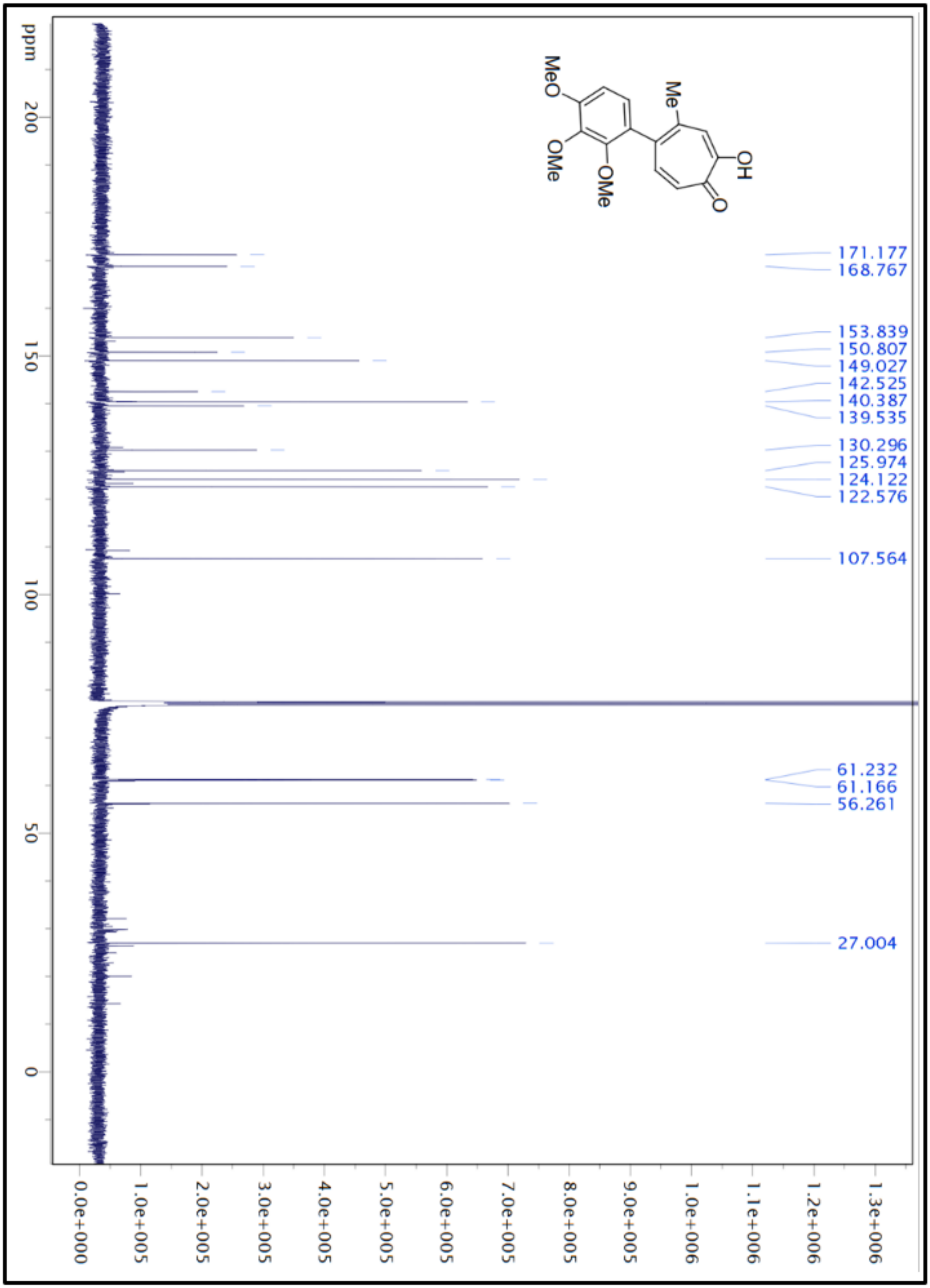

^{1}H NMR (400 MHz, $CDCl_3$) of 20

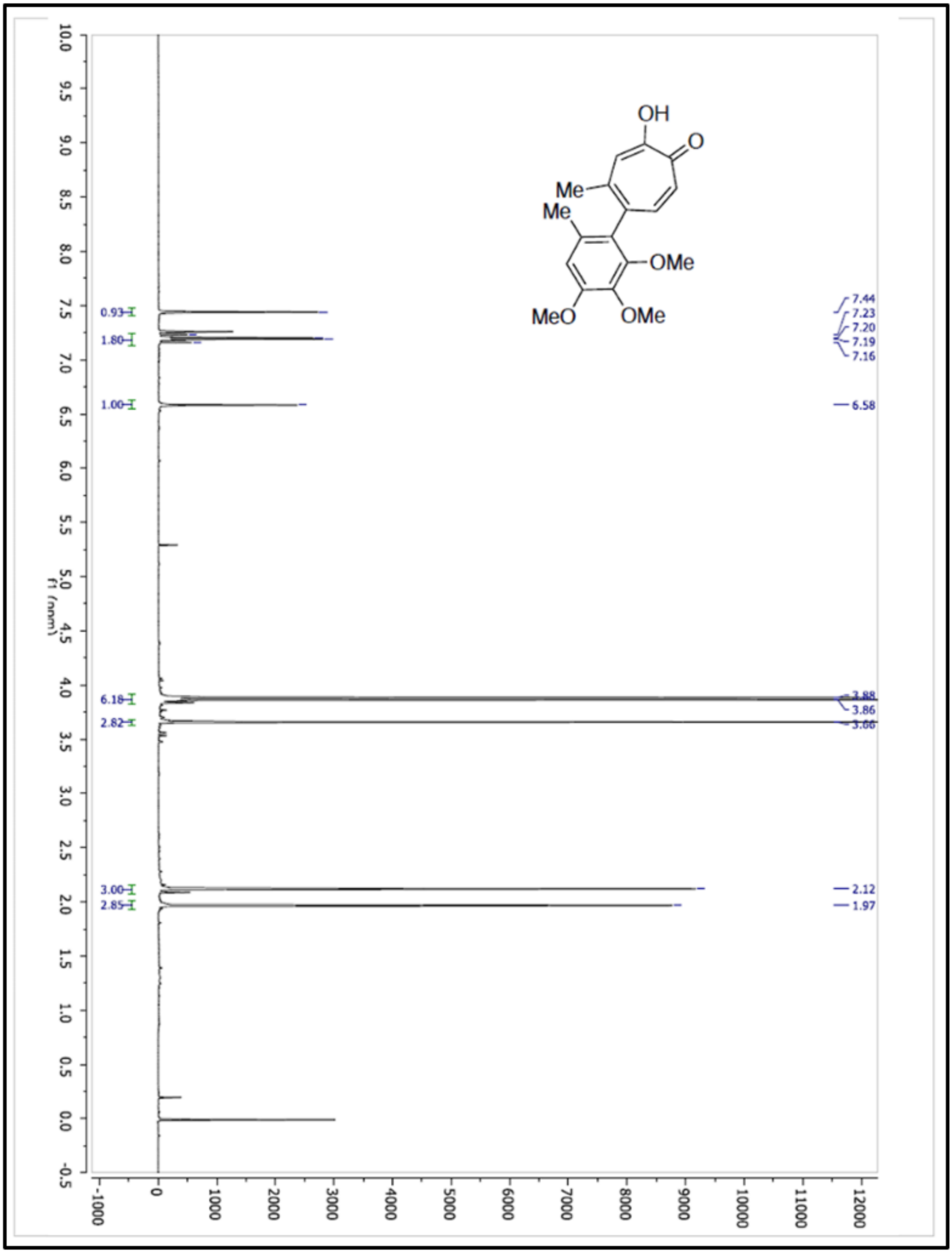

$^{13}C\{^{1}H\}$ **NMR (101 MHz, $CDCl_3$) of 20**

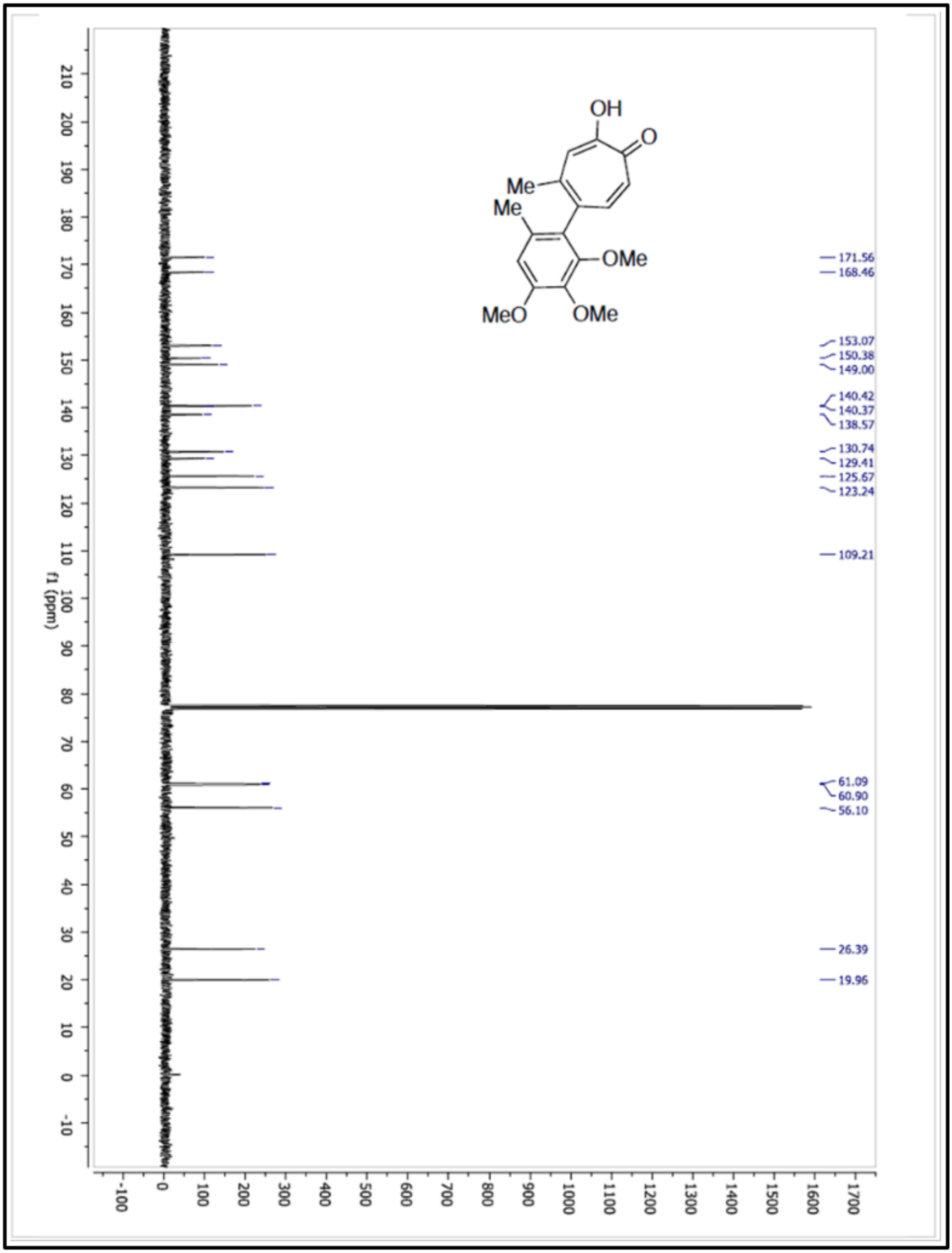

^{1}H NMR (400 MHz, $CDCl_3$) of MT-MTC (4)

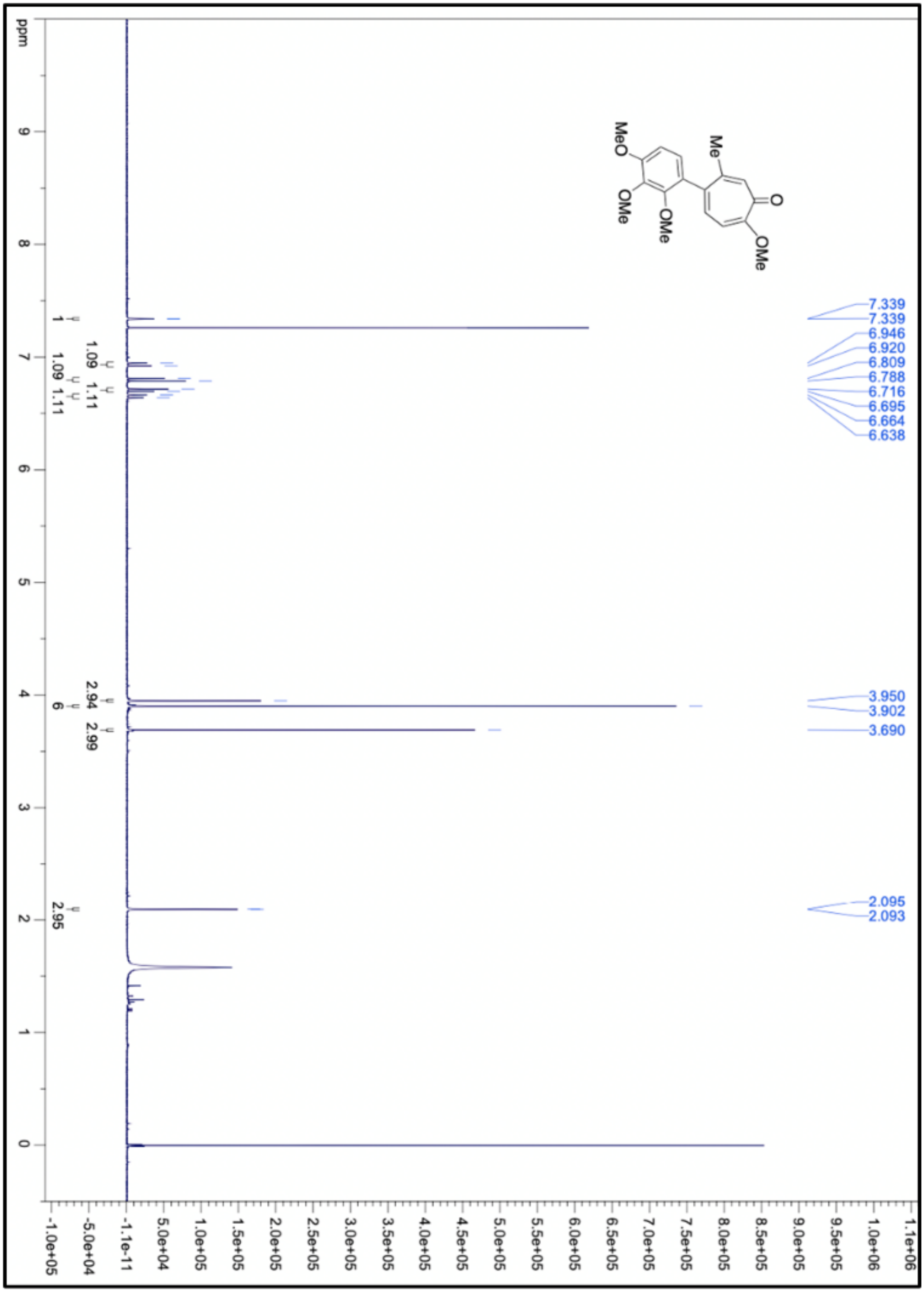

$^{13}C\{^1H\}$ NMR (101 MHz, $CDCl_3$) of MT-MTC (4)

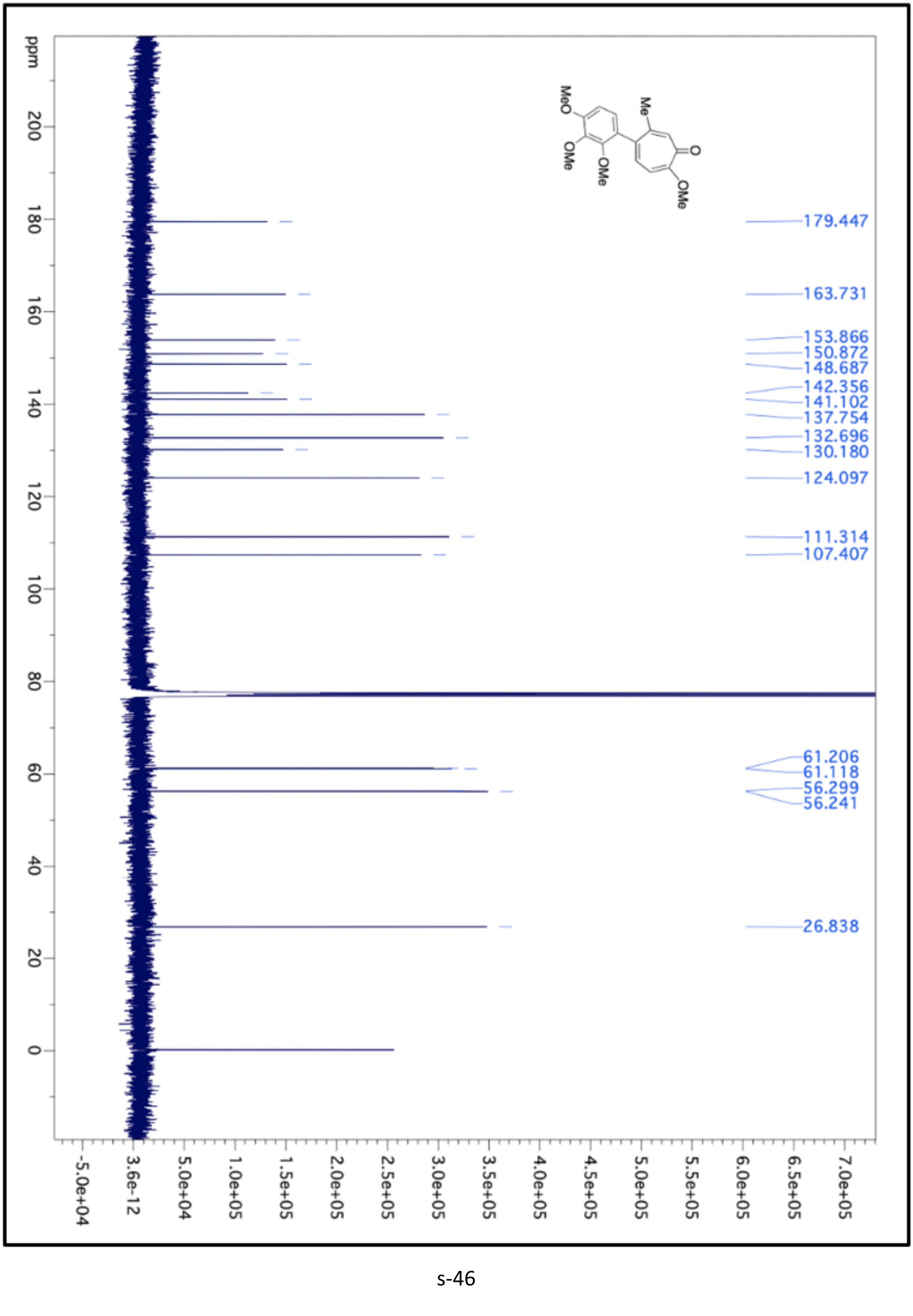

^{1}H NMR (400 MHz, CDCl$_3$) of DM-MTC (6)

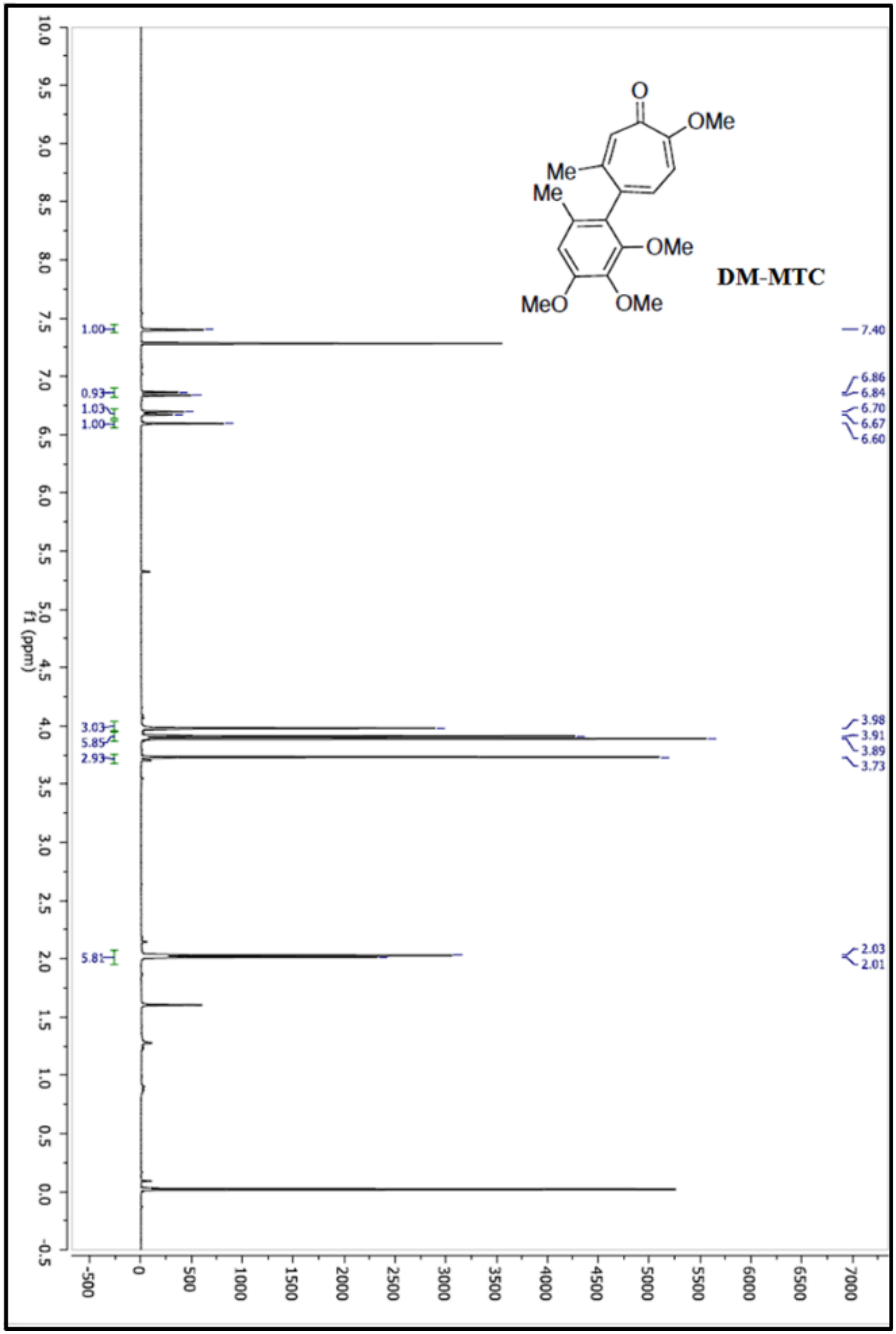

$^{13}C\{^{1}H\}$ NMR (101 MHz, $CDCl_3$) of DM-MTC (6)

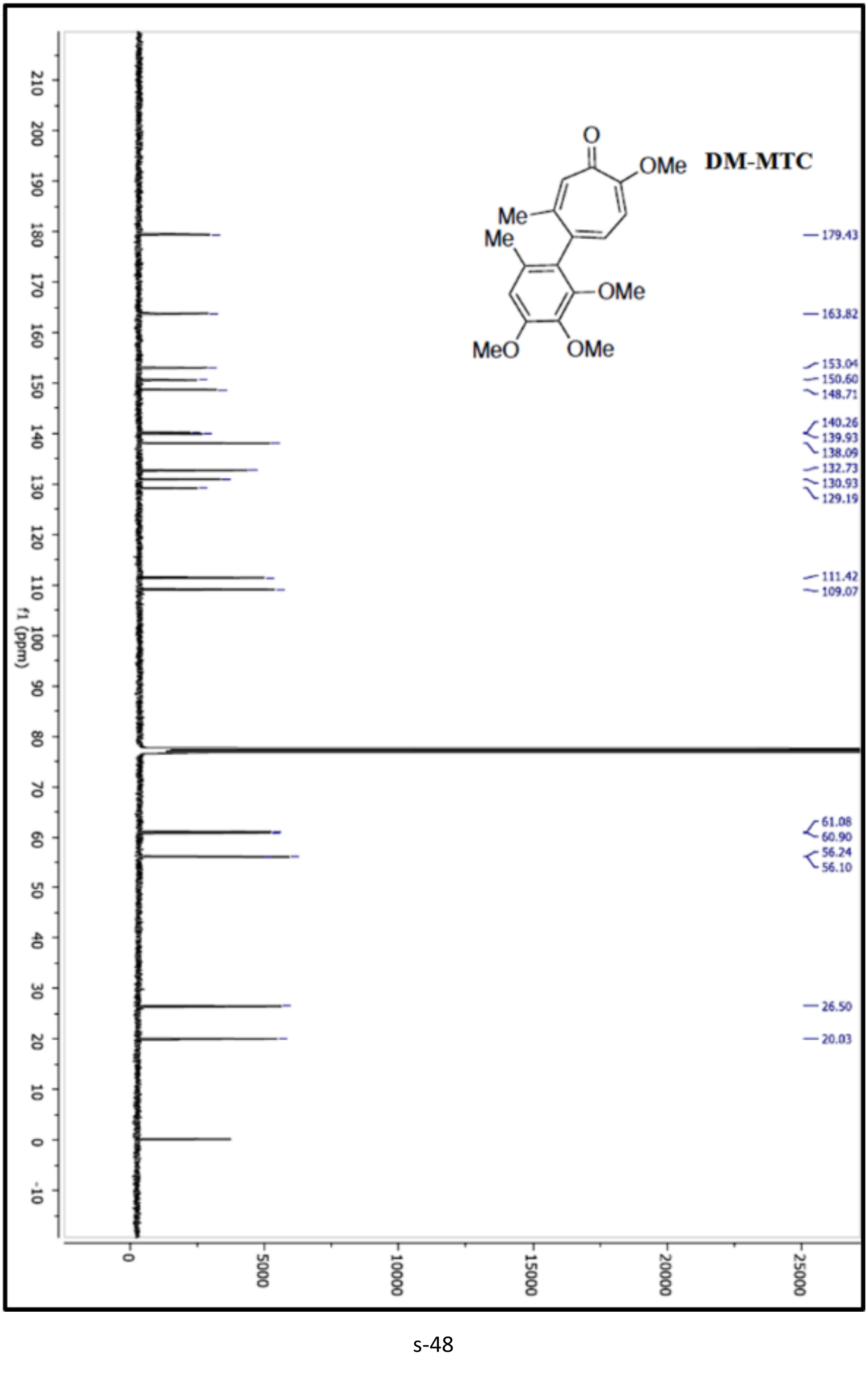

References

[14] Eli, O. S.; Bejcek, L. P.; Lyubimova, A.; Sackett, D. L.; Murelli, R. P. Intermolecular Oxidopyrylium (5 + 2) Cycloaddition/Reductive Ring-Opening Strategy for the Synthesis of α-Methoxytropones. *J. Org. Chem.* **2024**, *89* (23), 17813–17817. DOI: 10.1021/acs.joc.4c01989

[37] Azimi, S.; Khuttan, S.; Wu, J. Z.; Pal, R. K.; Gallicchio, E. Relative Binding Free Energy Calculations for Ligands with Diverse Scaffolds with the Alchemical Transfer Method. *J. Chem. Inf. Model* **2022**, *62* (2), 309–323. DOI: 10.1021/acs.jcim.1c01129.

[38] Sharma, A.; Aher, A.; Dynes, N. J.; Frey, D.; Katrukha, E. A.; Jaussi, R.; Grigoriev, I.; Croisier, M.; Kammerer, R. A.; Akhmanova, A.; Gönczy, P.; Steinmetz, M. O. Centriolar CPAP/SAS-4 Imparts Slow Processive Microtubule Growth. *Dev. Cell.* **2016**, *37* (4), 362–376. DOI: 10.1016/j.devcel.2016.04.024.

[39] (a) Agyemang, N.; Murelli, R. Synthesis of 5-Hydroxy-4-Methoxy-2-Methylpyrylium Trifluoromethanesulfonate from Kojic Acid. *Org. Synth.* **2019**, *96*, 494–510. DOI: 10.15227/orgsyn.096.0494. (b) Meck, C.; Mohd, N.; Murelli, R. P. An Oxidopyrylium Cyclization/Ring-Opening Route to Polysubstituted α-Hydroxytropolones. *Org. Lett.* **2012**, *14* (23), 5988–5991. DOI: 10.10121/ol302892g.

[40] Bursch, M.; Mewes, J. M.; Hansen, A.; Grimme, S. Best-Practice DFT Protocols for Basic Molecular Computational Chemistry. *Angew. Chem. Int. Ed.* **2022**, *61* (42). DOI: 10.1002/anie.202205735.

[43] Montecinos, F.; Loew, M.; Choi, T. I.; Bane, S. L.; Sackett, D. L. "Interaction of Colchicine-Site Ligands With the Blood Cell-Specific Isotype of β-Tubulin-Notable Affinity for Benzimidazoles" *Front. Cell Dev. Biol.* **2022**, *10*, 884287.

[45] GitHub - Gallicchio-Lab/AToM-OpenMM: OpenMM-based framework for absolute and relative binding free energy calculations with the Alchemical Transfer Method. https://github.com/Gallicchio-Lab/AToM-OpenMM (accessed 2023-08-19).

[46] GitHub - Gallicchio-Lab/openmm-atmmetaforce-plugin: An OpenMM plugin that implements the Alchemical Transfer Potential. https://github.com/Gallicchio-Lab/openmm-atmmetaforce-plugin (accessed 2023-08-19).

[47] (a) Eastman, P.; Swails, J.; Chodera, J. D.; McGibbon, R. T.; Zhao, Y.; Beauchamp, K. A.; Wang, L. P.; Simmonett, A. C.; Harrigan, M. P.; Stern, C. D.; Wiewiora, R. P.; Brooks, B. R.; Pande, V. S. OpenMM 7: Rapid Development of High-Performance Algorithms for Molecular Dynamics. *PLoS. Comput. Biol.* **2017**, *13* (7), e1005659. DOI: 10.1371/journal.pcbi.1005659. (b) GitHub - openmm/openmm: OpenMM is a toolkit for molecular simulation using high-performance GPU code. https://github.com/openmm/openmm (accessed 2023-08-19).

[48] Maier, J. A.; Martinez, C.; Kasavajhala, K.; Wickstrom, L.; Hauser, K. E.; Simmerling, C. Ff14SB: Improving the Accuracy of Protein Side Chain and Backbone Parameters from Ff99SB. *J. Chem. Theory. Comput.* **2015**, *11* (8), 3696–3713. DOI: 10.1021/acs.jctc.5b00255/suppl_file/ct5b00255_si_001.pdf.

[49] Wang, J.; Wang, W.; Kollman, P. A.; Case, D. A. Automatic Atom Type and Bond Type Perception in Molecular Mechanical Calculations. *J. Mol. Graph. Model.* **2006**, *25* (2), 247–260. DOI: 10.1016/j.jmgm.2005.12.005.
