# Supplementary material for: Deconstruction of Desacetamidocolchicine’s B Ring Reveals a Class 3 Atropisomeric AC Ring with Tubulin Binding Properties
Source: J Org Chem. 2025 May 27;90(22):7246–58. doi: 10.1021/acs.joc.5c00284 (PMC12150326; doi:10.1021/acs.joc.5c00284)

National Cancer Institute Developmental Therapeutics Program
Dose Response Curves

NSC: 830095 | SSPL: 0GZS | EXP. ID: 2106NS48

(aR)-6 | Test Date: June 21, 2021

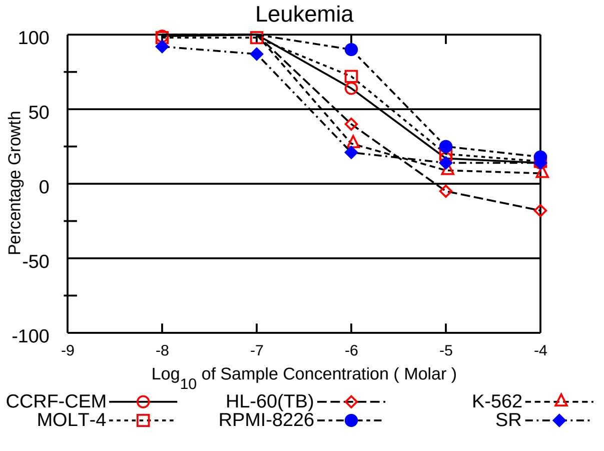

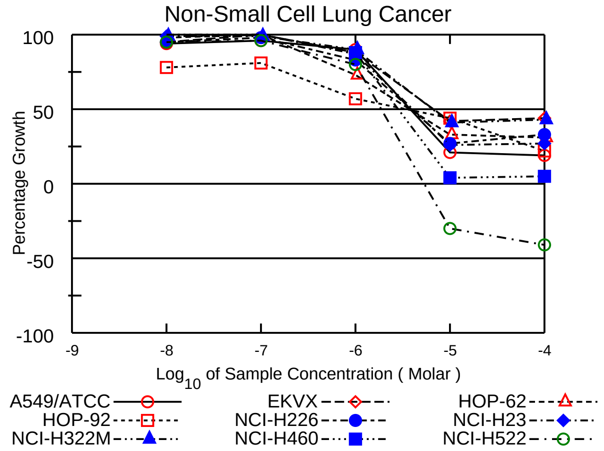

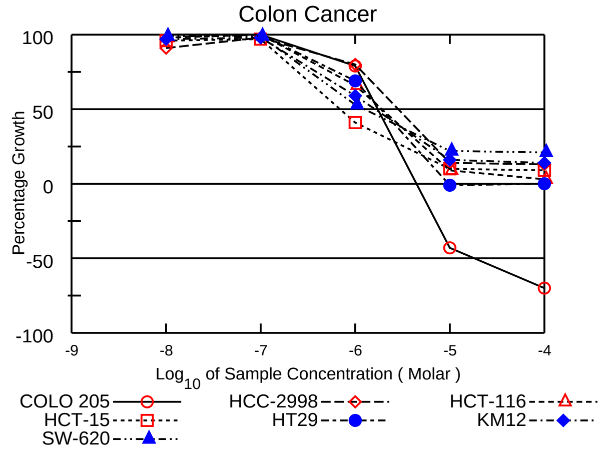

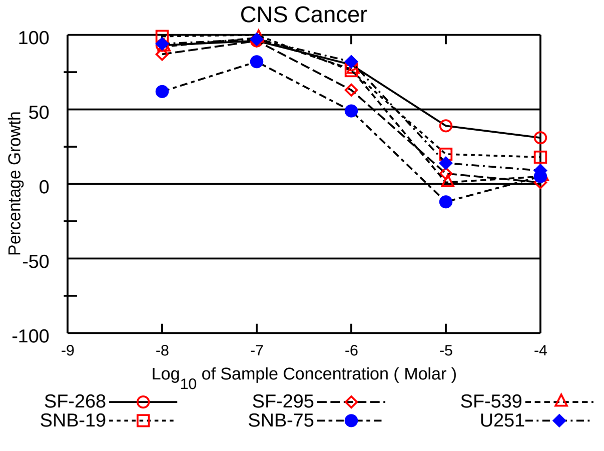

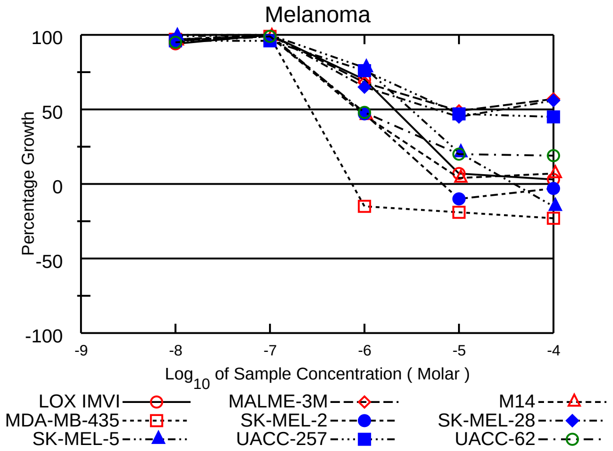

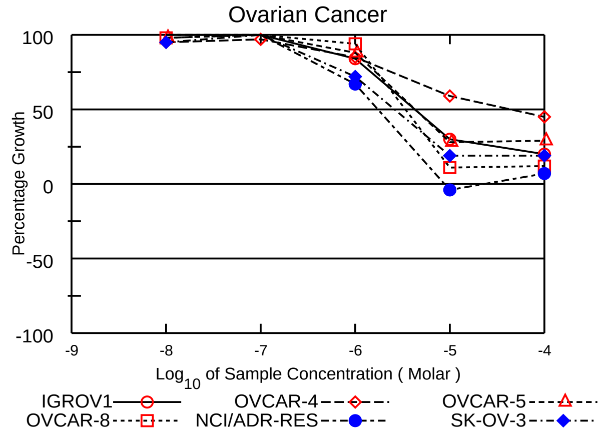

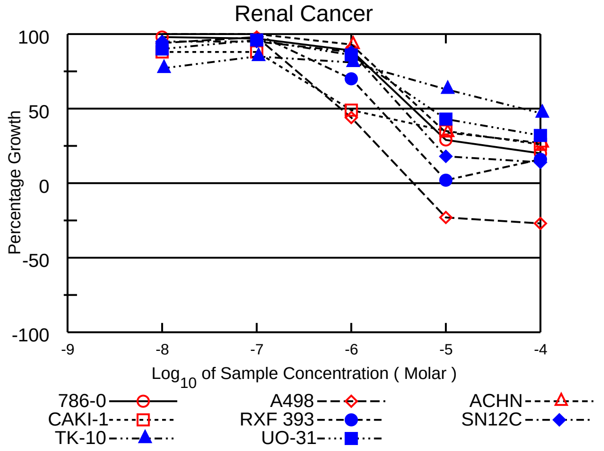

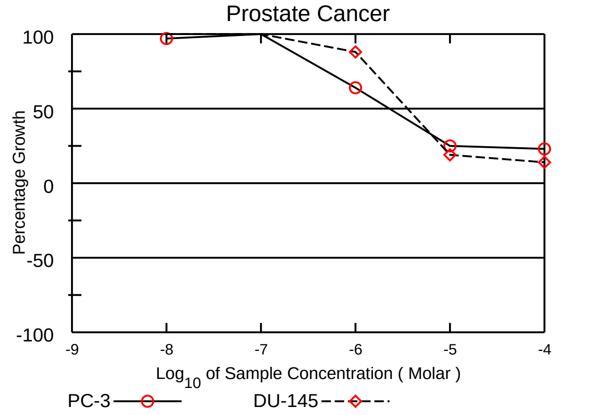

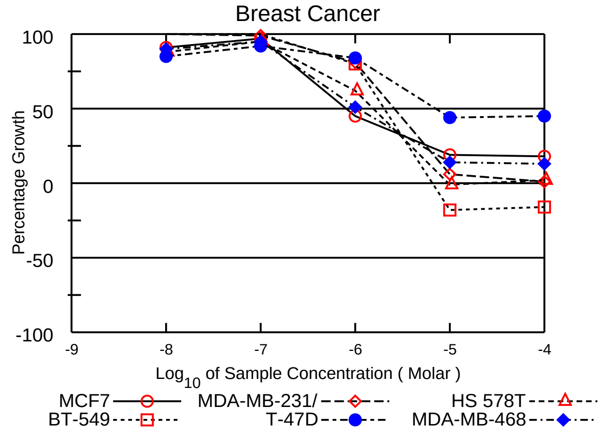

National Cancer Institute Developmental Therapeutics Program Mean Graphs	NSC : 830095	Units :Molar	SSPL :0GZS	EXP. ID :2106NS48
	(aR)-6		Test Date :June 21, 2021	

Panel/Cell Line	Log_{10}GI50	Log_{10}TGI	Log_{10}LC50
**Leukemia**			
CCRF-CEM	-5.71	> -4.00	> -4.00
HL-60(TB)	-6.16	-5.10	> -4.00
K-562	-6.31	> -4.00	> -4.00
MOLT-4	-5.58	> -4.00	> -4.00
RPMI-8226	-5.39	> -4.00	> -4.00
SR	-6.44	> -4.00	> -4.00
**Non-Small Cell Lung Cancer**			
A549/ATCC	-5.42	> -4.00	> -4.00
EKVX	-5.18	> -4.00	> -4.00
HOP-62	-5.42	> -4.00	> -4.00
HOP-92	-5.45	> -4.00	> -4.00
NCI-H226	-5.41	> -4.00	> -4.00
NCI-H23	-5.39	> -4.00	> -4.00
NCI-H322M	-5.18	> -4.00	> -4.00
NCI-H460	-5.55	> -4.00	> -4.00
NCI-H522	-5.73	-5.27	> -4.00
**Colon Cancer**			
COLO 205	-5.76	-5.35	-4.74
HCC-2998	-5.55	> -4.00	> -4.00
HCT-116	-5.71	> -4.00	> -4.00
HCT-15	-6.17	> -4.00	> -4.00
HT29	-5.73	-5.02	> -4.00
KM12	-5.79	> -4.00	> -4.00
SW-620	-5.91	> -4.00	> -4.00
**CNS Cancer**			
SF-268	-5.26	> -4.00	> -4.00
SF-295	-5.76	> -4.00	> -4.00
SF-539	-5.65	> -4.00	> -4.00
SNB-19	-5.53	> -4.00	> -4.00
SNB-75	-6.04		> -4.00
U251	-5.53	> -4.00	> -4.00
**Melanoma**			
LOX IMVI	-5.69	> -4.00	> -4.00
MALME-3M		> -4.00	> -4.00
M14	-6.07	> -4.00	> -4.00
MDA-MB-435	-6.57	-6.13	> -4.00
SK-MEL-2	-6.05	-5.18	> -4.00
SK-MEL-28		> -4.00	> -4.00
SK-MEL-5	-5.50	-4.42	> -4.00
UACC-257	-5.11	> -4.00	> -4.00
UACC-62	-6.03	> -4.00	> -4.00
**Ovarian Cancer**			
IGROV1	-5.37	> -4.00	> -4.00
OVCAR-4	-4.35	> -4.00	> -4.00
OVCAR-5	-5.36	> -4.00	> -4.00
OVCAR-8	-5.47	> -4.00	> -4.00
NCI/ADR-RES	-5.76		> -4.00
SK-OV-3	-5.59	> -4.00	> -4.00
**Renal Cancer**			
786-0	-5.35	> -4.00	> -4.00
A498	-6.10	-5.34	> -4.00
ACHN	-5.27	> -4.00	> -4.00
CAKI-1	-6.03	> -4.00	> -4.00
RXF 393	-5.71	> -4.00	> -4.00
SN12C	-5.46	> -4.00	> -4.00
TK-10	-4.18	> -4.00	> -4.00
UO-31	-5.16	> -4.00	> -4.00
**Prostate Cancer**			
PC-3	-5.64	> -4.00	> -4.00
DU-145	-5.45	> -4.00	> -4.00
**Breast Cancer**			
MCF7	-6.11	> -4.00	> -4.00
MDA-MB-231/ATCC	-5.59	> -4.00	> -4.00
HS 578T	-5.81		> -4.00
BT-549	-5.69	-5.18	> -4.00
T-47D	-5.15	> -4.00	> -4.00
MDA-MB-468	-5.97	> -4.00	> -4.00
_MID	-5.62	-4.2	-4.01
Delta	0.95	1.93	0.73
Range	2.39	2.13	0.74

GI50

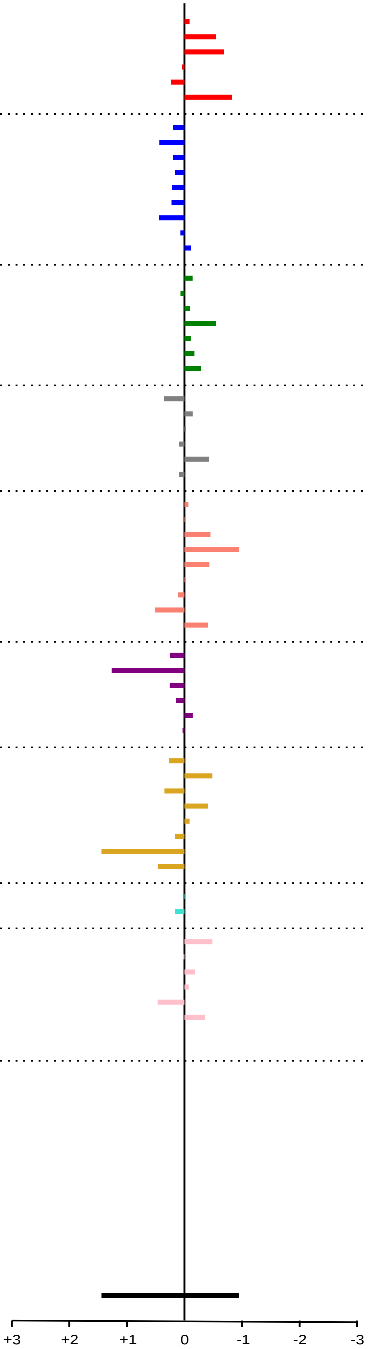

TGI

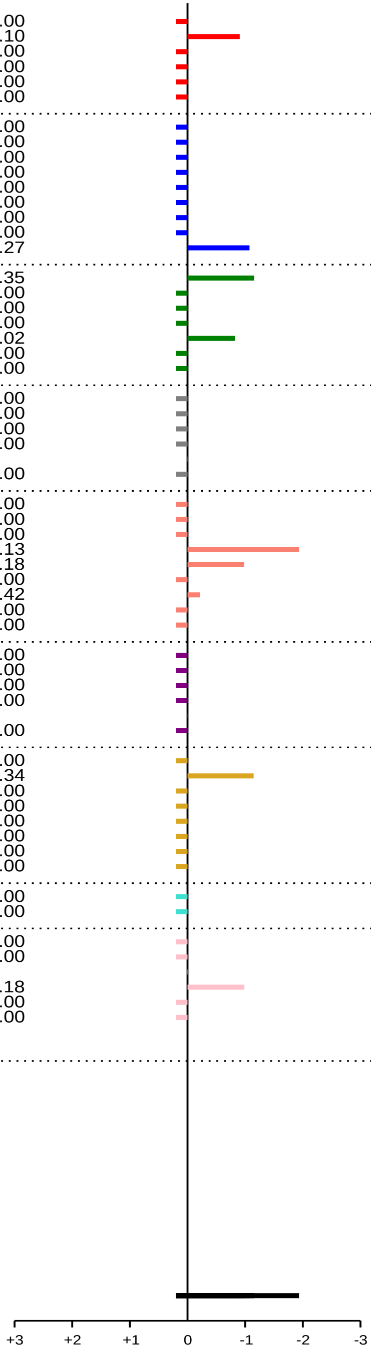

LC50

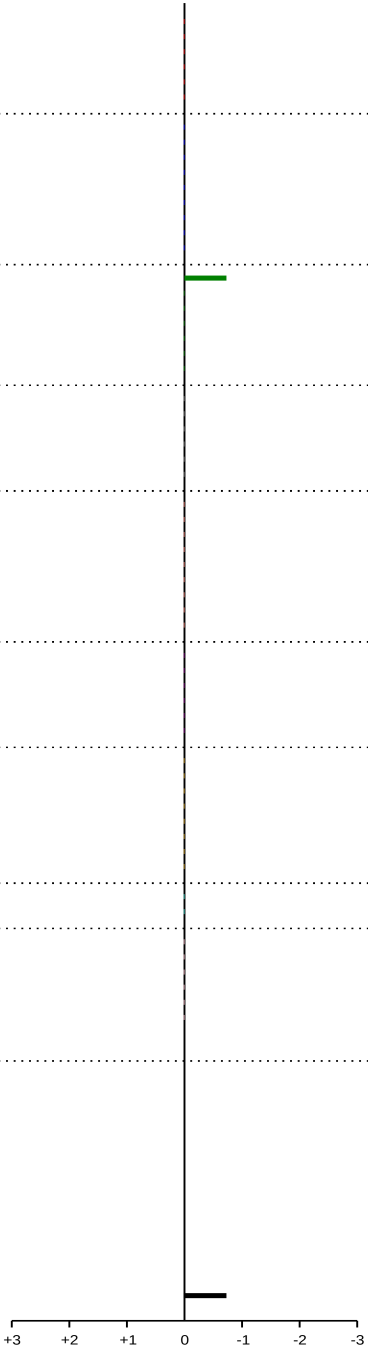

National Cancer Institute Developmental Therapeutics Program<br>Dose Response Curves	NSC: 830096	SSPL: 0GZS	EXP. ID: 2106NS48
	(aS)-6	Test Date: June 21, 2021	

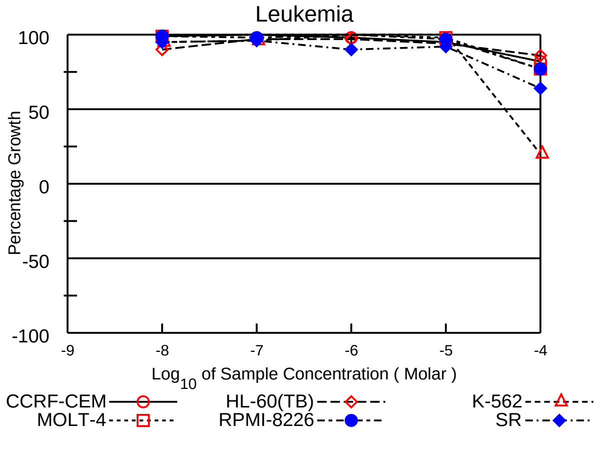

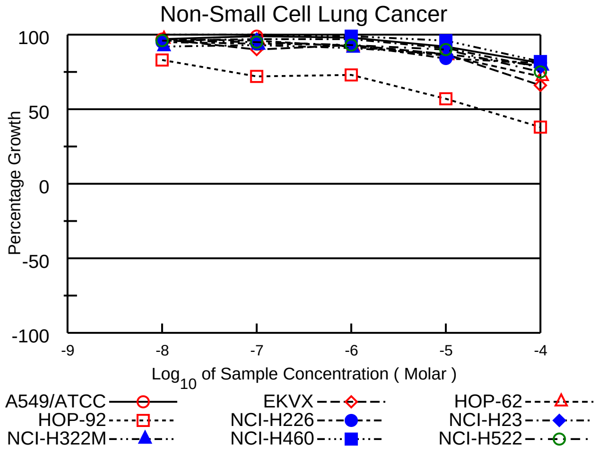

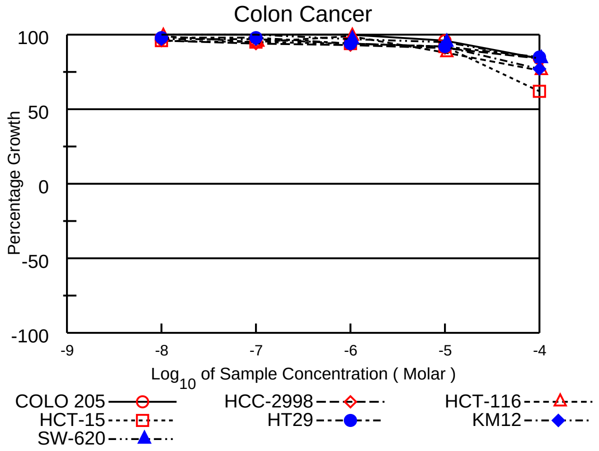

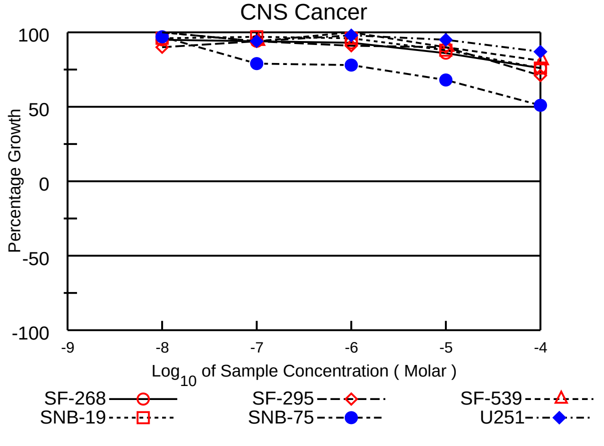

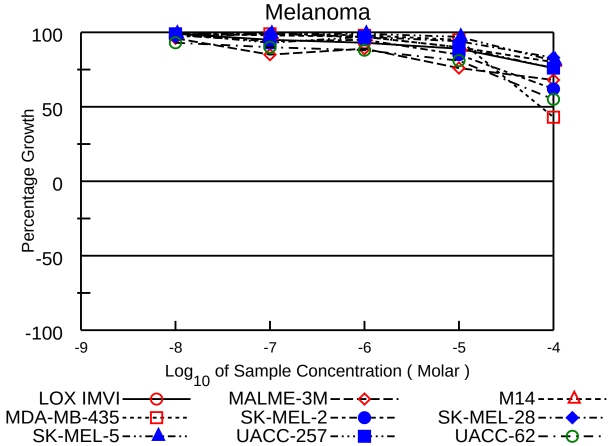

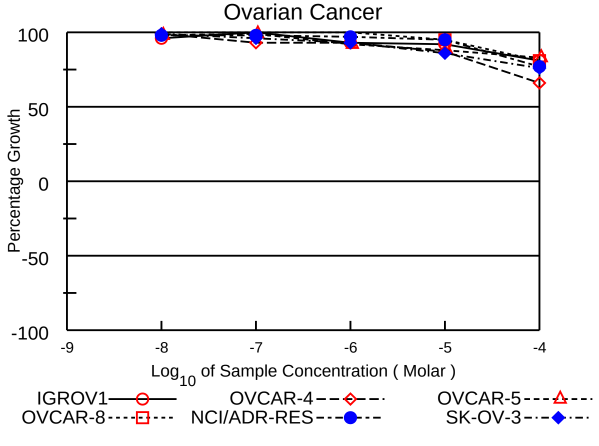

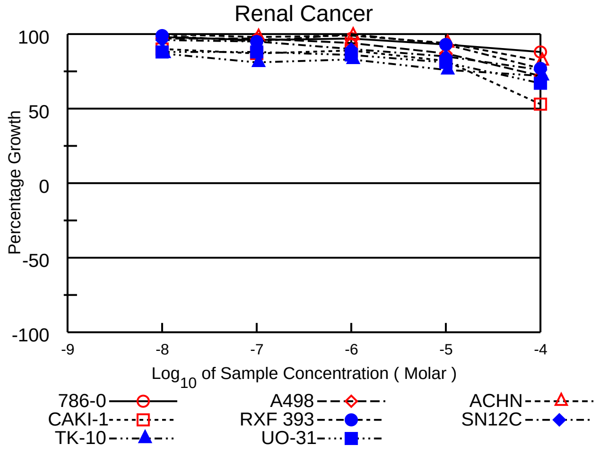

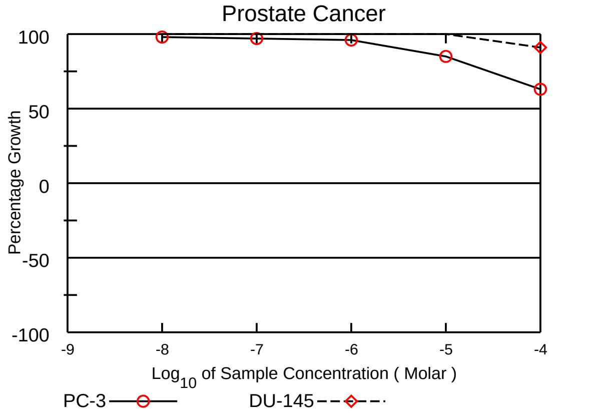

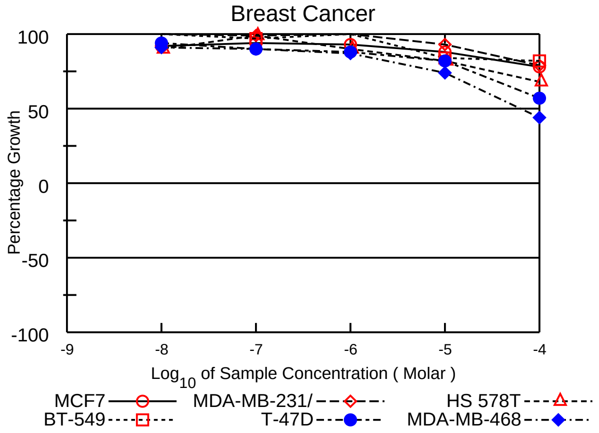

National Cancer Institute Developmental Therapeutics Program Mean Graphs	NSC : 830096	Units :Molar	SSPL :0GZS	EXP. ID :2106NS48
	(aS)-6		Test Date :June 21, 2021	

Panel/Cell Line	Log_{10}GI50	Log_{10}TGI	Log_{10}LC50
**Leukemia**			
CCRF-CEM	> -4.00	> -4.00	> -4.00
HL-60(TB)	> -4.00	> -4.00	> -4.00
K-562	-4.38	> -4.00	> -4.00
MOLT-4	> -4.00	> -4.00	> -4.00
RPMI-8226	> -4.00	> -4.00	> -4.00
SR	> -4.00	> -4.00	> -4.00
**Non-Small Cell Lung Cancer**			
A549/ATCC	> -4.00	> -4.00	> -4.00
EKVX	> -4.00	> -4.00	> -4.00
HOP-62	> -4.00	> -4.00	> -4.00
HOP-92	-4.65	> -4.00	> -4.00
NCI-H226	> -4.00	> -4.00	> -4.00
NCI-H23	> -4.00	> -4.00	> -4.00
NCI-H322M	> -4.00	> -4.00	> -4.00
NCI-H460	> -4.00	> -4.00	> -4.00
NCI-H522	> -4.00	> -4.00	> -4.00
**Colon Cancer**			
COLO 205	> -4.00	> -4.00	> -4.00
HCC-2998	> -4.00	> -4.00	> -4.00
HCT-116	> -4.00	> -4.00	> -4.00
HCT-15	> -4.00	> -4.00	> -4.00
HT29	> -4.00	> -4.00	> -4.00
KM12	> -4.00	> -4.00	> -4.00
SW-620	> -4.00	> -4.00	> -4.00
**CNS Cancer**			
SF-268	> -4.00	> -4.00	> -4.00
SF-295	> -4.00	> -4.00	> -4.00
SF-539	> -4.00	> -4.00	> -4.00
SNB-19	> -4.00	> -4.00	> -4.00
SNB-75	> -4.00	> -4.00	> -4.00
U251	> -4.00	> -4.00	> -4.00
**Melanoma**			
LOX IMVI	> -4.00	> -4.00	> -4.00
MALME-3M	> -4.00	> -4.00	> -4.00
M14	> -4.00	> -4.00	> -4.00
MDA-MB-435	-4.14	> -4.00	> -4.00
SK-MEL-2	> -4.00	> -4.00	> -4.00
SK-MEL-28	> -4.00	> -4.00	> -4.00
SK-MEL-5	> -4.00	> -4.00	> -4.00
UACC-257	> -4.00	> -4.00	> -4.00
UACC-62	> -4.00	> -4.00	> -4.00
**Ovarian Cancer**			
IGROV1	> -4.00	> -4.00	> -4.00
OVCAR-4	> -4.00	> -4.00	> -4.00
OVCAR-5	> -4.00	> -4.00	> -4.00
OVCAR-8	> -4.00	> -4.00	> -4.00
NCI/ADR-RES	> -4.00	> -4.00	> -4.00
SK-OV-3	> -4.00	> -4.00	> -4.00
**Renal Cancer**			
786-0	> -4.00	> -4.00	> -4.00
A498	> -4.00	> -4.00	> -4.00
ACHN	> -4.00	> -4.00	> -4.00
CAKI-1	> -4.00	> -4.00	> -4.00
RXF 393	> -4.00	> -4.00	> -4.00
SN12C	> -4.00	> -4.00	> -4.00
TK-10	> -4.00	> -4.00	> -4.00
UO-31	> -4.00	> -4.00	> -4.00
**Prostate Cancer**			
PC-3	> -4.00	> -4.00	> -4.00
DU-145	> -4.00	> -4.00	> -4.00
**Breast Cancer**			
MCF7	> -4.00	> -4.00	> -4.00
MDA-MB-231/ATCC	> -4.00	> -4.00	> -4.00
HS 578T	> -4.00	> -4.00	> -4.00
BT-549	> -4.00	> -4.00	> -4.00
T-47D	> -4.00	> -4.00	> -4.00
MDA-MB-468	-4.20	> -4.00	> -4.00
_MID	-4.02	-4.0	-4.0
Delta	0.63	0	0
Range	0.65	0.0	0.0

GI50

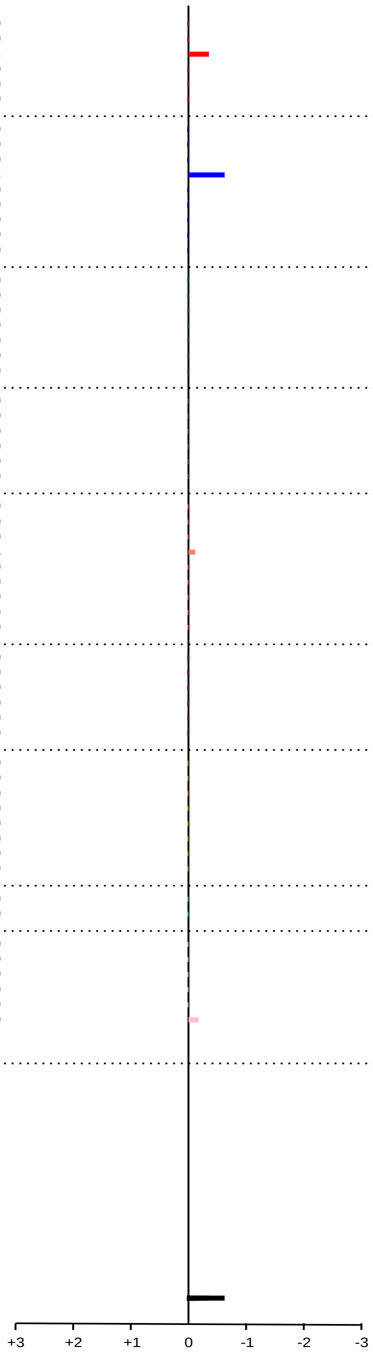

TGI

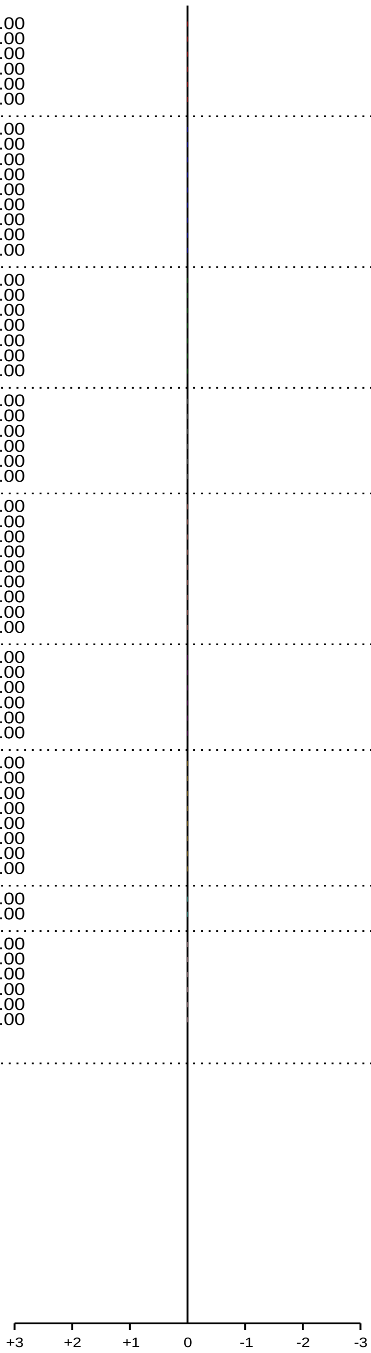

LC50

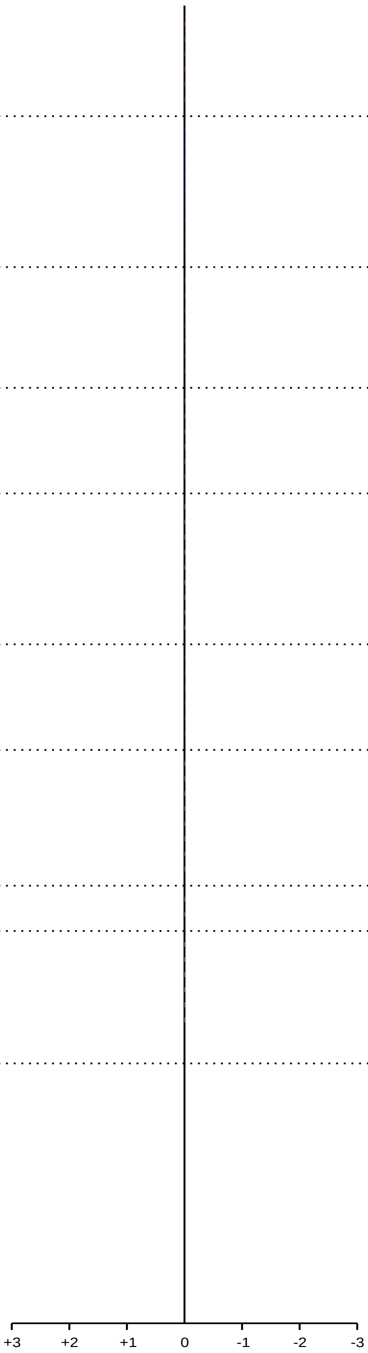

National Cancer Institute Developmental Therapeutics Program
Dose Response Curves

NSC: 830097 | SSPL: 0GZS | EXP. ID: 2106NS48

(aS)-*iso*-6 | Test Date: June 21, 2021

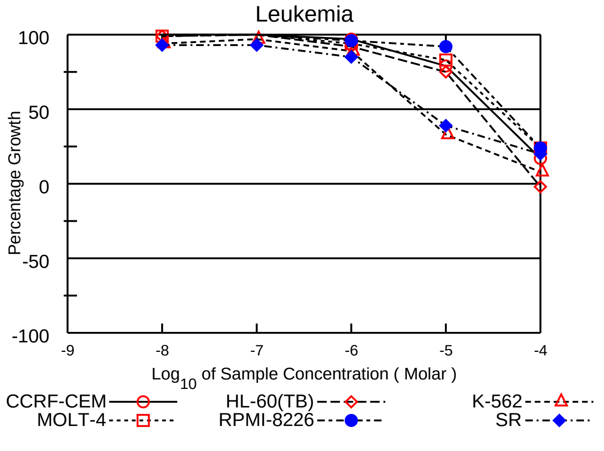

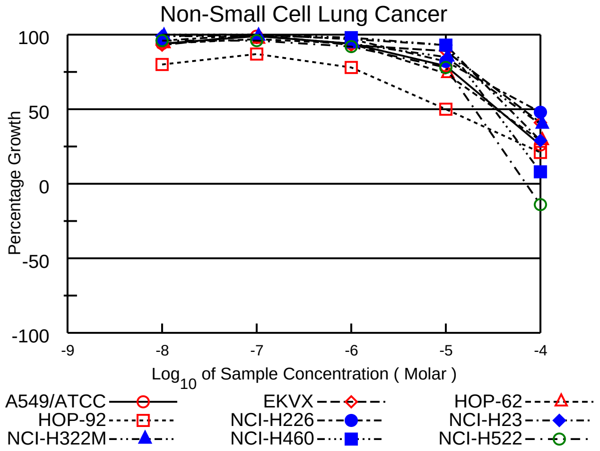

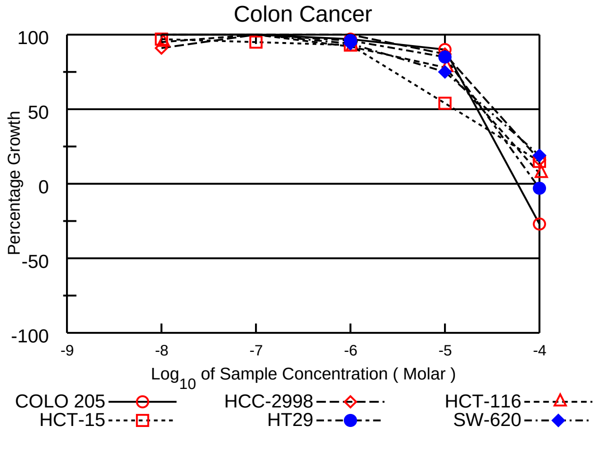

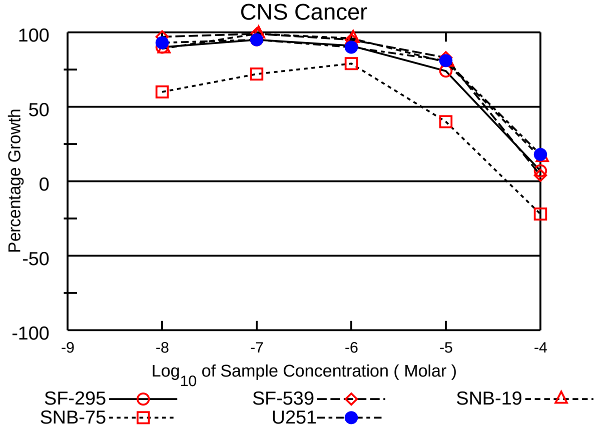

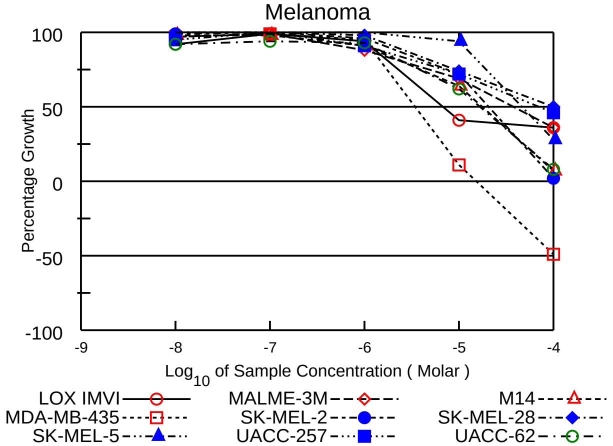

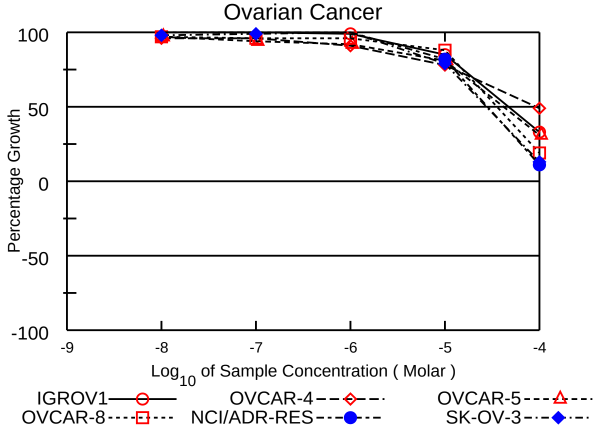

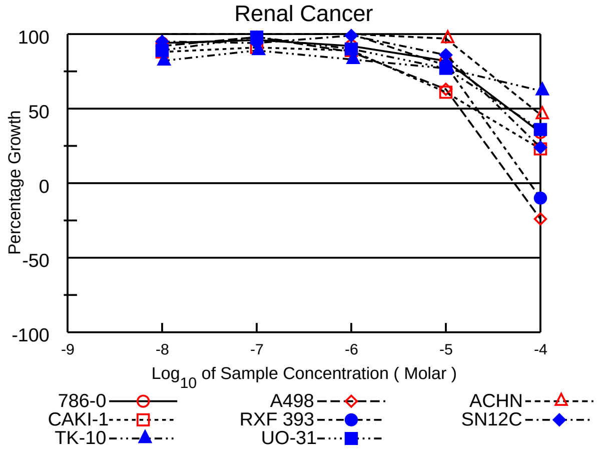

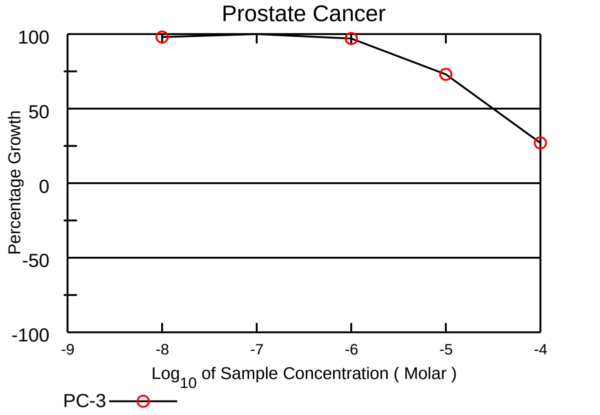

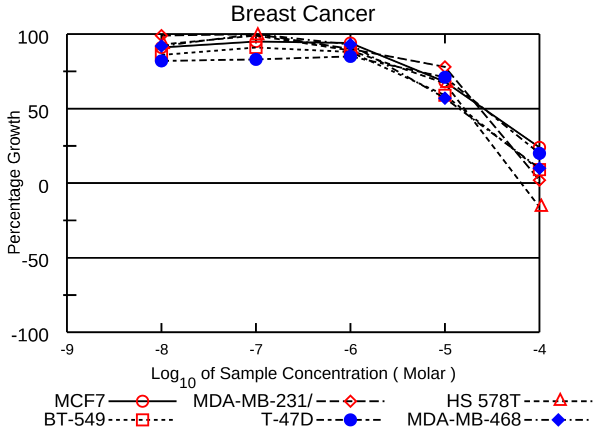

National Cancer Institute Developmental Therapeutics Program Mean Graphs	NSC : 830097	Units :Molar	SSPL :0GZS	EXP. ID :2106NS48
	(aS)-*iso*-6		Test Date :June 21, 2021	

Panel/Cell Line	Log_{10} GI50	Log_{10} TGI	Log_{10} LC50
**Leukemia**			
CCRF-CEM	-4.53	> -4.00	> -4.00
HL-60(TB)	-4.68	-4.02	> -4.00
K-562	-5.30	> -4.00	> -4.00
MOLT-4	-4.44	> -4.00	> -4.00
RPMI-8226	-4.38	> -4.00	> -4.00
SR	-5.24	> -4.00	> -4.00
**Non-Small Cell Lung Cancer**			
A549/ATCC	-4.45	> -4.00	> -4.00
EKVX	-4.18	> -4.00	> -4.00
HOP-62	-4.46	> -4.00	> -4.00
HOP-92	-4.99	> -4.00	> -4.00
NCI-H226	-4.06	> -4.00	> -4.00
NCI-H23	-4.33	> -4.00	> -4.00
NCI-H322M	-4.22	> -4.00	> -4.00
NCI-H460	-4.50	> -4.00	> -4.00
NCI-H522	-4.69	-4.15	> -4.00
**Colon Cancer**			
COLO 205	-4.66	-4.23	> -4.00
HCC-2998	-4.48	> -4.00	> -4.00
HCT-116	-4.60	> -4.00	> -4.00
HCT-15	-4.89	> -4.00	> -4.00
HT29	-4.60	-4.03	> -4.00
SW-620	-4.55	> -4.00	> -4.00
**CNS Cancer**			
SF-295	-4.64	> -4.00	> -4.00
SF-539	-4.59	> -4.00	> -4.00
SNB-19	-4.53	> -4.00	> -4.00
SNB-75	-5.27	-4.36	> -4.00
U251	-4.51	> -4.00	> -4.00
**Melanoma**			
LOX IMVI	-5.18	> -4.00	> -4.00
MALME-3M	-4.42	> -4.00	> -4.00
M14	-4.76	> -4.00	> -4.00
MDA-MB-435	-5.46	-4.81	> -4.00
SK-MEL-2	-4.69	> -4.00	> -4.00
SK-MEL-28	> -4.00	> -4.00	> -4.00
SK-MEL-5	-4.34	> -4.00	> -4.00
UACC-257	-4.15	> -4.00	> -4.00
UACC-62	-4.78	> -4.00	> -4.00
**Ovarian Cancer**			
IGROV1	-4.32	> -4.00	> -4.00
OVCAR-4	-4.04	> -4.00	> -4.00
OVCAR-5	-4.38	> -4.00	> -4.00
OVCAR-8	-4.45	> -4.00	> -4.00
NCI/ADR-RES	-4.55	> -4.00	> -4.00
SK-OV-3	-4.57	> -4.00	> -4.00
**Renal Cancer**			
786-0	-4.33	> -4.00	> -4.00
A498	-4.85	-4.28	> -4.00
ACHN	-4.07	> -4.00	> -4.00
CAKI-1	-4.71	> -4.00	> -4.00
RXF 393	-4.68	-4.12	> -4.00
SN12C	-4.42	> -4.00	> -4.00
TK-10	> -4.00	> -4.00	> -4.00
UO-31	-4.34	> -4.00	> -4.00
**Prostate Cancer**			
PC-3	-4.50	> -4.00	> -4.00
**Breast Cancer**			
MCF7	-4.60	> -4.00	> -4.00
MDA-MB-231/ATCC	-4.63	> -4.00	> -4.00
HS 578T	-4.80	-4.19	> -4.00
BT-549	-4.82	> -4.00	> -4.00
T-47D	-4.58	> -4.00	> -4.00
MDA-MB-468	-4.85	> -4.00	> -4.00
_MID	-4.57	-4.04	-4.0
Delta	0.89	0.77	0
Range	1.46	0.81	0.0

GI50

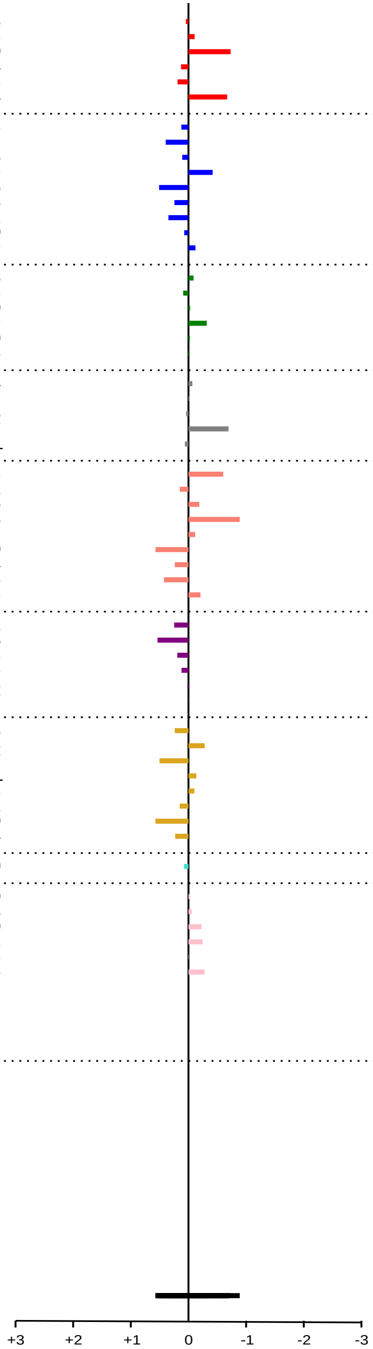

TGI

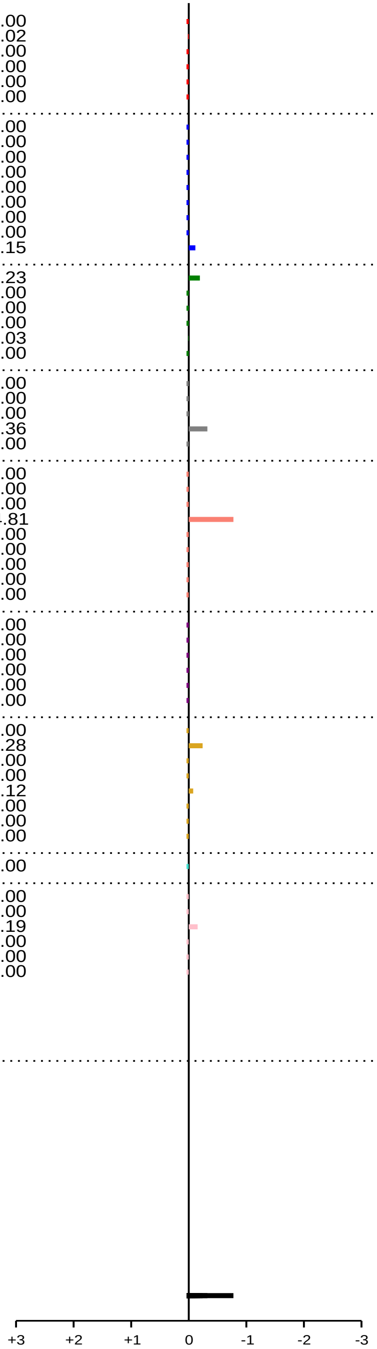

LC50

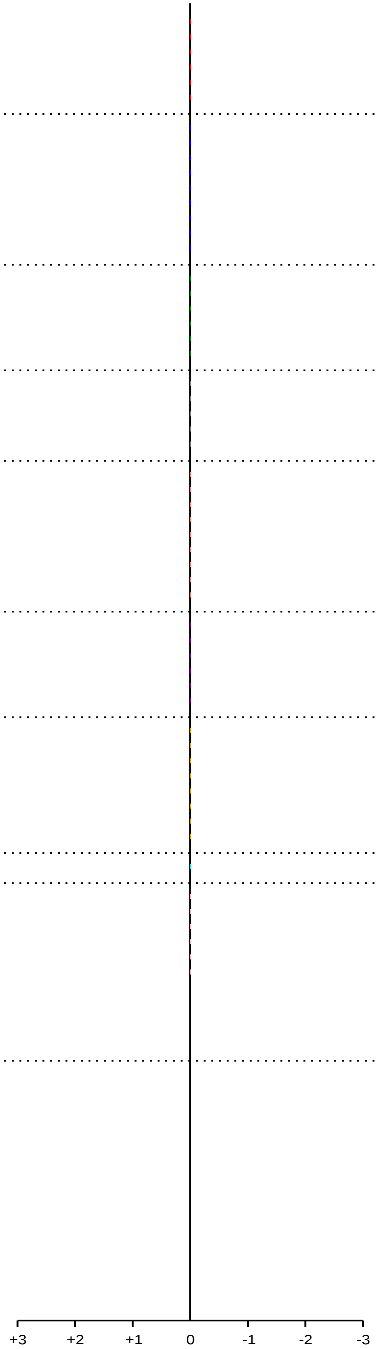

National Cancer Institute Developmental Therapeutics Program
Dose Response Curves

NSC: 830098	SSPL: 0GZS	EXP. ID: 2106NS48
(aR)-*iso*-6	Test Date: June 21, 2021	

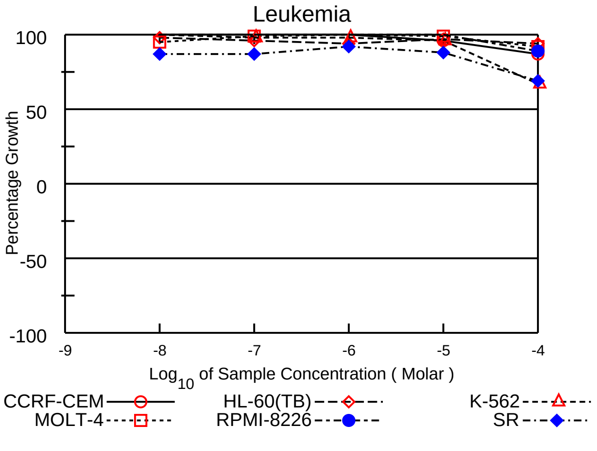

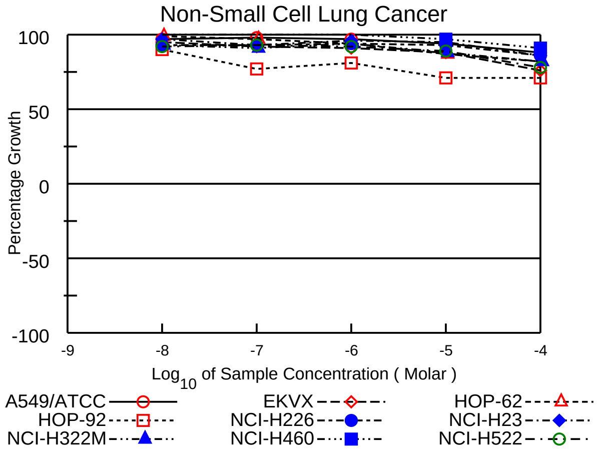

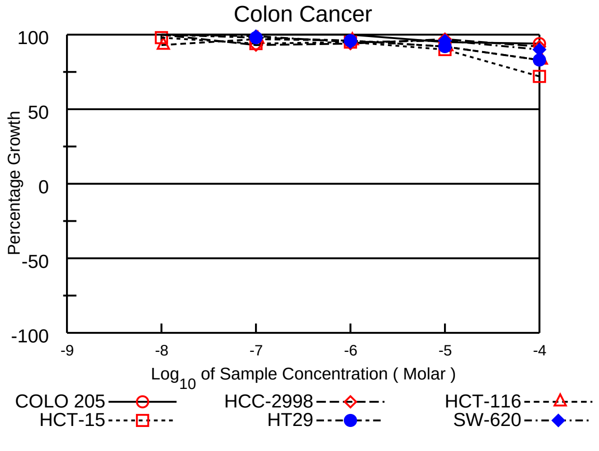

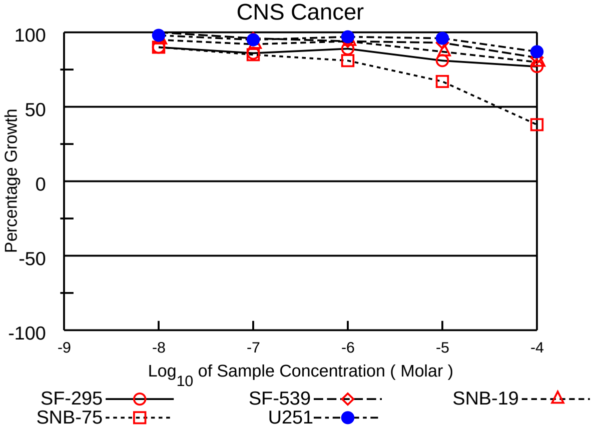

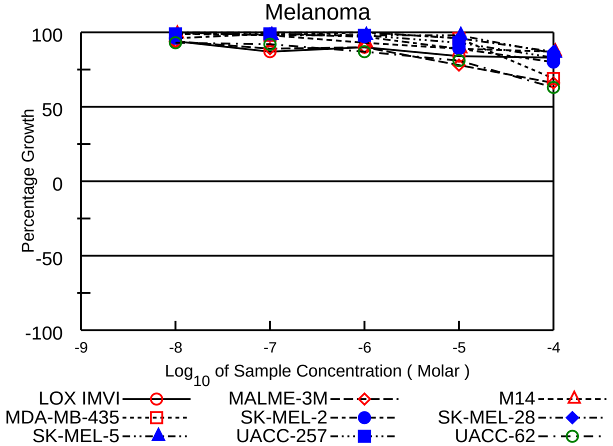

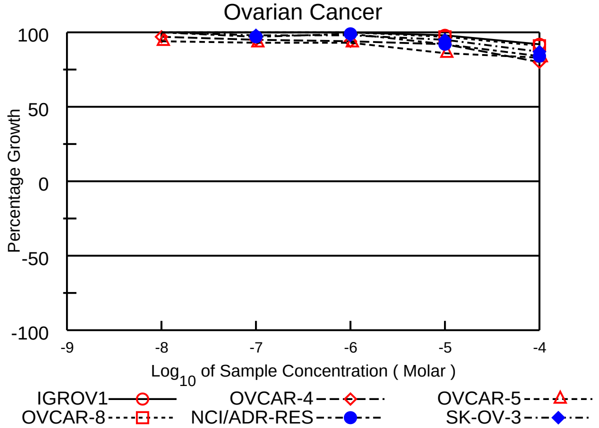

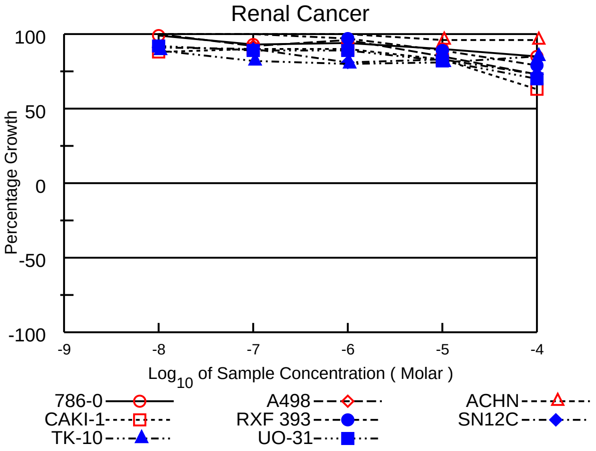

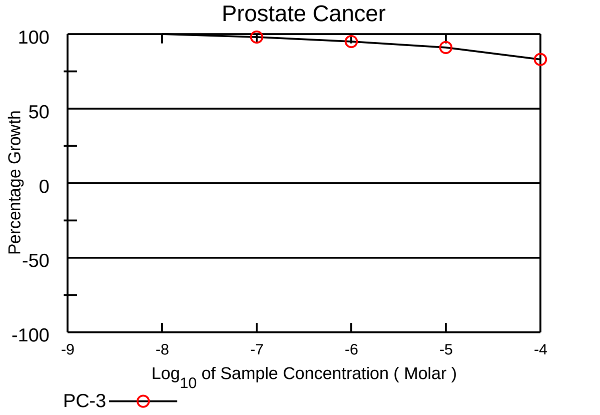

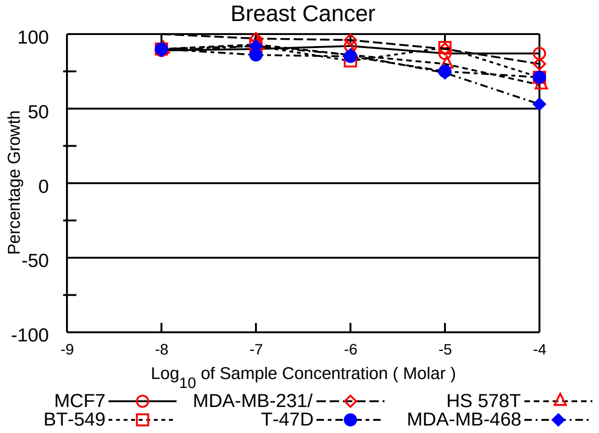

National Cancer Institute Developmental Therapeutics Program
Mean Graphs

NSC : 830098	Units :Molar	SSPL :0GZS	EXP. ID :2106NS48
(aR)-*iso*-6		Test Date :June 21, 2021	

Panel/Cell Line	Log_{10}GI50	Log_{10}TGI	Log_{10}LC50
Leukemia			
CCRF-CEM	> -4.00	> -4.00	> -4.00
HL-60(TB)	> -4.00	> -4.00	> -4.00
K-562	> -4.00	> -4.00	> -4.00
MOLT-4	> -4.00	> -4.00	> -4.00
RPMI-8226	> -4.00	> -4.00	> -4.00
SR	> -4.00	> -4.00	> -4.00
Non-Small Cell Lung Cancer			
A549/ATCC	> -4.00	> -4.00	> -4.00
EKVX	> -4.00	> -4.00	> -4.00
HOP-62	> -4.00	> -4.00	> -4.00
HOP-92	> -4.00	> -4.00	> -4.00
NCI-H226	> -4.00	> -4.00	> -4.00
NCI-H23	> -4.00	> -4.00	> -4.00
NCI-H322M	> -4.00	> -4.00	> -4.00
NCI-H460	> -4.00	> -4.00	> -4.00
NCI-H522	> -4.00	> -4.00	> -4.00
Colon Cancer			
COLO 205	> -4.00	> -4.00	> -4.00
HCC-2998	> -4.00	> -4.00	> -4.00
HCT-116	> -4.00	> -4.00	> -4.00
HCT-15	> -4.00	> -4.00	> -4.00
HT29	> -4.00	> -4.00	> -4.00
SW-620	> -4.00	> -4.00	> -4.00
CNS Cancer			
SF-295	> -4.00	> -4.00	> -4.00
SF-539	> -4.00	> -4.00	> -4.00
SNB-19	> -4.00	> -4.00	> -4.00
SNB-75	-4.40	> -4.00	> -4.00
U251	> -4.00	> -4.00	> -4.00
Melanoma			
LOX IMVI	> -4.00	> -4.00	> -4.00
MALME-3M	> -4.00	> -4.00	> -4.00
M14	> -4.00	> -4.00	> -4.00
MDA-MB-435	> -4.00	> -4.00	> -4.00
SK-MEL-2	> -4.00	> -4.00	> -4.00
SK-MEL-28	> -4.00	> -4.00	> -4.00
SK-MEL-5	> -4.00	> -4.00	> -4.00
UACC-257	> -4.00	> -4.00	> -4.00
UACC-62	> -4.00	> -4.00	> -4.00
Ovarian Cancer			
IGROV1	> -4.00	> -4.00	> -4.00
OVCAR-4	> -4.00	> -4.00	> -4.00
OVCAR-5	> -4.00	> -4.00	> -4.00
OVCAR-8	> -4.00	> -4.00	> -4.00
NCI/ADR-RES	> -4.00	> -4.00	> -4.00
SK-OV-3	> -4.00	> -4.00	> -4.00
Renal Cancer			
786-0	> -4.00	> -4.00	> -4.00
A498	> -4.00	> -4.00	> -4.00
ACHN	> -4.00	> -4.00	> -4.00
CAKI-1	> -4.00	> -4.00	> -4.00
RXF 393	> -4.00	> -4.00	> -4.00
SN12C	> -4.00	> -4.00	> -4.00
TK-10	> -4.00	> -4.00	> -4.00
UO-31	> -4.00	> -4.00	> -4.00
Prostate Cancer			
PC-3	> -4.00	> -4.00	> -4.00
Breast Cancer			
MCF7	> -4.00	> -4.00	> -4.00
MDA-MB-231/ATCC	> -4.00	> -4.00	> -4.00
HS 578T	> -4.00	> -4.00	> -4.00
BT-549	> -4.00	> -4.00	> -4.00
T-47D	> -4.00	> -4.00	> -4.00
MDA-MB-468	> -4.00	> -4.00	> -4.00
_MID	-4.01	-4.0	-4.0
Delta	0.39	0	0
Range	0.4	0.0	0.0

GI50

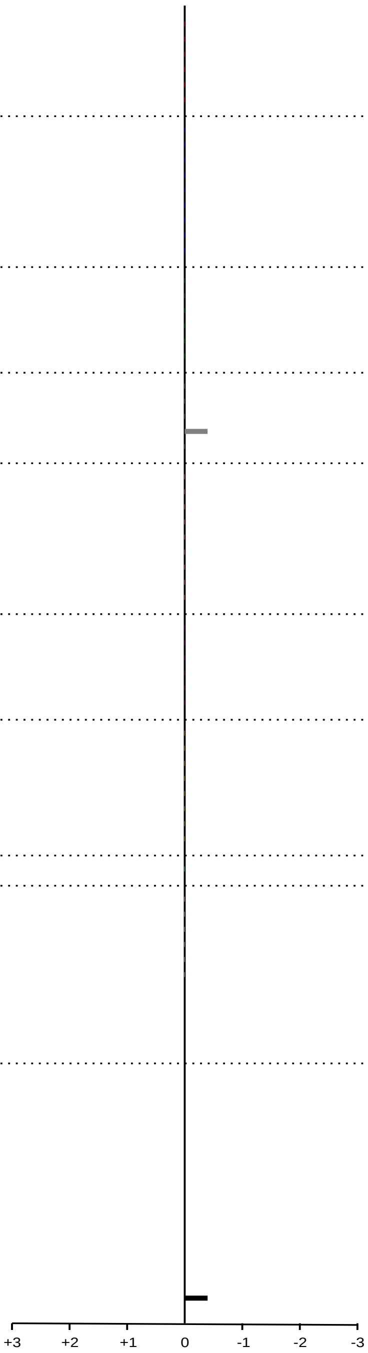

TGI

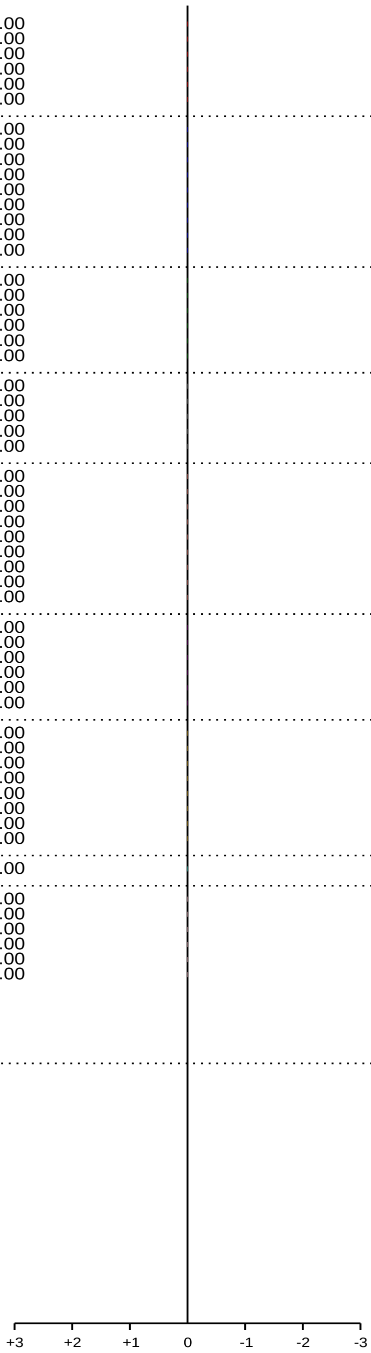

LC50

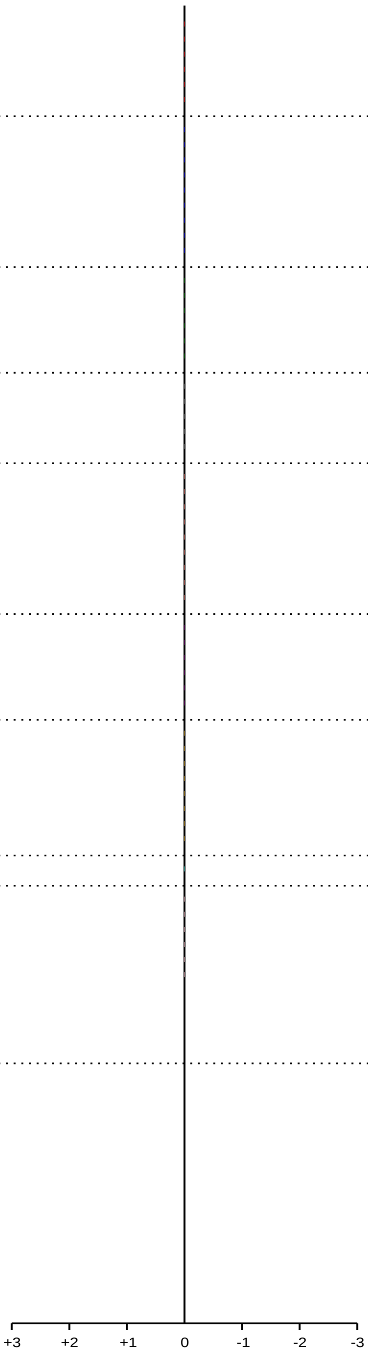

National Cancer Institute Developmental Therapeutics Program
Averaged Mean Graph - NSC: S757

Colchicine | Test Type:08 | HiConc:1.00E-4 | Unit:Molar

Exp. Id: 9508NS32 9212EC88 9102BG50 9102BS04 9103BG63 ... 7 separate experiments

Panel/Cell Line	Log_{10}GI50	Log_{10}TGI	Log_{10}LC50
**Leukemia**			
CCRF-CEM	-7.88	-4.00	-4.00
HL-60(TB)	-7.89	-7.55	-4.00
K-562	-7.87	-4.00	-4.00
MOLT-4	-7.62	-4.32	-4.00
RPMI-8226	-8.00	-4.00	-4.00
SR	-7.66	-4.87	-4.00
**Non-Small Cell Lung Cancer**			
A549/ATCC	-7.23	-4.00	-4.00
EKVX	-4.31	-4.00	-4.00
HOP-18	-4.16	-4.00	-4.00
HOP-62	-7.59	-4.00	-4.00
HOP-92	-7.93	-6.31	-4.00
NCI-H226	-7.00	-5.35	-4.00
NCI-H23	-7.79	-5.16	-4.00
NCI-H322M	-7.61	-4.81	-4.00
NCI-H460	-7.69	-5.86	-4.00
NCI-H522	-7.92	-7.59	-4.20
LXFL 529	-7.95	-4.00	-4.00
**Small Cell Lung Cancer**			
DMS 114	-7.35	-7.14	-4.00
DMS 273	-7.85	-4.00	-4.00
**Colon Cancer**			
COLO 205	-7.59	-7.46	-6.94
DLD-1	-7.76	-4.00	-4.00
HCC-2998	-7.76	-5.99	-4.00
HCT-116	-7.77	-4.74	-4.00
HCT-15	-6.91	-4.59	-4.00
HT29	-7.72	-5.98	-4.00
KM12	-7.84	-6.42	-4.00
KM20L2	-7.87	-7.63	-6.10
SW-620	-7.85	-4.09	-4.00
**CNS Cancer**			
SF-268	-7.19	-4.34	-4.00
SF-295	-7.84	-7.45	-4.10
SF-539	-7.84	-6.86	-4.01
SNB-19	-7.55	-4.08	-4.00
SNB-75	-7.59	-4.92	-4.00
SNB-78	-7.97	-4.00	-4.00
U251	-7.62	-5.14	-4.39
XF 498	-7.73	-4.89	-4.00
**Melanoma**			
LOX IMVI	-7.76	-4.41	-4.05
MALME-3M	-7.72	-4.06	-4.00
M14	-7.76	-4.50	-4.00
MDA-MB-435	-8.00	-7.75	-6.00
MDA-N	-8.00	-6.08	-4.00
M19-MEL	-7.68	-4.00	-4.00
SK-MEL-2	-7.93	-6.30	-4.00
SK-MEL-28	-7.25	-4.00	-4.00
SK-MEL-5	-7.94	-7.09	-4.33
UACC-257	-5.00	-4.00	-4.00
UACC-62	-7.61	-4.11	-4.03
**Ovarian Cancer**			
IGROV1	-7.61	-4.00	-4.00
OVCAR-3	-7.82	-7.58	-4.00
OVCAR-4	-4.89	-4.00	-4.00
OVCAR-5	-5.14	-4.07	-4.00
OVCAR-8	-7.68	-4.21	-4.00
NCI/ADR-RES	-6.31	-5.27	-4.00
SK-OV-3	-7.12	-5.09	-4.00
**Renal Cancer**			
786-0	-7.49	-4.00	-4.00
A498	-7.51	-4.93	-4.00
ACHN	-6.70	-4.00	-4.00
CAKI-1	-7.00	-4.45	-4.00
RXF 393	-7.92	-5.15	-4.17
RXF-631	-7.44	-4.04	-4.00
SN12C	-7.38	-4.03	-4.00
TK-10	-4.63	-4.00	-4.00
UO-31	-6.25	-4.03	-4.00
**Prostate Cancer**			
PC-3	-7.63	-5.18	-4.00
DU-145	-6.18	-4.92	-4.00
**Breast Cancer**			
MCF7	-7.94	-4.00	-4.00
MDA-MB-231/ATCC	-6.46	-4.00	-4.00
HS 578T			-4.00
BT-549	-7.69	-4.15	-4.00
T-47D	-4.11	-4.00	-4.00
MG_MID	-7.26	-4.97	-4.12
Delta	0.74	2.78	2.82
Range	3.89	3.75	2.94

Axis scale (GI50, TGI, LC50): +3 +2 +1 0 -1 -2 -3

National Cancer Institute Developmental Therapeutics Program
Dose Response Curves

NSC: 757 / 21

SSPL: L19P

EXP. ID: 9007SR18

Test Date: July 23, 1990

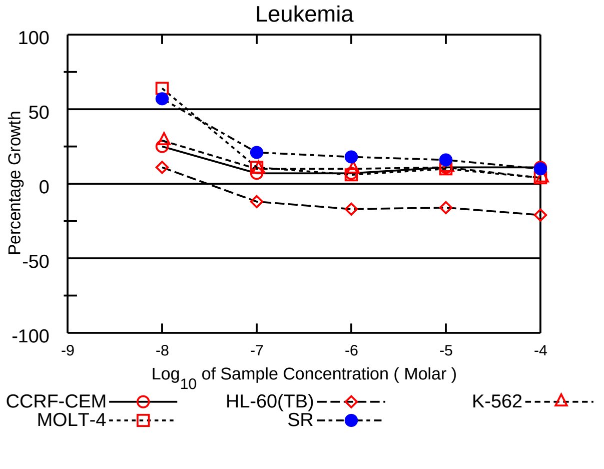

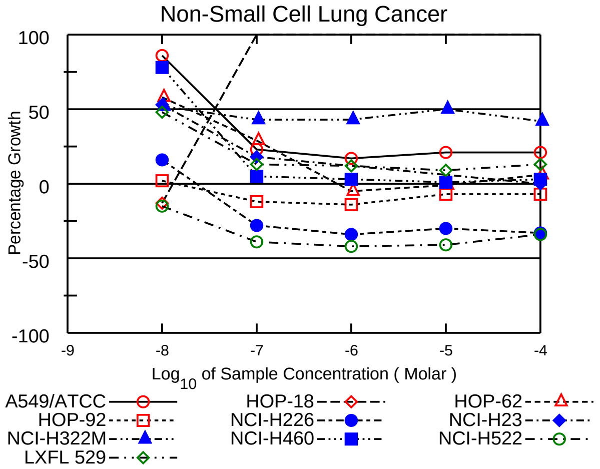

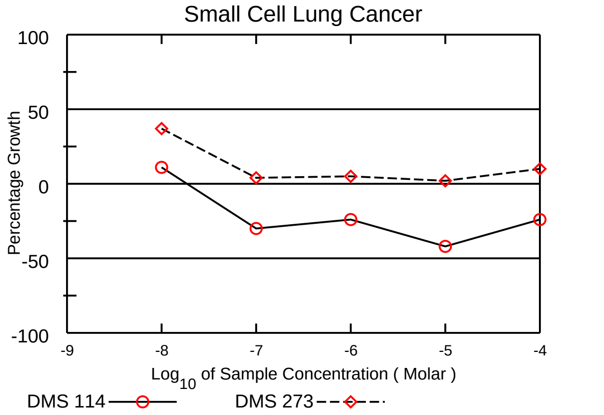

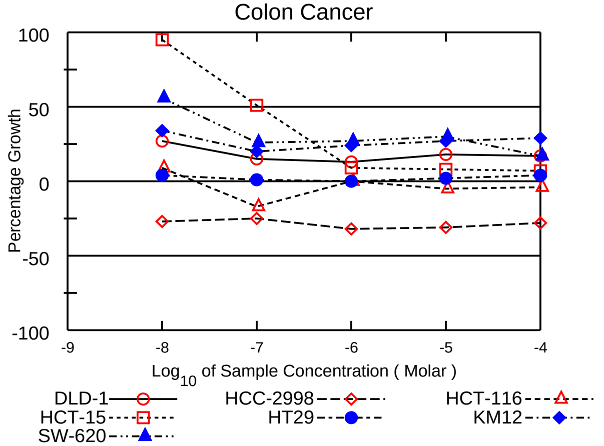

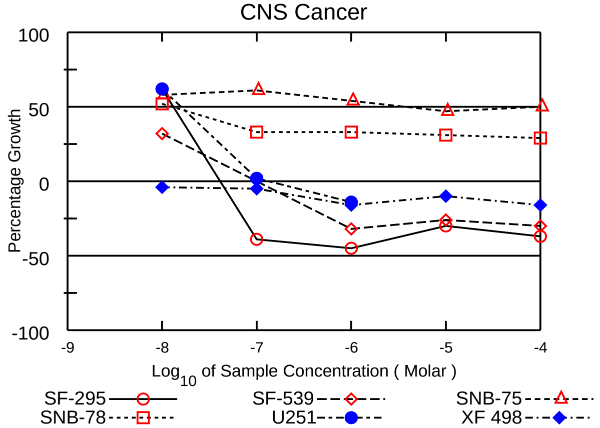

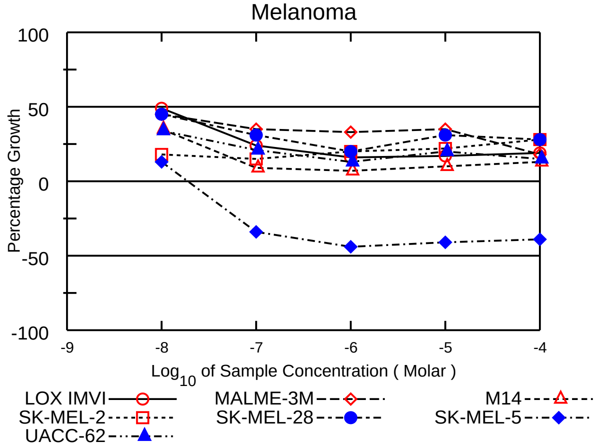

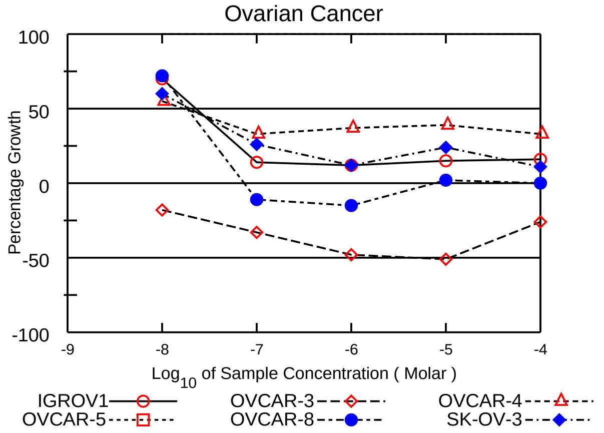

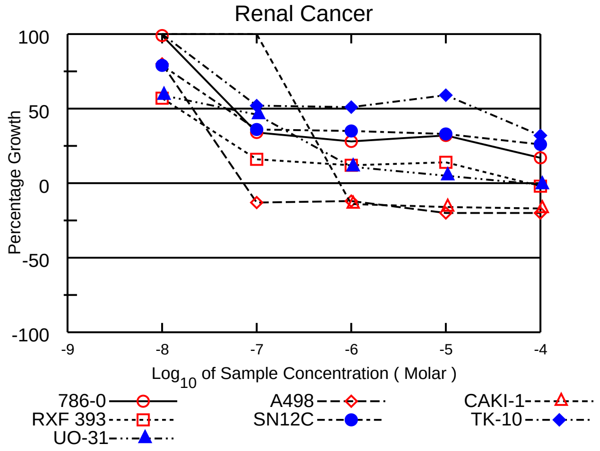

National Cancer Institute Developmental Therapeutics Program
Mean Graphs

NSC :757/21 | Units :Molar | SSPL :L19P | EXP. ID :9007SR18

Test Date :July 23, 1990

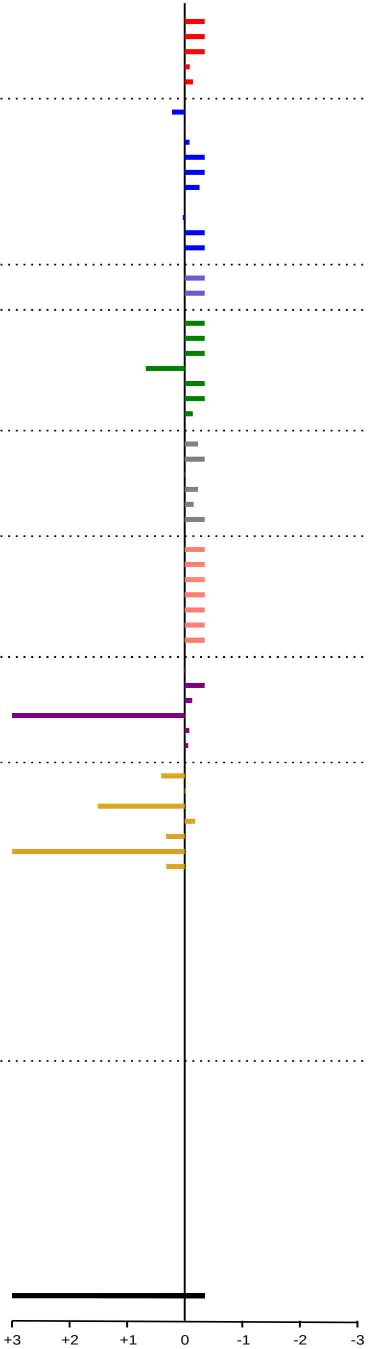

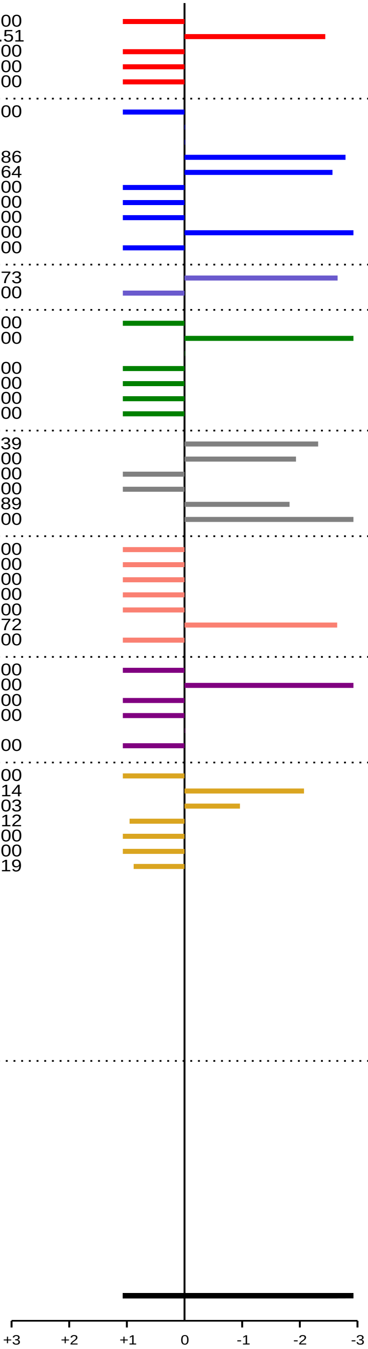

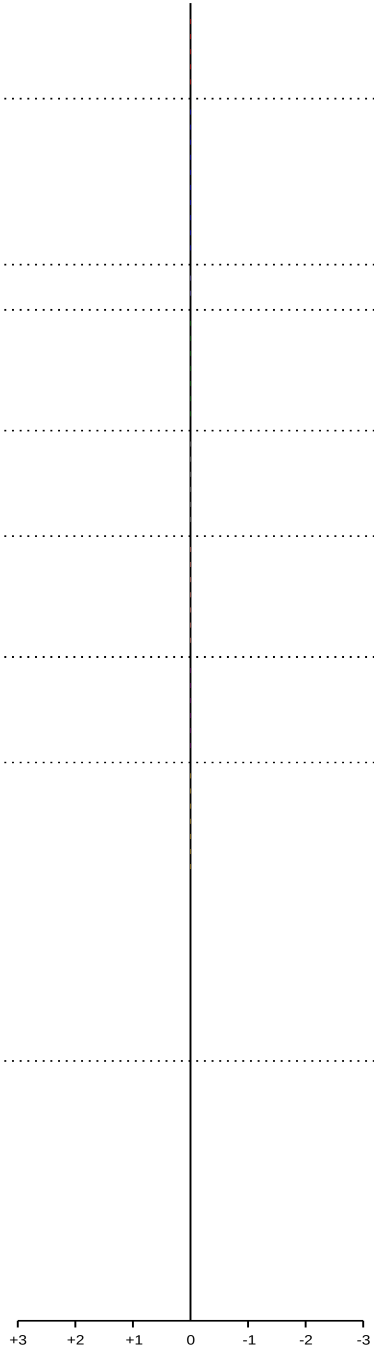

Panel/Cell Line	Log_{10}GI50	Log_{10}TGI	Log_{10}LC50
**Leukemia**			
CCRF-CEM	< -8.00	> -4.00	> -4.00
HL-60(TB)	< -8.00	-7.51	> -4.00
K-562	< -8.00	> -4.00	> -4.00
MOLT-4	-7.74	> -4.00	> -4.00
SR	-7.80	> -4.00	> -4.00
**Non-Small Cell Lung Cancer**			
A549/ATCC	-7.43	> -4.00	> -4.00
HOP-18			> -4.00
HOP-62	-7.74		> -4.00
HOP-92	< -8.00	-7.86	> -4.00
NCI-H226	< -8.00	-7.64	> -4.00
NCI-H23	-7.91	> -4.00	> -4.00
NCI-H322M		> -4.00	> -4.00
NCI-H460	-7.62	> -4.00	> -4.00
NCI-H522	< -8.00	< -8.00	> -4.00
LXFL 529	< -8.00	> -4.00	> -4.00
**Small Cell Lung Cancer**			
DMS 114	< -8.00	-7.73	> -4.00
DMS 273	< -8.00	> -4.00	> -4.00
**Colon Cancer**			
DLD-1	< -8.00	> -4.00	> -4.00
HCC-2998	< -8.00	< -8.00	> -4.00
HCT-116	< -8.00		> -4.00
HCT-15	-6.98	> -4.00	> -4.00
HT29	< -8.00	> -4.00	> -4.00
KM12	< -8.00	> -4.00	> -4.00
SW-620	-7.79	> -4.00	> -4.00
**CNS Cancer**			
SF-295	-7.88	-7.39	> -4.00
SF-539	< -8.00	-7.00	> -4.00
SNB-75		> -4.00	> -4.00
SNB-78	-7.88	> -4.00	> -4.00
U251	-7.81	-6.89	
XF 498	< -8.00	< -8.00	> -4.00
**Melanoma**			
LOX IMVI	< -8.00	> -4.00	> -4.00
MALME-3M	< -8.00	> -4.00	> -4.00
M14	< -8.00	> -4.00	> -4.00
SK-MEL-2	< -8.00	> -4.00	> -4.00
SK-MEL-28	< -8.00	> -4.00	> -4.00
SK-MEL-5	< -8.00	-7.72	> -4.00
UACC-62	< -8.00	> -4.00	> -4.00
**Ovarian Cancer**			
IGROV1	-7.64	> -4.00	> -4.00
OVCAR-3	< -8.00	< -8.00	
OVCAR-4	-7.78	> -4.00	> -4.00
OVCAR-5	> -4.00	> -4.00	> -4.00
OVCAR-8	-7.73		> -4.00
SK-OV-3	-7.71	> -4.00	> -4.00
**Renal Cancer**			
786-0	-7.24	> -4.00	> -4.00
A498	-7.68	-7.14	> -4.00
CAKI-1	-6.14	-6.03	> -4.00
RXF 393	-7.83	-4.12	> -4.00
SN12C	-7.33	> -4.00	> -4.00
TK-10	-4.66	> -4.00	> -4.00
UO-31	-7.33	-4.19	> -4.00
_MID	-7.65	-5.07	-4.0
Delta	0.35	2.93	0
Range	4.0	4.0	0.0

**National Cancer Institute Developmental Therapeutics Program Dose Response Curves**	NSC: 757 / 21	SSPL: L19P	EXP. ID: 9102BS04
		Test Date: February 04, 1991	

Leukemia

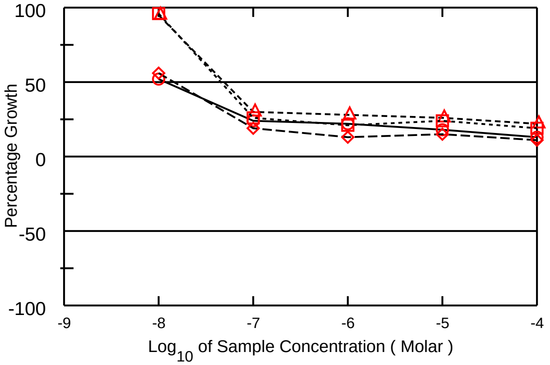

CCRF-CEM, K-562, MOLT-4, SR

Non-Small Cell Lung Cancer

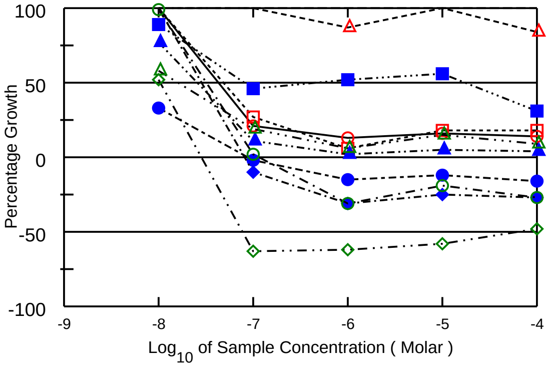

A549/ATCC, EKVX, HOP-18, HOP-62, HOP-92, NCI-H226, NCI-H23, NCI-H322M, NCI-H460, NCI-H522, LXFL 529

Small Cell Lung Cancer

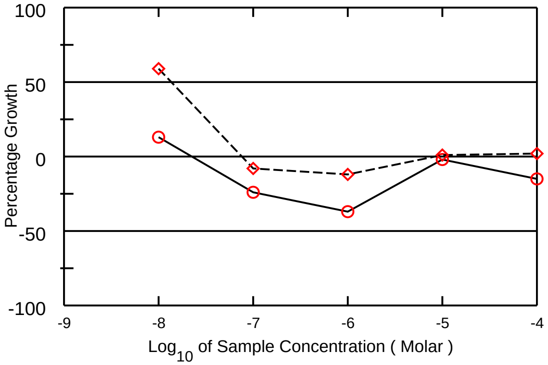

DMS 114, DMS 273

Colon Cancer

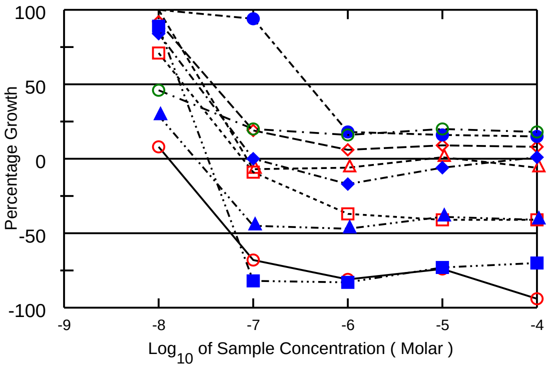

COLO 205, DLD-1, HCC-2998, HCT-116, HCT-15, HT29, KM12, KM20L2, SW-620

CNS Cancer

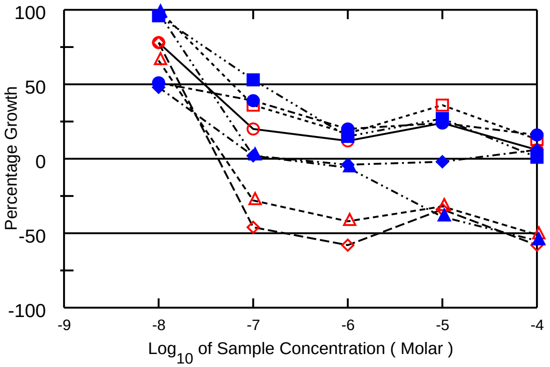

SF-268, SF-295, SF-539, SNB-19, SNB-75, SNB-78, U251, XF 498

Melanoma

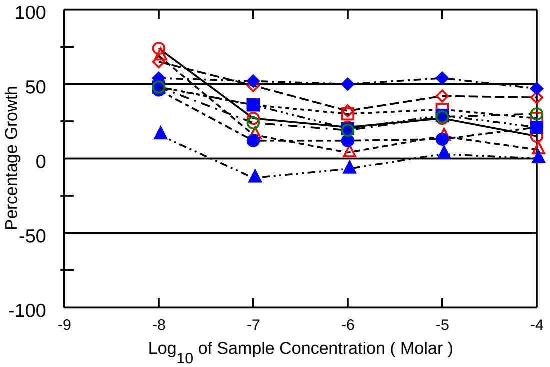

LOX IMVI, MALME-3M, M14, M19-MEL, SK-MEL-2, SK-MEL-28, SK-MEL-5, UACC-257, UACC-62

Ovarian Cancer

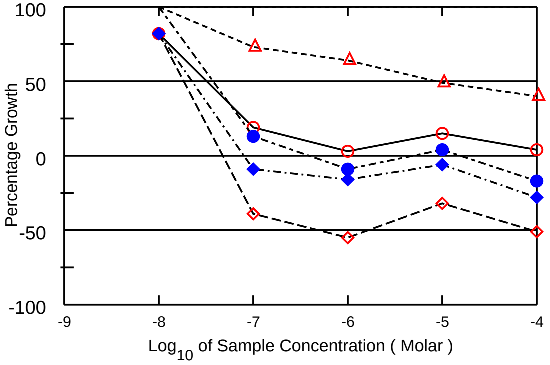

IGROV1, OVCAR-3, OVCAR-4, OVCAR-5, OVCAR-8, SK-OV-3

Renal Cancer

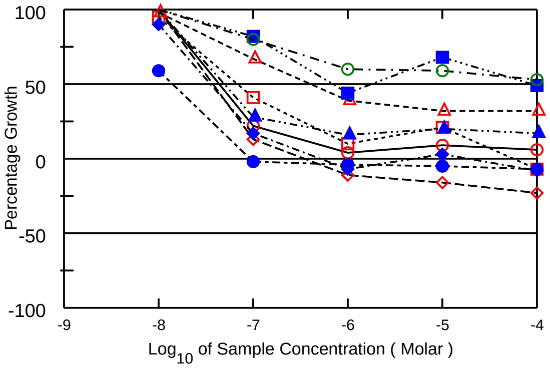

786-0, A498, ACHN, CAKI-1, RXF 393, RXF-631, SN12C, TK-10, UO-31

National Cancer Institute Developmental Therapeutics Program

Mean Graphs

NSC :757/21 | Units :Molar | SSPL :L19P | EXP. ID :9102BS04

Test Date :February 04, 1991

Panel/Cell Line	Log_{10}GI50	Log_{10}TGI	Log_{10}LC50
**Leukemia**			
CCRF-CEM	-7.93	> -4.00	> -4.00
K-562	-7.84	> -4.00	> -4.00
MOLT-4	-7.32	> -4.00	> -4.00
SR	-7.34	> -4.00	> -4.00
**Non-Small Cell Lung Cancer**			
A549/ATCC	-7.37	> -4.00	> -4.00
EKVX	> -4.00	> -4.00	> -4.00
HOP-18	> -4.00	> -4.00	> -4.00
HOP-62	-7.31	> -4.00	> -4.00
HOP-92	< -8.00	-7.06	> -4.00
NCI-H226	-7.52	-7.09	> -4.00
NCI-H23	-7.59	> -4.00	> -4.00
NCI-H322M		> -4.00	> -4.00
NCI-H460	-7.50	-6.95	> -4.00
NCI-H522	-7.98	-7.55	
LXFL 529	-7.80	> -4.00	> -4.00
**Small Cell Lung Cancer**			
DMS 114	< -8.00	-7.65	> -4.00
DMS 273	-7.87		> -4.00
**Colon Cancer**			
COLO 205	< -8.00	-7.89	-7.24
DLD-1	-7.43	> -4.00	> -4.00
HCC-2998	-7.52		> -4.00
HCT-116	-7.73	-7.11	> -4.00
HCT-15	-6.42	> -4.00	> -4.00
HT29	-7.59		> -4.00
KM12	< -8.00	-7.61	> -4.00
KM20L2	-7.77	-7.48	-7.19
SW-620	< -8.00	> -4.00	> -4.00
**CNS Cancer**			
SF-268	-7.52	> -4.00	> -4.00
SF-295	-7.77	-7.37	
SF-539	-7.83	-7.30	-4.05
SNB-19	-7.22	> -4.00	> -4.00
SNB-75	-7.90	> -4.00	> -4.00
SNB-78	< -8.00		> -4.00
U251	-7.50	-6.80	-4.31
XF 498	-6.93	> -4.00	> -4.00
**Melanoma**			
LOX IMVI	-7.49	> -4.00	> -4.00
MALME-3M	-7.04	> -4.00	> -4.00
M14	-7.64	> -4.00	> -4.00
M19-MEL	< -8.00	> -4.00	> -4.00
SK-MEL-2	< -8.00	> -4.00	> -4.00
SK-MEL-28		> -4.00	> -4.00
SK-MEL-5	< -8.00		> -4.00
UACC-257	< -8.00	> -4.00	> -4.00
UACC-62	< -8.00	> -4.00	> -4.00
**Ovarian Cancer**			
IGROV1	-7.49	> -4.00	> -4.00
OVCAR-3	-7.73	-7.32	
OVCAR-4	-5.08	> -4.00	> -4.00
OVCAR-5	> -4.00	> -4.00	> -4.00
OVCAR-8	-7.42		> -4.00
SK-OV-3	-7.65	-7.10	> -4.00
**Renal Cancer**			
786-0	-7.34	> -4.00	> -4.00
A498	-7.40	-6.47	> -4.00
ACHN	-6.40	> -4.00	> -4.00
CAKI-1	-7.16	-4.24	> -4.00
RXF 393	-7.85	-7.04	> -4.00
RXF-631	-7.45		> -4.00
SN12C	-7.28	> -4.00	> -4.00
TK-10		> -4.00	> -4.00
UO-31	> -4.00	> -4.00	> -4.00
_MID	-7.29	-5.02	-4.12
Delta	0.71	2.87	3.12
Range	4.0	3.89	3.24

GI50

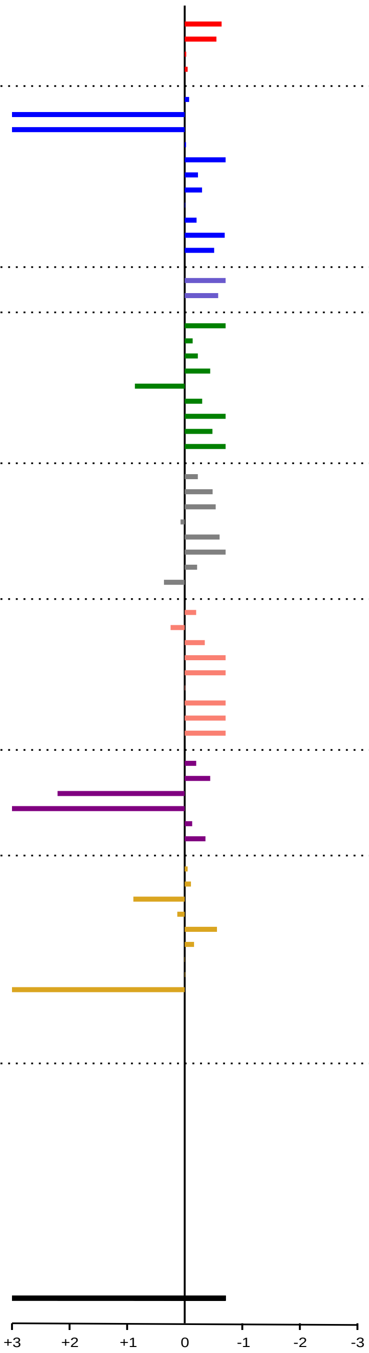

TGI

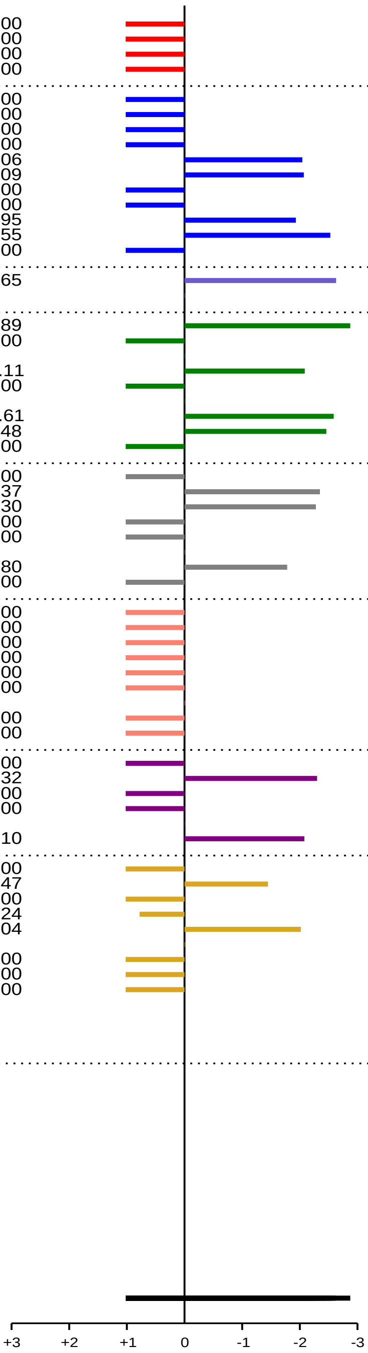

LC50

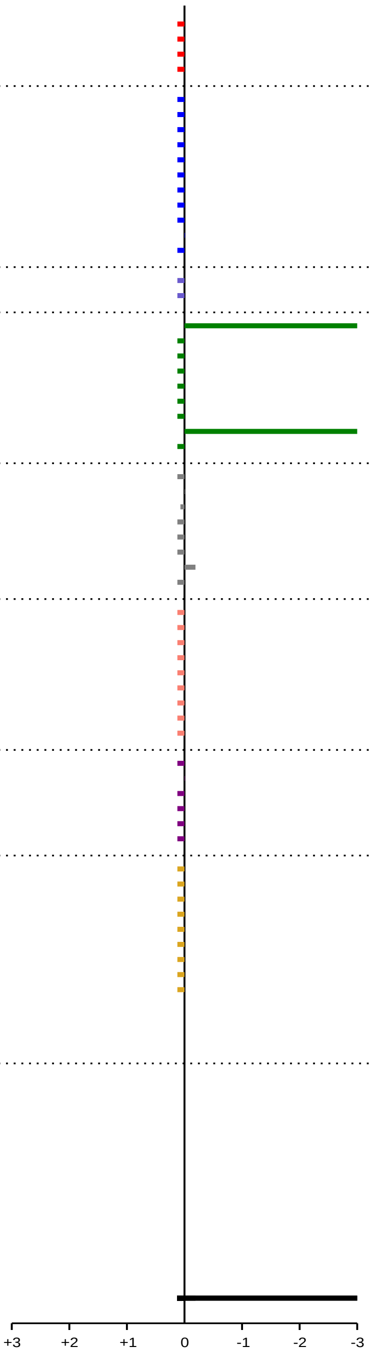

National Cancer Institute Developmental Therapeutics Program Dose Response Curves	NSC: 757 / 1	SSPL:	EXP. ID: 9103BG63
		Test Date: March 25, 1991	

Leukemia

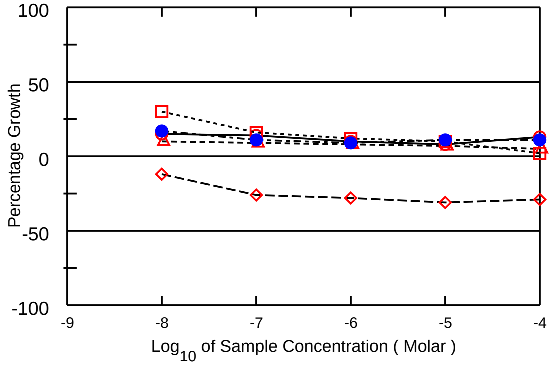

CCRF-CEM · HL-60(TB) · K-562 · MOLT-4 · RPMI-8226

Non-Small Cell Lung Cancer

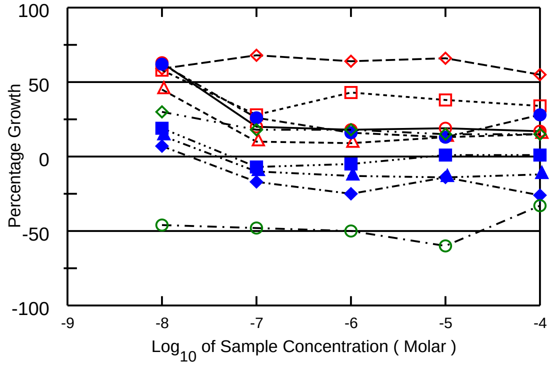

A549/ATCC · EKVX · HOP-62 · HOP-92 · NCI-H226 · NCI-H23 · NCI-H322M · NCI-H460 · NCI-H522 · LXFL 529

Small Cell Lung Cancer

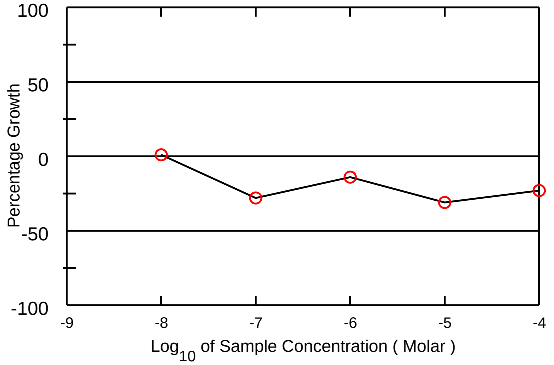

DMS 114

Colon Cancer

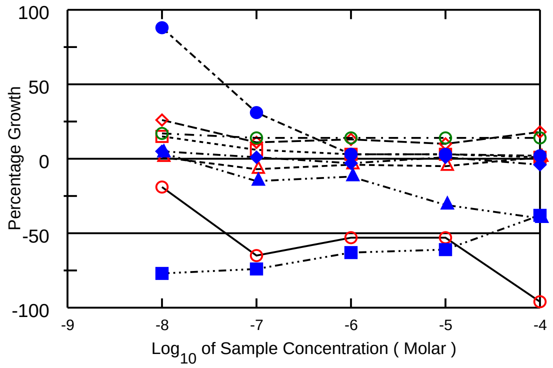

COLO 205 · DLD-1 · HCC-2998 · HCT-116 · HCT-15 · HT29 · KM12 · KM20L2 · SW-620

CNS Cancer

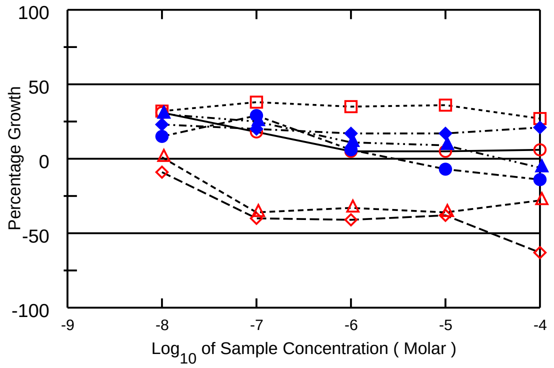

SF-268 · SF-295 · SF-539 · SNB-19 · SNB-75 · SNB-78 · XF 498

Melanoma

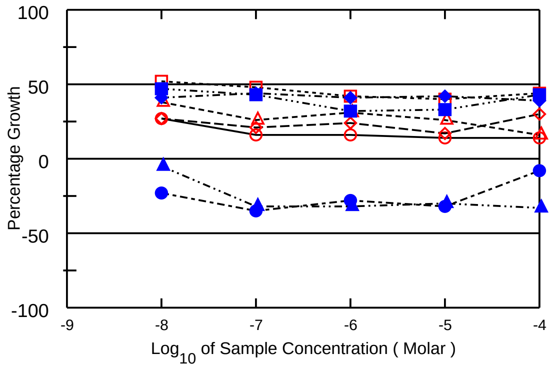

LOX IMVI · MALME-3M · M14 · M19-MEL · SK-MEL-2 · SK-MEL-28 · SK-MEL-5 · UACC-62

Ovarian Cancer

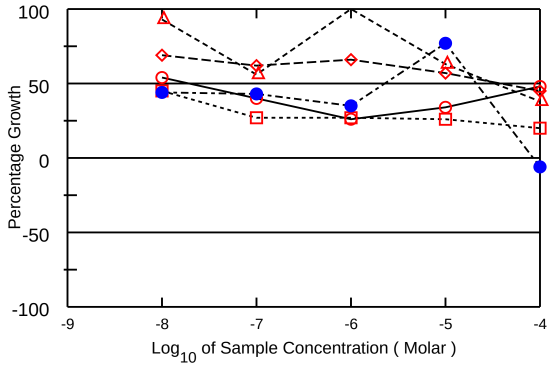

IGROV1 · OVCAR-4 · OVCAR-5 · OVCAR-8 · SK-OV-3

Renal Cancer

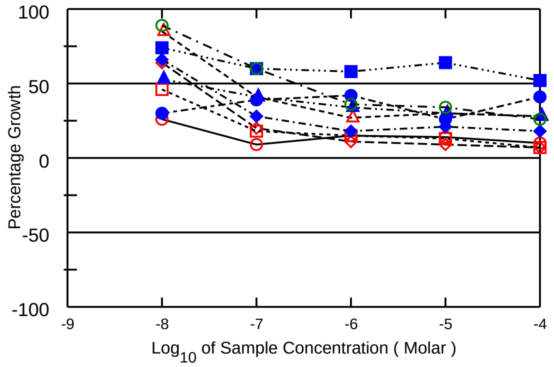

786-0 · A498 · ACHN · CAKI-1 · RXF 393 · RXF-631 · SN12C · TK-10 · UO-31

National Cancer Institute Developmental Therapeutics Program Mean Graphs	NSC :757/1	Units :Molar	SSPL :	EXP. ID :9103BG63
			Test Date :March 25, 1991	

Panel/Cell Line	Log_{10}GI50	Log_{10}TGI	Log_{10}LC50
**Leukemia**			
CCRF-CEM	< -8.00	> -4.00	> -4.00
HL-60(TB)	< -8.00	< -8.00	> -4.00
K-562	< -8.00	> -4.00	> -4.00
MOLT-4	< -8.00	> -4.00	> -4.00
RPMI-8226	< -8.00	> -4.00	> -4.00
**Non-Small Cell Lung Cancer**			
A549/ATCC	-7.70	> -4.00	> -4.00
EKVX	> -4.00	> -4.00	> -4.00
HOP-62	< -8.00	> -4.00	> -4.00
HOP-92	-7.73	> -4.00	> -4.00
NCI-H226	-7.67	> -4.00	> -4.00
NCI-H23	< -8.00	-7.72	> -4.00
NCI-H322M	< -8.00	-7.40	> -4.00
NCI-H460	< -8.00		> -4.00
NCI-H522	< -8.00	< -8.00	
LXFL 529	< -8.00	> -4.00	> -4.00
**Small Cell Lung Cancer**			
DMS 114	< -8.00	-7.98	> -4.00
**Colon Cancer**			
COLO 205	< -8.00	< -8.00	-7.33
DLD-1	< -8.00	> -4.00	> -4.00
HCC-2998	< -8.00		> -4.00
HCT-116	< -8.00	> -4.00	> -4.00
HCT-15	-7.34	> -4.00	> -4.00
HT29	< -8.00		> -4.00
KM12	< -8.00	-7.80	> -4.00
KM20L2	< -8.00	< -8.00	
SW-620	< -8.00	> -4.00	> -4.00
**CNS Cancer**			
SF-268	< -8.00	> -4.00	> -4.00
SF-295	< -8.00	< -8.00	-4.51
SF-539	< -8.00	-7.98	> -4.00
SNB-19	< -8.00	> -4.00	> -4.00
SNB-75	< -8.00	-5.53	> -4.00
SNB-78	< -8.00	> -4.00	> -4.00
XF 498	< -8.00	-4.41	> -4.00
**Melanoma**			
LOX IMVI	< -8.00	> -4.00	> -4.00
MALME-3M	< -8.00	> -4.00	> -4.00
M14	< -8.00	> -4.00	> -4.00
M19-MEL	-7.57	> -4.00	> -4.00
SK-MEL-2	< -8.00	< -8.00	> -4.00
SK-MEL-28	< -8.00	> -4.00	> -4.00
SK-MEL-5	< -8.00	< -8.00	> -4.00
UACC-62	< -8.00	> -4.00	> -4.00
**Ovarian Cancer**			
IGROV1	-7.72	> -4.00	> -4.00
OVCAR-4	-4.45	> -4.00	> -4.00
OVCAR-5	-4.48	> -4.00	> -4.00
OVCAR-8	< -8.00	> -4.00	> -4.00
SK-OV-3		-4.07	> -4.00
**Renal Cancer**			
786-0	< -8.00	> -4.00	> -4.00
A498	-7.68	> -4.00	> -4.00
ACHN	-7.20	> -4.00	> -4.00
CAKI-1	< -8.00	> -4.00	> -4.00
RXF 393	< -8.00	> -4.00	> -4.00
RXF-631	-7.58	> -4.00	> -4.00
SN12C	-7.74	> -4.00	> -4.00
TK-10	> -4.00	> -4.00	> -4.00
UO-31	-6.57	> -4.00	> -4.00

	GI50	TGI	LC50
_MID	-7.61	-4.96	-4.07
Delta	0.39	3.04	3.26
Range	4.0	4.0	3.33

GI50
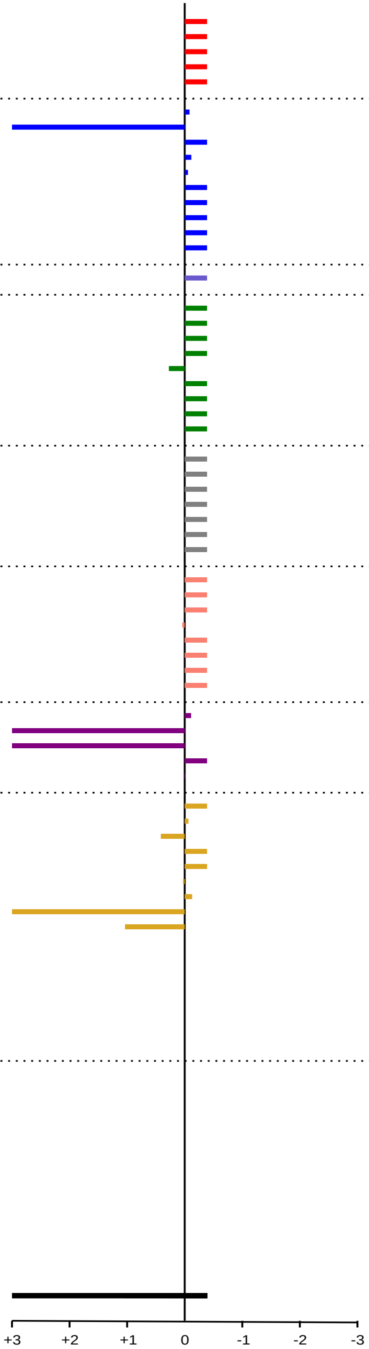

TGI
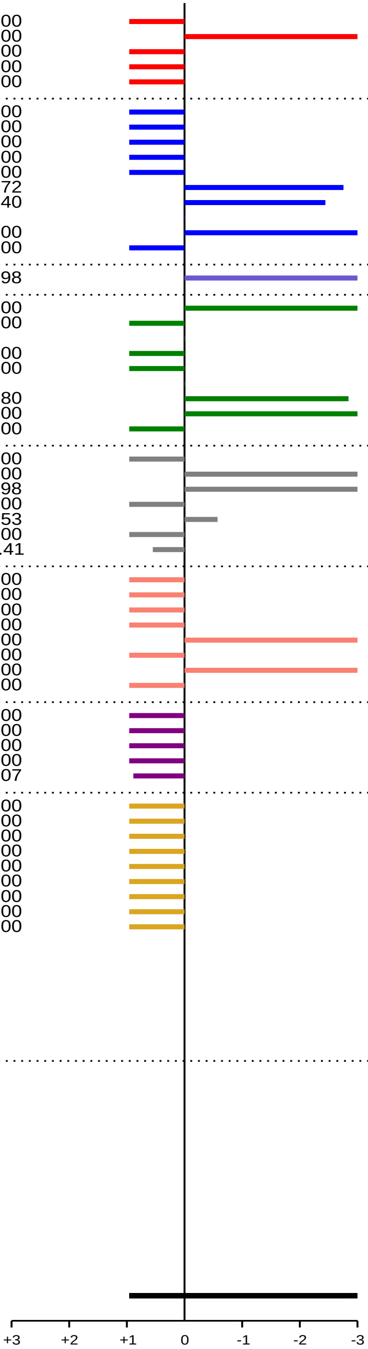

LC50
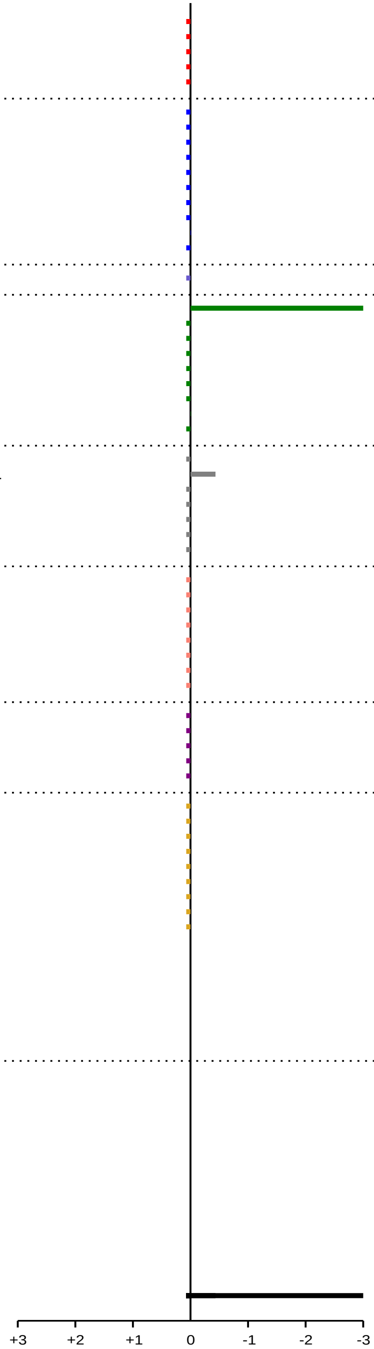

National Cancer Institute Developmental Therapeutics Program Dose Response Curves	NSC: 757 / 1	SSPL:	EXP. ID: 9103BG64
		Test Date: March 26, 1991	

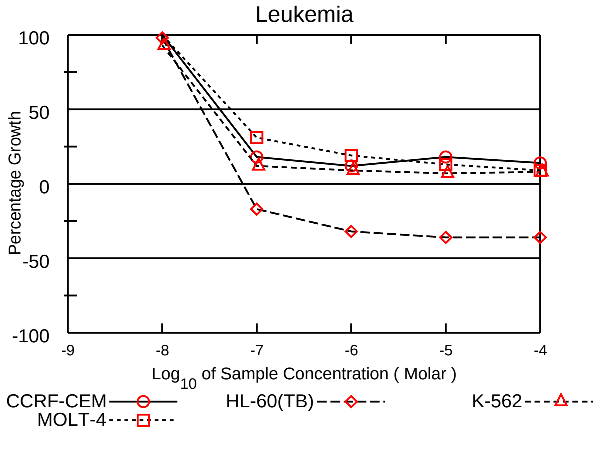

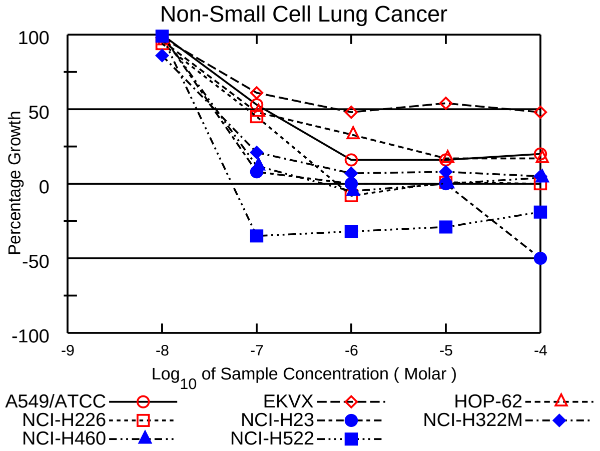

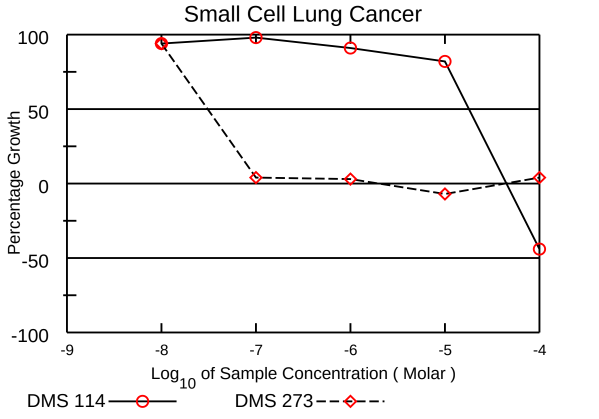

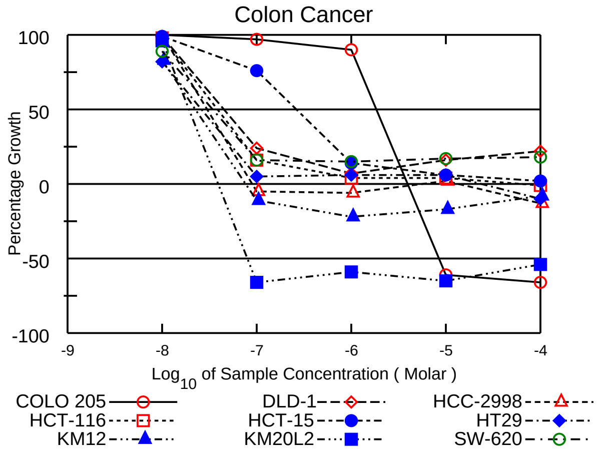

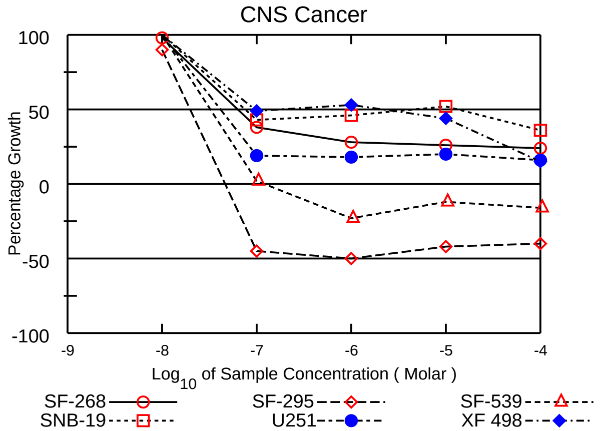

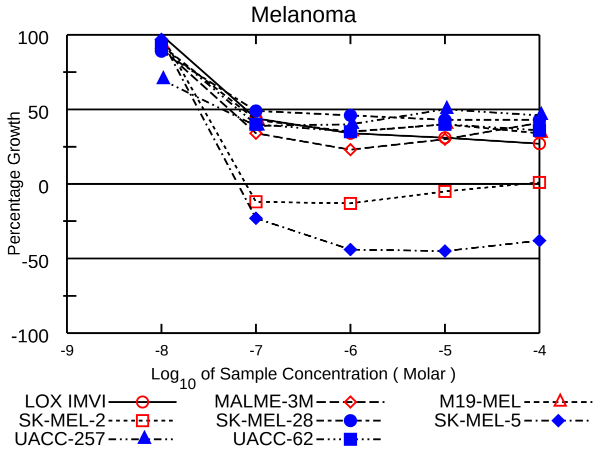

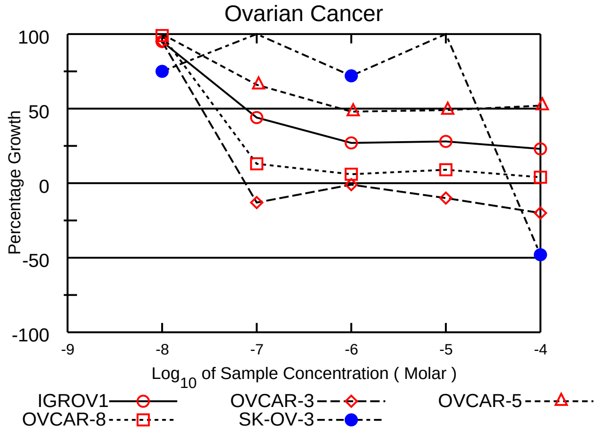

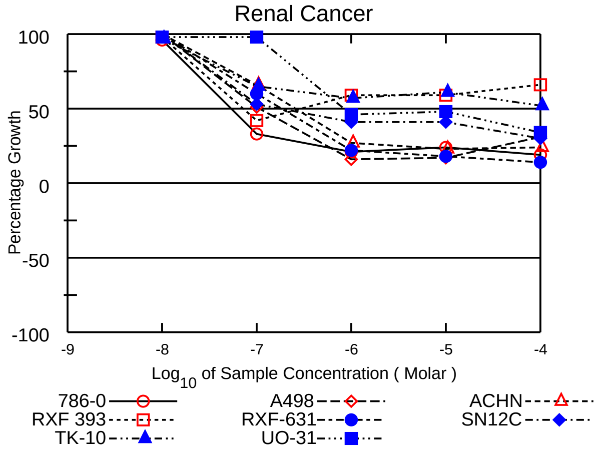

National Cancer Institute Developmental Therapeutics Program Mean Graphs	NSC :757/1	Units :Molar	SSPL :	EXP. ID :9103BG64
			Test Date :March 26, 1991	

Panel/Cell Line	Log_{10}GI50	Log_{10}TGI	Log_{10}LC50
**Leukemia**			
CCRF-CEM	-7.39	> -4.00	> -4.00
HL-60(TB)	-7.58	-7.15	> -4.00
K-562	-7.47	> -4.00	> -4.00
MOLT-4	-7.27	> -4.00	> -4.00
**Non-Small Cell Lung Cancer**			
A549/ATCC	-6.93	> -4.00	> -4.00
EKVX		> -4.00	> -4.00
HOP-62	-7.05	> -4.00	> -4.00
NCI-H226	-7.10		> -4.00
NCI-H23	-7.45	-5.00	> -4.00
NCI-H322M	-7.45	> -4.00	> -4.00
NCI-H460	-7.42		> -4.00
NCI-H522	-7.64	-7.26	> -4.00
**Small Cell Lung Cancer**			
DMS 114	-4.75	-4.35	> -4.00
DMS 273	-7.51		> -4.00
**Colon Cancer**			
COLO 205	-5.74	-5.41	-5.07
DLD-1	-7.35	> -4.00	> -4.00
HCC-2998	-7.48		> -4.00
HCT-116	-7.41	-4.23	> -4.00
HCT-15	-6.58	> -4.00	> -4.00
HT29	-7.59	-4.61	> -4.00
KM12	-7.65	-7.12	> -4.00
KM20L2	-7.72	-7.41	-7.10
SW-620	-7.46	> -4.00	> -4.00
**CNS Cancer**			
SF-268	-7.19	> -4.00	> -4.00
SF-295	-7.70	-7.33	> -4.00
SF-539	-7.48	-6.94	> -4.00
SNB-19		> -4.00	> -4.00
U251	-7.38	> -4.00	> -4.00
XF 498		> -4.00	> -4.00
**Melanoma**			
LOX IMVI	-7.11	> -4.00	> -4.00
MALME-3M	-7.28	> -4.00	> -4.00
M19-MEL	-7.14	> -4.00	> -4.00
SK-MEL-2	-7.53		> -4.00
SK-MEL-28	-7.02	> -4.00	> -4.00
SK-MEL-5	-7.61	-7.19	> -4.00
UACC-257		> -4.00	> -4.00
UACC-62	-7.19	> -4.00	> -4.00
**Ovarian Cancer**			
IGROV1	-7.11	> -4.00	> -4.00
OVCAR-3	-7.58	-7.12	> -4.00
OVCAR-5		> -4.00	> -4.00
OVCAR-8	-7.43	> -4.00	> -4.00
SK-OV-3	-4.43	-4.21	> -4.00
**Renal Cancer**			
786-0	-7.27	> -4.00	> -4.00
A498	-6.96	> -4.00	> -4.00
ACHN	-6.58	> -4.00	> -4.00
RXF 393		> -4.00	> -4.00
RXF-631	-6.73	> -4.00	> -4.00
SN12C	-6.78	> -4.00	> -4.00
TK-10	> -4.00	> -4.00	> -4.00
UO-31	-6.08	> -4.00	> -4.00
_MID	-7.04	-4.65	-4.08
Delta	0.68	2.76	3.02
Range	3.72	3.41	3.1

GI50

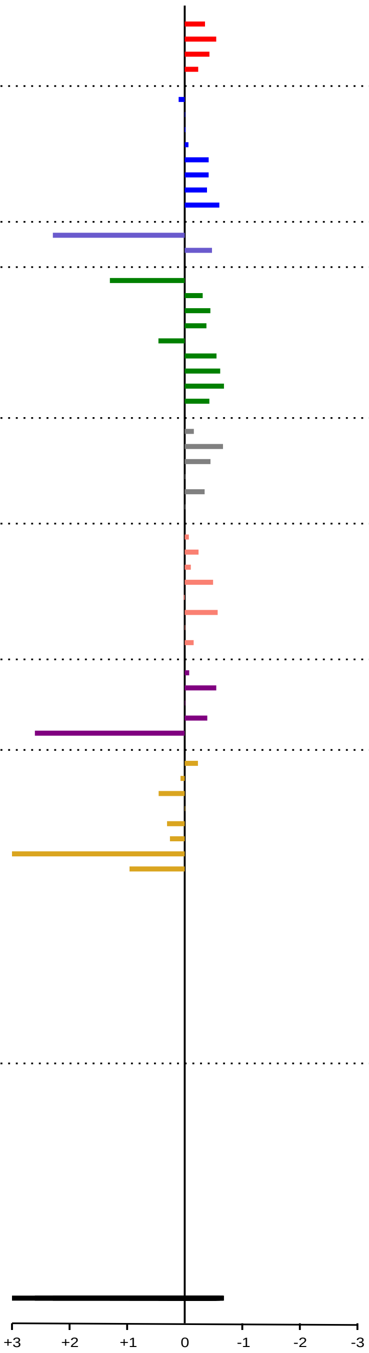

TGI

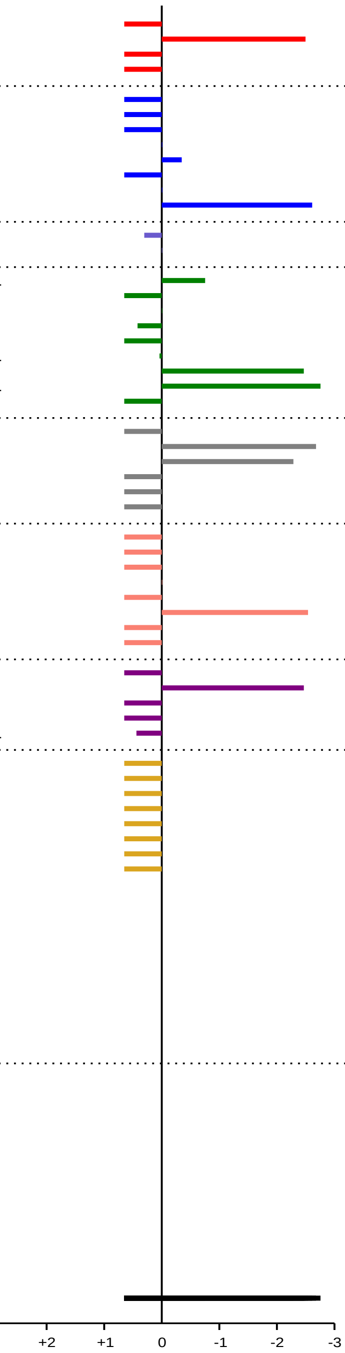

LC50

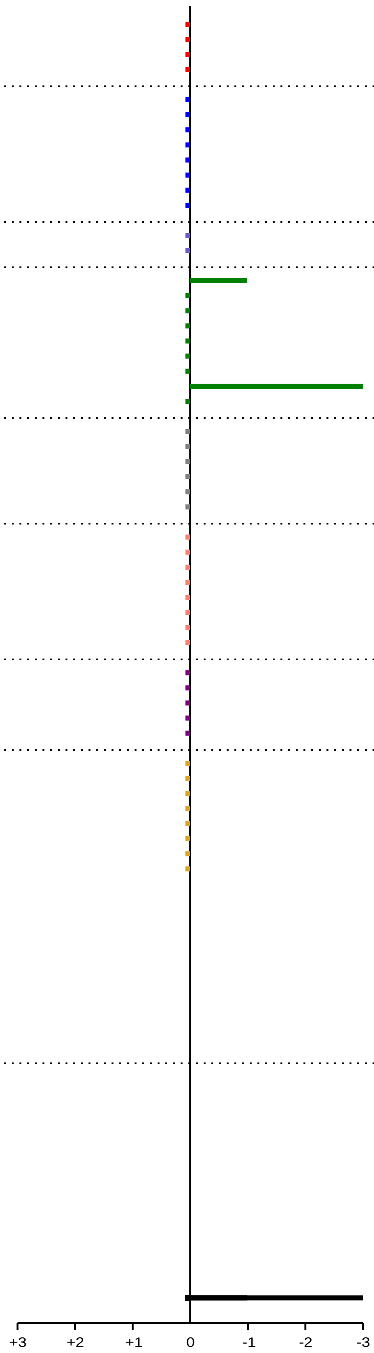

National Cancer Institute Developmental Therapeutics Program Dose Response Curves	NSC: 757 / 21	SSPL: L19P	EXP. ID: 9212EC88
		Test Date: December 14, 1992	

Leukemia

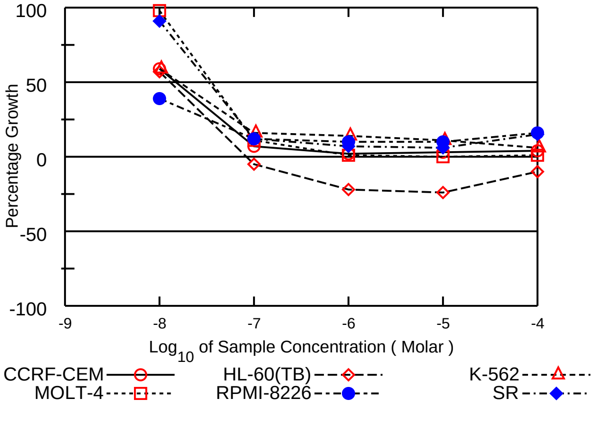

Non-Small Cell Lung Cancer

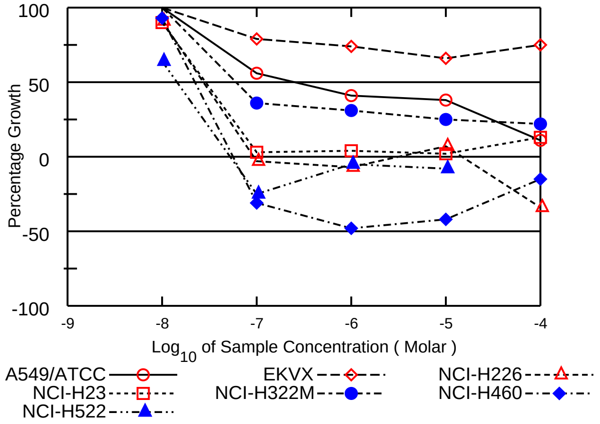

Colon Cancer

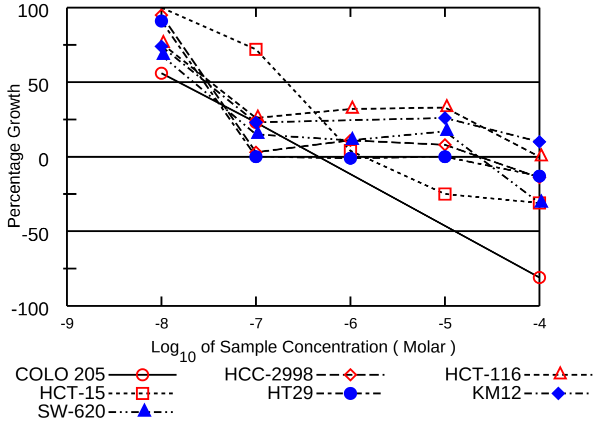

CNS Cancer

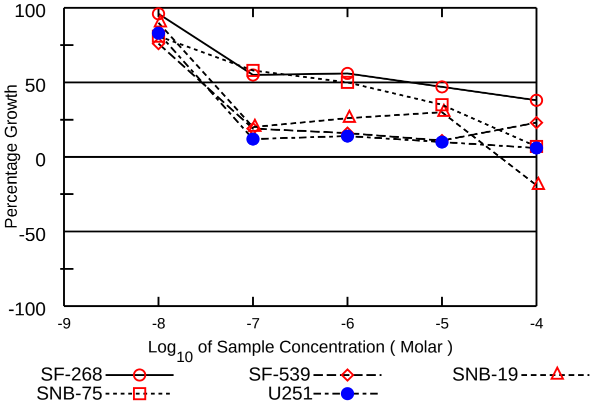

Melanoma

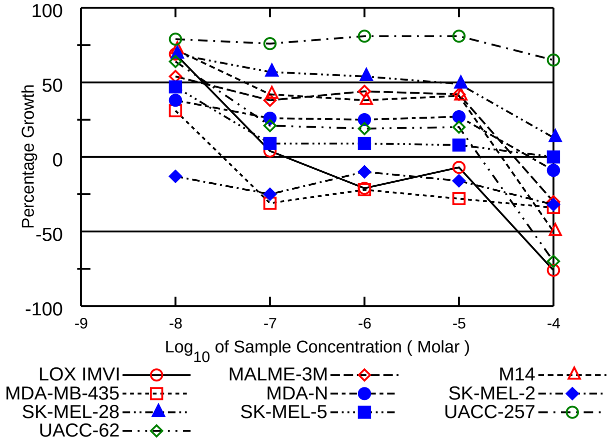

Ovarian Cancer

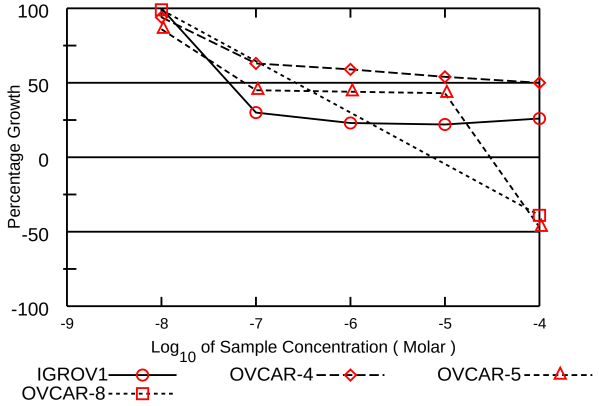

Renal Cancer

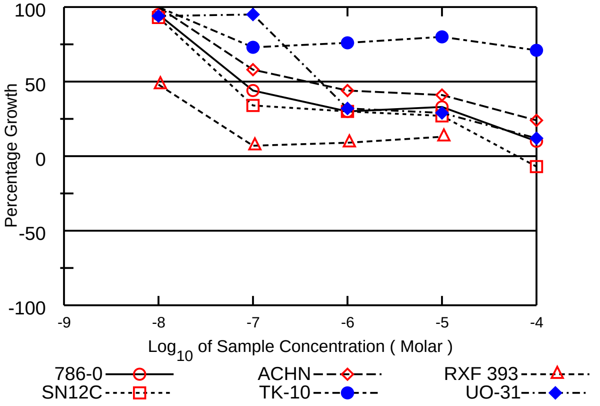

Prostate Cancer

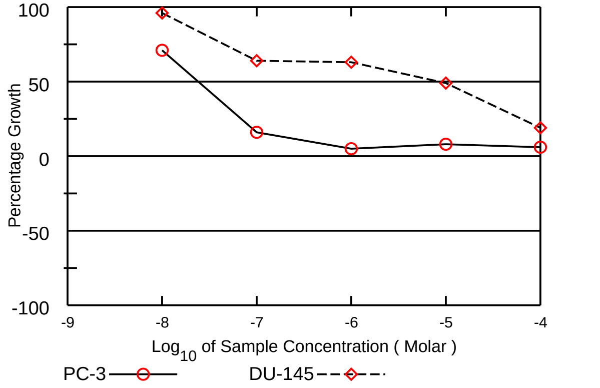

Breast Cancer

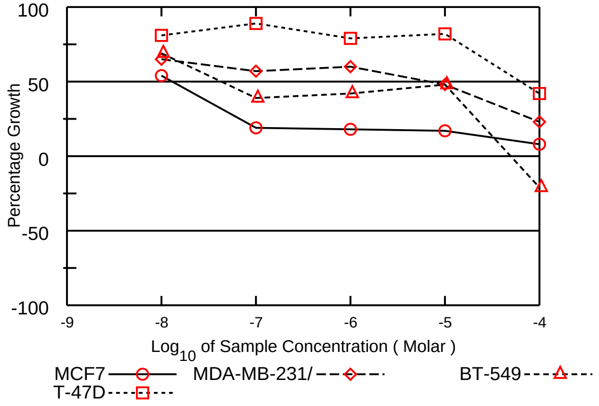

National Cancer Institute Developmental Therapeutics Program Mean Graphs	NSC :757/21	Units :Molar	SSPL :L19P	EXP. ID :9212EC88
			Test Date :December 14, 1992	

Panel/Cell Line	Log$_{10}$GI50	Log$_{10}$TGI	Log$_{10}$LC50
**Leukemia**			
CCRF-CEM	-7.82	> -4.00	> -4.00
HL-60(TB)	-7.89	-7.09	> -4.00
K-562	-7.79	> -4.00	> -4.00
MOLT-4	-7.45		> -4.00
RPMI-8226	< -8.00	> -4.00	> -4.00
SR	-7.49	> -4.00	> -4.00
**Non-Small Cell Lung Cancer**			
A549/ATCC	-6.58	> -4.00	> -4.00
EKVX	> -4.00	> -4.00	> -4.00
NCI-H226	-7.56		> -4.00
NCI-H23	-7.54	> -4.00	> -4.00
NCI-H322M	-7.21	> -4.00	> -4.00
NCI-H460	-7.65	-7.25	> -4.00
NCI-H522	-7.84	-7.28	
**Colon Cancer**			
COLO 205	-7.83		
HCC-2998	-7.51	-4.65	> -4.00
HCT-116	-7.49	-4.00	> -4.00
HCT-15	-6.68	-5.87	> -4.00
HT29	-7.55	-6.94	> -4.00
KM12	-7.53	> -4.00	> -4.00
SW-620	-7.66	-4.65	> -4.00
**CNS Cancer**			
SF-268	-5.33	> -4.00	> -4.00
SF-539	-7.54	> -4.00	> -4.00
SNB-19	-7.43	-4.39	> -4.00
SNB-75	-6.04	> -4.00	> -4.00
U251	-7.53	> -4.00	> -4.00
**Melanoma**			
LOX IMVI	-7.71	-6.84	-4.37
MALME-3M	-7.73	-4.41	> -4.00
M14	-7.26	-4.55	> -4.00
MDA-MB-435	< -8.00	-7.50	> -4.00
MDA-N	< -8.00	-4.25	> -4.00
SK-MEL-2	< -8.00	< -8.00	> -4.00
SK-MEL-28	-5.21	> -4.00	> -4.00
SK-MEL-5	< -8.00	-4.05	> -4.00
UACC-257	> -4.00	> -4.00	> -4.00
UACC-62	-7.67	-4.78	-4.22
**Ovarian Cancer**			
IGROV1	-7.28	> -4.00	> -4.00
OVCAR-4	-4.03	> -4.00	> -4.00
OVCAR-5	-7.12	-4.52	> -4.00
OVCAR-8			> -4.00
**Renal Cancer**			
786-0	-7.11	> -4.00	> -4.00
ACHN	-6.43	> -4.00	> -4.00
RXF 393	< -8.00		
SN12C	-7.27	-4.21	> -4.00
TK-10	> -4.00	> -4.00	> -4.00
UO-31	-6.29	> -4.00	> -4.00
**Prostate Cancer**			
PC-3	-7.62	> -4.00	> -4.00
DU-145	-5.08	> -4.00	> -4.00
**Breast Cancer**			
MCF7	-7.89	> -4.00	> -4.00
MDA-MB-231/ATCC	-5.15	> -4.00	> -4.00
BT-549	-7.37	-4.30	> -4.00
T-47D	-4.21	> -4.00	> -4.00
_MID	-6.95	-4.64	-4.01
Delta	1.05	3.36	0.36
Range	4.0	4.0	0.37

GI50
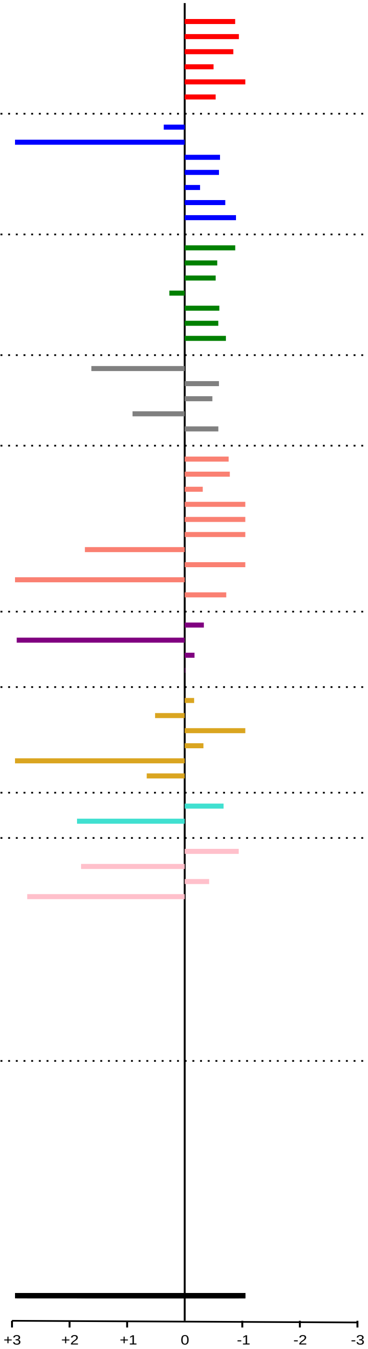

TGI
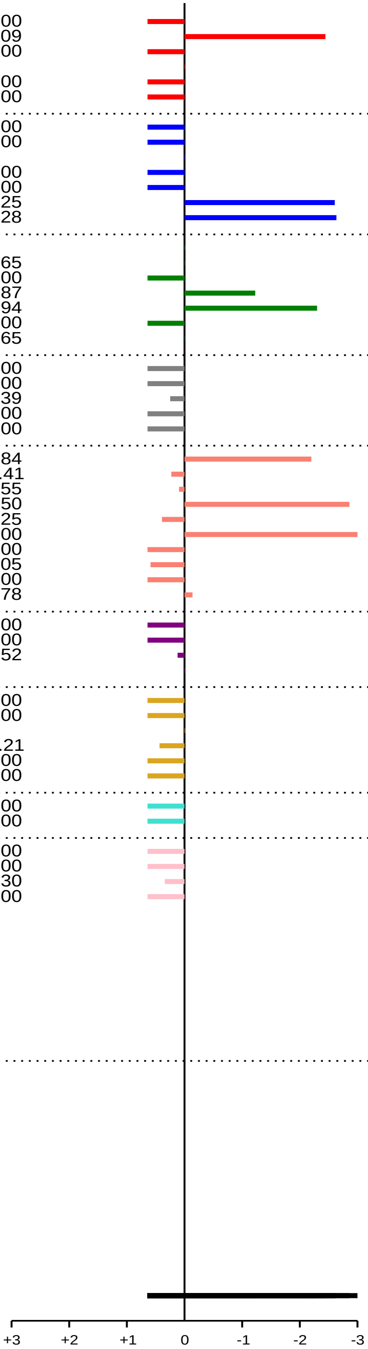

LC50
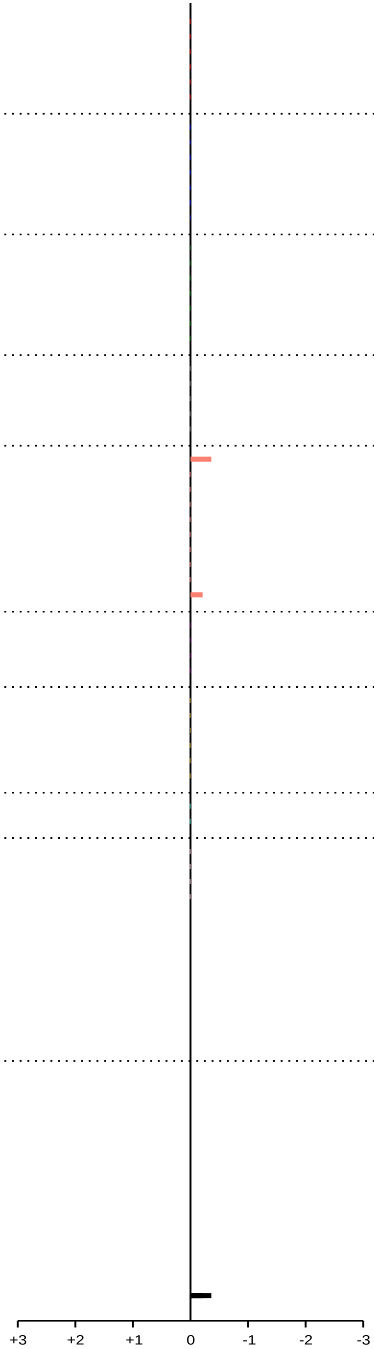

National Cancer Institute Developmental Therapeutics Program<br>Dose Response Curves	NSC: 757 / 9	SSPL:	EXP. ID: 9508LS33
		Test Date: August 29, 1995	

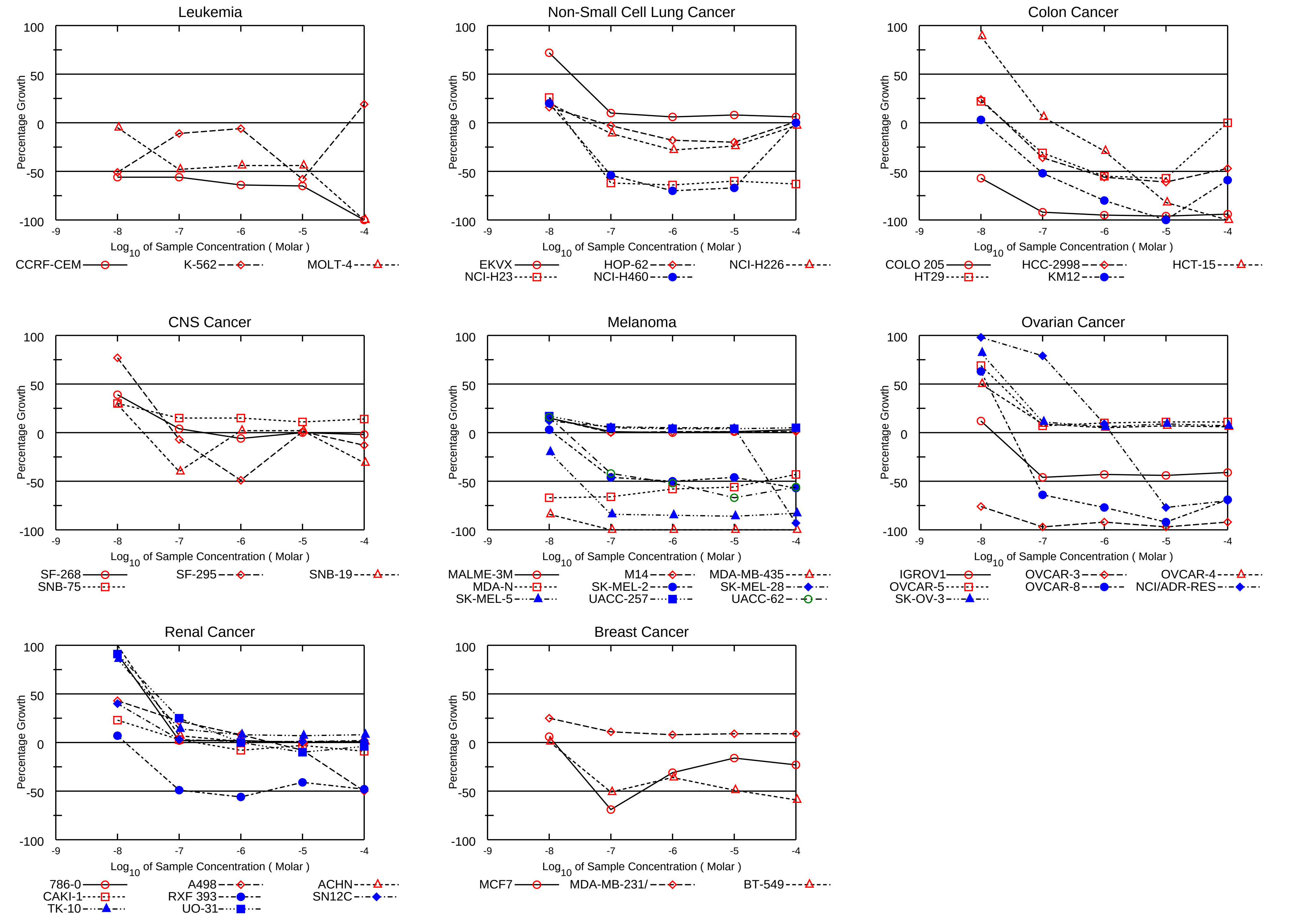

National Cancer Institute Developmental Therapeutics Program Mean Graphs	NSC :757/9	Units :Molar	SSPL :	EXP. ID :9508LS33
			Test Date :August 29, 1995	

Panel/Cell Line	Log_{10}GI50	Log_{10}TGI	Log_{10}LC50
**Leukemia**			
CCRF-CEM	< -8.00	< -8.00	< -8.00
K-562	< -8.00		
MOLT-4	< -8.00	< -8.00	-4.90
**Non-Small Cell Lung Cancer**			
EKVX	-7.64	> -4.00	> -4.00
HOP-62	< -8.00		> -4.00
NCI-H226	< -8.00	-7.35	> -4.00
NCI-H23	< -8.00	-7.71	-7.14
NCI-H460	< -8.00		
**Colon Cancer**			
COLO 205	< -8.00	< -8.00	< -8.00
HCC-2998	< -8.00	-7.59	
HCT-15	-7.53	-6.84	-5.60
HT29	< -8.00		
KM12	< -8.00	-7.95	-7.04
**CNS Cancer**			
SF-268	< -8.00		> -4.00
SF-295	-7.68		> -4.00
SNB-19	< -8.00		> -4.00
SNB-75	< -8.00	> -4.00	> -4.00
**Melanoma**			
MALME-3M	< -8.00	> -4.00	> -4.00
M14	< -8.00	> -4.00	> -4.00
MDA-MB-435	< -8.00	< -8.00	< -8.00
MDA-N	< -8.00	< -8.00	
SK-MEL-2	< -8.00	-7.93	
SK-MEL-28	< -8.00	-4.95	-4.44
SK-MEL-5	< -8.00	< -8.00	-7.53
UACC-257	< -8.00	> -4.00	> -4.00
UACC-62	< -8.00	-7.72	-6.18
**Ovarian Cancer**			
IGROV1	< -8.00	-7.80	> -4.00
OVCAR-3	< -8.00	< -8.00	< -8.00
OVCAR-4	< -8.00	> -4.00	> -4.00
OVCAR-5	-7.69	> -4.00	> -4.00
OVCAR-8	-7.90	-7.50	-7.11
NCI/ADR-RES	-6.59	-5.90	-5.31
SK-OV-3	-7.55	> -4.00	> -4.00
**Renal Cancer**			
786-0	-7.54	> -4.00	> -4.00
A498	< -8.00	-5.50	> -4.00
ACHN	-7.44	> -4.00	> -4.00
CAKI-1	< -8.00	-6.69	> -4.00
RXF 393	< -8.00	-7.88	
SN12C	< -8.00	> -4.00	> -4.00
TK-10	-7.50	> -4.00	> -4.00
UO-31	-7.38	-6.03	> -4.00
**Breast Cancer**			
MCF7	< -8.00	-7.92	
MDA-MB-231/ATCC	< -8.00	> -4.00	> -4.00
BT-549	< -8.00	-7.98	
_MID	-7.87	-6.2	-5.01
Delta	0.13	1.8	2.99
Range	1.41	4.0	4.0

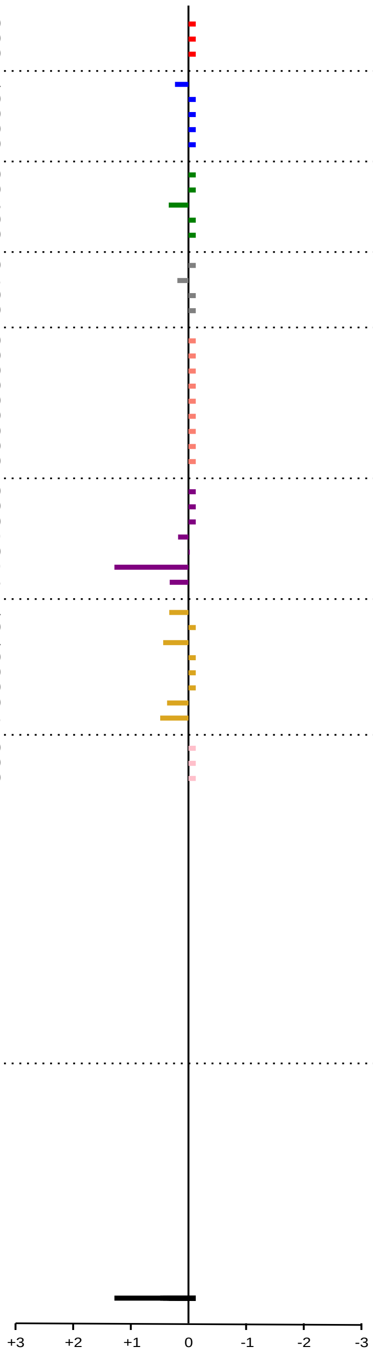

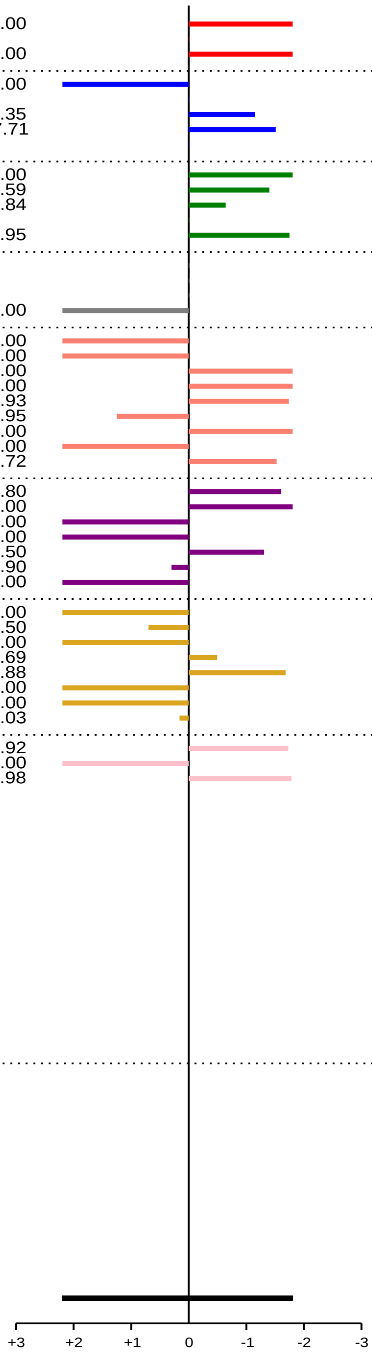

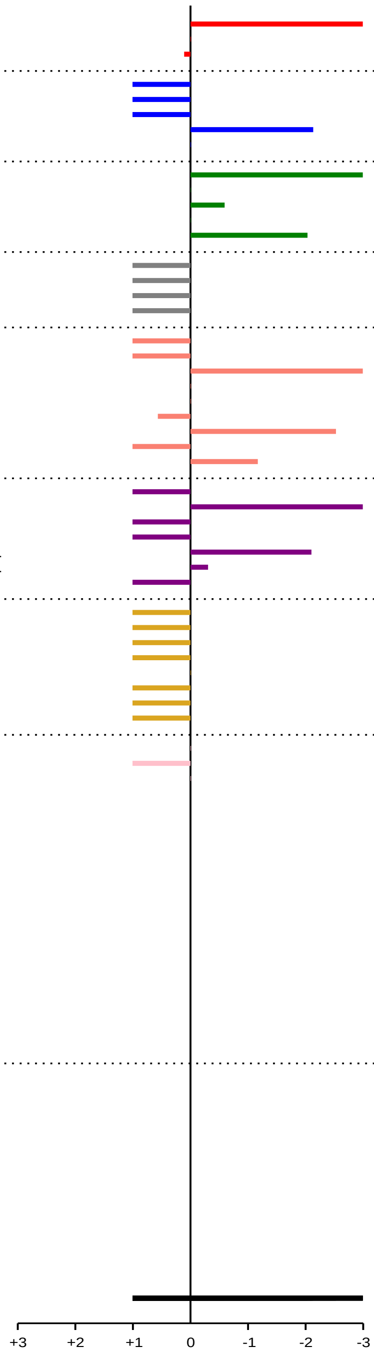

National Cancer Institute Developmental Therapeutics Program<br>Dose Response Curves	NSC: 757 / 9	SSPL:	EXP. ID: 9508NS32
		Test Date: August 29, 1995	

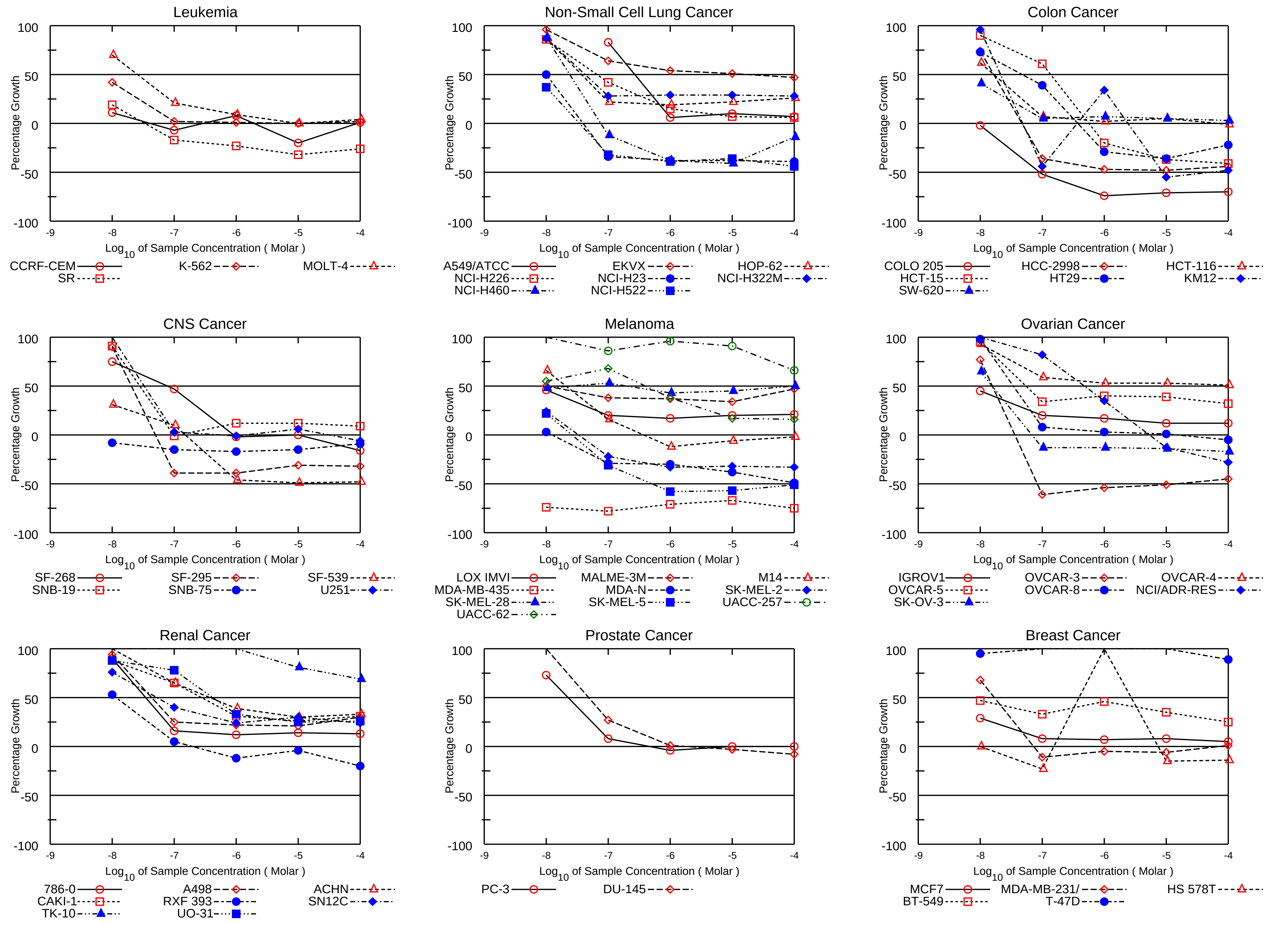

National Cancer Institute Developmental Therapeutics Program Mean Graphs	NSC :757/9	Units :Molar	SSPL :	EXP. ID :9508NS32
			Test Date :August 29, 1995	

Panel/Cell Line	Log_{10}GI50	Log_{10}TGI	Log_{10}LC50
**Leukemia**			
CCRF-CEM	< -8.00		> -4.00
K-562	< -8.00		> -4.00
MOLT-4	-7.60	> -4.00	> -4.00
SR	< -8.00	-7.47	> -4.00
**Non-Small Cell Lung Cancer**			
A549/ATCC	-6.57	> -4.00	> -4.00
EKVX	-4.70	> -4.00	> -4.00
HOP-62	-7.43	> -4.00	> -4.00
NCI-H226	-7.18	> -4.00	> -4.00
NCI-H23	< -8.00	-7.40	> -4.00
NCI-H322M	-7.37	> -4.00	> -4.00
NCI-H460	-7.62	-7.12	> -4.00
NCI-H522	< -8.00	-7.46	> -4.00
**Colon Cancer**			
COLO 205	< -8.00	< -8.00	-7.04
HCC-2998	-7.78	-7.33	> -4.00
HCT-116	-7.79	-4.23	> -4.00
HCT-15	-6.87	-6.25	> -4.00
HT29	-7.32	-6.43	> -4.00
KM12	-7.67		
SW-620	< -8.00	> -4.00	> -4.00
**CNS Cancer**			
SF-268	-7.12	-6.03	> -4.00
SF-295	-7.69	-7.30	> -4.00
SF-539	< -8.00	-6.82	> -4.00
SNB-19	-7.55		> -4.00
SNB-75	< -8.00	< -8.00	> -4.00
U251	-7.47		> -4.00
**Melanoma**			
LOX IMVI	< -8.00	> -4.00	> -4.00
MALME-3M	-7.97	> -4.00	> -4.00
M14	-7.68	-6.43	> -4.00
MDA-MB-435	< -8.00	< -8.00	< -8.00
MDA-N	< -8.00	-7.91	> -4.00
SK-MEL-2	< -8.00	-7.48	> -4.00
SK-MEL-28		> -4.00	> -4.00
SK-MEL-5	< -8.00	-7.58	-6.30
UACC-257	> -4.00	> -4.00	> -4.00
UACC-62	-6.40	> -4.00	> -4.00
**Ovarian Cancer**			
IGROV1	< -8.00	> -4.00	> -4.00
OVCAR-3	-7.81	-7.44	
OVCAR-4	> -4.00	> -4.00	> -4.00
OVCAR-5	-7.27	> -4.00	> -4.00
OVCAR-8	-7.47	-4.84	> -4.00
NCI/ADR-RES	-6.31	-5.27	> -4.00
SK-OV-3	-7.81	-7.17	> -4.00
**Renal Cancer**			
786-0	-7.46	> -4.00	> -4.00
A498	-7.36	> -4.00	> -4.00
ACHN	-6.42	> -4.00	> -4.00
CAKI-1	-6.57	> -4.00	> -4.00
RXF 393	-7.93	-6.72	> -4.00
SN12C	-7.28	> -4.00	> -4.00
TK-10	> -4.00	> -4.00	> -4.00
UO-31	-6.38	> -4.00	> -4.00
**Prostate Cancer**			
PC-3	-7.65	-6.37	> -4.00
DU-145	-7.29	-5.83	> -4.00
**Breast Cancer**			
MCF7	< -8.00	> -4.00	> -4.00
MDA-MB-231/ATCC	-7.78		> -4.00
HS 578T			> -4.00
BT-549	< -8.00	> -4.00	> -4.00
T-47D	> -4.00	> -4.00	> -4.00
_MID	-7.25	-5.42	-4.17
Delta	0.75	2.58	3.83
Range	4.0	4.0	4.0

GI50

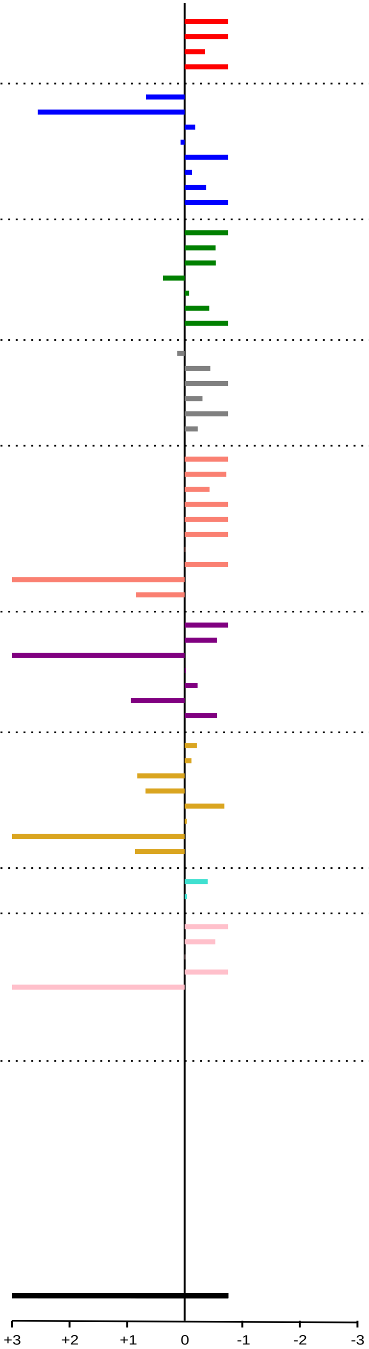

TGI

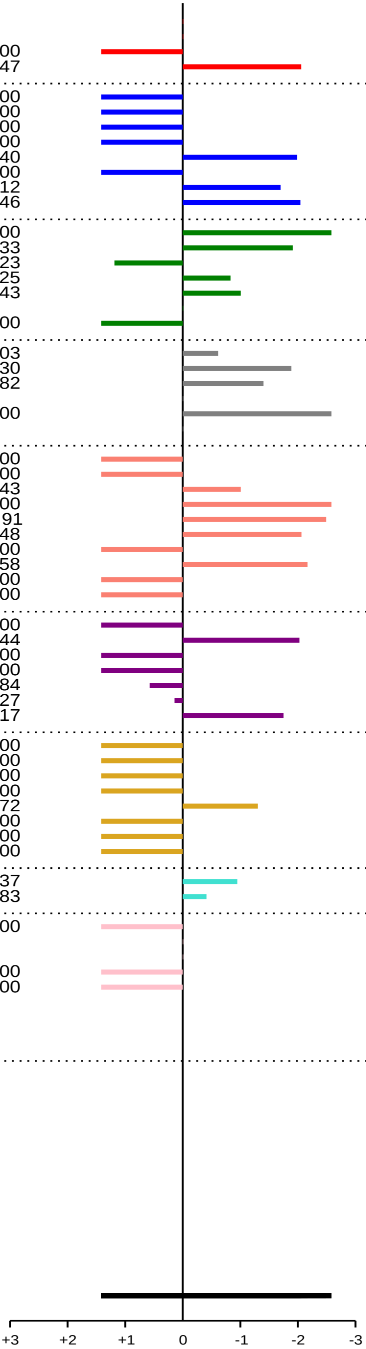

LC50

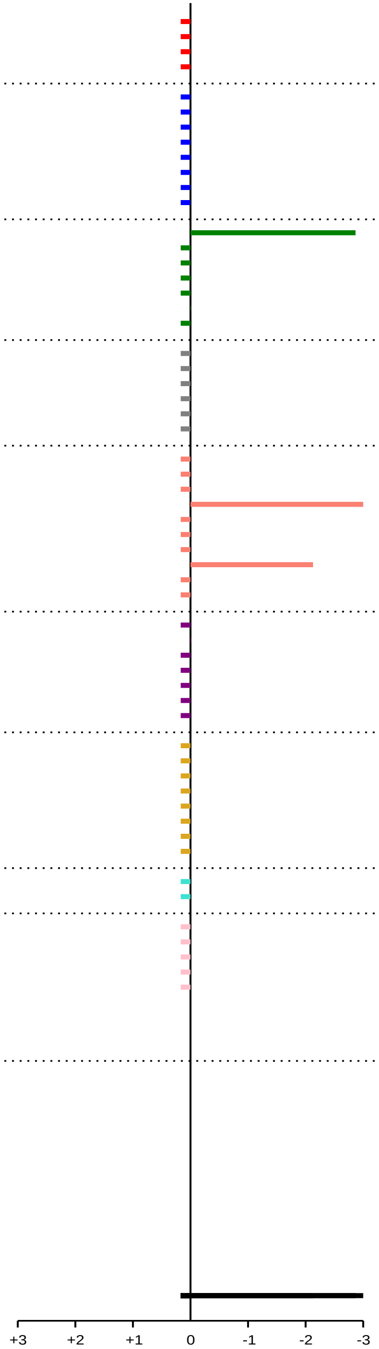

Supplement: Supplementary file 2 [file jo5c00284_si_002.pdf]
