# Supplementary material for: Deconstruction of Desacetamidocolchicine’s B Ring Reveals a Class 3 Atropisomeric AC Ring with Tubulin Binding Properties
Source: J Org Chem. 2025 May 27;90(22):7246–58. doi: 10.1021/acs.joc.5c00284 (PMC12150326; doi:10.1021/acs.joc.5c00284)
Supplement: Supplementary file 3 [file jo5c00284_si_003.zip › VCD Reports/DFT Coordinates for VCD.docx]

Supporting Information

DFT results for **bejcecine** (6 conformers at 1% or greater Boltzmann weight)

*There are no imaginary frequencies in any of the conformations

(aR)-configuration calculated at cc-pVTZ / B3LYP level

Conformer 1:

Energy = -1113.711448 Hartree

Standard orientation:

---------------------------------------------------------------------

Center Atomic Atomic Coordinates (Angstroms)

Number Number Type X Y Z

---------------------------------------------------------------------

1 6 0 1.390955 -0.295322 1.437548

2 6 0 2.712372 -0.510113 1.725751

3 6 0 4.024409 0.159012 -0.371499

4 6 0 2.972103 0.541249 -1.155687

5 6 0 1.581303 0.564441 -0.892709

6 6 0 0.846827 0.213492 0.207471

7 6 0 -1.447394 -0.706416 -0.221665

8 6 0 -0.639498 0.395848 0.090123

9 6 0 -1.241974 1.647610 0.265715

10 6 0 -2.624139 1.775457 0.112011

11 6 0 -3.421449 0.681243 -0.209408

12 6 0 -2.827772 -0.579344 -0.372831

13 6 0 0.406242 -0.630993 2.534921

14 8 0 5.296513 0.205970 -0.798706

15 6 0 3.965611 -0.347812 1.019704

16 8 0 5.020975 -0.641482 1.592890

17 6 0 5.599742 0.664797 -2.114191

18 8 0 -0.866506 -1.949594 -0.309604

19 6 0 -0.427511 2.869033 0.614296

20 8 0 -4.769768 0.728233 -0.375683

21 8 0 -3.585267 -1.670189 -0.721437

22 6 0 -0.752508 -2.494574 -1.631052

23 6 0 -5.430206 1.980541 -0.222502

24 6 0 -4.259213 -2.309228 0.370961

25 1 0 2.915223 -0.895557 2.718130

26 1 0 3.222072 0.887492 -2.148150

27 1 0 0.999918 0.929235 -1.731025

28 1 0 -3.072557 2.747524 0.249842

29 1 0 -0.255873 -1.438294 2.219476

30 1 0 -0.227744 0.224163 2.772361

31 1 0 0.920009 -0.942115 3.440897

32 1 0 5.281621 1.699239 -2.254676

33 1 0 5.134018 0.031168 -2.870880

34 1 0 6.679898 0.601571 -2.201908

35 1 0 0.198237 3.186493 -0.222124

36 1 0 -1.076118 3.702480 0.878855

37 1 0 0.242467 2.680896 1.454239

38 1 0 -0.126384 -1.854188 -2.256074

39 1 0 -0.276710 -3.465849 -1.519969

40 1 0 -1.733325 -2.615419 -2.089161

41 1 0 -5.067592 2.711887 -0.947533

42 1 0 -6.482973 1.784714 -0.404130

43 1 0 -5.304252 2.376342 0.787322

44 1 0 -3.539918 -2.679562 1.103979

45 1 0 -4.956809 -1.622985 0.852832

46 1 0 -4.808198 -3.146266 -0.053753

---------------------------------------------------------------------

Conformer 2:

Energy = -1113.709898 Hartree

Standard orientation:

---------------------------------------------------------------------

Center Atomic Atomic Coordinates (Angstroms)

Number Number Type X Y Z

---------------------------------------------------------------------

1 6 0 -1.480851 -0.059601 1.461428

2 6 0 -2.816708 0.106840 1.713194

3 6 0 -4.001686 -0.067406 -0.551579

4 6 0 -2.904575 -0.281337 -1.338848

5 6 0 -1.530105 -0.361170 -1.010027

6 6 0 -0.862502 -0.270528 0.181066

7 6 0 1.429664 0.734431 0.007683

8 6 0 0.630389 -0.418398 0.109627

9 6 0 1.239554 -1.675924 0.105296

10 6 0 2.630187 -1.771661 0.013879

11 6 0 3.419236 -0.633003 -0.099135

12 6 0 2.816566 0.635420 -0.120467

13 6 0 -0.566652 -0.020009 2.665769

14 8 0 -5.248137 -0.017392 -1.049360

15 6 0 -4.023458 0.142002 0.914804

16 8 0 -5.108253 0.342943 1.473179

17 6 0 -5.475177 -0.194209 -2.445642

18 8 0 0.768697 1.930786 -0.025185

19 6 0 0.422528 -2.940686 0.203960

20 8 0 4.774248 -0.640650 -0.212709

21 8 0 3.597229 1.760806 -0.244544

22 6 0 1.266294 3.047320 0.725680

23 6 0 5.447931 -1.894986 -0.209084

24 6 0 3.951116 2.091548 -1.593855

25 1 0 -3.078831 0.256370 2.754058

26 1 0 -3.096524 -0.406122 -2.394794

27 1 0 -0.897990 -0.524475 -1.874805

28 1 0 3.086532 -2.749396 0.025611

29 1 0 0.147737 0.799962 2.587747

30 1 0 -1.133783 0.106979 3.584381

31 1 0 0.019272 -0.936603 2.745022

32 1 0 -5.141699 -1.178597 -2.778619

33 1 0 -4.973639 0.580207 -3.028498

34 1 0 -6.549369 -0.110442 -2.578171

35 1 0 -0.166256 -3.106635 -0.700449

36 1 0 1.066893 -3.806621 0.346353

37 1 0 -0.283640 -2.904401 1.034776

38 1 0 1.992172 3.621088 0.154464

39 1 0 0.397781 3.664298 0.946531

40 1 0 1.725506 2.718455 1.657935

41 1 0 5.131095 -2.519832 -1.046670

42 1 0 6.504508 -1.664446 -0.310310

43 1 0 5.282480 -2.431811 0.727182

44 1 0 4.569629 2.984420 -1.540160

45 1 0 3.059061 2.300930 -2.188510

46 1 0 4.518290 1.282893 -2.056603

---------------------------------------------------------------------

Conformer 3:

Energy = -1113.709630 Hartree

Standard orientation:

---------------------------------------------------------------------

Center Atomic Atomic Coordinates (Angstroms)

Number Number Type X Y Z

---------------------------------------------------------------------

1 6 0 -1.461837 -0.270932 1.476542

2 6 0 -2.794918 -0.159411 1.768868

3 6 0 -4.013175 0.006274 -0.479466

4 6 0 -2.926857 -0.071227 -1.305664

5 6 0 -1.547397 -0.196112 -1.012206

6 6 0 -0.862668 -0.285427 0.169247

7 6 0 1.436161 0.713341 0.215611

8 6 0 0.628617 -0.422145 0.055523

9 6 0 1.230794 -1.652082 -0.229970

10 6 0 2.620076 -1.723243 -0.343568

11 6 0 3.421606 -0.599207 -0.174283

12 6 0 2.826969 0.645544 0.093498

13 6 0 -0.527206 -0.390376 2.659293

14 8 0 -5.267377 0.123199 -0.945273

15 6 0 -4.014511 -0.020821 1.001892

16 8 0 -5.093327 0.072303 1.598755

17 6 0 -5.515570 0.162410 -2.348532

18 8 0 0.823501 1.882141 0.595476

19 6 0 0.413657 -2.907125 -0.413167

20 8 0 4.782054 -0.616341 -0.217671

21 8 0 3.571683 1.768221 0.348257

22 6 0 0.831583 2.963964 -0.341664

23 6 0 5.442124 -1.857440 -0.446476

24 6 0 4.385011 2.268113 -0.723725

25 1 0 -3.042171 -0.170250 2.824032

26 1 0 -3.133388 -0.030244 -2.365480

27 1 0 -0.927963 -0.225957 -1.900700

28 1 0 3.074640 -2.680386 -0.548431

29 1 0 0.148094 0.465014 2.700543

30 1 0 0.095701 -1.282302 2.581059

31 1 0 -1.081279 -0.436896 3.593368

32 1 0 -6.592472 0.253769 -2.450588

33 1 0 -5.178973 -0.754866 -2.834695

34 1 0 -5.030666 1.023298 -2.811994

35 1 0 -0.171671 -2.875419 -1.334210

36 1 0 1.058536 -3.783165 -0.459335

37 1 0 -0.294568 -3.051089 0.403964

38 1 0 0.178565 3.728161 0.072862

39 1 0 1.832221 3.373596 -0.468066

40 1 0 0.439992 2.640579 -1.308455

41 1 0 5.177739 -2.275476 -1.419739

42 1 0 6.505165 -1.635679 -0.425937

43 1 0 5.207249 -2.581079 0.336402

44 1 0 5.193807 1.580810 -0.961551

45 1 0 3.780229 2.444083 -1.616249

46 1 0 4.794184 3.212718 -0.373676

---------------------------------------------------------------------

Conformer 4:

Energy = -1113.709602 Hartree

Standard orientation:

---------------------------------------------------------------------

Center Atomic Atomic Coordinates (Angstroms)

Number Number Type X Y Z

---------------------------------------------------------------------

1 6 0 -1.475442 -0.272373 1.470959

2 6 0 -2.809164 -0.162143 1.761148

3 6 0 -4.021631 0.019110 -0.488772

4 6 0 -2.933116 -0.051921 -1.312705

5 6 0 -1.554800 -0.183536 -1.017106

6 6 0 -0.873280 -0.283372 0.165210

7 6 0 1.430528 0.710787 0.197640

8 6 0 0.617711 -0.426577 0.054374

9 6 0 1.211546 -1.662595 -0.215940

10 6 0 2.599947 -1.745888 -0.336214

11 6 0 3.405902 -0.623919 -0.182748

12 6 0 2.821389 0.627593 0.078125

13 6 0 -0.542698 -0.393524 2.655206

14 8 0 -5.274505 0.142038 -0.957168

15 6 0 -4.027300 -0.022584 0.992095

16 8 0 -5.108065 0.061900 1.586945

17 6 0 -5.518350 0.195867 -2.360627

18 8 0 0.797643 1.872356 0.548506

19 6 0 0.384683 -2.913570 -0.383182

20 8 0 4.766077 -0.646406 -0.232404

21 8 0 3.589812 1.735480 0.335856

22 6 0 1.049811 3.065472 -0.202542

23 6 0 5.419932 -1.892808 -0.449224

24 6 0 4.367494 2.251814 -0.754571

25 1 0 -3.058557 -0.176663 2.815777

26 1 0 -3.136834 -0.000567 -2.372621

27 1 0 -0.933178 -0.208646 -1.904229

28 1 0 3.047525 -2.707715 -0.533748

29 1 0 0.138894 0.457048 2.693536

30 1 0 -1.097744 -0.432369 3.589057

31 1 0 0.074024 -1.290116 2.581256

32 1 0 -5.181568 -0.716885 -2.855149

33 1 0 -5.030881 1.060708 -2.813965

34 1 0 -6.594820 0.289760 -2.465065

35 1 0 -0.213267 -2.881448 -1.296002

36 1 0 1.023790 -3.793494 -0.434943

37 1 0 -0.313050 -3.050593 0.444196

38 1 0 0.198876 3.718216 -0.021161

39 1 0 1.964173 3.554584 0.124762

40 1 0 1.111119 2.845223 -1.269767

41 1 0 5.150056 -2.321039 -1.416542

42 1 0 6.484005 -1.675432 -0.435273

43 1 0 5.184898 -2.606365 0.342814

44 1 0 4.857162 3.146488 -0.377709

45 1 0 3.722950 2.518621 -1.594957

46 1 0 5.115757 1.532732 -1.080892

---------------------------------------------------------------------

Conformer 5:

Energy = -1113.709040 Hartree

Standard orientation:

---------------------------------------------------------------------

Center Atomic Atomic Coordinates (Angstroms)

Number Number Type X Y Z

---------------------------------------------------------------------

1 6 0 -1.449053 0.349070 1.377818

2 6 0 -2.773691 0.584181 1.635132

3 6 0 -4.053979 -0.164588 -0.453762

4 6 0 -2.990812 -0.584247 -1.203728

5 6 0 -1.604883 -0.602918 -0.917821

6 6 0 -0.887425 -0.212498 0.179998

7 6 0 1.407982 0.653269 -0.346505

8 6 0 0.599464 -0.409680 0.096728

9 6 0 1.187473 -1.633988 0.419636

10 6 0 2.569558 -1.787496 0.294693

11 6 0 3.367378 -0.748155 -0.167003

12 6 0 2.791237 0.494176 -0.488402

13 6 0 -0.481351 0.718706 2.480142

14 8 0 -5.319202 -0.223531 -0.900460

15 6 0 -4.015871 0.401107 0.914833

16 8 0 -5.078613 0.727210 1.456694

17 6 0 -5.602455 -0.731563 -2.202078

18 8 0 0.741664 1.793854 -0.694664

19 6 0 0.358710 -2.799381 0.900421

20 8 0 4.709012 -0.849940 -0.374493

21 8 0 3.540385 1.497762 -1.052877

22 6 0 1.285905 3.084637 -0.398665

23 6 0 5.348027 -2.093778 -0.106057

24 6 0 4.546820 2.103088 -0.228879

25 1 0 -2.990423 1.011432 2.607274

26 1 0 -3.226732 -0.969379 -2.185260

27 1 0 -1.010721 -1.001139 -1.731686

28 1 0 3.012591 -2.739522 0.542903

29 1 0 0.217313 1.485450 2.143432

30 1 0 0.120052 -0.139759 2.781578

31 1 0 -1.007220 1.095607 3.353629

32 1 0 -6.680529 -0.665613 -2.311189

33 1 0 -5.288813 -1.772567 -2.297061

34 1 0 -5.119624 -0.130600 -2.974521

35 1 0 -0.293128 -3.178931 0.111127

36 1 0 0.997255 -3.617834 1.228577

37 1 0 -0.288113 -2.521276 1.734036

38 1 0 1.763702 3.093172 0.582112

39 1 0 2.000422 3.399446 -1.154736

40 1 0 0.436413 3.764013 -0.386263

41 1 0 4.937212 -2.892539 -0.726567

42 1 0 6.395717 -1.947682 -0.352542

43 1 0 5.258337 -2.368505 0.946800

44 1 0 4.972001 2.911877 -0.818276

45 1 0 4.105382 2.512942 0.682206

46 1 0 5.324742 1.388920 0.031834

---------------------------------------------------------------------

Conformer 6:

Energy = -1113.708179 Hartree

Standard orientation:

---------------------------------------------------------------------

Center Atomic Atomic Coordinates (Angstroms)

Number Number Type X Y Z

---------------------------------------------------------------------

1 6 0 1.426077 -0.439883 1.414987

2 6 0 2.748529 -0.731561 1.617256

3 6 0 3.997869 0.130494 -0.448100

4 6 0 2.930731 0.641068 -1.133112

5 6 0 1.553570 0.678474 -0.808146

6 6 0 0.853075 0.223746 0.275775

7 6 0 -1.480866 -0.561515 -0.180247

8 6 0 -0.629988 0.463844 0.245284

9 6 0 -1.178668 1.702656 0.615843

10 6 0 -2.556859 1.883807 0.529153

11 6 0 -3.400777 0.866995 0.100222

12 6 0 -2.866533 -0.374786 -0.250413

13 6 0 0.473560 -0.864149 2.510333

14 8 0 5.251800 0.183429 -0.923506

15 6 0 3.975989 -0.538381 0.874519

16 8 0 5.040464 -0.941873 1.356230

17 6 0 5.520432 0.780716 -2.190164

18 8 0 -0.947733 -1.799363 -0.450143

19 6 0 -0.306967 2.836481 1.094014

20 8 0 -4.757468 1.080870 0.104708

21 8 0 -3.682944 -1.394162 -0.677961

22 6 0 -0.907419 -2.175403 -1.833909

23 6 0 -5.379326 1.153963 -1.186202

24 6 0 -4.167944 -2.246401 0.370423

25 1 0 2.976245 -1.235114 2.549463

26 1 0 3.154185 1.095155 -2.087707

27 1 0 0.952965 1.158391 -1.571679

28 1 0 -3.001655 2.829322 0.809525

29 1 0 -0.229699 -1.610168 2.137690

30 1 0 -0.119030 -0.021135 2.867672

31 1 0 1.011056 -1.290311 3.353515

32 1 0 6.592561 0.688372 -2.332940

33 1 0 5.240506 1.835472 -2.196961

34 1 0 4.998361 0.256502 -2.992375

35 1 0 0.320500 3.227204 0.290687

36 1 0 -0.915948 3.656563 1.470360

37 1 0 0.366071 2.519574 1.892195

38 1 0 -1.908350 -2.193234 -2.263455

39 1 0 -0.273138 -1.488186 -2.397733

40 1 0 -0.476308 -3.172865 -1.865896

41 1 0 -5.257093 0.221209 -1.735501

42 1 0 -6.435417 1.337861 -1.005158

43 1 0 -4.960946 1.981340 -1.764073

44 1 0 -4.800965 -2.990637 -0.106513

45 1 0 -3.339036 -2.742494 0.877669

46 1 0 -4.754710 -1.675017 1.091487

---------------------------------------------------------------------

DFT results for **colchicine monomer** (4 conformers at 1% or greater Boltzmann weight)

*There are no imaginary frequencies in any of the conformations

(aR,7S)-configuration calculated at 6-31G(d) / B3LYP level

Conformer 1:

Energy = -1359.435746 Hartree

Standard orientation:

---------------------------------------------------------------------

Center Atomic Atomic Coordinates (Angstroms)

Number Number Type X Y Z

---------------------------------------------------------------------

1 6 0 1.411490 0.618660 -0.427281

2 6 0 0.677325 1.974387 -0.388494

3 6 0 0.093633 2.340327 -1.769740

4 6 0 -0.659310 1.162546 -2.428423

5 6 0 -1.536718 0.430533 -1.436185

6 6 0 -0.931252 -0.346883 -0.426611

7 6 0 0.553534 -0.543176 -0.399253

8 6 0 1.006621 -1.845494 -0.356225

9 6 0 2.778769 0.647589 -0.506919

10 6 0 3.832748 -0.350240 -0.580551

11 6 0 3.546268 -1.798209 -0.428343

12 6 0 2.312787 -2.398104 -0.336508

13 8 0 4.698634 -2.497046 -0.405650

14 8 0 5.007485 0.017393 -0.739840

15 6 0 4.674164 -3.913754 -0.236818

16 6 0 -1.765681 -0.944136 0.542415

17 6 0 -3.156133 -0.792869 0.498630

18 6 0 -3.747815 -0.047963 -0.539112

19 6 0 -2.926999 0.569529 -1.488715

20 8 0 -3.942157 -1.324892 1.494331

21 8 0 -5.106876 0.018535 -0.520199

22 6 0 -4.333432 -2.685302 1.269150

23 6 0 -5.756589 0.771536 -1.540860

24 7 0 1.470706 3.080252 0.118190

25 6 0 1.594547 3.325181 1.455608

26 8 0 1.050866 2.620647 2.306324

27 6 0 2.453204 4.519949 1.831901

28 8 0 -1.225553 -1.702726 1.557447

29 6 0 -1.034354 -0.980171 2.784557

30 1 0 -0.149271 1.861519 0.316338

31 1 0 -0.582429 3.192842 -1.634066

32 1 0 0.902290 2.665594 -2.438152

33 1 0 0.071895 0.467189 -2.857734

34 1 0 -1.260134 1.542859 -3.260591

35 1 0 0.227278 -2.600107 -0.323783

36 1 0 3.226540 1.637837 -0.518545

37 1 0 2.314122 -3.480239 -0.260545

38 1 0 5.719893 -4.222383 -0.222676

39 1 0 4.194892 -4.192159 0.708658

40 1 0 4.158266 -4.404630 -1.070037

41 1 0 -3.362323 1.164305 -2.283826

42 1 0 -4.929561 -2.774318 0.353203

43 1 0 -4.942307 -2.975805 2.128299

44 1 0 -3.455626 -3.337927 1.204392

45 1 0 -6.824728 0.688359 -1.335301

46 1 0 -5.543565 0.363797 -2.536470

47 1 0 -5.460399 1.826976 -1.508938

48 1 0 1.899953 5.143033 2.540361

49 1 0 3.353903 4.161861 2.341846

50 1 0 2.754432 5.130038 0.975826

51 1 0 -1.995234 -0.645317 3.189091

52 1 0 -0.565564 -1.679379 3.480242

53 1 0 -0.375465 -0.116828 2.631021

54 1 0 1.948185 3.682210 -0.538605

---------------------------------------------------------------------

Conformer 2:

Energy = -1359.432676 Hartree

Standard orientation:

---------------------------------------------------------------------

Center Atomic Atomic Coordinates (Angstroms)

Number Number Type X Y Z

---------------------------------------------------------------------

1 6 0 1.369847 0.520104 -0.439347

2 6 0 0.690933 1.898395 -0.314921

3 6 0 0.103795 2.359227 -1.665484

4 6 0 -0.696488 1.243122 -2.373609

5 6 0 -1.589216 0.491100 -1.409880

6 6 0 -1.004073 -0.358864 -0.447350

7 6 0 0.471921 -0.609290 -0.443607

8 6 0 0.883418 -1.928975 -0.443996

9 6 0 2.734202 0.503296 -0.575178

10 6 0 3.732971 -0.530913 -0.790778

11 6 0 3.428788 -1.946717 -0.512163

12 6 0 2.173641 -2.505008 -0.414435

13 8 0 4.437159 -2.849610 -0.470289

14 8 0 4.871152 -0.202786 -1.176385

15 6 0 5.730633 -2.506285 0.061200

16 6 0 -1.851779 -0.972801 0.500247

17 6 0 -3.235742 -0.769047 0.478978

18 6 0 -3.808843 0.046193 -0.515665

19 6 0 -2.974112 0.680670 -1.441680

20 8 0 -4.030877 -1.319618 1.456802

21 8 0 -5.164089 0.159783 -0.480034

22 6 0 -4.482903 -2.648535 1.165804

23 6 0 -5.795721 0.980940 -1.458884

24 7 0 1.538215 2.940923 0.236989

25 6 0 1.708042 3.093319 1.583061

26 8 0 1.168137 2.349296 2.401894

27 6 0 2.614787 4.234385 2.009823

28 8 0 -1.332482 -1.798613 1.472362

29 6 0 -1.086787 -1.139141 2.725058

30 1 0 -0.130114 1.779519 0.395481

31 1 0 0.915002 2.690848 -2.327665

32 1 0 -0.540276 3.226136 -1.476005

33 1 0 -1.292678 1.685549 -3.177845

34 1 0 0.005684 0.546517 -2.847088

35 1 0 0.083702 -2.661910 -0.423219

36 1 0 3.216052 1.478014 -0.586919

37 1 0 2.189944 -3.588315 -0.316420

38 1 0 5.632383 -1.900761 0.967157

39 1 0 6.329351 -1.969485 -0.672623

40 1 0 6.188744 -3.465059 0.312901

41 1 0 -3.394288 1.329865 -2.201481

42 1 0 -5.092002 -2.663502 0.254275

43 1 0 -3.634590 -3.333276 1.055517

44 1 0 -5.094320 -2.957922 2.016481

45 1 0 -6.864386 0.924292 -1.247264

46 1 0 -5.604381 0.612532 -2.473990

47 1 0 -5.463449 2.023118 -1.380944

48 1 0 2.110067 4.813862 2.787954

49 1 0 3.526905 3.816713 2.449486

50 1 0 2.895148 4.901473 1.189833

51 1 0 -0.647540 -1.890857 3.384245

52 1 0 -0.384516 -0.306181 2.597469

53 1 0 -2.023209 -0.771142 3.157349

54 1 0 2.009368 3.574841 -0.393558

---------------------------------------------------------------------

Conformer 3:

Energy = -1359.432036 Hartree

Standard orientation:

---------------------------------------------------------------------

Center Atomic Atomic Coordinates (Angstroms)

Number Number Type X Y Z

---------------------------------------------------------------------

1 6 0 -1.391681 0.487302 0.370508

2 6 0 -0.699471 1.862993 0.469158

3 6 0 -0.175527 2.136900 1.895117

4 6 0 0.612341 0.945349 2.485828

5 6 0 1.536360 0.316047 1.466213

6 6 0 0.977923 -0.408937 0.393386

7 6 0 -0.498510 -0.644647 0.317926

8 6 0 -0.910432 -1.960478 0.225096

9 6 0 -2.762293 0.472515 0.331355

10 6 0 -3.787364 -0.549624 0.190358

11 6 0 -3.458029 -1.985603 0.254251

12 6 0 -2.198659 -2.541253 0.216652

13 8 0 -4.459771 -2.896962 0.228880

14 8 0 -4.968116 -0.193631 0.011790

15 6 0 -5.702534 -2.672804 0.922203

16 6 0 1.848937 -0.924297 -0.589291

17 6 0 3.233488 -0.741447 -0.501075

18 6 0 3.779630 -0.047381 0.595598

19 6 0 2.920401 0.487637 1.561812

20 8 0 4.060025 -1.189463 -1.504871

21 8 0 5.136095 0.054723 0.615784

22 6 0 4.435112 -2.568340 -1.387840

23 6 0 5.740533 0.758965 1.697245

24 7 0 -1.504539 2.984981 0.020682

25 6 0 -1.562489 3.351986 -1.293566

26 8 0 -0.949350 2.746960 -2.172826

27 6 0 -2.440834 4.550858 -1.605719

28 8 0 1.349115 -1.631418 -1.660264

29 6 0 1.175719 -0.842458 -2.848908

30 1 0 0.154798 1.830069 -0.210637

31 1 0 0.464244 3.026119 1.849478

32 1 0 -1.017852 2.372301 2.559372

33 1 0 -0.097324 0.193369 2.851304

34 1 0 1.181551 1.289894 3.354977

35 1 0 -0.109440 -2.692192 0.192503

36 1 0 -3.237511 1.449962 0.331095

37 1 0 -2.209006 -3.628722 0.199130

38 1 0 -5.530163 -2.192922 1.889808

39 1 0 -6.114124 -3.672189 1.080255

40 1 0 -6.381664 -2.063039 0.329070

41 1 0 3.320902 1.038822 2.405238

42 1 0 5.090195 -2.780529 -2.235799

43 1 0 4.979724 -2.747036 -0.453042

44 1 0 3.553922 -3.217531 -1.433500

45 1 0 6.815064 0.716932 1.513719

46 1 0 5.415837 1.806111 1.723376

47 1 0 5.515834 0.283996 2.659936

48 1 0 -2.796971 5.076072 -0.715091

49 1 0 -3.307633 4.213685 -2.184184

50 1 0 -1.877427 5.247511 -2.232846

51 1 0 0.495955 -0.001803 -2.663591

52 1 0 2.139782 -0.466700 -3.206991

53 1 0 0.738989 -1.508730 -3.595738

54 1 0 -2.035613 3.510328 0.701589

---------------------------------------------------------------------

Conformer 4:

Energy = -1359.432733 Hartree

Standard orientation:

---------------------------------------------------------------------

Center Atomic Atomic Coordinates (Angstroms)

Number Number Type X Y Z

---------------------------------------------------------------------

1 6 0 1.428194 0.617471 -0.436403

2 6 0 0.682449 1.966475 -0.484665

3 6 0 0.189767 2.287503 -1.911927

4 6 0 -0.514791 1.086810 -2.583817

5 6 0 -1.448254 0.376760 -1.628184

6 6 0 -0.903112 -0.367800 -0.556398

7 6 0 0.579753 -0.550962 -0.434895

8 6 0 1.036548 -1.848849 -0.341624

9 6 0 2.797348 0.656613 -0.422268

10 6 0 3.860949 -0.333394 -0.397915

11 6 0 3.575019 -1.783237 -0.254873

12 6 0 2.342648 -2.391963 -0.236326

13 8 0 4.728072 -2.472480 -0.152899

14 8 0 5.041547 0.041780 -0.469679

15 6 0 4.704257 -3.889257 0.017449

16 6 0 -1.784143 -0.942097 0.380358

17 6 0 -3.176777 -0.811685 0.245423

18 6 0 -3.701684 -0.093390 -0.838619

19 6 0 -2.831630 0.502996 -1.754018

20 8 0 -4.020304 -1.348133 1.191826

21 8 0 -5.055965 -0.002188 -1.052004

22 6 0 -4.249557 -2.758087 1.048750

23 6 0 -5.780930 0.793213 -0.102052

24 7 0 1.431496 3.093280 0.042272

25 6 0 1.471551 3.369397 1.378694

26 8 0 0.884920 2.676965 2.210720

27 6 0 2.294709 4.581259 1.778403

28 8 0 -1.297213 -1.665308 1.447363

29 6 0 -1.181894 -0.901971 2.660002

30 1 0 -0.188784 1.865492 0.166325

31 1 0 1.039581 2.597130 -2.534968

32 1 0 -0.497209 3.139522 -1.846313

33 1 0 -1.066481 1.440575 -3.460356

34 1 0 0.244687 0.385242 -2.949161

35 1 0 0.261175 -2.608356 -0.342213

36 1 0 3.237417 1.650299 -0.426097

37 1 0 2.346355 -3.473124 -0.148297

38 1 0 5.749546 -4.189493 0.095721

39 1 0 4.243542 -4.385199 -0.844492

40 1 0 4.170763 -4.169421 0.932813

41 1 0 -3.260253 1.064456 -2.579339

42 1 0 -4.936427 -3.037886 1.850335

43 1 0 -3.313824 -3.316228 1.153841

44 1 0 -4.709298 -2.979514 0.078406

45 1 0 -6.823777 0.772990 -0.425597

46 1 0 -5.417718 1.828558 -0.109256

47 1 0 -5.697082 0.378737 0.906110

48 1 0 1.700611 5.205005 2.452216

49 1 0 3.176142 4.243016 2.333725

50 1 0 2.626617 5.184519 0.928824

51 1 0 -2.166008 -0.557313 2.994917

52 1 0 -0.516857 -0.041704 2.517286

53 1 0 -0.754886 -1.576395 3.405102

54 1 0 1.940733 3.686211 -0.598603

---------------------------------------------------------------------

DFT results for **colchicine dimer** (6 conformers at 1% or greater Boltzmann weight)

*There are no imaginary frequencies in any of the conformations

(aR,7S)-configuration calculated at 6-31G(d) / B3LYP level

Conformer 1:

Energy = -2718.892915 Hartree

Standard orientation:

---------------------------------------------------------------------

Center Atomic Atomic Coordinates (Angstroms)

Number Number Type X Y Z

---------------------------------------------------------------------

1 6 0 3.045223 0.699744 0.179594

2 6 0 3.077237 -0.797199 0.550525

3 6 0 2.804729 -1.687957 -0.681462

4 6 0 3.615712 -1.259169 -1.923842

5 6 0 5.048793 -0.922121 -1.574754

6 6 0 5.319705 0.245028 -0.830457

7 6 0 4.222946 1.200429 -0.479647

8 6 0 4.421227 2.527560 -0.820663

9 6 0 1.897941 1.393503 0.482864

10 6 0 1.431363 2.744980 0.273613

11 6 0 2.347106 3.824217 -0.112720

12 6 0 3.621712 3.670985 -0.616284

13 8 0 1.907920 5.105668 -0.111945

14 6 0 1.042886 5.600254 0.928534

15 6 0 6.653399 0.494356 -0.440472

16 6 0 7.688899 -0.376997 -0.794102

17 6 0 7.409208 -1.515914 -1.573782

18 6 0 6.086566 -1.782937 -1.943450

19 8 0 8.966888 -0.153234 -0.338157

20 8 0 8.480630 -2.288104 -1.898436

21 6 0 9.753006 0.693476 -1.186573

22 6 0 8.253516 -3.464508 -2.670794

23 7 0 2.179671 -1.170775 1.626776

24 8 0 6.967364 1.612294 0.299815

25 6 0 7.020660 1.375535 1.716425

26 6 0 -2.975705 -0.604940 0.227088

27 6 0 -3.036941 0.932649 0.340615

28 6 0 -2.815470 1.607016 -1.031365

29 6 0 -3.648450 0.958778 -2.159895

30 6 0 -5.064038 0.661573 -1.715704

31 6 0 -5.286599 -0.365339 -0.775183

32 6 0 -4.157880 -1.230071 -0.307200

33 6 0 -4.332226 -2.595683 -0.429836

34 6 0 -1.811499 -1.216111 0.624288

35 6 0 -1.333366 -2.577186 0.678078

36 6 0 -2.200245 -3.729505 0.359822

37 6 0 -3.492024 -3.689679 -0.116120

38 8 0 -1.545930 -4.885313 0.585474

39 8 0 -0.154338 -2.809129 1.032968

40 6 0 -2.201145 -6.135567 0.369822

41 6 0 -6.601284 -0.565074 -0.303087

42 6 0 -7.665880 0.216000 -0.765002

43 6 0 -7.435487 1.208671 -1.737008

44 6 0 -6.130785 1.430181 -2.190971

45 8 0 -8.924666 0.055650 -0.234726

46 8 0 -8.533383 1.896340 -2.152268

47 6 0 -9.708348 -0.958035 -0.876901

48 6 0 -8.354771 2.926432 -3.120928

49 7 0 -2.132233 1.504403 1.319111

50 6 0 2.533133 -0.985632 2.928155

51 8 0 3.582793 -0.428858 3.262631

52 6 0 1.537355 -1.502788 3.953227

53 1 0 1.319198 -1.679768 1.405080

54 8 0 0.216969 3.015008 0.457586

55 6 0 -2.466050 1.544141 2.638402

56 8 0 -3.507787 1.050679 3.079765

57 6 0 -1.457823 2.230360 3.545208

58 1 0 -1.273027 1.962213 1.002588

59 8 0 -6.868456 -1.544050 0.628521

60 6 0 -6.862350 -1.069395 1.985307

61 1 0 4.091211 -0.999877 0.905683

62 1 0 3.052688 -2.719755 -0.406113

63 1 0 1.734952 -1.666080 -0.922293

64 1 0 3.133871 -0.387445 -2.383288

65 1 0 3.585058 -2.062870 -2.666404

66 1 0 5.370811 2.744748 -1.298329

67 1 0 1.123691 0.799339 0.961357

68 1 0 4.077541 4.610281 -0.920908

69 1 0 1.177971 6.683614 0.909064

70 1 0 0.004140 5.335487 0.738244

71 1 0 1.348421 5.213385 1.905310

72 1 0 5.848925 -2.665862 -2.525754

73 1 0 9.875698 0.246256 -2.180055

74 1 0 10.729924 0.786488 -0.706793

75 1 0 9.295305 1.684676 -1.280904

76 1 0 7.597069 -4.167322 -2.143919

77 1 0 9.234850 -3.919945 -2.810700

78 1 0 7.819933 -3.223925 -3.648972

79 1 0 6.058078 1.002020 2.086690

80 1 0 7.241828 2.340058 2.178245

81 1 0 7.813855 0.660195 1.957740

82 1 0 -4.047899 1.170627 0.681304

83 1 0 -1.751646 1.564921 -1.295516

84 1 0 -3.081616 2.665553 -0.927277

85 1 0 -3.654225 1.624594 -3.028695

86 1 0 -3.159026 0.030011 -2.477414

87 1 0 -5.290424 -2.899472 -0.838655

88 1 0 -1.050049 -0.539046 1.001498

89 1 0 -3.954018 -4.651006 -0.312950

90 1 0 -2.479729 -6.261084 -0.682636

91 1 0 -3.091209 -6.230548 1.001829

92 1 0 -1.471130 -6.896176 0.647744

93 1 0 -5.930212 2.203781 -2.923562

94 1 0 -9.231961 -1.939967 -0.778911

95 1 0 -10.674351 -0.968199 -0.366978

96 1 0 -9.858609 -0.723310 -1.937363

97 1 0 -9.349358 3.335351 -3.304389

98 1 0 -7.698247 3.719772 -2.743907

99 1 0 -7.945101 2.528884 -4.057385

100 1 0 2.065915 -2.138240 4.670447

101 1 0 0.716361 -2.066951 3.503643

102 1 0 1.125521 -0.654358 4.510838

103 1 0 -1.021377 1.486186 4.220661

104 1 0 -0.654637 2.723179 2.991930

105 1 0 -1.980838 2.965771 4.164030

106 1 0 -7.654969 -0.329224 2.137994

107 1 0 -7.048269 -1.942383 2.614543

108 1 0 -5.890735 -0.629960 2.242367

---------------------------------------------------------------------

Conformer 2:

Energy = -2718.891389 Hartree

Standard orientation:

---------------------------------------------------------------------

Center Atomic Atomic Coordinates (Angstroms)

Number Number Type X Y Z

---------------------------------------------------------------------

1 6 0 3.018847 0.716395 0.205520

2 6 0 3.070416 -0.782958 0.564039

3 6 0 2.801991 -1.666333 -0.674012

4 6 0 3.602038 -1.217467 -1.916416

5 6 0 5.032804 -0.866841 -1.571087

6 6 0 5.294061 0.296746 -0.817683

7 6 0 4.188303 1.236619 -0.453257

8 6 0 4.370323 2.569221 -0.781862

9 6 0 1.863808 1.393328 0.517183

10 6 0 1.380543 2.740625 0.319809

11 6 0 2.283481 3.834575 -0.055082

12 6 0 3.558625 3.701341 -0.563070

13 8 0 1.830061 5.110958 -0.038858

14 6 0 0.959348 5.583299 1.007080

15 6 0 6.626706 0.557522 -0.431553

16 6 0 7.670268 -0.298878 -0.797751

17 6 0 7.399807 -1.433793 -1.586460

18 6 0 6.078499 -1.712486 -1.952298

19 8 0 8.947757 -0.064584 -0.345778

20 8 0 8.478372 -2.190682 -1.923278

21 6 0 9.720439 0.797483 -1.191090

22 6 0 8.260638 -3.363407 -2.703919

23 7 0 2.182885 -1.175689 1.641794

24 8 0 6.931585 1.671981 0.317725

25 6 0 6.992887 1.422723 1.731879

26 6 0 -2.964753 -0.686387 0.203482

27 6 0 -3.049545 0.849918 0.316956

28 6 0 -2.808586 1.528717 -1.049496

29 6 0 -3.602348 0.865267 -2.196879

30 6 0 -5.023768 0.543321 -1.790380

31 6 0 -5.257572 -0.479332 -0.850024

32 6 0 -4.127537 -1.327320 -0.353570

33 6 0 -4.279854 -2.695173 -0.481792

34 6 0 -1.798796 -1.281400 0.620008

35 6 0 -1.301896 -2.635424 0.679501

36 6 0 -2.147845 -3.799602 0.348410

37 6 0 -3.430786 -3.777474 -0.152216

38 8 0 -1.482733 -4.946301 0.587877

39 8 0 -0.124829 -2.850797 1.051209

40 6 0 -2.117686 -6.205452 0.363165

41 6 0 -6.581199 -0.700463 -0.410126

42 6 0 -7.653759 0.043376 -0.920590

43 6 0 -7.407467 1.019484 -1.909692

44 6 0 -6.093828 1.275308 -2.312931

45 8 0 -8.925621 -0.252991 -0.498000

46 8 0 -8.506044 1.654079 -2.406668

47 6 0 -9.614514 0.782702 0.217585

48 6 0 -8.315893 2.657782 -3.400968

49 7 0 -2.172084 1.432484 1.313910

50 6 0 2.538524 -0.991092 2.942640

51 8 0 3.580887 -0.419862 3.275556

52 6 0 1.554346 -1.527368 3.969110

53 1 0 1.330078 -1.697918 1.421079

54 8 0 0.162770 2.994115 0.505287

55 6 0 -2.525653 1.453651 2.628134

56 8 0 -3.563706 0.935334 3.049634

57 6 0 -1.544555 2.151236 3.555780

58 1 0 -1.316865 1.908826 1.014423

59 8 0 -6.848553 -1.681221 0.518583

60 6 0 -6.740479 -1.250047 1.882354

61 1 0 4.088704 -0.976381 0.912005

62 1 0 3.062946 -2.697602 -0.408865

63 1 0 1.730909 -1.654518 -0.909666

64 1 0 3.107993 -0.347490 -2.366142

65 1 0 3.577115 -2.015048 -2.665746

66 1 0 5.315693 2.801997 -1.260524

67 1 0 1.097957 0.785625 0.992261

68 1 0 4.002986 4.648940 -0.858813

69 1 0 1.079864 6.668505 0.998465

70 1 0 -0.075905 5.306441 0.814854

71 1 0 1.270929 5.190751 1.979706

72 1 0 5.848118 -2.592767 -2.541501

73 1 0 10.698589 0.897439 -0.715241

74 1 0 9.251415 1.784297 -1.275325

75 1 0 9.843176 0.359605 -2.188714

76 1 0 7.614756 -4.077692 -2.179427

77 1 0 9.246311 -3.806628 -2.852292

78 1 0 7.819502 -3.119932 -3.677995

79 1 0 7.794674 0.713795 1.963567

80 1 0 6.035834 1.035351 2.102265

81 1 0 7.205450 2.385257 2.201819

82 1 0 -4.070817 1.073538 0.636272

83 1 0 -1.738260 1.505657 -1.288623

84 1 0 -3.095799 2.582405 -0.952029

85 1 0 -3.597316 1.529674 -3.066807

86 1 0 -3.088690 -0.055043 -2.500726

87 1 0 -5.225531 -3.011787 -0.909411

88 1 0 -1.053732 -0.594011 1.011083

89 1 0 -3.875564 -4.744956 -0.358357

90 1 0 -1.383712 -6.955876 0.657842

91 1 0 -2.373591 -6.337447 -0.694212

92 1 0 -3.018870 -6.309974 0.977640

93 1 0 -5.889810 2.040460 -3.053278

94 1 0 -9.033510 1.108863 1.088968

95 1 0 -9.823123 1.641801 -0.425501

96 1 0 -10.552969 0.337872 0.555524

97 1 0 -7.713121 3.490265 -3.018831

98 1 0 -7.840170 2.244221 -4.298062

99 1 0 -9.314570 3.017489 -3.652123

100 1 0 1.131295 -0.687385 4.531128

101 1 0 2.094981 -2.157208 4.682249

102 1 0 0.740598 -2.102398 3.520090

103 1 0 -1.102486 1.409243 4.229989

104 1 0 -0.743910 2.665425 3.018499

105 1 0 -2.090804 2.869837 4.174312

106 1 0 -7.510802 -0.506610 2.119501

107 1 0 -6.894260 -2.139492 2.497144

108 1 0 -5.751149 -0.824861 2.090702

---------------------------------------------------------------------

Conformer 3:

Energy = -2718.890231 Hartree

Standard orientation:

---------------------------------------------------------------------

Center Atomic Atomic Coordinates (Angstroms)

Number Number Type X Y Z

---------------------------------------------------------------------

1 6 0 -2.985756 -0.618129 0.216488

2 6 0 -3.071205 0.919764 0.329388

3 6 0 -2.885262 1.596696 -1.048356

4 6 0 -3.708002 0.931867 -2.172002

5 6 0 -5.113137 0.605523 -1.718429

6 6 0 -5.308129 -0.436662 -0.790650

7 6 0 -4.147806 -1.268979 -0.335500

8 6 0 -4.267121 -2.635603 -0.500645

9 6 0 -1.814638 -1.207691 0.627700

10 6 0 -1.304316 -2.557278 0.686611

11 6 0 -2.124727 -3.727149 0.317382

12 6 0 -3.400825 -3.712355 -0.199474

13 8 0 -1.443099 -4.868553 0.535427

14 6 0 -2.052704 -6.131423 0.267073

15 6 0 -6.617435 -0.680817 -0.312290

16 6 0 -7.696940 0.093868 -0.758537

17 6 0 -7.488871 1.104399 -1.717991

18 6 0 -6.196349 1.353836 -2.186809

19 8 0 -8.965691 -0.130932 -0.270614

20 8 0 -8.601319 1.789711 -2.099274

21 6 0 -9.340120 0.744771 0.803208

22 6 0 -8.448784 2.849552 -3.040399

23 7 0 -2.147167 1.516498 1.275648

24 8 0 -6.787947 -1.621882 0.672689

25 6 0 -7.712829 -2.691572 0.425595

26 6 0 3.061454 0.703574 0.164833

27 6 0 3.106996 -0.777517 0.593741

28 6 0 2.865737 -1.719180 -0.606858

29 6 0 3.685849 -1.323764 -1.854146

30 6 0 5.109836 -0.954775 -1.500409

31 6 0 5.356062 0.241707 -0.795307

32 6 0 4.242476 1.194933 -0.495435

33 6 0 4.428529 2.510757 -0.883833

34 6 0 1.900618 1.392543 0.424121

35 6 0 1.419094 2.728388 0.156427

36 6 0 2.325736 3.804438 -0.259535

37 6 0 3.610300 3.649721 -0.736816

38 8 0 1.869408 5.078524 -0.316905

39 8 0 0.198852 2.988108 0.313931

40 6 0 0.977062 5.601836 0.685762

41 6 0 6.681312 0.522804 -0.398318

42 6 0 7.732608 -0.346162 -0.708681

43 6 0 7.477774 -1.515507 -1.450912

44 6 0 6.163426 -1.813706 -1.826259

45 8 0 9.002167 -0.089328 -0.246695

46 8 0 8.563446 -2.283596 -1.735660

47 6 0 9.784692 0.742810 -1.112651

48 6 0 8.361806 -3.486886 -2.472795

49 7 0 2.197870 -1.121208 1.669992

50 6 0 -2.462590 1.637652 2.595744

51 8 0 -3.507276 1.193837 3.076739

52 6 0 -1.424503 2.348955 3.448722

53 1 0 -1.289322 1.951149 0.925923

54 8 0 -0.132155 -2.762238 1.078981

55 6 0 2.531853 -0.891675 2.969337

56 8 0 3.575528 -0.321895 3.300707

57 6 0 1.523055 -1.378752 3.996408

58 1 0 1.337228 -1.631388 1.452636

59 8 0 6.971251 1.670663 0.305126

60 6 0 7.006421 1.485989 1.729951

61 1 0 -4.076608 1.142002 0.697155

62 1 0 -3.177191 2.647950 -0.940256

63 1 0 -1.822751 1.583006 -1.320813

64 1 0 -3.200894 0.014368 -2.494126

65 1 0 -3.733702 1.598533 -3.039855

66 1 0 -5.199260 -2.960810 -0.951884

67 1 0 -1.074378 -0.515911 1.019832

68 1 0 -3.826443 -4.680653 -0.439517

69 1 0 -2.959516 -6.269128 0.866649

70 1 0 -1.309417 -6.877207 0.549990

71 1 0 -2.293063 -6.238098 -0.796834

72 1 0 -6.016592 2.138056 -2.913164

73 1 0 -10.347195 0.446777 1.103173

74 1 0 -9.351073 1.788146 0.470086

75 1 0 -8.656220 0.634596 1.652783

76 1 0 -8.051756 2.482139 -3.994200

77 1 0 -7.793112 3.638052 -2.651992

78 1 0 -9.450290 3.253117 -3.195669

79 1 0 -8.741026 -2.329154 0.381393

80 1 0 -7.591398 -3.383417 1.261743

81 1 0 -7.468747 -3.209500 -0.510283

82 1 0 4.117470 -0.952619 0.972621

83 1 0 1.799043 -1.724858 -0.861827

84 1 0 3.127219 -2.735371 -0.289202

85 1 0 3.675197 -2.153719 -2.567873

86 1 0 3.197619 -0.475751 -2.349725

87 1 0 5.382689 2.722689 -1.354720

88 1 0 1.126441 0.806546 0.912754

89 1 0 4.058087 4.582775 -1.071106

90 1 0 1.264540 5.254734 1.682658

91 1 0 -0.054620 5.319242 0.482923

92 1 0 1.101816 6.685148 0.628753

93 1 0 5.944558 -2.719851 -2.379515

94 1 0 9.315968 1.725363 -1.238038

95 1 0 10.756757 0.860919 -0.628544

96 1 0 9.920270 0.269592 -2.092322

97 1 0 7.936140 -3.283259 -3.462754

98 1 0 9.350937 -3.932435 -2.587324

99 1 0 7.709158 -4.182115 -1.931342

100 1 0 -1.926244 3.107410 4.056948

101 1 0 -0.634471 2.817719 2.856921

102 1 0 -0.971960 1.625429 4.136159

103 1 0 2.040866 -2.000413 4.733407

104 1 0 1.111249 -0.515238 4.530260

105 1 0 0.703115 -1.948865 3.552445

106 1 0 7.803927 0.789137 2.008358

107 1 0 7.210059 2.469063 2.159599

108 1 0 6.042770 1.114914 2.099897

---------------------------------------------------------------------

Conformer 4:

Energy = -2718.889817

Standard orientation:

---------------------------------------------------------------------

Center Atomic Atomic Coordinates (Angstroms)

Number Number Type X Y Z

---------------------------------------------------------------------

1 6 0 3.063538 0.706761 0.141913

2 6 0 3.095609 -0.771344 0.582589

3 6 0 2.855487 -1.721108 -0.611948

4 6 0 3.687458 -1.343149 -1.856706

5 6 0 5.111423 -0.981394 -1.495785

6 6 0 5.360660 0.220023 -0.800396

7 6 0 4.251318 1.183159 -0.517073

8 6 0 4.448040 2.493384 -0.919072

9 6 0 1.907676 1.407771 0.391563

10 6 0 1.437584 2.745213 0.111749

11 6 0 2.351954 3.809114 -0.318094

12 6 0 3.636859 3.639296 -0.789657

13 8 0 1.904890 5.085190 -0.395210

14 6 0 1.009981 5.630324 0.593475

15 6 0 6.684909 0.496444 -0.396971

16 6 0 7.732380 -0.382824 -0.690803

17 6 0 7.474862 -1.557957 -1.422953

18 6 0 6.161326 -1.850775 -1.805378

19 8 0 9.000274 -0.130048 -0.222158

20 8 0 8.557193 -2.336472 -1.691455

21 6 0 9.795084 0.687541 -1.090791

22 6 0 8.352738 -3.546022 -2.417509

23 7 0 2.176808 -1.101115 1.655220

24 8 0 6.977794 1.649898 0.295990

25 6 0 6.999922 1.481448 1.723015

26 6 0 -2.968190 -0.595632 0.158983

27 6 0 -3.036818 0.944542 0.220575

28 6 0 -2.814088 1.573642 -1.172941

29 6 0 -3.643383 0.888455 -2.282419

30 6 0 -5.057373 0.600156 -1.829428

31 6 0 -5.278604 -0.399834 -0.854738

32 6 0 -4.143036 -1.244206 -0.362378

33 6 0 -4.306391 -2.612928 -0.456599

34 6 0 -1.804802 -1.188423 0.585346

35 6 0 -1.321322 -2.544967 0.686960

36 6 0 -2.173685 -3.711688 0.378231

37 6 0 -3.459097 -3.693327 -0.115505

38 8 0 -1.512329 -4.856040 0.635381

39 8 0 -0.148995 -2.760492 1.071570

40 6 0 -2.151390 -6.116993 0.432267

41 6 0 -6.586897 -0.594765 -0.371897

42 6 0 -7.662525 0.165578 -0.860167

43 6 0 -7.431015 1.136253 -1.845776

44 6 0 -6.131025 1.349839 -2.309653

45 8 0 -8.926978 -0.013068 -0.346120

46 8 0 -8.461689 1.853062 -2.403898

47 6 0 -9.641812 -1.121382 -0.912755

48 6 0 -9.096052 2.804681 -1.536050

49 7 0 -2.137716 1.553244 1.181539

50 6 0 2.501966 -0.859517 2.954752

51 8 0 3.543182 -0.286239 3.287518

52 6 0 1.486001 -1.336045 3.979714

53 1 0 1.320771 -1.618543 1.437051

54 8 0 0.219725 3.017146 0.268346

55 6 0 -2.482375 1.650383 2.495065

56 8 0 -3.530004 1.180373 2.947985

57 6 0 -1.478410 2.369743 3.380477

58 1 0 -1.274271 1.994226 0.852719

59 8 0 -6.844918 -1.553862 0.582721

60 6 0 -6.865677 -1.043036 1.926716

61 1 0 4.102525 -0.950389 0.969183

62 1 0 3.106609 -2.736569 -0.283776

63 1 0 1.790589 -1.720433 -0.874499

64 1 0 3.209125 -0.496067 -2.363321

65 1 0 3.675734 -2.179451 -2.562957

66 1 0 5.405777 2.693434 -1.387894

67 1 0 1.127660 0.832395 0.883469

68 1 0 4.091786 4.564876 -1.134858

69 1 0 1.147950 6.711382 0.524841

70 1 0 -0.023101 5.357701 0.384268

71 1 0 1.284150 5.291659 1.596890

72 1 0 5.940284 -2.760761 -2.351411

73 1 0 10.764462 0.803548 -0.600829

74 1 0 9.334432 1.672117 -1.229521

75 1 0 9.934297 0.203368 -2.064573

76 1 0 9.339570 -3.999565 -2.519794

77 1 0 7.936005 -3.349837 -3.412748

78 1 0 7.691169 -4.230999 -1.873819

79 1 0 7.209130 2.467549 2.142947

80 1 0 7.788751 0.780699 2.015967

81 1 0 6.029967 1.123524 2.089429

82 1 0 -4.049177 1.190108 0.551601

83 1 0 -1.749539 1.526166 -1.432891

84 1 0 -3.083315 2.634148 -1.103155

85 1 0 -3.653293 1.528929 -3.169884

86 1 0 -3.150207 -0.047106 -2.573120

87 1 0 -5.259825 -2.933146 -0.864122

88 1 0 -1.049912 -0.496376 0.948091

89 1 0 -3.910275 -4.662350 -0.298980

90 1 0 -1.417838 -6.864618 0.734853

91 1 0 -2.412306 -6.263755 -0.621886

92 1 0 -3.049871 -6.209892 1.052477

93 1 0 -5.973876 2.114877 -3.064631

94 1 0 -10.620393 -1.133053 -0.428144

95 1 0 -9.769619 -0.988569 -1.993415

96 1 0 -9.120576 -2.063251 -0.712644

97 1 0 -9.863395 3.296267 -2.137794

98 1 0 -9.557967 2.310228 -0.677346

99 1 0 -8.371554 3.551843 -1.188909

100 1 0 1.065739 -0.466637 4.497236

101 1 0 1.999801 -1.944649 4.730226

102 1 0 0.672421 -1.916046 3.536839

103 1 0 -1.039583 1.649546 4.080055

104 1 0 -0.676202 2.847358 2.812620

105 1 0 -2.005270 3.122567 3.974449

106 1 0 -7.049023 -1.901644 2.575985

107 1 0 -5.903716 -0.583821 2.184853

108 1 0 -7.670247 -0.310096 2.048504

---------------------------------------------------------------------

Conformer 5:

Energy = -2718.886640 Hartree

Standard orientation:

---------------------------------------------------------------------

Center Atomic Atomic Coordinates (Angstroms)

Number Number Type X Y Z

---------------------------------------------------------------------

1 6 0 2.971953 0.693097 0.314915

2 6 0 3.041571 -0.837782 0.503849

3 6 0 2.697298 -1.582997 -0.805967

4 6 0 3.397413 -0.988243 -2.044713

5 6 0 4.849593 -0.670735 -1.767974

6 6 0 5.168237 0.412360 -0.924022

7 6 0 4.083950 1.291223 -0.379687

8 6 0 4.218373 2.651441 -0.598954

9 6 0 1.846849 1.325387 0.789625

10 6 0 1.347204 2.681357 0.782354

11 6 0 2.207969 3.817955 0.439197

12 6 0 3.427023 3.748159 -0.201752

13 8 0 1.757602 5.081266 0.624598

14 6 0 0.970449 5.437023 1.777534

15 6 0 6.529394 0.649881 -0.614181

16 6 0 7.535836 -0.171198 -1.140242

17 6 0 7.200504 -1.225557 -2.012506

18 6 0 5.858236 -1.466513 -2.317175

19 8 0 8.856729 0.048811 -0.816678

20 8 0 8.248241 -1.957569 -2.478376

21 6 0 9.333533 -0.762353 0.267263

22 6 0 7.970190 -3.059245 -3.339588

23 7 0 2.206737 -1.357656 1.570902

24 8 0 6.831256 1.635212 0.292436

25 6 0 7.741619 2.667856 -0.116448

26 6 0 -2.976638 -0.691186 0.295980

27 6 0 -3.039240 0.828979 0.549528

28 6 0 -2.690365 1.626700 -0.725966

29 6 0 -3.415025 1.094663 -1.981829

30 6 0 -4.867185 0.774427 -1.702134

31 6 0 -5.183339 -0.332967 -0.887298

32 6 0 -4.110022 -1.250656 -0.392604

33 6 0 -4.284274 -2.602197 -0.637470

34 6 0 -1.845519 -1.348616 0.717410

35 6 0 -1.362140 -2.708050 0.639081

36 6 0 -2.252313 -3.822307 0.293540

37 6 0 -3.496089 -3.719570 -0.293840

38 8 0 -1.812773 -5.096429 0.424381

39 8 0 -0.157402 -2.951651 0.904264

40 6 0 -0.988236 -5.498115 1.535187

41 6 0 -6.538972 -0.559145 -0.564923

42 6 0 -7.550859 0.274249 -1.052514

43 6 0 -7.223924 1.350412 -1.900088

44 6 0 -5.880494 1.596535 -2.203718

45 8 0 -8.855573 0.079894 -0.664289

46 8 0 -8.273750 2.087173 -2.352229

47 6 0 -9.574743 -0.862227 -1.470618

48 6 0 -7.999569 3.202898 -3.195949

49 7 0 -2.217181 1.289518 1.651900

50 6 0 2.632707 -1.369957 2.864864

51 8 0 3.707104 -0.879779 3.218346

52 6 0 1.680032 -2.020354 3.855376

53 1 0 1.333808 -1.833058 1.328098

54 8 0 0.150665 2.900754 1.101152

55 6 0 -2.652023 1.189117 2.937753

56 8 0 -3.721944 0.653773 3.241179

57 6 0 -1.721236 1.773246 3.987657

58 1 0 -1.340901 1.779033 1.451946

59 8 0 -6.899357 -1.615981 0.241258

60 6 0 -7.033122 -1.268320 1.629536

61 1 0 4.076733 -1.062689 0.776081

62 1 0 2.986878 -2.632444 -0.676133

63 1 0 1.611650 -1.565128 -0.961187

64 1 0 2.870810 -0.076650 -2.351844

65 1 0 3.314955 -1.694480 -2.876968

66 1 0 5.104449 2.943389 -1.152893

67 1 0 1.121561 0.664640 1.257467

68 1 0 3.836812 4.724336 -0.450904

69 1 0 -0.078124 5.181795 1.633492

70 1 0 1.097706 6.517010 1.876333

71 1 0 1.349519 4.942187 2.676540

72 1 0 5.581642 -2.281976 -2.975385

73 1 0 10.378224 -0.483205 0.420979

74 1 0 9.272489 -1.826434 0.014623

75 1 0 8.762532 -0.565820 1.182131

76 1 0 7.476432 -2.729976 -4.261584

77 1 0 7.346559 -3.810131 -2.839897

78 1 0 8.939830 -3.495584 -3.583173

79 1 0 8.755553 2.282856 -0.235133

80 1 0 7.711311 3.416821 0.677626

81 1 0 7.413804 3.125185 -1.058154

82 1 0 -4.075219 1.047878 0.821607

83 1 0 -1.606855 1.597572 -0.893825

84 1 0 -2.961970 2.673926 -0.548204

85 1 0 -3.336914 1.837266 -2.782168

86 1 0 -2.900425 0.193532 -2.336879

87 1 0 -5.202540 -2.863546 -1.152694

88 1 0 -1.102078 -0.713498 1.192437

89 1 0 -3.930648 -4.683734 -0.547648

90 1 0 -1.332164 -5.033579 2.464107

91 1 0 0.056521 -5.244468 1.363567

92 1 0 -1.120274 -6.580073 1.599998

93 1 0 -5.607081 2.432254 -2.837705

94 1 0 -9.108814 -1.852770 -1.422669

95 1 0 -10.584202 -0.912801 -1.056342

96 1 0 -9.624903 -0.526673 -2.513243

97 1 0 -7.503374 2.891554 -4.123102

98 1 0 -8.970678 3.639725 -3.432902

99 1 0 -7.379745 3.948282 -2.683385

100 1 0 2.234328 -2.751343 4.451942

101 1 0 0.831698 -2.511601 3.372307

102 1 0 1.304632 -1.254780 4.543391

103 1 0 -2.288639 2.462760 4.620338

104 1 0 -1.357492 0.964388 4.630710

105 1 0 -0.865340 2.297139 3.554653

106 1 0 -7.833368 -0.533627 1.767667

107 1 0 -7.288570 -2.192723 2.151769

108 1 0 -6.091034 -0.869611 2.025355

---------------------------------------------------------------------

Conformer 6:

Energy = -2718.886123

Standard orientation:

---------------------------------------------------------------------

Center Atomic Atomic Coordinates (Angstroms)

Number Number Type X Y Z

---------------------------------------------------------------------

1 6 0 -2.950789 -0.670188 0.283000

2 6 0 -2.990699 0.868306 0.380090

3 6 0 -2.589846 1.522252 -0.960412

4 6 0 -3.295883 0.876003 -2.173577

5 6 0 -4.760556 0.615137 -1.900144

6 6 0 -5.124671 -0.393630 -0.977889

7 6 0 -4.080612 -1.277369 -0.368261

8 6 0 -4.277406 -2.642746 -0.480971

9 6 0 -1.836550 -1.299724 0.784303

10 6 0 -1.374065 -2.667276 0.839170

11 6 0 -2.282869 -3.796114 0.602431

12 6 0 -3.516029 -3.733252 -0.012502

13 8 0 -1.871632 -5.056062 0.875331

14 6 0 -1.052890 -5.350981 2.023521

15 6 0 -6.486464 -0.556672 -0.657602

16 6 0 -7.475318 0.238620 -1.259775

17 6 0 -7.101405 1.212628 -2.197266

18 6 0 -5.749503 1.397841 -2.495643

19 8 0 -8.796090 0.090672 -0.902557

20 8 0 -8.037746 1.960469 -2.868440

21 6 0 -9.465902 -0.997761 -1.556690

22 6 0 -8.754273 2.918370 -2.074331

23 7 0 -2.192550 1.422274 1.456742

24 8 0 -6.883009 -1.516283 0.247151

25 6 0 -7.034947 -1.019360 1.588176

26 6 0 2.978238 0.700214 0.277669

27 6 0 3.020428 -0.819948 0.534691

28 6 0 2.659944 -1.615132 -0.738947

29 6 0 3.392596 -1.095811 -1.995651

30 6 0 4.849073 -0.794928 -1.716382

31 6 0 5.180687 0.309173 -0.903122

32 6 0 4.120447 1.242611 -0.409815

33 6 0 4.315367 2.591577 -0.653978

34 6 0 1.854370 1.373804 0.693355

35 6 0 1.390103 2.739416 0.607883

36 6 0 2.300370 3.840896 0.274104

37 6 0 3.544350 3.720432 -0.309534

38 8 0 1.881944 5.121252 0.410834

39 8 0 0.185965 3.000822 0.858832

40 6 0 1.052888 5.528346 1.516175

41 6 0 6.539475 0.516675 -0.581199

42 6 0 7.539630 -0.331189 -1.068065

43 6 0 7.197629 -1.403646 -1.914439

44 6 0 5.850794 -1.631570 -2.217220

45 8 0 8.846951 -0.155110 -0.680149

46 8 0 8.236929 -2.155417 -2.365995

47 6 0 9.578042 0.780694 -1.483129

48 6 0 7.947184 -3.266826 -3.210227

49 7 0 2.193402 -1.267137 1.639270

50 6 0 -2.664568 1.439842 2.733441

51 8 0 -3.743910 0.932935 3.052650

52 6 0 -1.764459 2.117439 3.753196

53 1 0 -1.314970 1.900945 1.235841

54 8 0 -0.174313 -2.904051 1.129800

55 6 0 2.630316 -1.162347 2.924350

56 8 0 3.701199 -0.626720 3.223452

57 6 0 1.700643 -1.739796 3.978855

58 1 0 1.321894 -1.766245 1.442256

59 8 0 6.915204 1.569221 0.223639

60 6 0 7.039971 1.222625 1.613020

61 1 0 -4.029593 1.131947 0.595663

62 1 0 -2.841137 2.587726 -0.900390

63 1 0 -1.503536 1.452424 -1.095255

64 1 0 -2.792025 -0.066318 -2.420827

65 1 0 -3.184766 1.530503 -3.043778

66 1 0 -5.191902 -2.938184 -0.984385

67 1 0 -1.089356 -0.631922 1.205772

68 1 0 -3.965399 -4.710564 -0.173097

69 1 0 -1.214067 -6.414451 2.211121

70 1 0 -1.379167 -4.774152 2.893847

71 1 0 -0.002512 -5.147732 1.821701

72 1 0 -5.483190 2.168030 -3.213828

73 1 0 -9.464172 -0.856799 -2.643857

74 1 0 -10.494042 -0.987972 -1.188767

75 1 0 -8.993700 -1.952728 -1.304278

76 1 0 -9.437777 3.425380 -2.758619

77 1 0 -8.063543 3.651855 -1.640014

78 1 0 -9.321430 2.427397 -1.279149

79 1 0 -7.322030 -1.877045 2.199903

80 1 0 -7.820274 -0.257116 1.629859

81 1 0 -6.091208 -0.600551 1.958279

82 1 0 4.053893 -1.051921 0.805768

83 1 0 1.576994 -1.570088 -0.907462

84 1 0 2.916161 -2.665922 -0.559350

85 1 0 3.304529 -1.839034 -2.794358

86 1 0 2.890494 -0.188531 -2.352942

87 1 0 5.238151 2.839297 -1.167787

88 1 0 1.101064 0.749703 1.167540

89 1 0 3.994953 4.678339 -0.559042

90 1 0 1.203705 6.607114 1.591854

91 1 0 1.378131 5.049610 2.444608

92 1 0 0.005647 5.295257 1.330958

93 1 0 5.565627 -2.464281 -2.849966

94 1 0 10.588627 0.815298 -1.069965

95 1 0 9.622231 0.449256 -2.527345

96 1 0 9.125982 1.777346 -1.429986

97 1 0 8.912088 -3.717089 -3.447353

98 1 0 7.316956 -4.003715 -2.698056

99 1 0 7.455412 -2.948080 -4.137218

100 1 0 -1.419963 1.370311 4.476546

101 1 0 -2.349934 2.859455 4.304844

102 1 0 -0.896416 2.602329 3.299737

103 1 0 2.270090 -2.421644 4.617913

104 1 0 1.334048 -0.926298 4.614406

105 1 0 0.846668 -2.270289 3.550229

106 1 0 7.826522 0.473885 1.754511

107 1 0 7.311227 2.143527 2.133464

108 1 0 6.090050 0.842155 2.008021

---------------------------------------------------------------------

DFT results for **isobejcecine** (4 conformers at 1% or greater Boltzmann weight)

*There are no imaginary frequencies in any of the conformations

(aR)-configuration calculated at cc-pVTZ / B3PW91 level

Conformer 1:

Energy = -1113.265053 Hartree

Standard orientation:

---------------------------------------------------------------------

Center Atomic Atomic Coordinates (Angstroms)

Number Number Type X Y Z

---------------------------------------------------------------------

1 6 0 -1.381739 0.980590 1.235950

2 6 0 -2.662268 1.105779 1.682159

3 6 0 -3.985230 -0.036611 -0.151348

4 6 0 -2.956701 -0.381892 -0.986235

5 6 0 -1.545431 -0.211901 -0.935464

6 6 0 -0.822921 0.392608 0.071201

7 6 0 1.326601 1.648654 -0.343609

8 6 0 0.668358 0.460514 -0.007588

9 6 0 1.433346 -0.673532 0.294203

10 6 0 2.826331 -0.636971 0.274043

11 6 0 3.476511 0.560470 -0.052429

12 6 0 2.719796 1.684944 -0.361345

13 8 0 -5.257810 -0.332145 -0.444859

14 6 0 -3.927695 0.683689 1.141361

15 8 0 -4.974192 0.917879 1.750386

16 6 0 -5.581138 -1.030839 -1.635393

17 6 0 0.555305 2.889298 -0.698588

18 8 0 0.798183 -1.857345 0.556855

19 8 0 4.829404 0.520162 -0.047945

20 6 0 5.532240 1.707660 -0.370002

21 6 0 -0.816160 -0.794632 -2.117832

22 8 0 3.547381 -1.748249 0.614638

23 6 0 0.799443 -2.257061 1.926441

24 6 0 3.996288 -2.521393 -0.496535

25 1 0 -0.639389 1.408392 1.903554

26 1 0 -2.789715 1.613584 2.632575

27 1 0 -3.274257 -0.897779 -1.883452

28 1 0 3.211415 2.610929 -0.625452

29 1 0 -6.662004 -1.144489 -1.622597

30 1 0 -5.112446 -2.017703 -1.657501

31 1 0 -5.286653 -0.464297 -2.522217

32 1 0 -0.183813 2.690128 -1.477236

33 1 0 0.007560 3.283906 0.160662

34 1 0 1.223124 3.672910 -1.055001

35 1 0 6.588458 1.459513 -0.298425

36 1 0 5.307810 2.041736 -1.386463

37 1 0 5.301564 2.510592 0.334976

38 1 0 -1.444554 -0.791603 -3.008203

39 1 0 -0.537761 -1.832209 -1.911888

40 1 0 0.101933 -0.252129 -2.335565

41 1 0 0.266242 -1.527125 2.541622

42 1 0 1.817386 -2.380155 2.298969

43 1 0 0.277539 -3.211259 1.969503

44 1 0 3.148677 -2.899838 -1.073529

45 1 0 4.556739 -3.359512 -0.085974

46 1 0 4.648797 -1.932264 -1.144903

---------------------------------------------------------------------

Conformer 2:

Energy = -1113.264389 Hartree

Standard orientation:

---------------------------------------------------------------------

Center Atomic Atomic Coordinates (Angstroms)

Number Number Type X Y Z

---------------------------------------------------------------------

1 6 0 -1.258587 0.501939 -1.413106

2 6 0 -2.494401 0.830084 -1.876551

3 6 0 -4.009187 -0.022225 -0.031419

4 6 0 -3.060595 -0.517781 0.818897

5 6 0 -1.638919 -0.604542 0.775115

6 6 0 -0.815024 -0.159267 -0.234170

7 6 0 1.272804 -1.570215 -0.462370

8 6 0 0.667018 -0.347553 -0.154353

9 6 0 1.479751 0.746418 0.171125

10 6 0 2.867692 0.632060 0.221760

11 6 0 3.463777 -0.603972 -0.061238

12 6 0 2.660993 -1.685123 -0.405979

13 8 0 -5.315688 -0.075135 0.260697

14 6 0 -3.819421 0.629849 -1.347662

15 8 0 -4.805695 1.006941 -1.983419

16 6 0 -5.758266 -0.653296 1.476798

17 6 0 0.454163 -2.768301 -0.854169

18 8 0 0.898534 1.968110 0.378638

19 8 0 4.814795 -0.641333 0.013598

20 6 0 5.463845 -1.868822 -0.268731

21 6 0 -1.059222 -1.256026 2.004423

22 8 0 3.631705 1.707399 0.582767

23 6 0 0.861027 2.407279 1.734986

24 6 0 4.189691 2.426866 -0.515606

25 1 0 -0.448534 0.796158 -2.073183

26 1 0 -2.526202 1.338847 -2.834454

27 1 0 -3.464717 -0.943250 1.729487

28 1 0 3.112562 -2.638009 -0.644858

29 1 0 -6.841844 -0.569306 1.463107

30 1 0 -5.363480 -0.113625 2.341203

31 1 0 -5.476675 -1.707138 1.542228

32 1 0 1.092560 -3.568404 -1.227737

33 1 0 -0.110005 -3.164034 -0.006012

34 1 0 -0.272196 -2.520788 -1.630201

35 1 0 6.527464 -1.681177 -0.143001

36 1 0 5.151357 -2.653381 0.425376

37 1 0 5.273559 -2.194938 -1.294660

38 1 0 -1.386179 -0.723751 2.901151

39 1 0 0.026618 -1.274419 1.993680

40 1 0 -1.422239 -2.282839 2.100029

41 1 0 0.396080 3.391377 1.726973

42 1 0 1.866093 2.481269 2.152595

43 1 0 0.256967 1.729385 2.343916

44 1 0 4.768477 3.244476 -0.089513

45 1 0 3.400598 2.834130 -1.152726

46 1 0 4.847693 1.787328 -1.108134

---------------------------------------------------------------------

Conformer 3:

Energy = -1113.263301 Hartree

Standard orientation:

---------------------------------------------------------------------

Center Atomic Atomic Coordinates (Angstroms)

Number Number Type X Y Z

---------------------------------------------------------------------

1 6 0 -1.365231 -0.438539 -1.482723

2 6 0 -2.628371 -0.373916 -1.988540

3 6 0 -4.022182 0.010876 0.090970

4 6 0 -3.026943 0.011433 1.031490

5 6 0 -1.614487 -0.138917 0.969616

6 6 0 -0.853932 -0.333457 -0.164040

7 6 0 1.267778 -1.689139 -0.109159

8 6 0 0.633017 -0.446709 -0.054925

9 6 0 1.407734 0.717730 0.068521

10 6 0 2.801134 0.651941 0.153943

11 6 0 3.423926 -0.606026 0.116556

12 6 0 2.657156 -1.754598 -0.026375

13 8 0 -5.306326 0.185097 0.428302

14 6 0 -3.914213 -0.172386 -1.374415

15 8 0 -4.937242 -0.150084 -2.063509

16 6 0 -5.676723 0.388769 1.781502

17 6 0 0.477123 -2.960615 -0.241927

18 8 0 0.728783 1.893104 0.183249

19 8 0 4.771153 -0.605074 0.265319

20 6 0 5.444905 -1.851404 0.273247

21 6 0 -0.920823 -0.040651 2.303664

22 8 0 3.516102 1.791935 0.396432

23 6 0 1.099809 2.991884 -0.644524

24 6 0 4.433906 2.197756 -0.619379

25 1 0 -0.596535 -0.595952 -2.233760

26 1 0 -2.719127 -0.488337 -3.063785

27 1 0 -3.380152 0.158537 2.044067

28 1 0 3.135647 -2.723478 -0.052132

29 1 0 -5.410989 -0.471282 2.401053

30 1 0 -6.757124 0.507361 1.773567

31 1 0 -5.215375 1.291543 2.189164

32 1 0 -0.034734 -3.018670 -1.205685

33 1 0 -0.294303 -3.033452 0.527602

34 1 0 1.126337 -3.831566 -0.157073

35 1 0 5.315913 -2.381799 -0.673795

36 1 0 5.099758 -2.485150 1.094263

37 1 0 6.498079 -1.621208 0.415466

38 1 0 -0.547024 0.975488 2.458033

39 1 0 -1.600037 -0.273860 3.122763

40 1 0 -0.062226 -0.708202 2.360556

41 1 0 1.935337 3.547300 -0.221332

42 1 0 1.355896 2.652180 -1.650840

43 1 0 0.223737 3.636341 -0.701092

44 1 0 4.867052 3.136815 -0.279594

45 1 0 5.223660 1.460638 -0.759355

46 1 0 3.914954 2.362742 -1.567434

---------------------------------------------------------------------

Conformer 4:

Energy = -1113.262739 Hartree

Standard orientation:

---------------------------------------------------------------------

Center Atomic Atomic Coordinates (Angstroms)

Number Number Type X Y Z

---------------------------------------------------------------------

1 6 0 -1.375546 -0.478059 -1.498916

2 6 0 -2.642247 -0.464789 -1.995659

3 6 0 -4.029105 0.012871 0.071516

4 6 0 -3.025062 0.097055 0.995468

5 6 0 -1.608511 -0.040186 0.932056

6 6 0 -0.853649 -0.304818 -0.187932

7 6 0 1.235339 -1.663942 0.184623

8 6 0 0.634283 -0.436205 -0.091942

9 6 0 1.437935 0.690542 -0.331160

10 6 0 2.832695 0.598964 -0.297390

11 6 0 3.423327 -0.645805 -0.023997

12 6 0 2.625515 -1.754071 0.225058

13 8 0 -5.313996 0.189186 0.408198

14 6 0 -3.929285 -0.269615 -1.379007

15 8 0 -4.958220 -0.328012 -2.055716

16 6 0 -5.673369 0.482409 1.747468

17 6 0 0.408313 -2.891335 0.446817

18 8 0 0.778987 1.831012 -0.671681

19 8 0 4.778036 -0.678559 -0.063976

20 6 0 5.424395 -1.919289 0.159475

21 6 0 -0.942156 0.151408 2.269951

22 8 0 3.593152 1.678402 -0.654818

23 6 0 1.167495 3.076284 -0.100512

24 6 0 4.433351 2.229694 0.358902

25 1 0 -0.610059 -0.659919 -2.247443

26 1 0 -2.739368 -0.635743 -3.062786

27 1 0 -3.367486 0.312829 2.000356

28 1 0 3.080400 -2.712375 0.432267

29 1 0 -6.755460 0.584946 1.743966

30 1 0 -5.221227 1.418100 2.085548

31 1 0 -5.388167 -0.327818 2.423071

32 1 0 -0.200902 -2.780328 1.347081

33 1 0 -0.280658 -3.093907 -0.375844

34 1 0 1.043724 -3.766499 0.579478

35 1 0 6.489924 -1.722001 0.069186

36 1 0 5.212531 -2.306505 1.159516

37 1 0 5.129992 -2.661362 -0.587102

38 1 0 -1.352528 -0.545943 3.004506

39 1 0 -1.138741 1.158549 2.647202

40 1 0 0.133414 0.009013 2.221131

41 1 0 0.291538 3.720388 -0.165010

42 1 0 1.994619 3.527853 -0.645662

43 1 0 1.442469 2.956313 0.949841

44 1 0 3.840140 2.568136 1.212873

45 1 0 4.932851 3.085328 -0.091974

46 1 0 5.177024 1.508028 0.694830

---------------------------------------------------------------------

DFT results for **isocolchicine monomer** (9 conformers at 1% or greater Boltzmann weight)

*There are no imaginary frequencies in any of the conformations

(aR,7S)-configuration calculated at 6-31G(d) / B3LYP level

Conformer 1:

Energy = -1359.435679 Hartree

Standard orientation:

---------------------------------------------------------------------

Center Atomic Atomic Coordinates (Angstroms)

Number Number Type X Y Z

---------------------------------------------------------------------

1 6 0 1.393461 0.158009 -0.380136

2 6 0 0.936003 1.630516 -0.334996

3 6 0 0.452498 2.126001 -1.714400

4 6 0 -0.516528 1.129130 -2.389574

5 6 0 -1.507256 0.535352 -1.410981

6 6 0 -1.046325 -0.358800 -0.422563

7 6 0 0.384557 -0.796454 -0.389367

8 6 0 0.599127 -2.211651 -0.416968

9 6 0 2.799674 -0.060181 -0.428998

10 6 0 3.575836 -1.194980 -0.479073

11 6 0 3.147538 -2.618430 -0.485183

12 6 0 1.739700 -2.963314 -0.452873

13 8 0 4.006316 -3.512123 -0.523029

14 8 0 4.924912 -1.134891 -0.527187

15 6 0 -1.974429 -0.849662 0.518930

16 6 0 -3.322455 -0.481335 0.474263

17 6 0 -3.776113 0.383477 -0.541169

18 6 0 -2.858240 0.893781 -1.465527

19 8 0 -4.191823 -0.913531 1.448024

20 8 0 -5.106723 0.666660 -0.525240

21 6 0 -4.800670 -2.181557 1.171052

22 6 0 -5.619513 1.544700 -1.523687

23 7 0 1.919122 2.558533 0.202358

24 6 0 2.065779 2.755546 1.546315

25 8 0 1.426427 2.111653 2.377390

26 6 0 3.075747 3.814515 1.953115

27 1 0 2.440619 3.141497 -0.437968

28 8 0 -1.563267 -1.718409 1.505575

29 6 0 -1.237744 -1.075750 2.748955

30 6 0 5.595651 0.122450 -0.531973

31 1 0 0.088217 1.668014 0.353009

32 1 0 1.311929 2.292718 -2.377950

33 1 0 -0.039617 3.095078 -1.566873

34 1 0 0.069578 0.323135 -2.846803

35 1 0 -1.044186 1.637564 -3.202845

36 1 0 -0.317966 -2.793107 -0.417874

37 1 0 3.359220 0.867381 -0.404890

38 1 0 1.602819 -4.042284 -0.474558

39 1 0 -3.186340 1.574203 -2.243232

40 1 0 -5.459756 -2.396153 2.015280

41 1 0 -5.391797 -2.138515 0.248675

42 1 0 -4.042865 -2.968752 1.088473

43 1 0 -5.465018 1.139519 -2.531072

44 1 0 -5.159720 2.538013 -1.456126

45 1 0 -6.689106 1.625861 -1.325098

46 1 0 3.514076 4.351158 1.107101

47 1 0 2.585088 4.533201 2.616341

48 1 0 3.879228 3.337683 2.523969

49 1 0 -2.123129 -0.593151 3.176224

50 1 0 -0.889717 -1.865432 3.418050

51 1 0 -0.441791 -0.334207 2.609244

52 1 0 5.388843 0.688651 0.383658

53 1 0 6.658675 -0.116343 -0.580391

54 1 0 5.314332 0.721472 -1.406084

---------------------------------------------------------------------

Conformer 2:

Energy = -1359.432809 Hartree

Standard orientation:

---------------------------------------------------------------------

Center Atomic Atomic Coordinates (Angstroms)

Number Number Type X Y Z

---------------------------------------------------------------------

1 6 0 1.359847 0.343168 -0.353328

2 6 0 0.805320 1.779948 -0.309356

3 6 0 0.305615 2.249572 -1.690981

4 6 0 -0.581315 1.188371 -2.380615

5 6 0 -1.540183 0.520256 -1.417575

6 6 0 -1.034481 -0.348905 -0.427921

7 6 0 0.421900 -0.683985 -0.370484

8 6 0 0.743153 -2.077523 -0.389698

9 6 0 2.770518 0.215268 -0.433577

10 6 0 3.635599 -0.857257 -0.432304

11 6 0 3.312229 -2.283257 -0.226431

12 6 0 1.939249 -2.739025 -0.339186

13 8 0 4.217308 -3.100775 0.021929

14 8 0 4.941365 -0.490248 -0.508033

15 6 0 -1.940755 -0.908850 0.496544

16 6 0 -3.309709 -0.632042 0.434542

17 6 0 -3.806161 0.207537 -0.582175

18 6 0 -2.911596 0.785403 -1.489674

19 8 0 -4.162983 -1.127118 1.392105

20 8 0 -5.152479 0.400342 -0.584412

21 6 0 -4.650509 -2.447439 1.118761

22 6 0 -5.708159 1.253470 -1.581809

23 7 0 1.719898 2.762380 0.249268

24 6 0 1.854865 2.934017 1.597404

25 8 0 1.240987 2.243575 2.410693

26 6 0 2.821012 4.021906 2.032577

27 1 0 2.234473 3.365255 -0.377849

28 8 0 -1.485878 -1.753203 1.485062

29 6 0 -1.211843 -1.094563 2.733006

30 6 0 5.906886 -1.304987 -1.198535

31 1 0 -0.051335 1.755931 0.368263

32 1 0 1.158719 2.479933 -2.343654

33 1 0 -0.255471 3.180629 -1.545605

34 1 0 0.066745 0.428740 -2.833813

35 1 0 -1.134556 1.660485 -3.198638

36 1 0 -0.125146 -2.727652 -0.440271

37 1 0 3.302452 1.161356 -0.489831

38 1 0 1.885342 -3.825723 -0.326592

39 1 0 -3.274271 1.447298 -2.267978

40 1 0 -5.308755 -2.709295 1.950183

41 1 0 -5.220336 -2.467505 0.182190

42 1 0 -3.824291 -3.164943 1.064853

43 1 0 -5.312525 2.272956 -1.500586

44 1 0 -6.782890 1.265333 -1.395227

45 1 0 -5.517019 0.867631 -2.590482

46 1 0 3.173363 4.649923 1.209521

47 1 0 2.329566 4.651269 2.779835

48 1 0 3.687320 3.555109 2.513467

49 1 0 -2.129693 -0.669980 3.153285

50 1 0 -0.820668 -1.862797 3.403141

51 1 0 -0.462979 -0.303983 2.601989

52 1 0 6.722966 -0.621211 -1.442597

53 1 0 6.258496 -2.120689 -0.569247

54 1 0 5.485746 -1.711818 -2.123079

---------------------------------------------------------------------

Conformer 3:

Energy = -1359.432793 Hartree

Standard orientation:

---------------------------------------------------------------------

Center Atomic Atomic Coordinates (Angstroms)

Number Number Type X Y Z

---------------------------------------------------------------------

1 6 0 1.356809 0.321374 -0.426574

2 6 0 0.798635 1.758638 -0.424075

3 6 0 0.266854 2.167327 -1.813936

4 6 0 -0.637149 1.079884 -2.438030

5 6 0 -1.571545 0.453545 -1.424671

6 6 0 -1.038991 -0.372265 -0.412255

7 6 0 0.418741 -0.705158 -0.384732

8 6 0 0.738878 -2.099086 -0.356541

9 6 0 2.769076 0.189299 -0.445315

10 6 0 3.631534 -0.884216 -0.496804

11 6 0 3.297478 -2.315312 -0.634374

12 6 0 1.930694 -2.764224 -0.443871

13 8 0 4.187180 -3.143121 -0.904016

14 8 0 4.939609 -0.517629 -0.535753

15 6 0 -1.919440 -0.890094 0.560179

16 6 0 -3.290249 -0.618317 0.520848

17 6 0 -3.814480 0.175745 -0.518254

18 6 0 -2.944603 0.715168 -1.472331

19 8 0 -4.118599 -1.072889 1.519667

20 8 0 -5.160960 0.365567 -0.495067

21 6 0 -4.597177 -2.410219 1.322995

22 6 0 -5.743810 1.172054 -1.515547

23 7 0 1.717756 2.770074 0.071142

24 6 0 1.852471 3.031268 1.404966

25 8 0 1.239704 2.396875 2.263515

26 6 0 2.810862 4.152547 1.766259

27 1 0 2.235945 3.327886 -0.593789

28 8 0 -1.435240 -1.687416 1.573724

29 6 0 -1.158259 -0.976580 2.792153

30 6 0 5.950526 -1.298746 0.129156

31 1 0 -0.042643 1.761490 0.272861

32 1 0 -0.290032 3.104614 -1.695267

33 1 0 1.104981 2.367835 -2.494933

34 1 0 -0.000915 0.301406 -2.875670

35 1 0 -1.209818 1.519532 -3.260740

36 1 0 -0.129009 -2.747028 -0.281692

37 1 0 3.304334 1.133433 -0.398495

38 1 0 1.872120 -3.850705 -0.449136

39 1 0 -3.328291 1.342818 -2.268778

40 1 0 -5.236689 -2.635777 2.179294

41 1 0 -3.764552 -3.121415 1.289236

42 1 0 -5.184503 -2.483187 0.399946

43 1 0 -6.814075 1.187233 -1.305089

44 1 0 -5.573305 0.743477 -2.510533

45 1 0 -5.351938 2.195849 -1.487730

46 1 0 3.235777 4.664052 0.898198

47 1 0 2.282100 4.881981 2.387199

48 1 0 3.627207 3.740106 2.367985

49 1 0 -0.741285 -1.710696 3.484646

50 1 0 -0.428420 -0.176120 2.620123

51 1 0 -2.078844 -0.555678 3.209732

52 1 0 6.777748 -0.603722 0.290026

53 1 0 5.589833 -1.662958 1.095950

54 1 0 6.265311 -2.141836 -0.483382

---------------------------------------------------------------------

Conformer 4:

Energy = -1359.432593 Hartree

Standard orientation:

---------------------------------------------------------------------

Center Atomic Atomic Coordinates (Angstroms)

Number Number Type X Y Z

---------------------------------------------------------------------

1 6 0 1.411792 0.135903 -0.396103

2 6 0 0.950252 1.605554 -0.480493

3 6 0 0.545768 1.998877 -1.917323

4 6 0 -0.382757 0.952572 -2.574299

5 6 0 -1.426182 0.426818 -1.612844

6 6 0 -1.021499 -0.394115 -0.534827

7 6 0 0.407209 -0.822539 -0.396095

8 6 0 0.623244 -2.235423 -0.320532

9 6 0 2.818733 -0.077600 -0.348759

10 6 0 3.597512 -1.209783 -0.282605

11 6 0 3.171962 -2.633866 -0.229383

12 6 0 1.764983 -2.982835 -0.250011

13 8 0 4.033001 -3.523716 -0.169250

14 8 0 4.946255 -1.146646 -0.254005

15 6 0 -1.992011 -0.820775 0.390213

16 6 0 -3.342928 -0.466718 0.245908

17 6 0 -3.733066 0.330168 -0.841078

18 6 0 -2.770035 0.776853 -1.749009

19 8 0 -4.266364 -0.866337 1.184941

20 8 0 -5.051586 0.643509 -1.066720

21 6 0 -4.765784 -2.197781 0.986979

22 6 0 -5.652132 1.535095 -0.115077

23 7 0 1.900313 2.574040 0.044354

24 6 0 1.972919 2.861856 1.378022

25 8 0 1.291956 2.269596 2.214522

26 6 0 2.952720 3.955193 1.766893

27 1 0 2.451065 3.118302 -0.605536

28 8 0 -1.631087 -1.621514 1.451284

29 6 0 -1.375309 -0.897361 2.666388

30 6 0 5.615547 0.111429 -0.285867

31 1 0 0.065002 1.687187 0.154465

32 1 0 1.441600 2.123029 -2.540685

33 1 0 0.044616 2.973163 -1.867069

34 1 0 0.228067 0.118832 -2.940227

35 1 0 -0.865147 1.400530 -3.448728

36 1 0 -0.291918 -2.819858 -0.331638

37 1 0 3.374458 0.852540 -0.352605

38 1 0 1.630678 -4.061590 -0.210816

39 1 0 -3.096337 1.400104 -2.576715

40 1 0 -5.483812 -2.376935 1.790010

41 1 0 -5.270769 -2.281838 0.017669

42 1 0 -3.954308 -2.930381 1.048557

43 1 0 -5.122299 2.495742 -0.099778

44 1 0 -5.657365 1.099455 0.887534

45 1 0 -6.677524 1.692650 -0.456124

46 1 0 3.457209 4.416931 0.913639

47 1 0 2.415288 4.728298 2.324492

48 1 0 3.707324 3.532333 2.437899

49 1 0 -2.283400 -0.392242 3.012562

50 1 0 -1.063662 -1.640179 3.403475

51 1 0 -0.573945 -0.162888 2.521262

52 1 0 5.349250 0.726299 0.581629

53 1 0 6.679471 -0.125664 -0.252138

54 1 0 5.391243 0.659630 -1.208393

---------------------------------------------------------------------

Conformer 5:

Energy = -1359.431167 Hartree

Standard orientation:

---------------------------------------------------------------------

Center Atomic Atomic Coordinates (Angstroms)

Number Number Type X Y Z

---------------------------------------------------------------------

1 6 0 1.356730 0.313723 -0.374204

2 6 0 0.732996 1.721501 -0.426587

3 6 0 0.241437 2.083467 -1.842796

4 6 0 -0.573409 0.938378 -2.485042

5 6 0 -1.519500 0.275987 -1.505436

6 6 0 -0.999358 -0.501280 -0.450580

7 6 0 0.470299 -0.757806 -0.342269

8 6 0 0.859779 -2.132495 -0.268515

9 6 0 2.773082 0.251664 -0.429389

10 6 0 3.689157 -0.774195 -0.350358

11 6 0 3.432782 -2.197716 -0.054015

12 6 0 2.085879 -2.728277 -0.156143

13 8 0 4.372894 -2.950468 0.260055

14 8 0 4.976741 -0.349444 -0.437305

15 6 0 -1.898494 -1.053705 0.487168

16 6 0 -3.282729 -0.876715 0.366475

17 6 0 -3.789101 -0.144731 -0.729550

18 6 0 -2.901768 0.441858 -1.636501

19 8 0 -4.110542 -1.483736 1.276874

20 8 0 -5.146228 -0.067195 -0.816987

21 6 0 -4.895768 -0.611740 2.102896

22 6 0 -5.714619 0.659764 -1.903713

23 7 0 1.588504 2.778380 0.088380

24 6 0 1.695083 3.030750 1.426057

25 8 0 1.101073 2.359258 2.269625

26 6 0 2.600961 4.186919 1.811889

27 1 0 2.089841 3.364748 -0.564707

28 8 0 -1.418322 -1.810513 1.531896

29 6 0 -1.082994 -1.059680 2.707366

30 6 0 5.987045 -1.159251 -1.066762

31 1 0 -0.136071 1.695495 0.235029

32 1 0 1.096724 2.319301 -2.490515

33 1 0 -0.369075 2.991222 -1.764522

34 1 0 0.123696 0.189357 -2.879437

35 1 0 -1.130835 1.331857 -3.340965

36 1 0 0.025579 -2.827068 -0.291149

37 1 0 3.258996 1.217479 -0.540475

38 1 0 2.085298 -3.813360 -0.074864

39 1 0 -3.280619 1.022932 -2.469664

40 1 0 -5.632313 -0.059222 1.513613

41 1 0 -5.406453 -1.259379 2.818736

42 1 0 -4.254866 0.093963 2.645512

43 1 0 -5.419276 1.715390 -1.876706

44 1 0 -6.795278 0.581233 -1.778191

45 1 0 -5.425955 0.224244 -2.867599

46 1 0 2.960786 4.766403 0.957191

47 1 0 2.056928 4.849556 2.491114

48 1 0 3.464480 3.793090 2.358303

49 1 0 -0.367405 -0.260726 2.479477

50 1 0 -1.980491 -0.624119 3.162295

51 1 0 -0.631284 -1.768943 3.404018

52 1 0 6.769429 -0.453388 -1.354496

53 1 0 6.375101 -1.910803 -0.381384

54 1 0 5.593662 -1.649673 -1.962478

---------------------------------------------------------------------

Conformer 6:

Energy = -1359.430575 Hartree

Standard orientation:

---------------------------------------------------------------------

Center Atomic Atomic Coordinates (Angstroms)

Number Number Type X Y Z

---------------------------------------------------------------------

1 6 0 1.387377 0.345869 -0.383414

2 6 0 0.790823 1.769024 -0.388682

3 6 0 0.270757 2.166305 -1.788295

4 6 0 -0.575461 1.057627 -2.448939

5 6 0 -1.516919 0.390426 -1.470000

6 6 0 -0.987370 -0.434877 -0.458775

7 6 0 0.481841 -0.711183 -0.396064

8 6 0 0.851423 -2.093364 -0.408139

9 6 0 2.803120 0.254883 -0.342025

10 6 0 3.703027 -0.787971 -0.375561

11 6 0 3.425754 -2.224121 -0.566415

12 6 0 2.065514 -2.719621 -0.467237

13 8 0 4.354857 -3.016447 -0.809162

14 8 0 4.998869 -0.377350 -0.343395

15 6 0 -1.874113 -1.013683 0.480638

16 6 0 -3.253208 -0.783273 0.398622

17 6 0 -3.768742 0.012837 -0.644795

18 6 0 -2.896409 0.597056 -1.566646

19 8 0 -4.111440 -1.332201 1.325888

20 8 0 -5.120809 0.168376 -0.647233

21 6 0 -4.479935 -0.433734 2.382727

22 6 0 -5.702363 0.985960 -1.659910

23 7 0 1.677638 2.811189 0.103327

24 6 0 1.713271 3.187461 1.416921

25 8 0 1.035461 2.632353 2.279617

26 6 0 2.654931 4.333143 1.747280

27 1 0 2.232593 3.328031 -0.565180

28 8 0 -1.316326 -1.726719 1.507631

29 6 0 -1.860710 -3.011491 1.847706

30 6 0 5.999693 -1.137734 0.359472

31 1 0 -0.055614 1.754927 0.302272

32 1 0 1.117224 2.406856 -2.445992

33 1 0 -0.322058 3.081611 -1.673089

34 1 0 0.098295 0.305458 -2.876384

35 1 0 -1.139171 1.486714 -3.283413

36 1 0 0.004102 -2.772572 -0.413411

37 1 0 3.305553 1.213323 -0.248958

38 1 0 2.040696 -3.806077 -0.521910

39 1 0 -3.278750 1.221862 -2.365612

40 1 0 -5.147103 -0.994361 3.041212

41 1 0 -3.596654 -0.109160 2.945642

42 1 0 -5.006639 0.440717 1.986227

43 1 0 -5.331937 2.016503 -1.603612

44 1 0 -6.776197 0.974330 -1.468409

45 1 0 -5.505624 0.581880 -2.660064

46 1 0 3.113868 4.793368 0.867807

47 1 0 2.100755 5.095568 2.302522

48 1 0 3.448247 3.960602 2.403746

49 1 0 -2.178950 -3.552281 0.949918

50 1 0 -1.046524 -3.556322 2.331557

51 1 0 -2.707863 -2.920801 2.529589

52 1 0 6.798115 -0.421845 0.567448

53 1 0 5.604507 -1.524113 1.304095

54 1 0 6.366958 -1.963867 -0.246866

---------------------------------------------------------------------

Conformer 7:

Energy = -1359.431072 Hartree

Standard orientation:

---------------------------------------------------------------------

Center Atomic Atomic Coordinates (Angstroms)

Number Number Type X Y Z

---------------------------------------------------------------------

1 6 0 1.354538 0.277775 -0.450799

2 6 0 0.723241 1.679149 -0.573184

3 6 0 0.200023 1.946501 -2.000029

4 6 0 -0.631559 0.765472 -2.550742

5 6 0 -1.552796 0.168581 -1.508087

6 6 0 -1.003042 -0.538871 -0.419926

7 6 0 0.469362 -0.789917 -0.341832

8 6 0 0.859885 -2.159077 -0.200250

9 6 0 2.771785 0.219516 -0.438942

10 6 0 3.689080 -0.807757 -0.394955

11 6 0 3.430987 -2.260657 -0.425548

12 6 0 2.085892 -2.765044 -0.218930

13 8 0 4.366524 -3.058706 -0.618565

14 8 0 4.976983 -0.376993 -0.445445

15 6 0 -1.874775 -1.026562 0.577270

16 6 0 -3.261913 -0.855733 0.483977

17 6 0 -3.799291 -0.197252 -0.643542

18 6 0 -2.938048 0.327938 -1.611238

19 8 0 -4.063105 -1.400105 1.455664

20 8 0 -5.158437 -0.125276 -0.698557

21 6 0 -4.829431 -0.475155 2.240970

22 6 0 -5.757570 0.531345 -1.813196

23 7 0 1.578519 2.774636 -0.147197

24 6 0 1.673800 3.148898 1.162707

25 8 0 1.076908 2.555006 2.060684

26 6 0 2.572786 4.339538 1.446628

27 1 0 2.081790 3.302804 -0.846937

28 8 0 -1.364468 -1.713232 1.656029

29 6 0 -1.004464 -0.886732 2.771751

30 6 0 6.017141 -1.052058 0.287324

31 1 0 -0.131166 1.693297 0.107586

32 1 0 -0.407763 2.858830 -1.966944

33 1 0 1.041014 2.137747 -2.680157

34 1 0 -1.209429 1.107476 -3.415239

35 1 0 0.054041 -0.008690 -2.915511

36 1 0 0.025198 -2.843847 -0.086834

37 1 0 3.256747 1.191434 -0.458932

38 1 0 2.083562 -3.850362 -0.141189

39 1 0 -3.340129 0.854101 -2.469570

40 1 0 -4.177259 0.268426 2.715337

41 1 0 -5.584541 0.032965 1.635561

42 1 0 -5.316868 -1.073212 3.013868

43 1 0 -5.495002 0.035422 -2.755071

44 1 0 -5.463057 1.586456 -1.861242

45 1 0 -6.834242 0.461297 -1.653187

46 1 0 3.435919 4.001083 2.029551

47 1 0 2.933323 4.841493 0.544506

48 1 0 2.022659 5.057819 2.061289

49 1 0 -0.295014 -0.104729 2.475789

50 1 0 -1.892177 -0.421948 3.216711

51 1 0 -0.536727 -1.548621 3.503617

52 1 0 6.804963 -0.304370 0.403704

53 1 0 5.661944 -1.361259 1.275065

54 1 0 6.383554 -1.920995 -0.256442

---------------------------------------------------------------------

Conformer 8:

Energy = -1359.429694 Hartree

Standard orientation:

---------------------------------------------------------------------

Center Atomic Atomic Coordinates (Angstroms)

Number Number Type X Y Z

---------------------------------------------------------------------

1 6 0 1.377192 0.330166 -0.378044

2 6 0 0.818431 1.764736 -0.443143

3 6 0 0.401903 2.156711 -1.875612

4 6 0 -0.443526 1.057209 -2.557851

5 6 0 -1.455603 0.439273 -1.616908

6 6 0 -1.008016 -0.375335 -0.550553

7 6 0 0.444690 -0.701279 -0.395236

8 6 0 0.767742 -2.092566 -0.327386

9 6 0 2.790414 0.205659 -0.372885

10 6 0 3.655678 -0.861784 -0.270062

11 6 0 3.322945 -2.276635 -0.005539

12 6 0 1.960273 -2.744918 -0.175311

13 8 0 4.213530 -3.074077 0.339926

14 8 0 4.962319 -0.494492 -0.297878

15 6 0 -1.956906 -0.887724 0.353666

16 6 0 -3.327837 -0.627367 0.198537

17 6 0 -3.759287 0.164716 -0.876571

18 6 0 -2.819184 0.696885 -1.762335

19 8 0 -4.235997 -1.104035 1.115526

20 8 0 -5.093053 0.392686 -1.112030

21 6 0 -4.578881 -2.488260 0.946179

22 6 0 -5.762819 1.221167 -0.149555

23 7 0 1.696820 2.778516 0.116959

24 6 0 1.750960 3.018421 1.460395

25 8 0 1.089164 2.367954 2.269186

26 6 0 2.688522 4.130195 1.897208

27 1 0 2.248864 3.349281 -0.508365

28 8 0 -1.551382 -1.676282 1.407848

29 6 0 -1.356933 -0.951253 2.634470

30 6 0 5.966383 -1.344773 -0.883163

31 1 0 -0.076919 1.773396 0.182749

32 1 0 -0.166615 3.092516 -1.814661

33 1 0 1.292431 2.353843 -2.487621

34 1 0 0.229189 0.276570 -2.932410

35 1 0 -0.950372 1.483523 -3.429175

36 1 0 -0.095010 -2.748494 -0.395309

37 1 0 3.322653 1.150089 -0.450064

38 1 0 1.907975 -3.829850 -0.111474

39 1 0 -3.178984 1.313593 -2.581020

40 1 0 -5.307002 -2.718776 1.726677

41 1 0 -5.030830 -2.657406 -0.038202

42 1 0 -3.696445 -3.124655 1.065775

43 1 0 -5.749814 0.764078 0.843389

44 1 0 -6.792591 1.317087 -0.500122

45 1 0 -5.298824 2.214504 -0.107345

46 1 0 3.098633 4.709567 1.065386

47 1 0 2.149076 4.801806 2.571082

48 1 0 3.517841 3.692617 2.463186

49 1 0 -0.606583 -0.161816 2.506095

50 1 0 -2.300817 -0.513851 2.977019

51 1 0 -1.001983 -1.680382 3.365519

52 1 0 6.791506 -0.671803 -1.126558

53 1 0 6.286861 -2.116075 -0.185057

54 1 0 5.596518 -1.812485 -1.800650

---------------------------------------------------------------------

Conformer 9:

Energy = -1359.430543 Hartree

Standard orientation:

---------------------------------------------------------------------

Center Atomic Atomic Coordinates (Angstroms)

Number Number Type X Y Z

---------------------------------------------------------------------

1 6 0 -1.451771 0.135005 0.365205

2 6 0 -1.041560 1.620528 0.462421

3 6 0 -0.621037 2.008714 1.897634

4 6 0 0.339821 0.984057 2.537029

5 6 0 1.389240 0.498686 1.562282

6 6 0 1.001589 -0.333790 0.489286

7 6 0 -0.418883 -0.794066 0.362193

8 6 0 -0.600591 -2.212463 0.303222

9 6 0 -2.851505 -0.123018 0.311524

10 6 0 -3.598506 -1.275574 0.232606

11 6 0 -3.134263 -2.686337 0.182590

12 6 0 -1.718525 -2.994456 0.230580

13 8 0 -3.968131 -3.600846 0.108427

14 8 0 -4.948643 -1.248553 0.190402

15 6 0 1.974459 -0.736657 -0.451729

16 6 0 3.311438 -0.321958 -0.319785

17 6 0 3.687881 0.461300 0.783582

18 6 0 2.726349 0.869375 1.707722

19 8 0 4.241580 -0.666480 -1.278027

20 8 0 4.975850 0.918723 0.928111

21 6 0 4.484418 0.369418 -2.244653

22 6 0 5.972022 -0.088426 1.166383

23 7 0 -2.040255 2.566938 -0.011373

24 6 0 -2.114719 2.959818 -1.318975

25 8 0 -1.390936 2.482162 -2.190073

26 6 0 -3.157303 4.020377 -1.629503

27 1 0 -2.622537 3.034872 0.669761

28 8 0 1.547803 -1.447673 -1.543772

29 6 0 2.214620 -2.680991 -1.857344

30 6 0 -5.651240 -0.008764 0.217409

31 1 0 -0.178359 1.747051 -0.195647

32 1 0 -1.511006 2.106683 2.534266

33 1 0 -0.145967 2.995853 1.847567

34 1 0 -0.243979 0.129987 2.900218

35 1 0 0.816749 1.437342 3.411804

36 1 0 0.328383 -2.774056 0.345621

37 1 0 -3.434770 0.789837 0.315462

38 1 0 -1.552309 -4.069261 0.209512

39 1 0 3.042132 1.492175 2.539494

40 1 0 4.881738 1.267131 -1.759732

41 1 0 5.224069 -0.031167 -2.940934

42 1 0 3.564469 0.616445 -2.786729

43 1 0 6.917528 0.445820 1.279634

44 1 0 6.037869 -0.787143 0.328358

45 1 0 5.750067 -0.636033 2.090529

46 1 0 -2.665451 4.864586 -2.122193

47 1 0 -3.883163 3.606167 -2.336741

48 1 0 -3.691852 4.384173 -0.747539

49 1 0 3.205213 -2.504620 -2.279028

50 1 0 2.307309 -3.310646 -0.964412

51 1 0 1.575436 -3.181612 -2.587451

52 1 0 -6.708087 -0.273696 0.170274

53 1 0 -5.452673 0.542643 1.143976

54 1 0 -5.390801 0.615581 -0.645093

---------------------------------------------------------------------

DFT results for **isocolchicine dimer** (7 conformers at 1% or greater Boltzmann weight)

*There are no imaginary frequencies in any of the conformations

(aR,7S)-configuration calculated at 6-31G(d) / B3LYP level

Conformer 1:

Energy = -2718.881528 Hartree

Standard orientation:

---------------------------------------------------------------------

Center Atomic Atomic Coordinates (Angstroms)

Number Number Type X Y Z

---------------------------------------------------------------------

1 6 0 2.575344 -0.872960 0.045584

2 6 0 2.869667 -0.695679 -1.459508

3 6 0 2.123885 0.520706 -2.059935

4 6 0 2.221749 1.793771 -1.193034

5 6 0 3.605972 1.993828 -0.619040

6 6 0 4.067322 1.106393 0.373323

7 6 0 3.173923 0.036499 0.916516

8 6 0 2.975747 0.069035 2.332886

9 6 0 1.784674 -1.990552 0.419719

10 6 0 1.215888 -2.383960 1.609330

11 6 0 1.097615 -1.597050 2.843139

12 6 0 2.099104 -0.600583 3.145861

13 8 0 0.163155 -1.825678 3.647820

14 8 0 0.526347 -3.559439 1.547323

15 6 0 5.378960 1.270132 0.864104

16 6 0 6.208364 2.292105 0.392435

17 6 0 5.723906 3.192037 -0.577662

18 6 0 4.429425 3.024942 -1.081561

19 8 0 7.507744 2.387510 0.832512

20 8 0 6.587817 4.171113 -0.958164

21 6 0 7.670993 3.188424 2.010489

22 6 0 6.157135 5.100047 -1.950019

23 7 0 2.604670 -1.863496 -2.282148

24 6 0 3.563829 -2.805708 -2.506091

25 8 0 4.677972 -2.760195 -1.979577

26 6 0 3.166429 -3.933124 -3.443552

27 1 0 1.697281 -1.949673 -2.741781

28 8 0 5.858421 0.420545 1.836353

29 6 0 6.681843 -0.643693 1.330809

30 6 0 0.692765 -4.497510 2.626202

31 6 0 -2.575354 -0.872966 -0.045590

32 6 0 -2.869659 -0.695645 1.459502

33 6 0 -2.123857 0.520746 2.059892

34 6 0 -2.221719 1.793791 1.192963

35 6 0 -3.605948 1.993846 0.618983

36 6 0 -4.067317 1.106393 -0.373356

37 6 0 -3.173930 0.036482 -0.916536

38 6 0 -2.975745 0.068999 -2.332907

39 6 0 -1.784684 -1.990565 -0.419707

40 6 0 -1.215895 -2.383987 -1.609312

41 6 0 -1.097615 -1.597098 -2.843131

42 6 0 -2.099102 -0.600632 -3.145870

43 8 0 -0.163153 -1.825746 -3.647803

44 8 0 -0.526378 -3.559479 -1.547310

45 6 0 -5.378968 1.270122 -0.864108

46 6 0 -6.208359 2.292108 -0.392445

47 6 0 -5.723877 3.192063 0.577619

48 6 0 -4.429386 3.024975 1.081496

49 8 0 -7.507750 2.387503 -0.832492

50 8 0 -6.587779 4.171147 0.958119

51 6 0 -7.671027 3.188389 -2.010484

52 6 0 -6.157078 5.100103 1.949944

53 7 0 -2.604669 -1.863444 2.282170

54 6 0 -3.563839 -2.805635 2.506152

55 8 0 -4.677987 -2.760121 1.979651

56 6 0 -3.166442 -3.933030 3.443640

57 1 0 -1.697276 -1.949621 2.741797

58 8 0 -5.858466 0.420502 -1.836310

59 6 0 -6.681884 -0.643710 -1.330702

60 6 0 -0.692786 -4.497510 -2.626223

61 1 0 3.943582 -0.508814 -1.543018

62 1 0 2.556870 0.704380 -3.050356

63 1 0 1.067277 0.274070 -2.213591

64 1 0 1.495168 1.723771 -0.374128

65 1 0 1.931884 2.659564 -1.797184

66 1 0 3.579274 0.813223 2.843386

67 1 0 1.571149 -2.684095 -0.388412

68 1 0 2.073472 -0.292670 4.189193

69 1 0 4.045219 3.700140 -1.837607

70 1 0 8.737989 3.176668 2.243835

71 1 0 7.107391 2.766120 2.850082

72 1 0 7.348440 4.220355 1.829344

73 1 0 6.994297 5.783567 -2.098029

74 1 0 5.279118 5.665115 -1.614688

75 1 0 5.926479 4.595336 -2.895732

76 1 0 2.228447 -3.742734 -3.971319

77 1 0 3.968203 -4.085352 -4.171743

78 1 0 3.065936 -4.859371 -2.867012

79 1 0 6.953239 -1.254732 2.194138

80 1 0 6.127551 -1.254050 0.607599

81 1 0 7.586018 -0.242340 0.862389

82 1 0 0.109677 -5.373400 2.338689

83 1 0 1.748626 -4.775413 2.720389

84 1 0 0.327457 -4.089298 3.569137

85 1 0 -3.943571 -0.508766 1.543018

86 1 0 -2.556826 0.704447 3.050316

87 1 0 -1.067248 0.274102 2.213540

88 1 0 -1.495150 1.723763 0.374048

89 1 0 -1.931838 2.659595 1.797087

90 1 0 -3.579262 0.813187 -2.843419

91 1 0 -1.571170 -2.684103 0.388432

92 1 0 -2.073461 -0.292729 -4.189205

93 1 0 -4.045164 3.700186 1.837520

94 1 0 -8.738029 3.176628 -2.243804

95 1 0 -7.107447 2.766064 -2.850081

96 1 0 -7.348469 4.220324 -1.829372

97 1 0 -6.994239 5.783624 2.097957

98 1 0 -5.279070 5.665166 1.614583

99 1 0 -5.926401 4.595412 2.895663

100 1 0 -2.228463 -3.742626 3.971407

101 1 0 -3.968219 -4.085243 4.171832

102 1 0 -3.065944 -4.859290 2.867123

103 1 0 -6.953308 -1.254781 -2.193998

104 1 0 -6.127576 -1.254041 -0.607482

105 1 0 -7.586042 -0.242331 -0.862272

106 1 0 -0.109742 -5.373431 -2.338715

107 1 0 -1.748653 -4.775370 -2.720466

108 1 0 -0.327424 -4.089278 -3.569130

---------------------------------------------------------------------

Conformer 2:

Energy = -2718.880504 Hartree

Standard orientation:

---------------------------------------------------------------------

Center Atomic Atomic Coordinates (Angstroms)

Number Number Type X Y Z

---------------------------------------------------------------------

1 6 0 -2.841940 -1.391471 0.179876

2 6 0 -3.663436 -1.092173 -1.074736

3 6 0 -5.168023 -1.329296 -0.800552

4 6 0 -5.613007 -0.642161 0.510985

5 6 0 -4.988878 0.726197 0.709124

6 6 0 -3.608354 0.839814 0.993861

7 6 0 -2.760613 -0.382688 1.141374

8 6 0 -1.954881 -0.459620 2.317086

9 6 0 -2.350304 -2.720008 0.301070

10 6 0 -1.467369 -3.318385 1.175048

11 6 0 -0.611502 -2.646579 2.175082

12 6 0 -1.026468 -1.381831 2.728295

13 8 0 0.416700 -3.226280 2.591874

14 8 0 -1.209580 -4.638700 1.140922

15 6 0 -3.068431 2.133221 1.165388

16 6 0 -3.860752 3.278227 1.039530

17 6 0 -5.236263 3.149285 0.767162

18 6 0 -5.782775 1.872358 0.602246

19 8 0 -3.289372 4.525932 1.130441

20 8 0 -5.933898 4.313354 0.682609

21 6 0 -3.265590 5.065316 2.458437

22 6 0 -7.328300 4.243369 0.393304

23 7 0 -3.187689 -1.825687 -2.254025

24 6 0 -3.591375 -1.458243 -3.506125

25 8 0 -4.461785 -0.608240 -3.708374

26 6 0 -2.921567 -2.204042 -4.649494

27 1 0 -2.309197 -2.340259 -2.198518

28 8 0 -1.741047 2.296233 1.493006

29 6 0 -0.873126 2.545151 0.378103

30 6 0 -1.903887 -5.483310 0.216148

31 6 0 2.769206 -1.443812 -0.114902

32 6 0 3.604648 -1.104617 1.119243

33 6 0 5.097062 -1.418819 0.853163

34 6 0 5.556622 -0.837228 -0.503046

35 6 0 4.992901 0.543725 -0.780288

36 6 0 3.617719 0.705435 -1.063339

37 6 0 2.721371 -0.490225 -1.133346

38 6 0 1.914368 -0.616167 -2.302821

39 6 0 2.238699 -2.762360 -0.160022

40 6 0 1.340311 -3.386777 -0.998958

41 6 0 0.508177 -2.752488 -2.041524

42 6 0 0.959483 -1.532455 -2.663481

43 8 0 -0.529802 -3.331079 -2.433996

44 8 0 1.045250 -4.694976 -0.887560

45 6 0 3.125945 2.012802 -1.293768

46 6 0 3.977343 3.123423 -1.235161

47 6 0 5.351310 2.939454 -0.982076

48 6 0 5.844140 1.652005 -0.755902

49 8 0 3.482137 4.392518 -1.436816

50 8 0 6.100753 4.074409 -0.960380

51 6 0 3.245238 5.130041 -0.228424

52 6 0 7.492341 3.958047 -0.670538

53 7 0 3.105102 -1.750313 2.339061

54 6 0 3.525218 -1.326757 3.567254

55 8 0 4.424550 -0.496410 3.718708

56 6 0 2.833438 -1.982307 4.751983

57 1 0 2.216167 -2.248905 2.312402

58 8 0 1.775000 2.169809 -1.488455

59 6 0 1.343820 2.855169 -2.676865

60 6 0 1.717740 -5.503235 0.085105

61 1 0 -3.552713 -0.025028 -1.293810

62 1 0 -5.723050 -0.935989 -1.655665

63 1 0 -5.367067 -2.406163 -0.737615

64 1 0 -5.339944 -1.283860 1.357779

65 1 0 -6.704378 -0.556017 0.521422

66 1 0 -2.054865 0.394734 2.977944

67 1 0 -2.733786 -3.386200 -0.462471

68 1 0 -0.479872 -1.137265 3.636545

69 1 0 -6.839489 1.754898 0.391195

70 1 0 -2.679044 4.426232 3.128209

71 1 0 -4.281972 5.180627 2.852645

72 1 0 -2.791869 6.046683 2.382524

73 1 0 -7.676417 5.276833 0.366622

74 1 0 -7.871653 3.695098 1.172237

75 1 0 -7.510229 3.771451 -0.579508

76 1 0 -3.633910 -2.925007 -5.067401

77 1 0 -2.678566 -1.488742 -5.440503

78 1 0 -2.019450 -2.736472 -4.339481

79 1 0 0.131573 2.657329 0.787308

80 1 0 -0.882740 1.700513 -0.320307

81 1 0 -1.165926 3.463091 -0.142227

82 1 0 -2.987938 -5.417018 0.361702

83 1 0 -1.634371 -5.222651 -0.811114

84 1 0 -1.566668 -6.494815 0.444548

85 1 0 3.537727 -0.023451 1.277665

86 1 0 5.674356 -0.993821 1.677936

87 1 0 5.252377 -2.504684 0.859056

88 1 0 5.246001 -1.518293 -1.304829

89 1 0 6.650494 -0.800943 -0.528857

90 1 0 2.053002 0.182056 -3.024603

91 1 0 2.603194 -3.393418 0.641595

92 1 0 0.425812 -1.327575 -3.589037

93 1 0 6.897315 1.497141 -0.552408

94 1 0 4.176863 5.281317 0.326503

95 1 0 2.515767 4.613969 0.406947

96 1 0 2.841608 6.097080 -0.536171

97 1 0 8.006069 3.347196 -1.422177

98 1 0 7.658161 3.528858 0.324646

99 1 0 7.885392 4.975147 -0.698740

100 1 0 2.594492 -1.211132 5.490094

101 1 0 1.925407 -2.520507 4.470384

102 1 0 3.530658 -2.683422 5.225222

103 1 0 1.849000 2.450724 -3.562142

104 1 0 0.271547 2.664789 -2.753597

105 1 0 1.535518 3.926789 -2.610157

106 1 0 1.351299 -6.516366 -0.083141

107 1 0 2.802821 -5.476560 -0.065683

108 1 0 1.457817 -5.174451 1.094963

---------------------------------------------------------------------

Conformer 3:

Energy = -2718.878457 Hartree

Standard orientation:

---------------------------------------------------------------------

Center Atomic Atomic Coordinates (Angstroms)

Number Number Type X Y Z

---------------------------------------------------------------------

1 6 0 2.554198 -0.455284 0.085304

2 6 0 3.089416 -1.076417 -1.222904

3 6 0 3.462587 0.007660 -2.261210

4 6 0 4.287306 1.166561 -1.660501

5 6 0 5.339334 0.680048 -0.688710

6 6 0 4.932852 0.142527 0.549705

7 6 0 3.489153 0.128281 0.938055

8 6 0 3.177440 0.771338 2.176851

9 6 0 1.163273 -0.583676 0.329051

10 6 0 0.315499 -0.091636 1.295114

11 6 0 0.616385 0.883118 2.349621

12 6 0 1.983143 1.094479 2.766604

13 8 0 -0.316889 1.504716 2.914300

14 8 0 -0.987851 -0.456489 1.131242

15 6 0 5.920873 -0.359454 1.421316

16 6 0 7.276838 -0.317307 1.084194

17 6 0 7.672785 0.251510 -0.142767

18 6 0 6.696344 0.733879 -1.021381

19 8 0 8.217309 -0.878130 1.915972

20 8 0 9.011685 0.273050 -0.378610

21 6 0 8.692438 0.001042 2.944082

22 6 0 9.468607 0.817240 -1.614480

23 7 0 2.204381 -2.043128 -1.844764

24 6 0 2.269558 -3.371758 -1.546461

25 8 0 3.058754 -3.831737 -0.717465

26 6 0 1.308741 -4.257453 -2.322014

27 1 0 1.506409 -1.714329 -2.513573

28 8 0 5.555878 -0.897017 2.635971

29 6 0 5.465433 -2.331788 2.636788

30 6 0 -1.705330 -0.957462 2.274219

31 6 0 -2.861371 1.153331 -0.969008

32 6 0 -3.241489 2.152361 0.140699

33 6 0 -4.338223 3.144146 -0.312826

34 6 0 -5.451608 2.428261 -1.102723

35 6 0 -5.895937 1.130798 -0.463640

36 6 0 -5.036566 0.008371 -0.464964

37 6 0 -3.717864 0.072916 -1.177651

38 6 0 -3.460135 -0.981357 -2.107584

39 6 0 -1.689772 1.474597 -1.709399

40 6 0 -0.919755 0.759693 -2.597608

41 6 0 -1.067658 -0.656866 -2.986507

42 6 0 -2.349552 -1.301885 -2.843483

43 8 0 -0.110794 -1.257519 -3.523691

44 8 0 0.167037 1.298522 -3.197432

45 6 0 -5.474064 -1.190200 0.159480

46 6 0 -6.759072 -1.267329 0.729023

47 6 0 -7.580234 -0.127469 0.742793

48 6 0 -7.145967 1.055633 0.153460

49 8 0 -7.221509 -2.448180 1.274717

50 8 0 -8.851664 -0.181075 1.263765

51 6 0 -8.105262 -3.178381 0.408929

52 6 0 -8.929560 -0.345269 2.688509

53 7 0 -2.106273 2.812059 0.796967

54 6 0 -1.488595 3.967995 0.426337

55 8 0 -1.724142 4.573819 -0.625751

56 6 0 -0.466066 4.502341 1.418223

57 1 0 -1.702899 2.315240 1.591255

58 8 0 -4.604478 -2.243069 0.130206

59 6 0 -4.580772 -3.231299 1.170209

60 6 0 0.456008 2.689217 -3.038042

61 1 0 3.995987 -1.625835 -0.957182

62 1 0 2.549206 0.408704 -2.716422

63 1 0 4.031849 -0.489314 -3.055828

64 1 0 3.608724 1.856985 -1.144618

65 1 0 4.754107 1.732815 -2.472991

66 1 0 4.050320 1.117080 2.722004

67 1 0 0.641124 -1.205674 -0.391473

68 1 0 2.042490 1.677400 3.683399

69 1 0 6.983290 1.161586 -1.975228

70 1 0 9.429963 -0.566634 3.515842

71 1 0 7.873640 0.307813 3.604393

72 1 0 9.170801 0.887189 2.510491

73 1 0 10.555851 0.731915 -1.590754

74 1 0 9.076516 0.251751 -2.468194

75 1 0 9.187089 1.872284 -1.715645

76 1 0 0.610912 -3.688011 -2.940892

77 1 0 1.887379 -4.934099 -2.960393

78 1 0 0.748871 -4.876510 -1.614188

79 1 0 6.443810 -2.779650 2.435795

80 1 0 5.127709 -2.614684 3.635885

81 1 0 4.738825 -2.676522 1.891035

82 1 0 -1.222098 -1.866930 2.649733

83 1 0 -2.698239 -1.199473 1.896278

84 1 0 -1.766737 -0.209556 3.065066

85 1 0 -3.687935 1.545548 0.933603

86 1 0 -4.749602 3.617898 0.587218

87 1 0 -3.900287 3.930410 -0.929161

88 1 0 -5.086889 2.226252 -2.117349

89 1 0 -6.307981 3.102682 -1.203452

90 1 0 -4.275928 -1.689746 -2.210679

91 1 0 -1.329349 2.473795 -1.502518

92 1 0 -2.400834 -2.226068 -3.415277

93 1 0 -7.809647 1.914929 0.166373

94 1 0 -8.990315 -2.582790 0.164033

95 1 0 -7.590466 -3.473090 -0.513571

96 1 0 -8.403939 -4.071414 0.962092

97 1 0 -9.993647 -0.346805 2.933382

98 1 0 -8.474593 -1.287965 3.004251

99 1 0 -8.439517 0.493361 3.198138

100 1 0 0.448412 4.762462 0.876815

101 1 0 -0.233019 3.801388 2.222078

102 1 0 -0.860738 5.427492 1.853731

103 1 0 -5.324206 -4.012611 1.001451

104 1 0 -4.747773 -2.779667 2.152044

105 1 0 -3.577645 -3.662041 1.128113

106 1 0 0.739068 2.920128 -2.005295

107 1 0 1.298316 2.886512 -3.701710

108 1 0 -0.399978 3.307830 -3.327542

---------------------------------------------------------------------

Conformer 4:

Energy = -2718.878352 Hartree

Standard orientation:

---------------------------------------------------------------------

Center Atomic Atomic Coordinates (Angstroms)

Number Number Type X Y Z

---------------------------------------------------------------------

1 6 0 -2.610740 -0.847876 -0.035300

2 6 0 -2.910535 -0.633486 1.463868

3 6 0 -2.179098 0.606967 2.031641

4 6 0 -2.293168 1.855225 1.130901

5 6 0 -3.679486 2.020923 0.550881

6 6 0 -4.127415 1.101711 -0.418366

7 6 0 -3.218555 0.030093 -0.931551

8 6 0 -3.016587 0.028017 -2.347816

9 6 0 -1.805026 -1.965053 -0.377504

10 6 0 -1.227511 -2.381674 -1.554755

11 6 0 -1.114476 -1.625283 -2.808000

12 6 0 -2.127805 -0.650245 -3.140104

13 8 0 -0.174504 -1.863292 -3.603465

14 8 0 -0.523687 -3.546932 -1.461594

15 6 0 -5.441024 1.233203 -0.913470

16 6 0 -6.285714 2.254378 -0.467762

17 6 0 -5.814932 3.186077 0.478770

18 6 0 -4.518170 3.051493 0.986492

19 8 0 -7.586425 2.318246 -0.909552

20 8 0 -6.693708 4.161239 0.834442

21 6 0 -7.764023 3.094513 -2.101888

22 6 0 -6.277820 5.120962 1.803093

23 7 0 -2.635554 -1.776210 2.317870

24 6 0 -3.585801 -2.722024 2.563708

25 8 0 -4.698768 -2.701373 2.033252

26 6 0 -3.179882 -3.821243 3.530587

27 1 0 -1.728676 -1.841091 2.781739

28 8 0 -5.906873 0.351520 -1.863383

29 6 0 -6.717563 -0.709558 -1.331407

30 6 0 -0.684360 -4.516183 -2.513460

31 6 0 2.556356 -0.817097 0.066433

32 6 0 2.852944 -0.676056 -1.442057

33 6 0 2.107702 0.526604 -2.071138

34 6 0 2.210490 1.822637 -1.238777

35 6 0 3.595193 2.030683 -0.670176

36 6 0 4.053485 1.164896 0.347424

37 6 0 3.150533 0.114412 0.915994

38 6 0 2.943033 0.189786 2.328827

39 6 0 1.767920 -1.927007 0.467390

40 6 0 1.197467 -2.290172 1.665761

41 6 0 1.068321 -1.467916 2.876138

42 6 0 2.062803 -0.458069 3.155570

43 8 0 0.130471 -1.678187 3.681401

44 8 0 0.516919 -3.471185 1.634577

45 6 0 5.362973 1.323832 0.836119

46 6 0 6.204304 2.334093 0.343480

47 6 0 5.731620 3.196097 -0.658118

48 6 0 4.438266 3.029067 -1.158886

49 8 0 7.492413 2.454220 0.813672

50 8 0 6.491604 4.241159 -1.122944

51 6 0 7.600049 3.168903 2.054623

52 6 0 7.651362 3.876925 -1.887512

53 7 0 2.588454 -1.862758 -2.236954

54 6 0 3.551168 -2.805145 -2.444744

55 8 0 4.666492 -2.744191 -1.922132

56 6 0 3.156645 -3.952127 -3.359211

57 1 0 1.680449 -1.961014 -2.693353

58 8 0 5.828293 0.497051 1.834761

59 6 0 6.664155 -0.575039 1.365747

60 6 0 0.678244 -4.375179 2.743207

61 1 0 -3.986485 -0.455852 1.540276

62 1 0 -2.613799 0.811481 3.017203

63 1 0 -1.119426 0.377320 2.190569

64 1 0 -1.564724 1.773315 0.314809

65 1 0 -2.015790 2.740636 1.712026

66 1 0 -3.628729 0.750166 -2.879329

67 1 0 -1.586281 -2.634773 0.449123

68 1 0 -2.102703 -0.369541 -4.191086

69 1 0 -4.144299 3.751778 1.724669

70 1 0 -8.830514 3.058645 -2.335063

71 1 0 -7.460789 4.135368 -1.939616

72 1 0 -7.192339 2.667580 -2.933685

73 1 0 -6.038808 4.643077 2.760579

74 1 0 -7.125895 5.794238 1.934990

75 1 0 -5.409175 5.691805 1.453455

76 1 0 -2.249100 -3.604953 4.061149

77 1 0 -3.985372 -3.968161 4.255697

78 1 0 -3.061058 -4.759141 2.976772

79 1 0 -6.977021 -1.348078 -2.178373

80 1 0 -6.157790 -1.291827 -0.589482

81 1 0 -7.629078 -0.308033 -0.877623

82 1 0 -1.735922 -4.815471 -2.588324

83 1 0 -0.082101 -5.373510 -2.210006

84 1 0 -0.336531 -4.126500 -3.470822

85 1 0 3.927065 -0.492586 -1.529683

86 1 0 2.539103 0.683685 -3.066752

87 1 0 1.050421 0.277677 -2.216315

88 1 0 1.482915 1.779325 -0.418939

89 1 0 1.927071 2.673284 -1.866764

90 1 0 3.541412 0.950404 2.820926

91 1 0 1.560016 -2.642934 -0.322497

92 1 0 2.029157 -0.119672 4.189143

93 1 0 4.097650 3.706743 -1.936456

94 1 0 7.050049 2.653287 2.848610

95 1 0 7.224396 4.192921 1.945584

96 1 0 8.663641 3.193916 2.300721

97 1 0 8.118102 4.816269 -2.191185

98 1 0 8.352612 3.290693 -1.287879

99 1 0 7.360993 3.309454 -2.780385

100 1 0 3.954703 -4.110565 -4.090206

101 1 0 2.213742 -3.778385 -3.883837

102 1 0 3.067852 -4.868897 -2.765906

103 1 0 7.571884 -0.180572 0.898352

104 1 0 6.928055 -1.162553 2.247375

105 1 0 6.121319 -1.205571 0.651398

106 1 0 0.105189 -5.263766 2.475361

107 1 0 0.299366 -3.941896 3.669349

108 1 0 1.735068 -4.641548 2.857088

---------------------------------------------------------------------

Conformer 5:

Energy = -2718.878288 Hartree

Standard orientation:

---------------------------------------------------------------------

Center Atomic Atomic Coordinates (Angstroms)

Number Number Type X Y Z

---------------------------------------------------------------------

1 6 0 -2.347582 -0.370868 -0.135345

2 6 0 -2.782761 -0.816866 1.277333

3 6 0 -3.207935 0.379635 2.163900

4 6 0 -4.144425 1.378772 1.452310

5 6 0 -5.192513 0.681254 0.615844

6 6 0 -4.786306 -0.003341 -0.545953

7 6 0 -3.361789 0.036421 -1.001131

8 6 0 -3.162126 0.542368 -2.323175

9 6 0 -0.970693 -0.495753 -0.460962

10 6 0 -0.223252 -0.108860 -1.549270

11 6 0 -0.631836 0.781291 -2.642844

12 6 0 -2.024306 0.876156 -3.008979

13 8 0 0.239181 1.411452 -3.289877

14 8 0 1.096668 -0.462974 -1.501403

15 6 0 -5.757059 -0.706376 -1.287772

16 6 0 -7.098949 -0.721514 -0.896375

17 6 0 -7.498988 -0.003939 0.248872

18 6 0 -6.536905 0.680676 1.000030

19 8 0 -8.015418 -1.476037 -1.590366

20 8 0 -8.826359 -0.050535 0.540282

21 6 0 -8.609658 -0.799304 -2.706094

22 6 0 -9.286773 0.642370 1.697964

23 7 0 -1.801138 -1.598832 2.006532

24 6 0 -1.732342 -2.954951 1.890724

25 8 0 -2.428422 -3.590908 1.095943

26 6 0 -0.731171 -3.633018 2.811136

27 1 0 -1.221722 -1.125588 2.700008

28 8 0 -5.390173 -1.388314 -2.427241

29 6 0 -5.154329 -2.790696 -2.217321

30 6 0 1.683775 -0.997665 -2.707620

31 6 0 2.844403 1.464764 0.856945

32 6 0 3.212362 2.122563 -0.491766

33 6 0 4.433549 3.068564 -0.397932

34 6 0 5.584356 2.458379 0.421898

35 6 0 5.821362 0.997248 0.117347

36 6 0 4.888816 0.030787 0.562947

37 6 0 3.660729 0.431596 1.320872

38 6 0 3.429229 -0.293470 2.533049

39 6 0 1.702828 2.003014 1.516797

40 6 0 0.919481 1.540933 2.549424

41 6 0 1.040498 0.257863 3.271387

42 6 0 2.331158 -0.380786 3.345338

43 8 0 0.061398 -0.205528 3.894538

44 8 0 -0.156015 2.228098 2.997825

45 6 0 5.166614 -1.331381 0.310232

46 6 0 6.302744 -1.720247 -0.417251

47 6 0 7.198689 -0.744751 -0.878544

48 6 0 6.951110 0.599609 -0.599410

49 8 0 6.520013 -3.054248 -0.681497

50 8 0 8.358184 -1.087522 -1.530722

51 6 0 7.301894 -3.724914 0.318943

52 6 0 8.189755 -1.647720 -2.842110

53 7 0 2.097730 2.748292 -1.217470

54 6 0 1.631410 4.019088 -1.061390

55 8 0 1.968551 4.765172 -0.134460

56 6 0 0.646971 4.490581 -2.120379

57 1 0 1.665513 2.188665 -1.951895

58 8 0 4.375423 -2.323712 0.842971

59 6 0 3.413026 -2.909770 -0.043800

60 6 0 -0.436361 3.533745 2.488095

61 1 0 -3.651449 -1.466431 1.137439

62 1 0 -2.317587 0.909286 2.521669

63 1 0 -3.709218 -0.040620 3.044086

64 1 0 -3.545326 2.036235 0.810280

65 1 0 -4.619380 2.019172 2.202594

66 1 0 -4.084105 0.766171 -2.851140

67 1 0 -0.381799 -1.015624 0.289789

68 1 0 -2.163702 1.355881 -3.975518

69 1 0 -6.825006 1.224796 1.892233

70 1 0 -9.306577 -1.508187 -3.158722

71 1 0 -7.847710 -0.514472 -3.440182

72 1 0 -9.157765 0.091174 -2.376518

73 1 0 -10.363372 0.470773 1.735982

74 1 0 -8.822136 0.248489 2.609737

75 1 0 -9.090916 1.718858 1.624380

76 1 0 -0.010862 -4.191911 2.204940

77 1 0 -0.194865 -2.927402 3.450810

78 1 0 -1.261393 -4.357465 3.437939

79 1 0 -6.071722 -3.291531 -1.891639

80 1 0 -4.836097 -3.193626 -3.181016

81 1 0 -4.362936 -2.946200 -1.473917

82 1 0 1.149795 -1.905307 -3.009798

83 1 0 2.710570 -1.248852 -2.442786

84 1 0 1.668205 -0.262957 -3.512071

85 1 0 3.513201 1.292638 -1.137410

86 1 0 4.763519 3.270032 -1.424682

87 1 0 4.132290 4.018997 0.044984

88 1 0 5.355078 2.568841 1.489044

89 1 0 6.498642 3.031222 0.235417

90 1 0 4.250076 -0.939214 2.825773

91 1 0 1.369224 2.934536 1.081455

92 1 0 2.388432 -1.094196 4.164793

93 1 0 7.671763 1.336814 -0.941522

94 1 0 8.293100 -3.266521 0.408724

95 1 0 6.791665 -3.701596 1.288006

96 1 0 7.405711 -4.759109 -0.016128

97 1 0 9.196072 -1.851119 -3.213970

98 1 0 7.613114 -2.575813 -2.805099

99 1 0 7.694786 -0.928535 -3.506443

100 1 0 -0.206265 4.961479 -1.623179

101 1 0 0.299361 3.692523 -2.779253

102 1 0 1.137781 5.262643 -2.724286

103 1 0 3.892839 -3.266931 -0.960731

104 1 0 2.622462 -2.190342 -0.282059

105 1 0 2.981688 -3.756855 0.494153

106 1 0 -0.728696 3.490402 1.433226

107 1 0 -1.270828 3.902934 3.084934

108 1 0 0.425959 4.199115 2.600382

---------------------------------------------------------------------

Conformer 6:

Energy = -2718.878014 Hartree

Standard orientation:

---------------------------------------------------------------------

Center Atomic Atomic Coordinates (Angstroms)

Number Number Type X Y Z

---------------------------------------------------------------------

1 6 0 -2.508145 -0.392249 -0.008699

2 6 0 -3.062748 -0.880804 1.347147

3 6 0 -3.403464 0.300042 2.287239

4 6 0 -4.189962 1.430602 1.588520

5 6 0 -5.257472 0.895031 0.661743

6 6 0 -4.869456 0.233228 -0.525106

7 6 0 -3.425183 0.147257 -0.908557

8 6 0 -3.097097 0.678290 -2.193865

9 6 0 -1.124576 -0.592911 -0.242753

10 6 0 -0.264670 -0.218794 -1.251207

11 6 0 -0.534779 0.693714 -2.369351

12 6 0 -1.893299 0.913675 -2.806532

13 8 0 0.417477 1.252164 -2.967116

14 8 0 1.016332 -0.641807 -1.074318

15 6 0 -5.864610 -0.322511 -1.349631

16 6 0 -7.225649 -0.207659 -1.024704

17 6 0 -7.598110 0.467876 0.147838

18 6 0 -6.611481 1.001337 0.980474

19 8 0 -8.183121 -0.785987 -1.826860

20 8 0 -8.917243 0.665778 0.474316

21 6 0 -8.522102 -0.014040 -2.989425

22 6 0 -9.653435 -0.512853 0.836245

23 7 0 -2.208422 -1.816450 2.053272

24 6 0 -2.308878 -3.162758 1.863674

25 8 0 -3.105424 -3.666538 1.067784

26 6 0 -1.379438 -4.006824 2.719826

27 1 0 -1.505757 -1.453285 2.698673

28 8 0 -5.510354 -0.974592 -2.510344

29 6 0 -5.472140 -2.406840 -2.385860

30 6 0 1.718851 -1.206196 -2.200797

31 6 0 2.874416 1.224632 0.920332

32 6 0 3.262952 2.129793 -0.263406

33 6 0 4.385238 3.128270 0.105986

34 6 0 5.508981 2.439682 0.910153

35 6 0 5.897512 1.092077 0.343028

36 6 0 5.004427 -0.000223 0.446247

37 6 0 3.711566 0.144545 1.189944

38 6 0 3.454844 -0.839588 2.191766

39 6 0 1.720063 1.619135 1.651665

40 6 0 0.954116 0.979741 2.599303

41 6 0 1.084297 -0.410565 3.081843

42 6 0 2.352401 -1.088538 2.965975

43 8 0 0.125906 -0.955036 3.672580

44 8 0 -0.112590 1.577069 3.178264

45 6 0 5.359898 -1.229826 -0.153425

46 6 0 6.585267 -1.375275 -0.826024

47 6 0 7.485834 -0.298120 -0.860336

48 6 0 7.129513 0.923047 -0.290212

49 8 0 6.890262 -2.564501 -1.453316

50 8 0 8.682712 -0.390879 -1.529522

51 6 0 6.718757 -2.532399 -2.880206

52 6 0 9.640432 -1.299448 -0.962317

53 7 0 2.133818 2.753612 -0.961088

54 6 0 1.530609 3.939636 -0.673773

55 8 0 1.784477 4.622198 0.325751

56 6 0 0.497940 4.401962 -1.691095

57 1 0 1.723541 2.200621 -1.714142

58 8 0 4.423138 -2.234345 -0.160863

59 6 0 4.802693 -3.525681 0.343371

60 6 0 -0.375304 2.961485 2.936773

61 1 0 -3.985113 -1.424165 1.127728

62 1 0 -3.990736 -0.109348 3.117724

63 1 0 -2.479102 0.708637 2.712072

64 1 0 -3.490018 2.052872 1.017502

65 1 0 -4.639115 2.077602 2.348810

66 1 0 -3.959207 1.007993 -2.765621

67 1 0 -0.623171 -1.175582 0.524058

68 1 0 -1.934163 1.424458 -3.766348

69 1 0 -6.925989 1.513866 1.885045

70 1 0 -7.649838 0.114587 -3.638315

71 1 0 -8.919994 0.965899 -2.701138

72 1 0 -9.292454 -0.580382 -3.516942

73 1 0 -10.663322 -0.174212 1.076870

74 1 0 -9.208666 -0.987975 1.719437

75 1 0 -9.688197 -1.226738 0.009251

76 1 0 -1.979159 -4.582458 3.433646

77 1 0 -0.859717 -4.723931 2.077274

78 1 0 -0.647969 -3.407656 3.267834

79 1 0 -5.143830 -2.787562 -3.355045

80 1 0 -4.759428 -2.710410 -1.609528

81 1 0 -6.466364 -2.800245 -2.151040

82 1 0 1.940785 -0.445353 -2.949495

83 1 0 2.634081 -1.622834 -1.779372

84 1 0 1.120929 -2.007396 -2.650060

85 1 0 3.689641 1.456474 -1.011906

86 1 0 4.781673 3.541386 -0.829837

87 1 0 3.974945 3.956650 0.685362

88 1 0 5.172698 2.315789 1.946864

89 1 0 6.383159 3.098147 0.938678

90 1 0 4.267594 -1.544574 2.340281

91 1 0 1.374383 2.608614 1.381072

92 1 0 2.397821 -1.970114 3.601768

93 1 0 7.834409 1.746621 -0.355053

94 1 0 6.981438 -3.529259 -3.240015

95 1 0 7.380640 -1.788673 -3.334870

96 1 0 5.677080 -2.310813 -3.139730

97 1 0 10.524438 -1.240929 -1.600371

98 1 0 9.257494 -2.323385 -0.950826

99 1 0 9.904640 -0.990175 0.056262

100 1 0 0.876037 5.307915 -2.178032

101 1 0 0.274492 3.653616 -2.454245

102 1 0 -0.420477 4.676210 -1.163209

103 1 0 5.463969 -4.046085 -0.350745

104 1 0 5.298709 -3.429921 1.316303

105 1 0 3.869235 -4.078471 0.465940

106 1 0 -1.202735 3.217518 3.599007

107 1 0 0.498175 3.577327 3.175443

108 1 0 -0.669485 3.135058 1.895979

---------------------------------------------------------------------

Conformer 7:

Energy = -2718.877963 Hartree

Standard orientation:

---------------------------------------------------------------------

Center Atomic Atomic Coordinates (Angstroms)

Number Number Type X Y Z

---------------------------------------------------------------------

1 6 0 -2.638461 -0.764645 -0.185300

2 6 0 -2.954182 -0.745044 1.326669

3 6 0 -2.258006 0.429010 2.061785

4 6 0 -2.376645 1.788424 1.342232

5 6 0 -3.755054 2.009036 0.766242

6 6 0 -4.162636 1.216395 -0.327638

7 6 0 -3.214454 0.242157 -0.958359

8 6 0 -2.947429 0.466040 -2.342559

9 6 0 -1.850794 -1.844764 -0.666682

10 6 0 -1.240542 -2.079559 -1.880285

11 6 0 -1.035779 -1.115427 -2.979402

12 6 0 -2.011243 -0.074146 -3.186096

13 8 0 -0.079342 -1.267577 -3.769527

14 8 0 -0.625230 -3.247915 -2.165204

15 6 0 -5.464185 1.376732 -0.835398

16 6 0 -6.348150 2.315714 -0.280255

17 6 0 -5.925330 3.105964 0.799482

18 6 0 -4.638940 2.937893 1.316736

19 8 0 -7.629609 2.433531 -0.769773

20 8 0 -6.732895 4.084774 1.327147

21 6 0 -7.740387 3.285987 -1.919519

22 6 0 -7.876303 3.615317 2.057476

23 7 0 -2.648285 -1.974243 2.040262

24 6 0 -3.582011 -2.955156 2.200065

25 8 0 -4.694844 -2.904864 1.672183

26 6 0 -3.157616 -4.130135 3.064300

27 1 0 -1.749357 -2.051263 2.516304

28 8 0 -5.878769 0.626836 -1.914203

29 6 0 -6.680332 -0.512838 -1.561627

30 6 0 -0.961640 -4.428239 -1.421277

31 6 0 2.613110 -0.736186 -0.020079

32 6 0 2.927050 -0.500908 -1.515132

33 6 0 2.269395 0.793826 -2.055441

34 6 0 2.431283 2.014839 -1.126144

35 6 0 3.816479 2.100158 -0.529937

36 6 0 4.207637 1.136083 0.423980

37 6 0 3.238433 0.099781 0.902138

38 6 0 3.009251 0.088587 2.314939

39 6 0 1.787069 -1.847251 0.297166

40 6 0 1.211050 -2.283472 1.465335

41 6 0 1.084507 -1.549215 2.730534

42 6 0 2.094292 -0.578603 3.086202

43 8 0 0.139416 -1.809356 3.510678

44 8 0 0.514145 -3.455569 1.363344

45 6 0 5.516240 1.179640 0.938517

46 6 0 6.423108 2.170200 0.529842

47 6 0 6.016464 3.132238 -0.407683

48 6 0 4.723213 3.081037 -0.933001

49 8 0 7.709057 2.174862 1.021004

50 8 0 6.843634 4.162464 -0.782123

51 6 0 7.846779 2.824619 2.294293

52 6 0 7.990761 3.789568 -1.561728

53 7 0 2.571847 -1.601954 -2.392690

54 6 0 3.432706 -2.629309 -2.637769

55 8 0 4.534807 -2.719784 -2.091592

56 6 0 2.942261 -3.652718 -3.648593

57 1 0 1.672395 -1.577944 -2.877909

58 8 0 5.915764 0.256048 1.878895

59 6 0 6.681920 -0.834638 1.339644

60 6 0 0.724153 -4.442780 2.392567

61 1 0 -4.035612 -0.609001 1.413887

62 1 0 -2.714911 0.491960 3.056470

63 1 0 -1.198338 0.196080 2.213459

64 1 0 -1.634327 1.833039 0.535758

65 1 0 -2.125121 2.587015 2.047540

66 1 0 -3.540093 1.262378 -2.783057

67 1 0 -1.689539 -2.617844 0.077056

68 1 0 -1.941515 0.362014 -4.180374

69 1 0 -4.336293 3.558606 2.155263

70 1 0 -7.413580 4.304337 -1.679426

71 1 0 -7.149406 2.892071 -2.753134

72 1 0 -8.797909 3.295971 -2.191756

73 1 0 -8.544552 3.034376 1.415935

74 1 0 -8.391997 4.508402 2.416695

75 1 0 -7.562772 3.006558 2.914699

76 1 0 -3.949624 -4.336687 3.789882

77 1 0 -3.050582 -5.019614 2.433756

78 1 0 -2.218001 -3.957639 3.595222

79 1 0 -7.610839 -0.197196 -1.078945

80 1 0 -6.906510 -1.027406 -2.497742

81 1 0 -6.126817 -1.186199 -0.895927

82 1 0 -0.494806 -4.412332 -0.434688

83 1 0 -0.565536 -5.258462 -2.007699

84 1 0 -2.048796 -4.523159 -1.325900

85 1 0 4.012645 -0.388918 -1.587439

86 1 0 2.727976 0.999536 -3.029730

87 1 0 1.202555 0.621953 -2.235292

88 1 0 1.690184 1.951262 -0.319916

89 1 0 2.204364 2.924514 -1.691232

90 1 0 3.626315 0.793575 2.863677

91 1 0 1.572057 -2.496449 -0.546749

92 1 0 2.056130 -0.307035 4.139095

93 1 0 4.433569 3.833875 -1.660372

94 1 0 7.549122 3.877342 2.226369

95 1 0 8.904631 2.759704 2.557263

96 1 0 7.246099 2.316891 3.056237

97 1 0 8.644719 3.113577 -1.004747

98 1 0 7.679949 3.316066 -2.501242

99 1 0 8.519224 4.719371 -1.782068

100 1 0 1.878335 -3.546822 -3.875930

101 1 0 3.136255 -4.656680 -3.260575

102 1 0 3.515701 -3.542043 -4.575882

103 1 0 7.616988 -0.470565 0.902362

104 1 0 6.900583 -1.497190 2.179473

105 1 0 6.103667 -1.377989 0.582490

106 1 0 0.128783 -5.305987 2.092692

107 1 0 0.395626 -4.073576 3.364665

108 1 0 1.782697 -4.721967 2.430417

---------------------------------------------------------------------

CompareVOA Similarity Results:

1. Bejcecine A

NS (IR) 91.8

NS (VCD aR) 80.7740

NS (VCD aS) 9.3552

ESI 71.419

Confidence Level 99%

2. Colchicine

NS (IR) 93.9

NS (VCD aR,7S) 80.8464

NS (VCD (aS,7R) 7.1687

ESI 73.678

Confidence Level 99%

3. Isobejcecine

NS (IR) 92.1

NS (VCD aR) 80.2978

NS (VCD aS) 13.8579

ESI 66.440

Confidence Level 99%

4. Isocolchicine

NS (IR) 93.5

NS (VCD aR,7S) 69.5903

NS (VCD aS,7R) 9.4126

ESI 60.178

Confidence Level 98%
