# Supplementary material for: Deconstruction of Desacetamidocolchicine’s B Ring Reveals a Class 3 Atropisomeric AC Ring with Tubulin Binding Properties
Source: J Org Chem. 2025 May 27;90(22):7246–58. doi: 10.1021/acs.joc.5c00284 (PMC12150326; doi:10.1021/acs.joc.5c00284)
Supplement: Supplementary file 3 [file jo5c00284_si_003.zip › VCD Reports/(+) and (-) DM-MTC VCD Report.pdf]

Title:

Absolute Configuration Determination Report

GENERAL INFORMATION	
**Customer**	CUNY Brooklyn
**Sales Order Number**	2021-41 LSNC
**Sample code (Our ref.)**	**Bejcecine A / Bejcecine B**
**Sample description (Your ref.)**	**Bejcecine A / Bejcecine B**
**VCD-spectrometer**	ChiralIR w/ Dual*PEM*
**Report prepared by**	Jordan Nafie
**Report validated and signed by**	Rina K Dukor
**Date**	May 19, 2021
**RESULTS**	
Absolute Configuration of Bejcecine A is (aR)<br>Absolute Configuration of Bejcecine B is (aS)	Confidence Level: **99%**
**MEASUREMENT PARAMETERS**	
**Concentration**	7mg / 150uL
**Solvent**	$CDCl_3$
**Resolution**	4 cm^{-1}
**PEM setting**	1400 cm^{-1}
**Number of scans/Measurement time**	12 hours per enantiomer
**Sample cell**	BaF_2
**Path length**	100 µm
**CALCULATION DETAILS**	
**Molecular Mechanics Force Field**	**MMFF94 (Compute VOA)**
**DFT Software version**	**Gaussian '09**
**Number of conformers used for Boltzmann sum**	**15 (cc-pVTZ / B3LYP)**
**Methodology and basis sets for DFT calculations**	**6-31Gd, cc-pVTZ / B3LYP, B3PW91 / CPCM (Chloroform)**
**Enantiomer used for calculation**	**aR**
**Total calculated conformers**	**82 (6-31Gd), 34 (cc-pVTZ)**
**Number of low-energy conformations shown in report**	**4**

COMMENTS

The confidence level is a measure of the degree of congruence between a calculated and measured spectrum. If identical spectra are being compared the confidence level is 100%. The confidence level (CL) is not the likelihood that the assignment is correct. Rather it's a measure of quality or degree of agreement between calculated and measured spectra. With a CL of 99% for this molecule, the visual agreement between measured and calculated spectra is excellent – this is a very high confidence assignment. Four different calculations were performed, two functionals (B3LYP and B3PW91) each with two basis sets (6-31G(d) and cc-pVTZ)– the CPCM solvent shell method was employed in every case. While all four indicated the same result for stereochemistry – the better overall match was cc-pVTZ / B3LYP. The nomenclature for atropisomers was used in this case (aR) indicating clockwise rotation from B to C substituents when viewed down the chiral axis.

Title:

Absolute Configuration Determination Report

Structure of Bejcecine A:

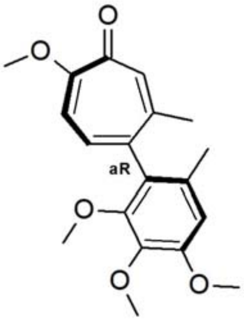

Bejcecine A

Structure of Bejcecine B:

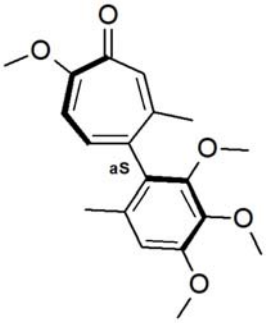

Bejcecine B

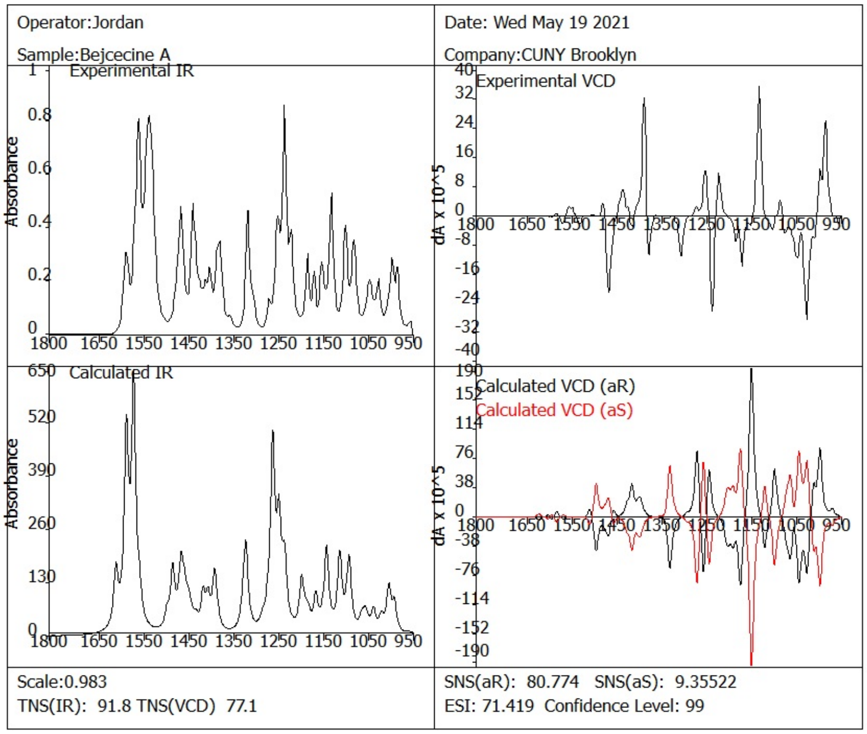

CompareVOA Results.
Please note: In this plot the frequency scaling factor is not applied.

17546 Bee Line Hwy | Jupiter, Florida 33458, USA
ph. 561 625.0133 | fax. 561 625.0717 | www.btools.com

Table 1. Numerical comparison describing the similarity in the range of 950- 1800 cm^{-1} between the calculated IR and VCD spectra for the **(aR)** enantiomer at the cc-pVTZ / B3LYP w/ CPCM (Chloroform) level and the observed IR and VCD spectra for **Bejcecine A**.

Cal. (950-1800cm^{-1})	Numerical comparison	Observed **Bejcecine A**
**(aR)**	scaling factor	0.983
	IR similarity (%)	91.8
	$^{a}\sum$ (%)	80.774
	$^{b}\Delta$ (%)	71.419
	Confidence Level (%)	99

$^{a}\sum$: single VCD similarity, gives the similarity between the calculated and observed VCD spectra.
$^{b}\Delta$: enatiomeric similarity index, gives the difference between the values of ∑ for both enantiomers of a given diastereoisomer.

17546 Bee Line Hwy │ Jupiter, Florida 33458, USA
ph. 561 625.0133 │ fax. 561 625.0717 │ www.btools.com

Title:

Absolute Configuration Determination Report

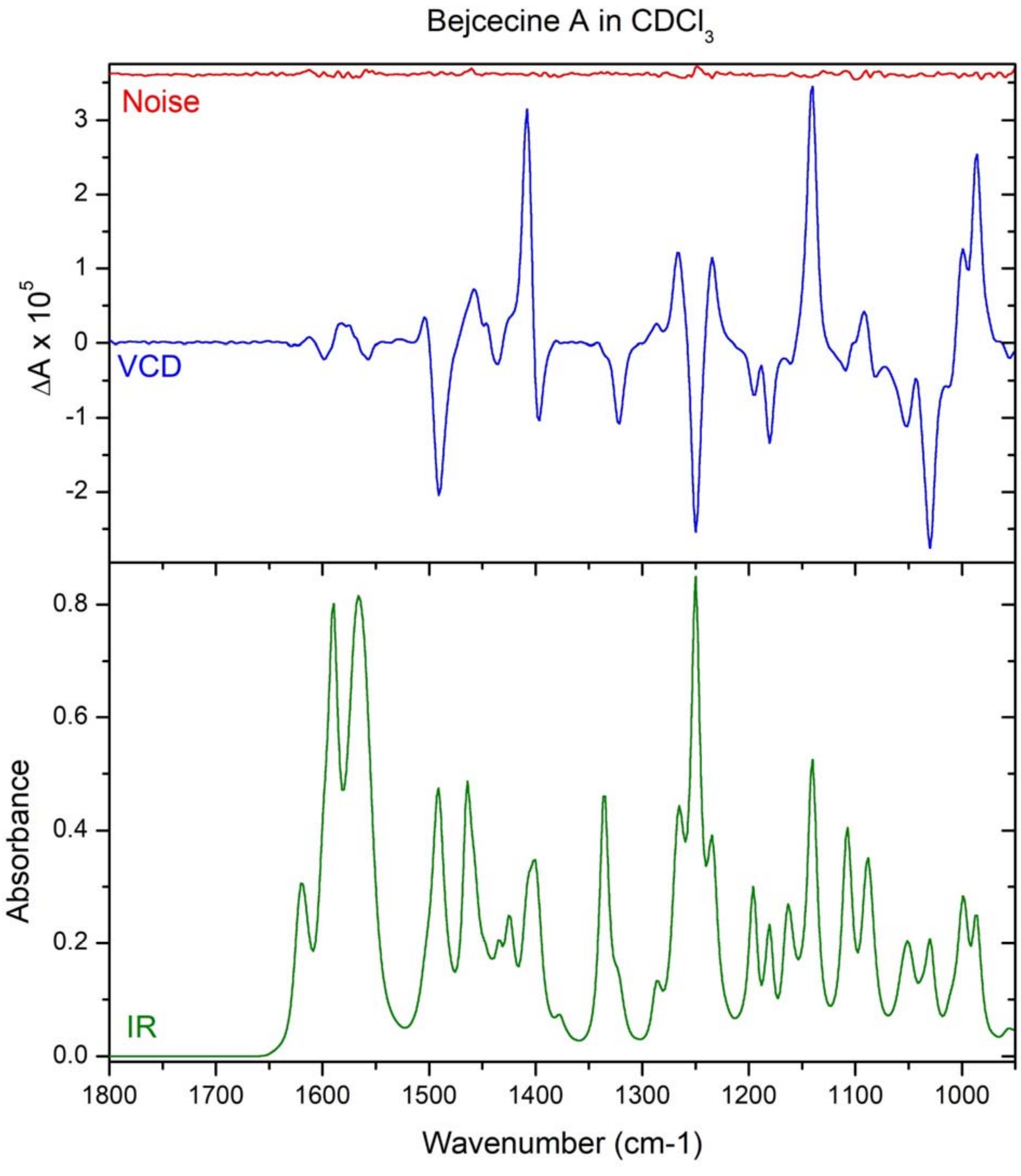

IR (lower frame) and VCD (upper frame) spectra of **Bejcecine A** in $CDCl_3$; 100um path-length cell with BaF_2 windows; 12 h collection for each enantiomer; instrument optimized at 1400 cm^{-1}. Solvent subtracted IR and enantiomer subtracted VCD spectra are shown. Uppermost trace is the VCD noise spectrum.

Title:

Absolute Configuration Determination Report

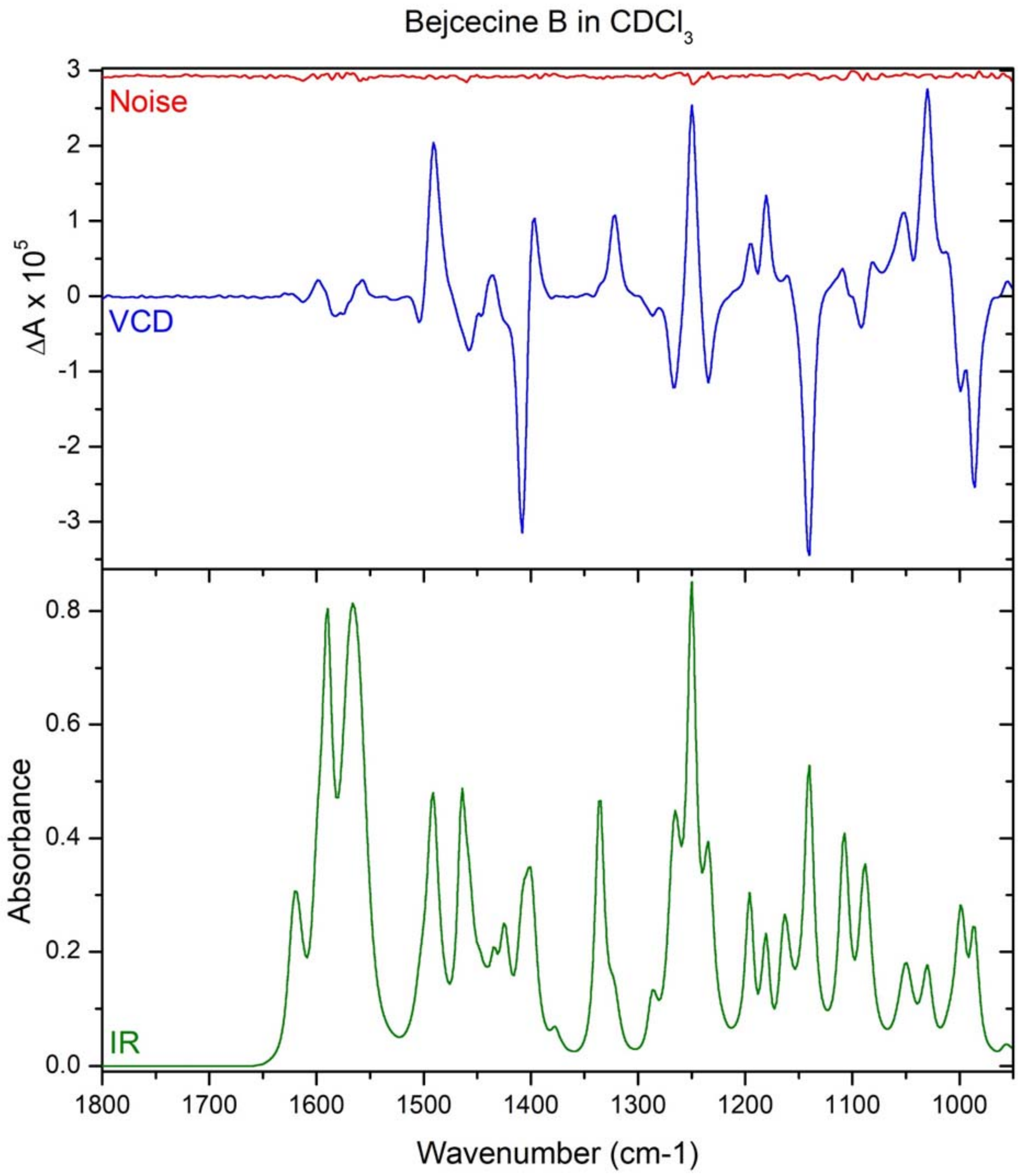

IR (lower frame) and VCD (upper frame) spectra of **Bejcecine B** in $CDCl_3$; 100um path-length cell with BaF_2 windows; 12 h collection for each enantiomer; instrument optimized at 1400 cm^{-1}. Solvent subtracted IR and enantiomer subtracted VCD spectra are shown. Uppermost trace is the VCD noise spectrum.

Title:

Absolute Configuration Determination Report

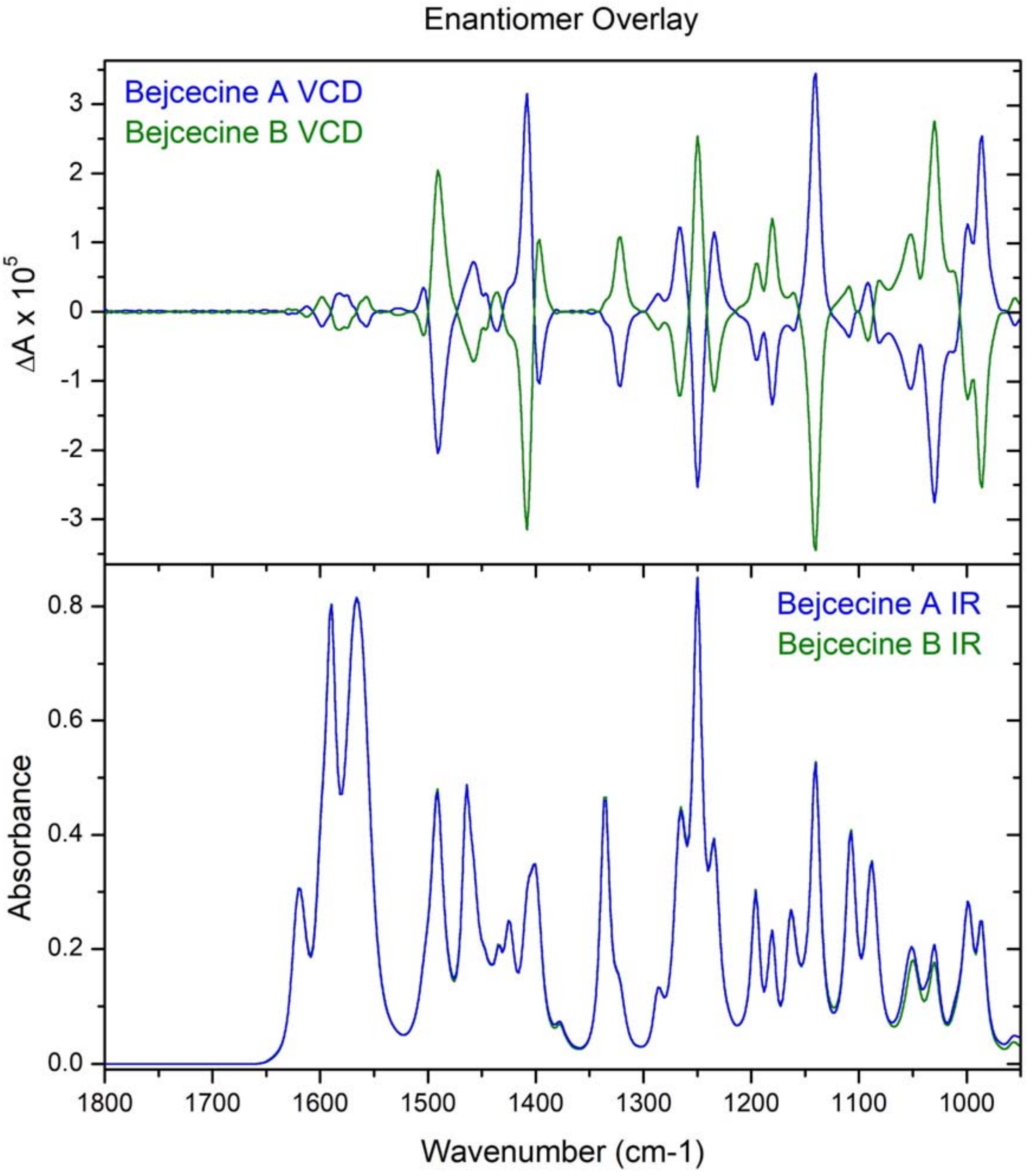

Overlay of both enantiomers, **Bejcecine A** and **Bejcecine B**. The IR are nearly identical as expected. The VCD are mirror images due to the half difference processing (E1 – E2) / 2.

Absolute Configuration Determination Report

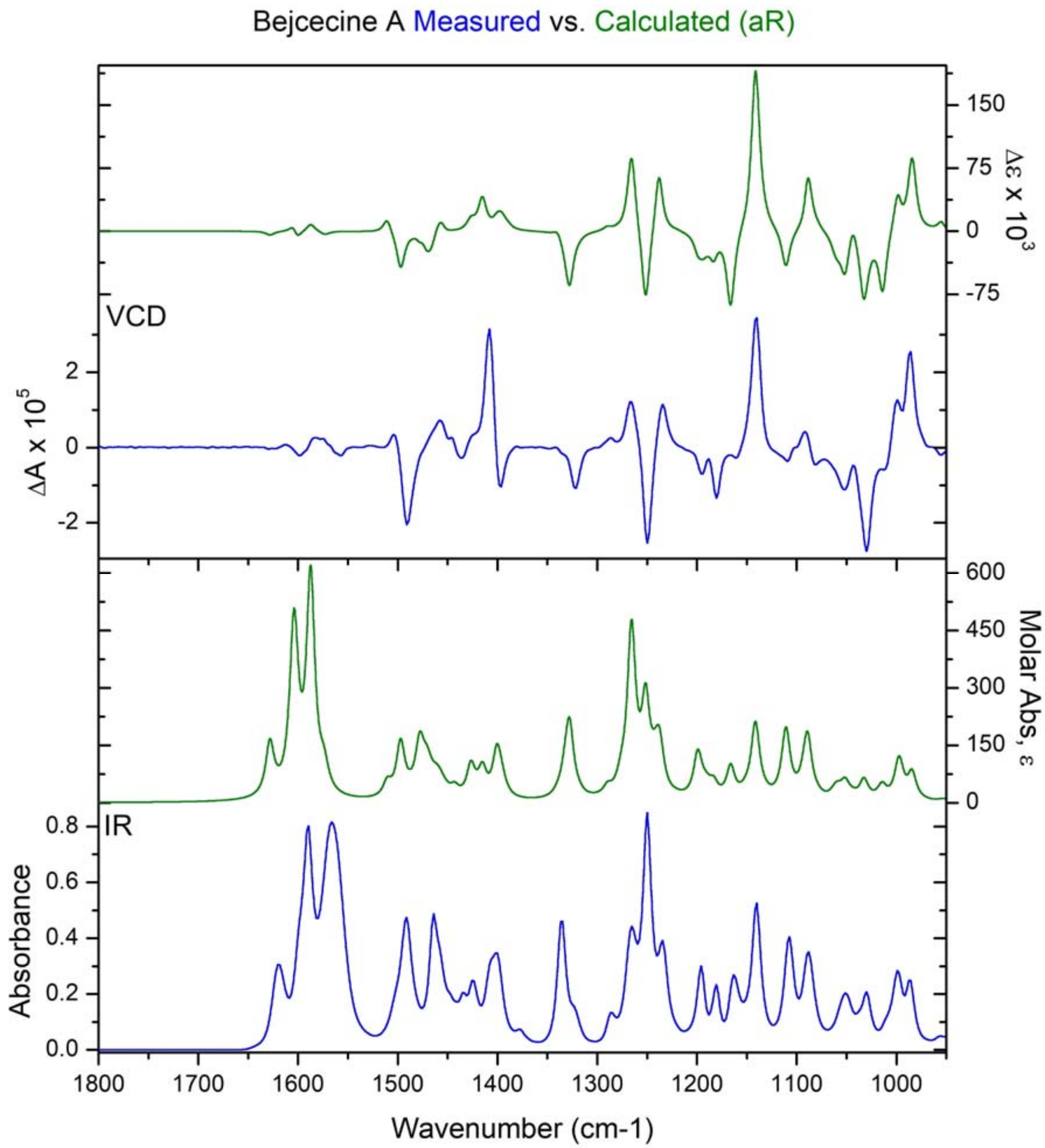

IR (lower frame) and VCD (upper frame) spectra observed for **Bejcecine A** (left axes) compared with Boltzmann-averaged spectra of the calculated conformations for the **(aR)** configuration, (right axes).

17546 Bee Line Hwy │ Jupiter, Florida 33458, USA
ph. 561 625.0133 │ fax. 561 625.0717 │ www.btools.com

Four lowest energy conformers (of 15 from Boltzmann average) - (aR) Configuration:

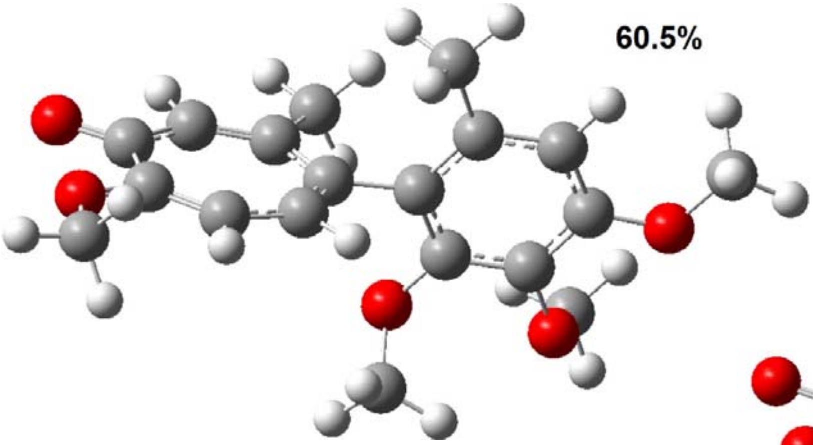

60.5%

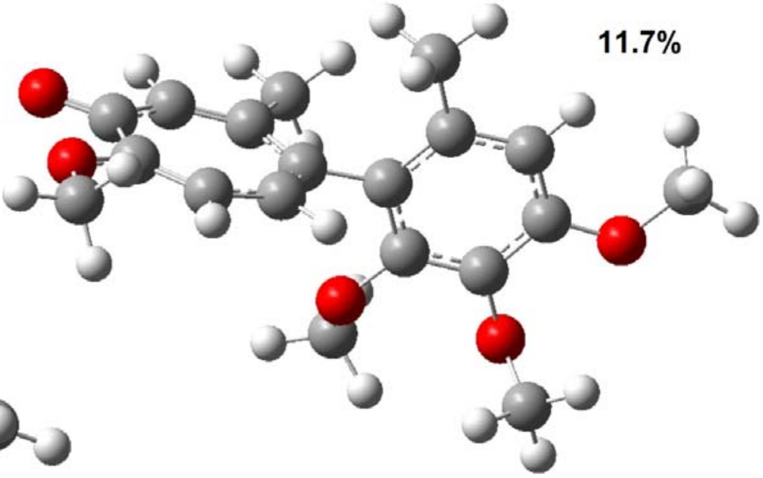

11.7%

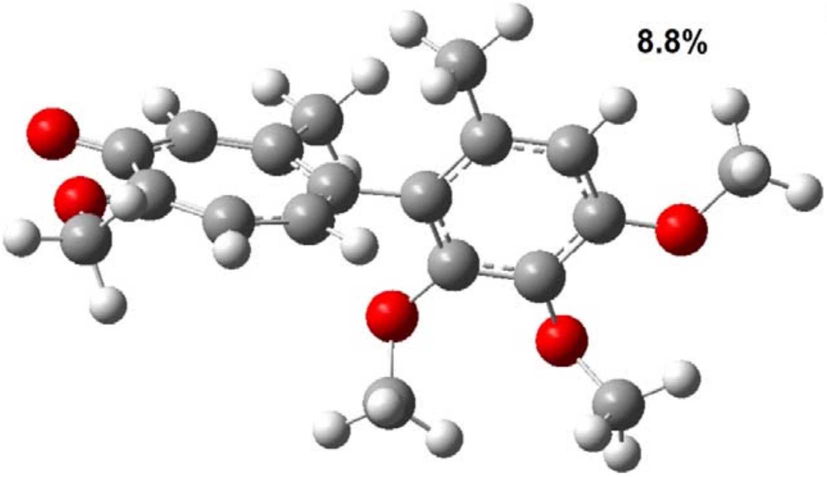

8.8%

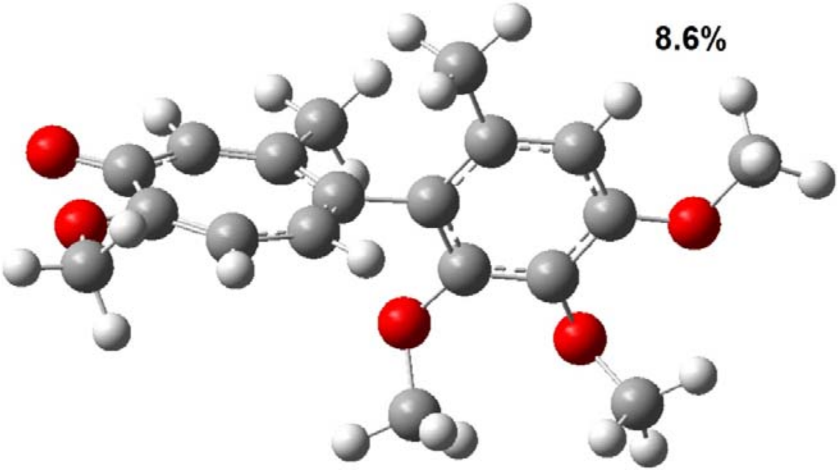

8.6%

Absolute Configuration Determination Report

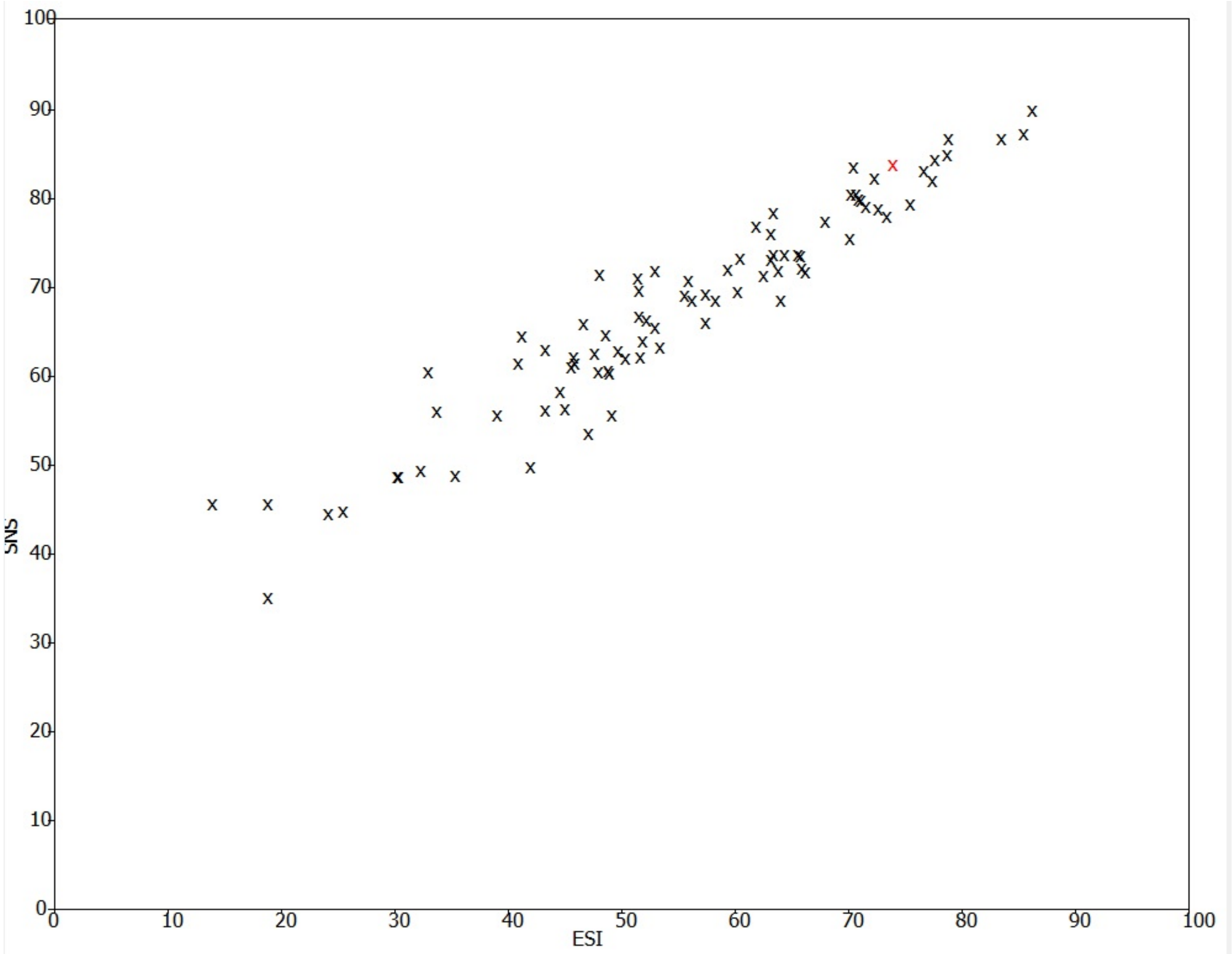

Plot of ESI (similarity of correct enantiomer minus incorrect enantiomer to calculated) vs SNS (overall similarity of correct enantiomer to calculated) for a library of correct assignments verified independently by X-Ray other method (Black X marks). Red X is **Bejcecine A**.

17546 Bee Line Hwy | Jupiter, Florida 33458, USA
ph. 561 625.0133 | fax. 561 625.0717 | www.btools.com
