# Supplementary material for: Deconstruction of Desacetamidocolchicine’s B Ring Reveals a Class 3 Atropisomeric AC Ring with Tubulin Binding Properties
Source: J Org Chem. 2025 May 27;90(22):7246–58. doi: 10.1021/acs.joc.5c00284 (PMC12150326; doi:10.1021/acs.joc.5c00284)
Supplement: Supplementary file 3 [file jo5c00284_si_003.zip › VCD Reports/(+) and (-) IsoDM-MTC VCD Report.pdf]

Title:

VCD Absolute Configuration Determination Report

**GENERAL INFORMATION**	
**Customer**	CUNY Brooklyn
**Sales Order Number**	2021-41 LSNC
**Sample code (BT ref.)**	**Isobejcecine C / Isobejcecine D**
**Sample description (Customer ref.)**	**Isobejcecine C / Isobejcecine D**
**VCD-spectrometer**	ChiralIR w/ Dual*PEM*
**Report prepared by: (name / signature as needed)**	Jordan Nafie
**Report validated and signed by**	Rina K Dukor
**Date**	August 26, 2022
**RESULTS**	
Absolute Configuration of Isobejcecine D is (aR)<br>Absolute Configuration of Isobejcecine C is (aS)	Confidence Level: **99%**
**MEASUREMENT PARAMETERS**	
**Concentration**	7.7mg / 125uL
**Solvent**	$CDCl_3$
**Instrument Resolution**	4 cm^{-1}
**PEM setting**	1400 cm^{-1}
**Number of scans/Measurement time**	12 hours per enantiomer
**Sample cell**	BaF_2
**Path length**	100 µm
**CALCULATION DETAILS**	
**Molecular Mechanics Force Field**	**MMFF94 (Compute VOA)**
**DFT Software version**	**Gaussian '09**
**Number of conformers used for Boltzmann sum**	**16 (cc-pVTZ / B3PW91)**
**Methodology and basis sets for DFT calculations**	**6-31G(d), cc-pVTZ / B3LYP, B3PW91 / CPCM (Chloroform)**
**Enantiomer used for calculation**	**aR**
**Total calculated conformers**	**151**
**Number of low-energy conformations shown in report**	**4**

COMMENTS

The confidence level is a measure of the degree of congruence between a calculated and measured spectrum. If identical spectra are being compared the confidence level is 100%. The confidence level (CL) is not the likelihood that the assignment is correct. Rather it's a measure of quality or degree of agreement between calculated and measured spectra. With a CL of 99% for this molecule, the visual agreement between measured and calculated spectra is excellent – this is a very high confidence assignment. Four different calculations were performed, two functionals (B3LYP and B3PW91) each with two basis sets (6-31G(d) and cc-pVTZ). All four gave the same answer for the stereochemistry, with the combination of cc-pVTZ with B3PW91 giving the best overall agreement with the experiment.

Title:

VCD Absolute Configuration Determination Report

Structure of Isobejcecine D:

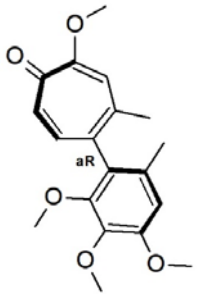

Isobejcecine D

Structure of Isobejcecine C:

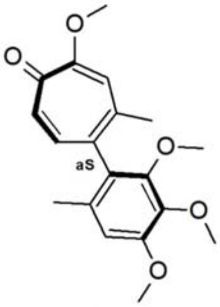

Isobejcecine C

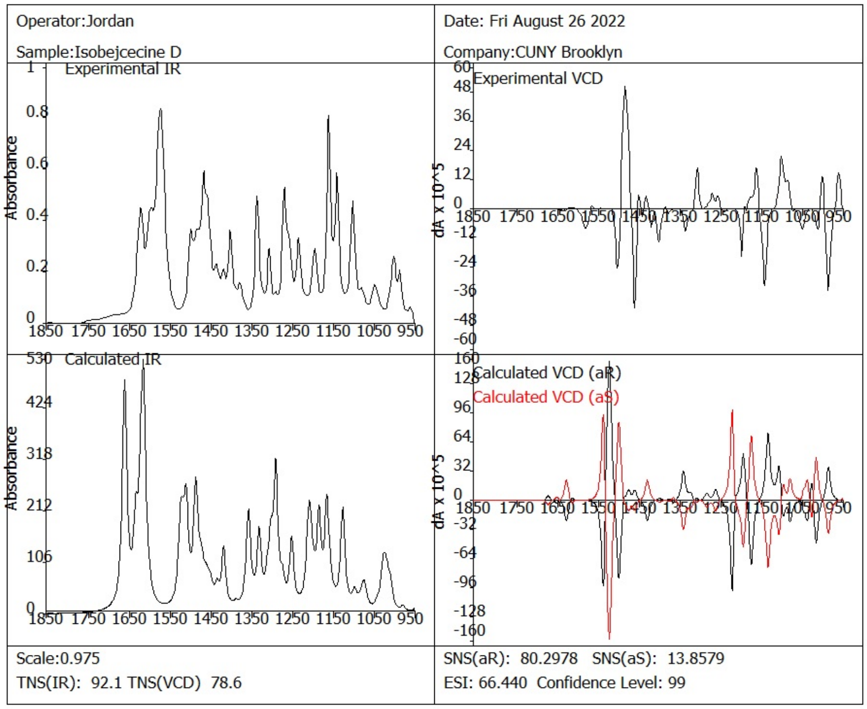

CompareVOA Results.
Please note: In this plot the frequency scaling factor is not applied.

Table 1. Numerical comparison describing the similarity in the range of 950- 1850 cm^{-1} between the calculated IR and VCD spectra for the **(aR)** enantiomer at the cc-pVTZ / B3PW91 w/ CPCM (Chloroform) level and the observed IR and VCD spectra for **Isobejcecine D**.

Cal. (950-1850cm^{-1})	Numerical comparison	Observed **Isobejcecine D**
**(aR)**	scaling factor	0.975
	IR similarity (%)	92.1
	$^{a}\sum$ (%)	80.2978
	$^{b}\Delta$ (%)	66.440
	Confidence Level (%)	99

$^{a}\sum$: single VCD similarity, gives the similarity between the calculated and observed VCD spectra.
$^{b}\Delta$: enatiomeric similarity index, gives the difference between the values of $\sum$ for both enantiomers of a given diastereoisomer.

Title:

VCD Absolute Configuration Determination Report

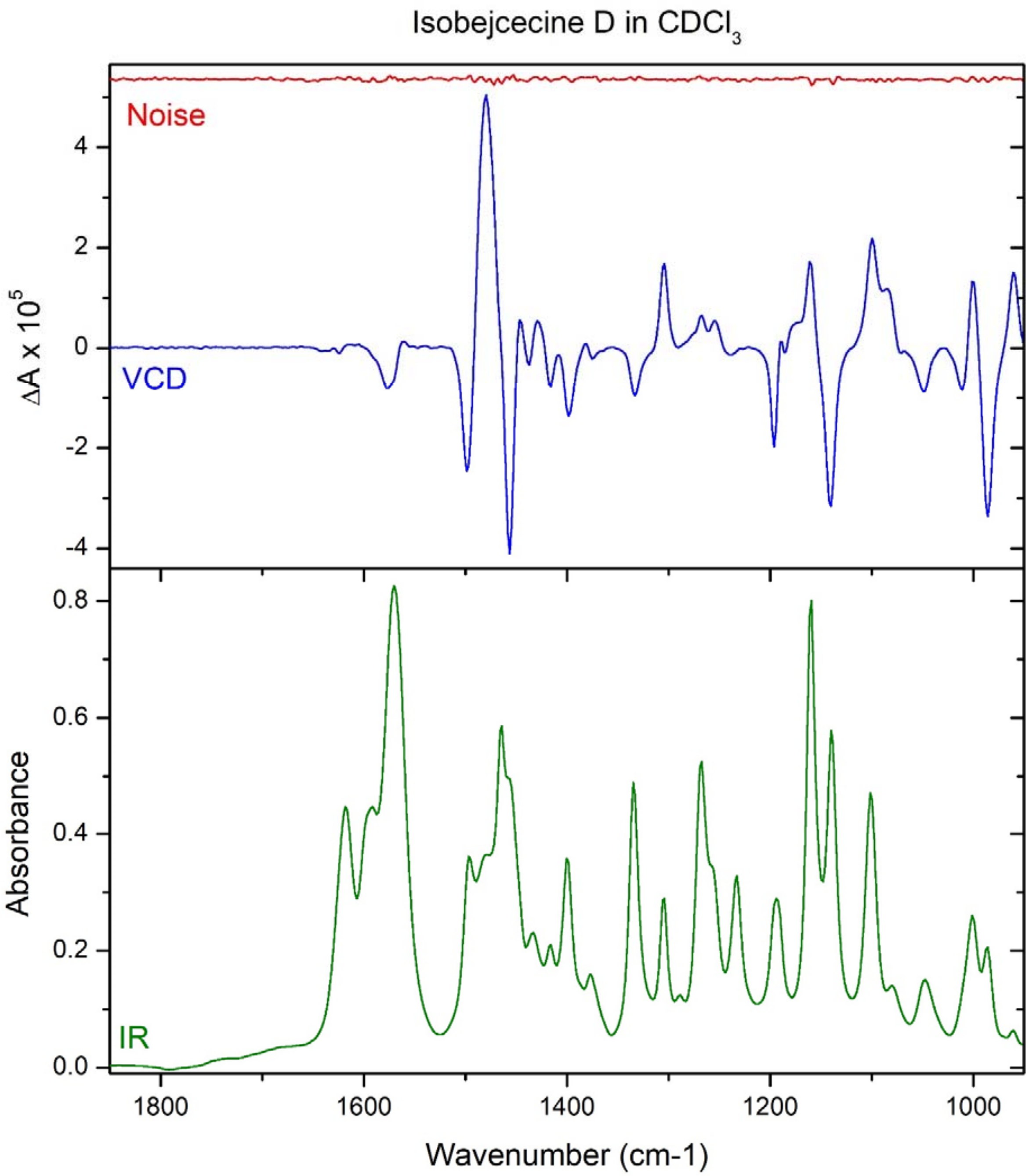

IR (lower frame) and VCD (upper frame) spectra of **Isobejcecine D** in $CDCl_3$; 100um path-length cell with BaF_2 windows; 12 h collection for each enantiomer; instrument optimized at 1400 cm^{-1}. Solvent subtracted IR and enantiomer subtracted VCD spectra are shown. Uppermost trace is the VCD noise spectrum.

Title:

VCD Absolute Configuration Determination Report

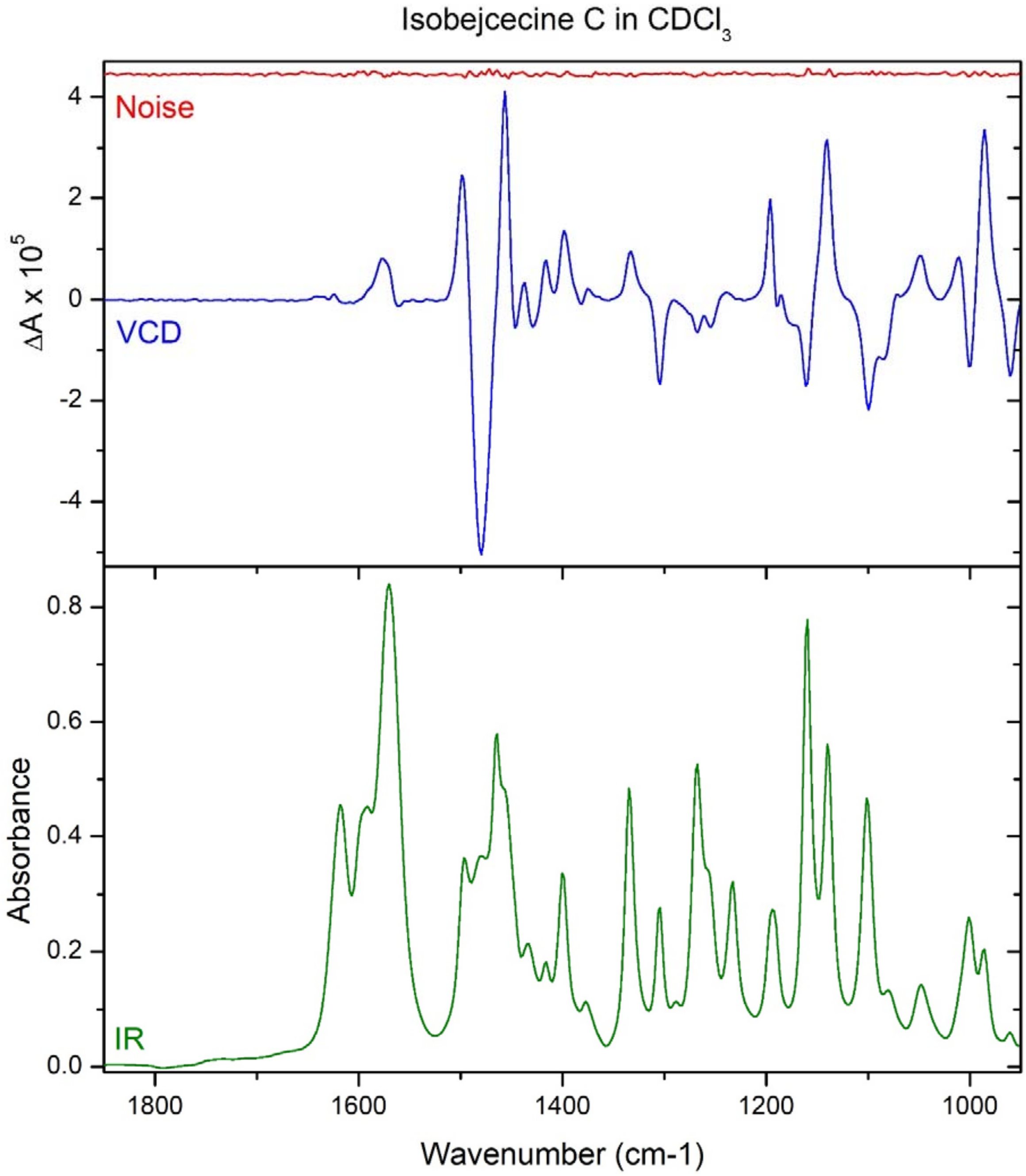

IR (lower frame) and VCD (upper frame) spectra of **Isobejcecine C** in $CDCl_3$; 100um path-length cell with BaF_2 windows; 12 h collection for each enantiomer; instrument optimized at 1400 cm^{-1}. Solvent subtracted IR and enantiomer subtracted VCD spectra are shown. Uppermost trace is the VCD noise spectrum.

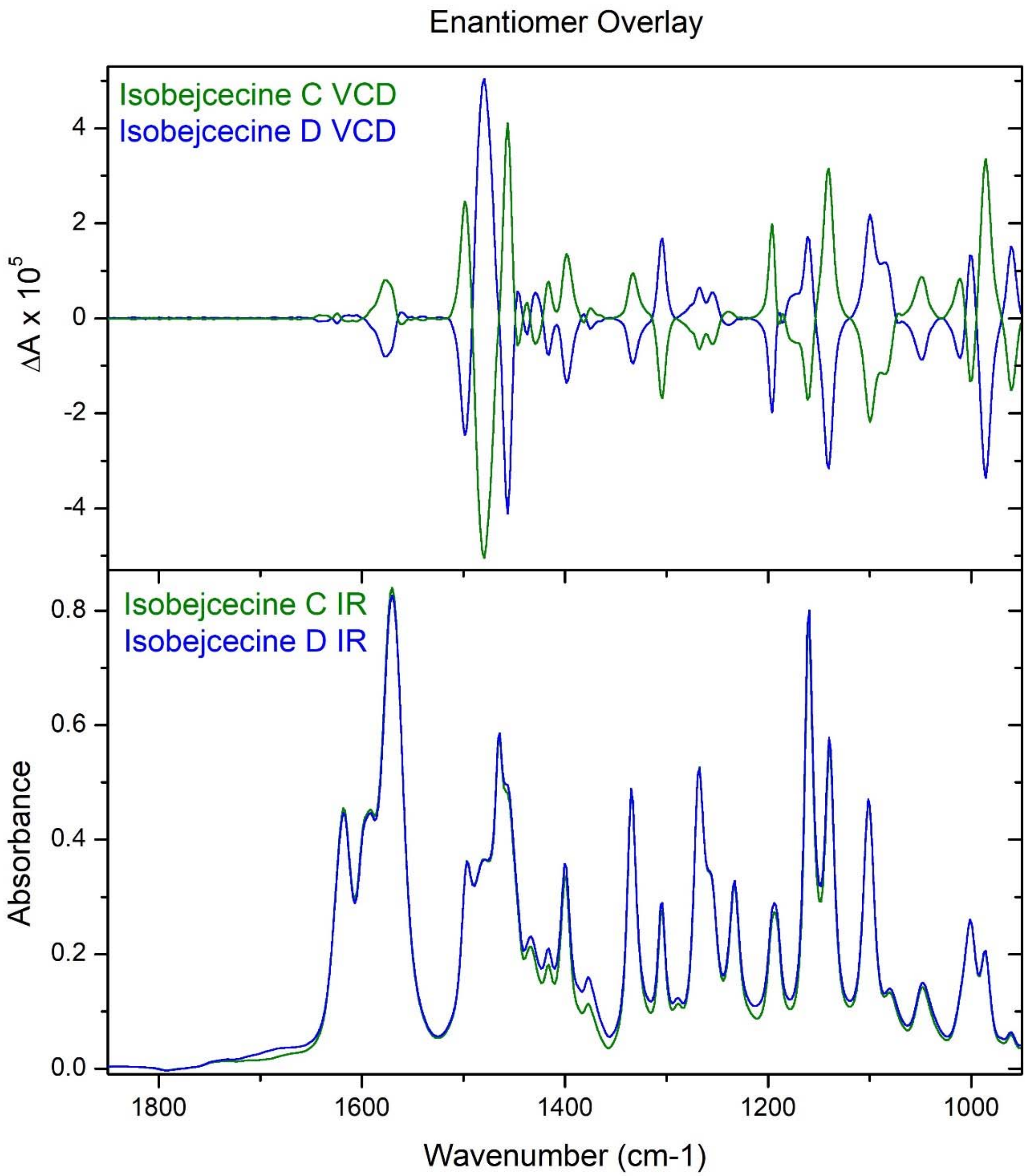

Overlay of both enantiomers, **Isobejcecine D** and **Isobejcecine C**. The IR are nearly identical as expected. The VCD are mirror images due to the half difference processing (E1 – E2) / 2.

VCD Absolute Configuration Determination Report

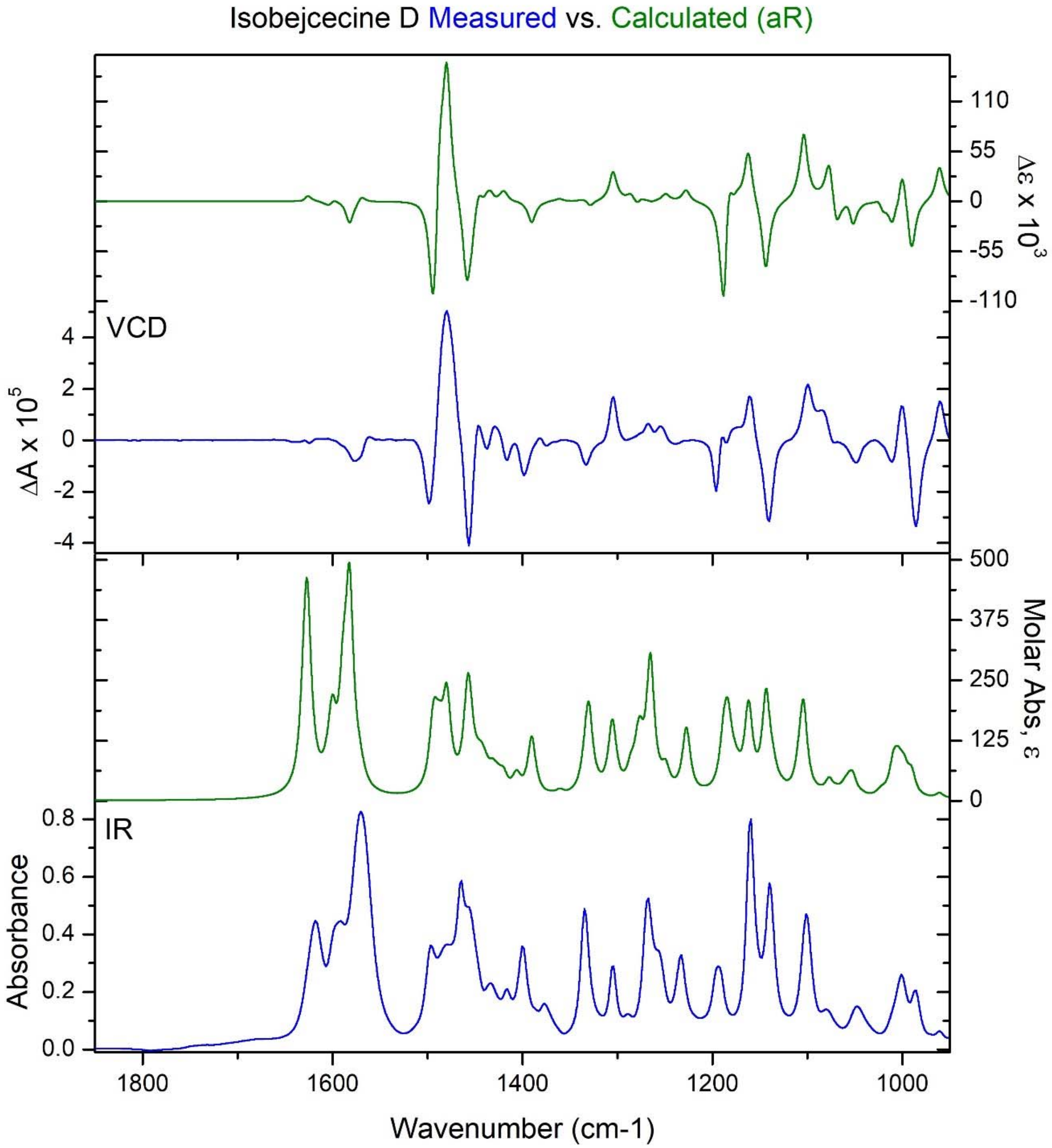

IR (lower frame) and VCD (upper frame) spectra observed for **Isobejcecine D** (left axes) compared with Boltzmann-averaged spectra of the calculated conformations for the **(aR)** configuration, (right axes).

Four lowest energy conformers (of 16 from Boltzmann average) - (aR) Configuration:

55.5%

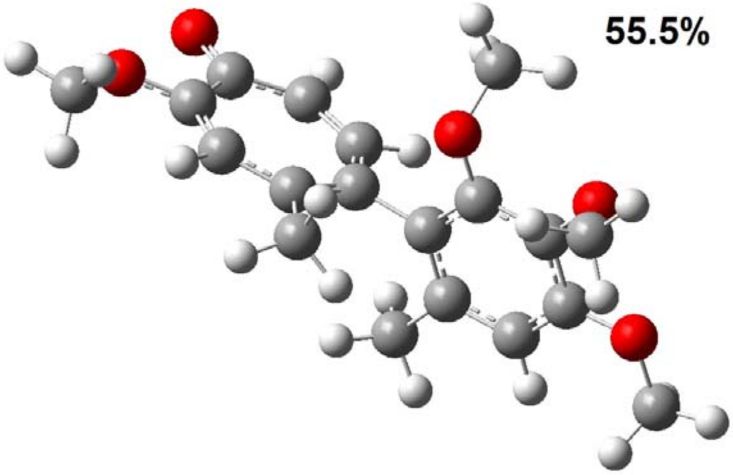

27.5%

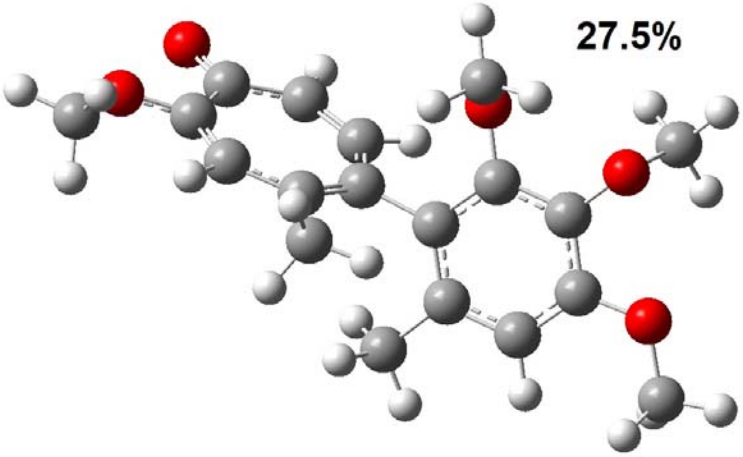

8.7%

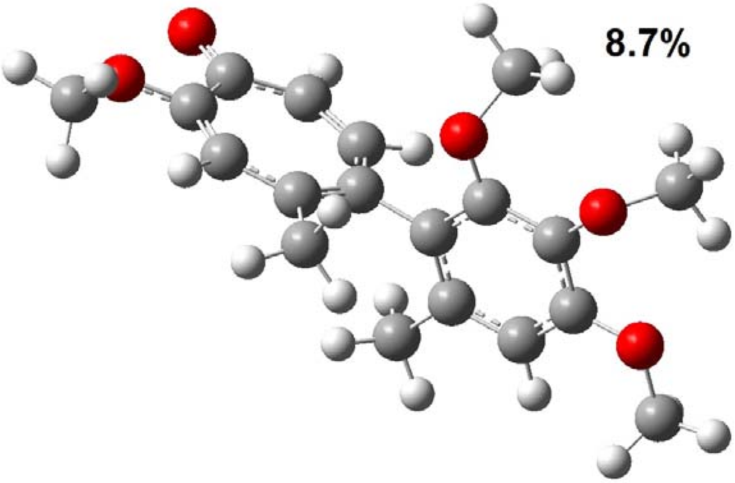

4.8%

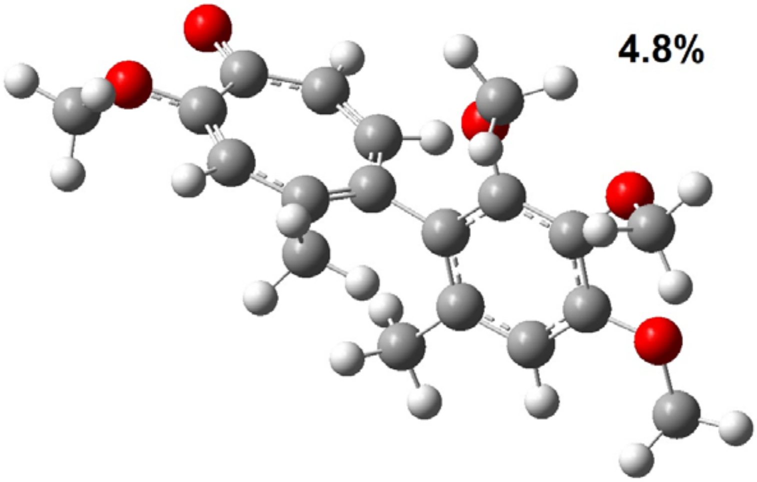

VCD Absolute Configuration Determination Report

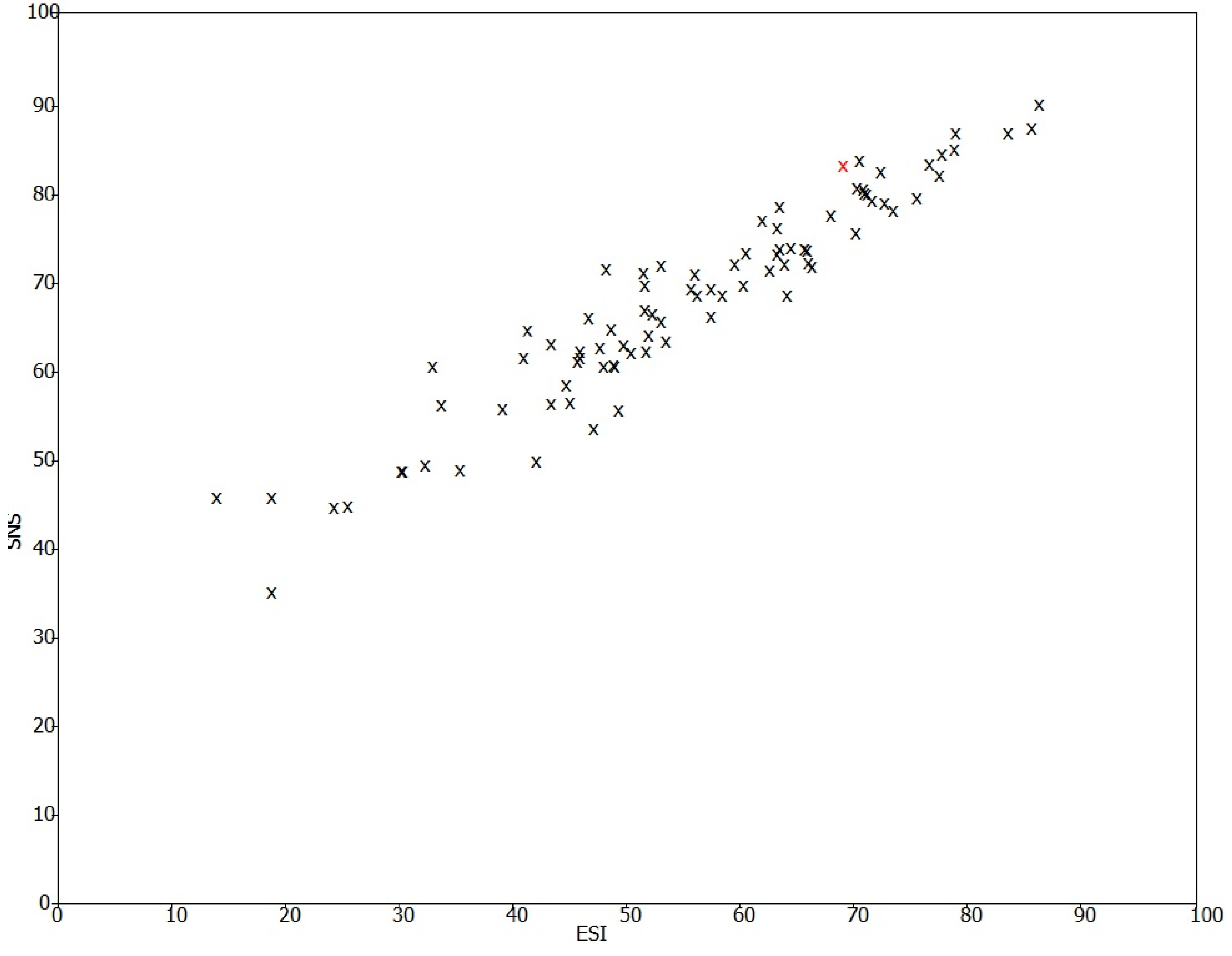

Plot of ESI (similarity of correct enantiomer minus incorrect enantiomer to calculated) vs SNS (overall similarity of correct enantiomer to calculated) for a library of correct assignments verified independently by X-Ray other method (Black X marks). Red X is **Isobejcecine D**. Upper right corner is the strongest results, lower left is the weakest.
