# Supplementary material for: Deconstruction of Desacetamidocolchicine’s B Ring Reveals a Class 3 Atropisomeric AC Ring with Tubulin Binding Properties
Source: J Org Chem. 2025 May 27;90(22):7246–58. doi: 10.1021/acs.joc.5c00284 (PMC12150326; doi:10.1021/acs.joc.5c00284)
Supplement: Supplementary file 3 [file jo5c00284_si_003.zip › VCD Reports/(+) and (-) Isocolchicine VCD Report.pdf]

Title:

VCD Absolute Configuration Determination Report

**GENERAL INFORMATION**	
**Customer**	CUNY Brooklyn
**Sales Order Number**	2021-41 LSNC
**Sample code (BT ref.)**	**(-)-isocolchicine / (+)-isocolchicine**
**Sample description (Customer ref.)**	**(-)-isocolchicine / (+)-isocolchicine**
**VCD-spectrometer**	ChiralIR w/ Dual*PEM*
**Report prepared by: (name / signature as needed)**	Jordan Nafie
**Report validated and signed by**	Rina K Dukor
**Date**	September 9, 2022
**RESULTS**	
Absolute Configuration of (-)-isocolchicine is (aR,7S)<br>Absolute Configuration of (+)-isocolchicine is (aS,7R)	Confidence Level: **98%**
**MEASUREMENT PARAMETERS**	
**Concentration**	7.8mg / 125uL
**Solvent**	$CDCl_3$
**Instrument Resolution**	4 cm^{-1}
**PEM setting**	1400 cm^{-1}
**Number of scans/Measurement time**	12 hours per enantiomer
**Sample cell**	BaF_2
**Path length**	100 μm
**CALCULATION DETAILS**	
**Molecular Mechanics Force Field**	**MMFF94 (Compute VOA)**
**DFT Software version**	**Gaussian '09**
**Number of conformers used for Boltzmann sum**	**12 (Mono) / 10 (Dimer) B3LYP / 6-31G(d)**
**Methodology and basis sets for DFT calculations**	**6-31G(d), cc-pVTZ / B3LYP, B3PW91 / CPCM (Chloroform)**
**Enantiomer used for calculation**	**aR,7S**
**Total calculated conformers**	**236**
**Number of low-energy conformations shown in report**	**3 (2 Mono, 1 Dimer)**

COMMENTS

The confidence level is a measure of the degree of congruence between a calculated and measured spectrum. If identical spectra are being compared the confidence level is 100%. The confidence level (CL) is not the likelihood that the assignment is correct. Rather it's a measure of quality or degree of agreement between calculated and measured spectra. With a CL of 98% for this molecule, the visual agreement between measured and calculated spectra is excellent – this is a very high confidence assignment. We saw in the analysis of colchicine enantiomers that significant dimerization was occurring in $CDCl_3$ solution. For this reason, we also explored both monomeric and dimer forms of isocolchicine in DFT calculations. The best fit in this case was a 7:1 ratio of mono : dimer – this was significantly better than mono or dimer alone. We think that dimerization is less stabilizing for the isocolchicine structure due to the different spacing (compared to colchicine) between the groups involved in dimerization – the N-H and the C=O. One monomeric form accounted for nearly 90% of the Boltzmann weight (for monomer alone), when estimating dimer content at 7:1 in favor of monomer, this comes out to just under 77% of the solution population.

17546 Bee Line Hwy | Jupiter, Florida 33478, USA
ph. 561 625.0133 | fax. 561 625.0717 | www.biotools.us

VCD Absolute Configuration Determination Report

Structure of (-)-isocolchicine:

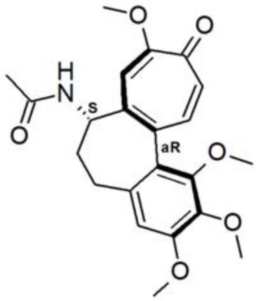

(-)-isocolchicine

Structure of (+)-isocolchicine:

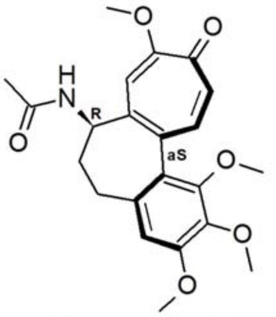

(+)-isocolchicine

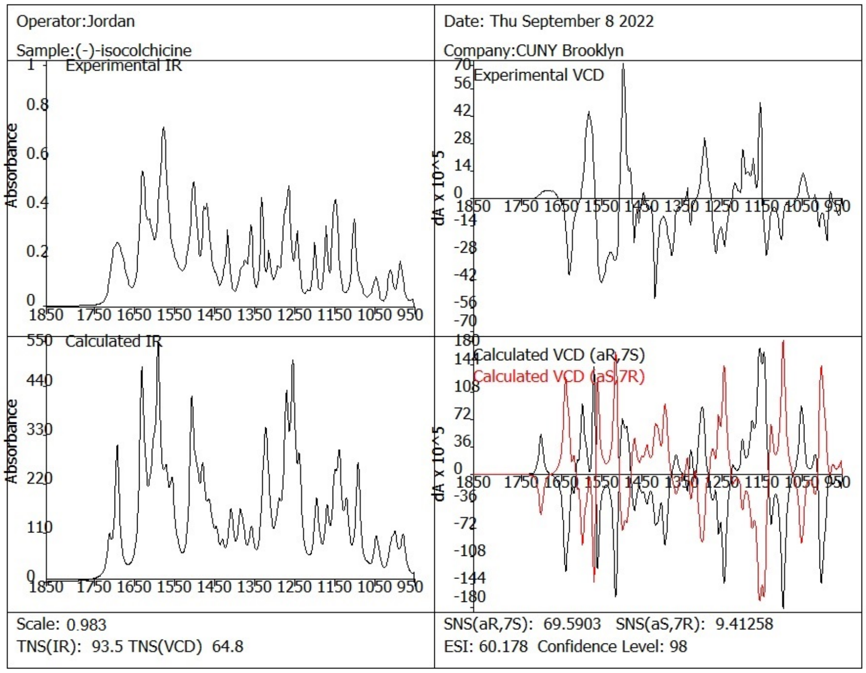

CompareVOA Results.
Please note: In this plot the frequency scaling factor is not applied.

Table 1. Numerical comparison describing the similarity in the range of 950- 1850 cm^{-1} between the calculated IR and VCD spectra for the **(aR,7S)** enantiomer (in a ratio of 7:1 mono : dimer) at the 6-31G(d) / B3LYP w/ CPCM (Chloroform) level and the observed IR and VCD spectra for **(-)-isocolchicine**.

Cal. (950-1850cm^{-1})	Numerical comparison	Observed **(-)-isocolchicine**
**(aR,7S)**	scaling factor	0.983
	IR similarity (%)	93.5
	$^{a}\Sigma$ (%)	69.5903
	$^{b}\Delta$ (%)	60.178
	Confidence Level (%)	98

$^{a}\Sigma$: single VCD similarity, gives the similarity between the calculated and observed VCD spectra.
$^{b}\Delta$: enatiomeric similarity index, gives the difference between the values of Σ for both enantiomers of a given diastereoisomer.

17546 Bee Line Hwy | Jupiter, Florida 33478, USA
ph. 561 625.0133 | fax. 561 625.0717 | www.biotools.us

Title:

VCD Absolute Configuration Determination Report

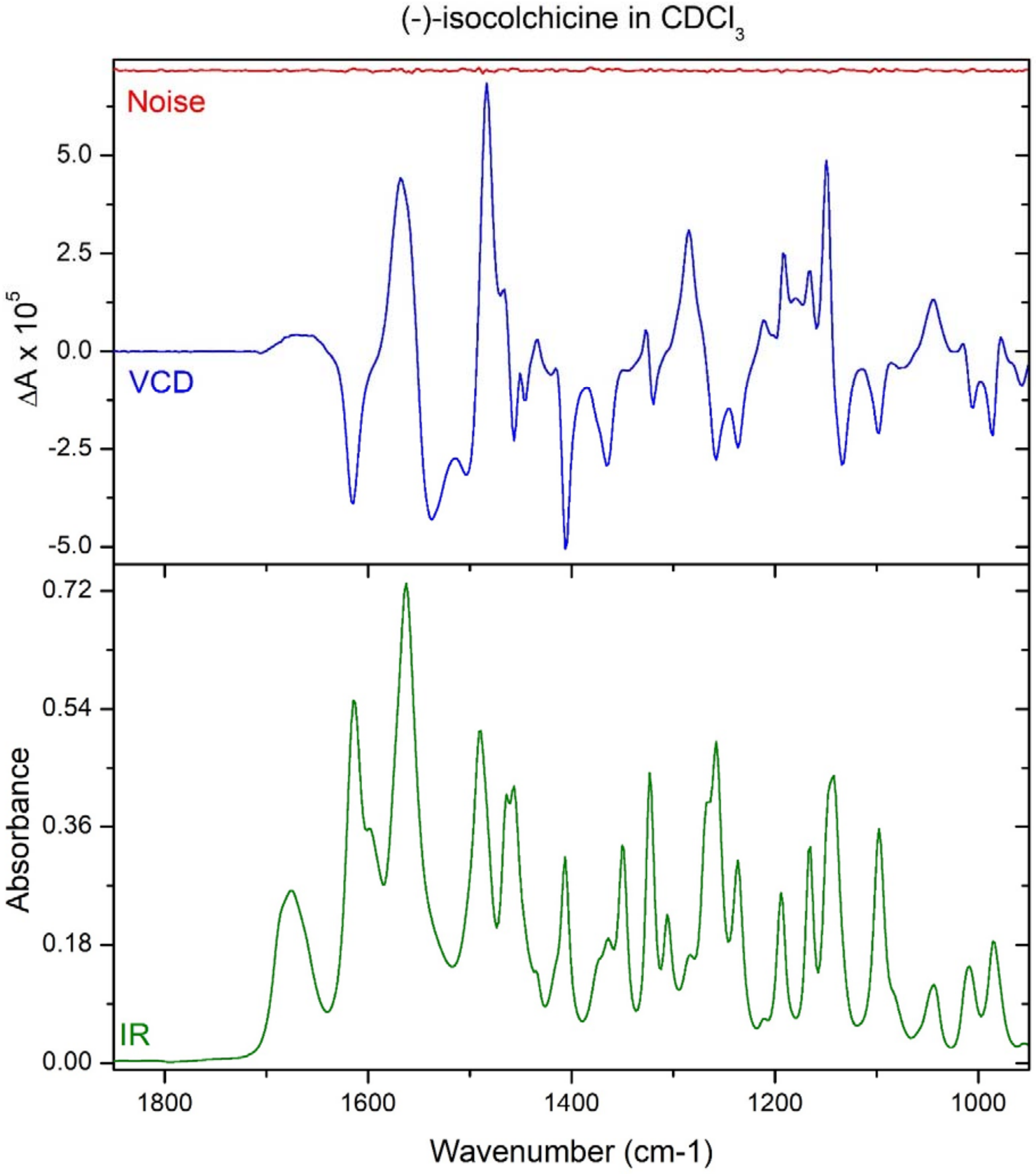

IR (lower frame) and VCD (upper frame) spectra of **(-)-isocolchicine** in $CDCl_3$; 100um path-length cell with BaF_2 windows; 12 h collection for each enantiomer; instrument optimized at 1400 cm^{-1}. Solvent subtracted IR and enantiomer subtracted VCD spectra are shown. Uppermost trace is the VCD noise spectrum.

VCD Absolute Configuration Determination Report

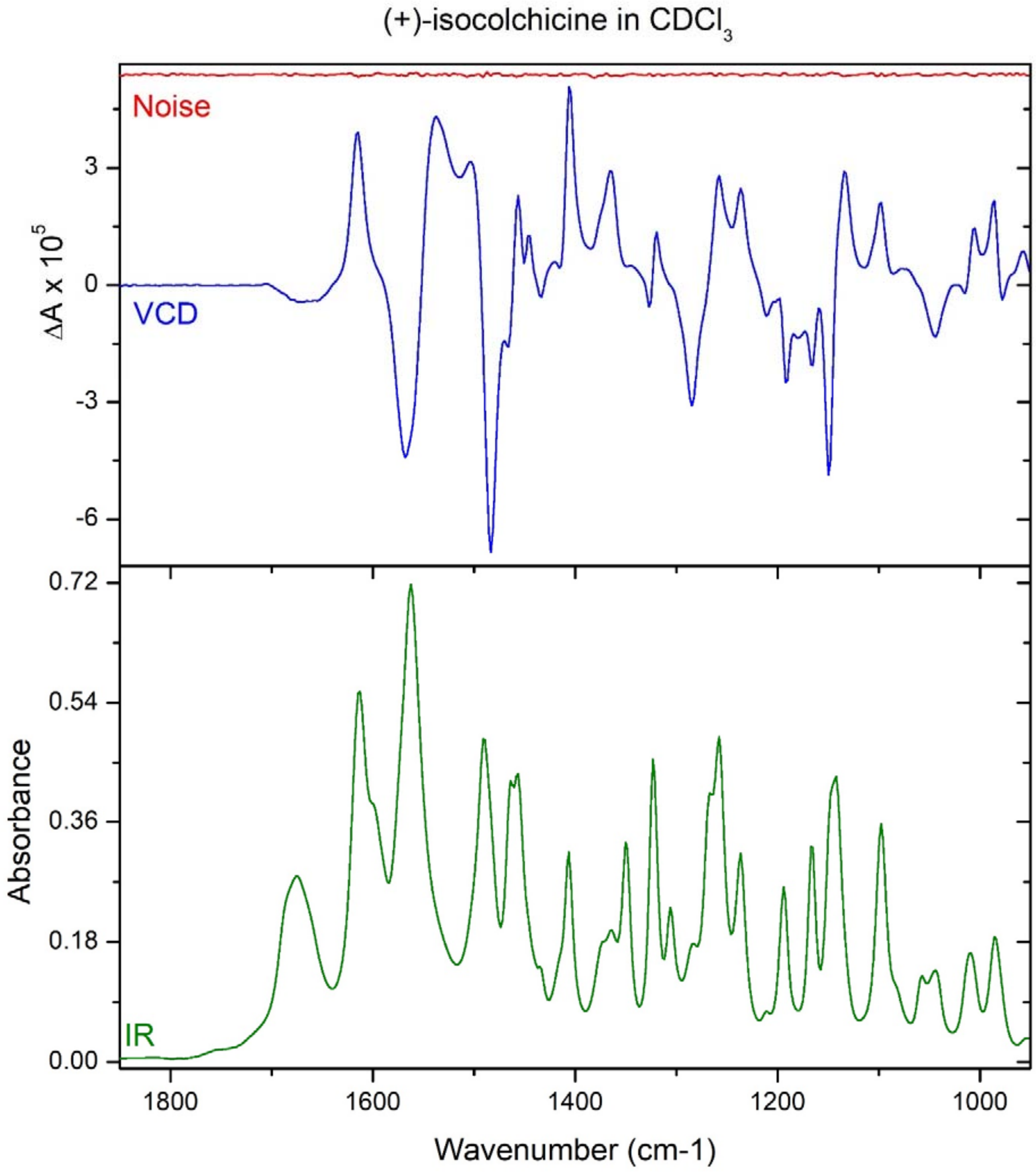

IR (lower frame) and VCD (upper frame) spectra of **(+)-isocolchicine** in $CDCl_3$; 100um path-length cell with BaF_2 windows; 12 h collection for each enantiomer; instrument optimized at 1400 cm^{-1}. Solvent subtracted IR and enantiomer subtracted VCD spectra are shown. Uppermost trace is the VCD noise spectrum.

Title:

VCD Absolute Configuration Determination Report

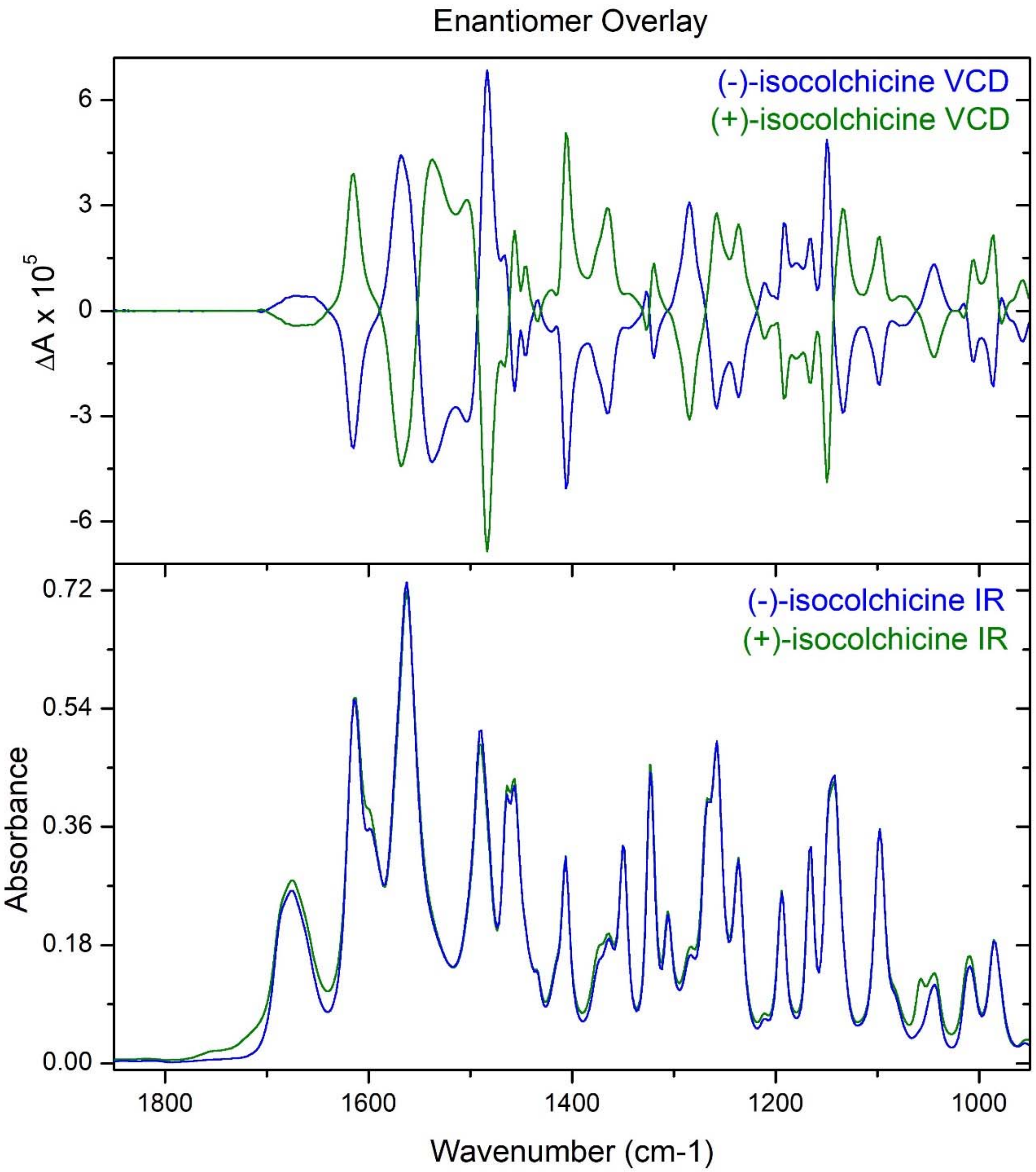

Overlay of both enantiomers, **(-)-isocolchicine** and **(+)-isocolchicine**. The IR are nearly identical as expected. The VCD are mirror images due to the half difference processing (E1 – E2) / 2.

Title:

VCD Absolute Configuration Determination Report

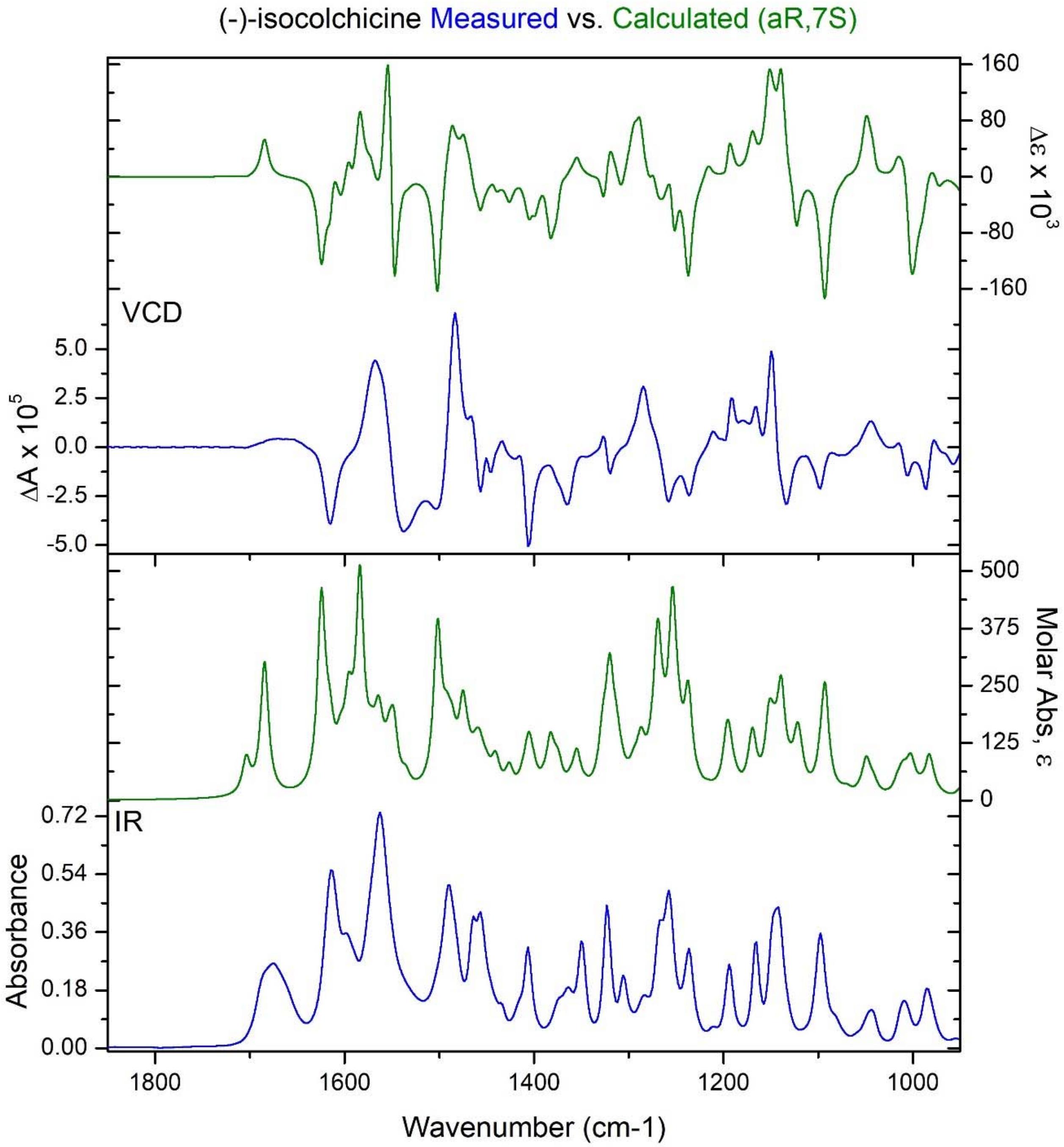

IR (lower frame) and VCD (upper frame) spectra observed for **(-)-isocolchicine** (left axes) compared with Boltzmann-averaged spectra of the calculated conformations for the **(aR,7S)** configuration, (right axes).

VCD Absolute Configuration Determination Report

Lowest energy conformers (2 mono / 1 dimer) - (aR,7S) Configuration:

76.9%

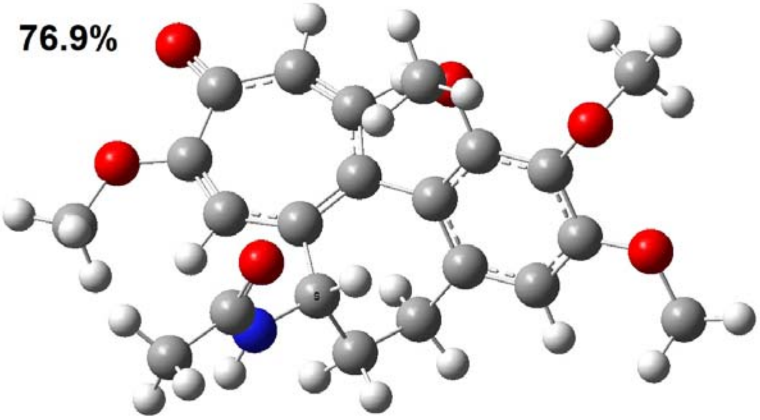

2.5%

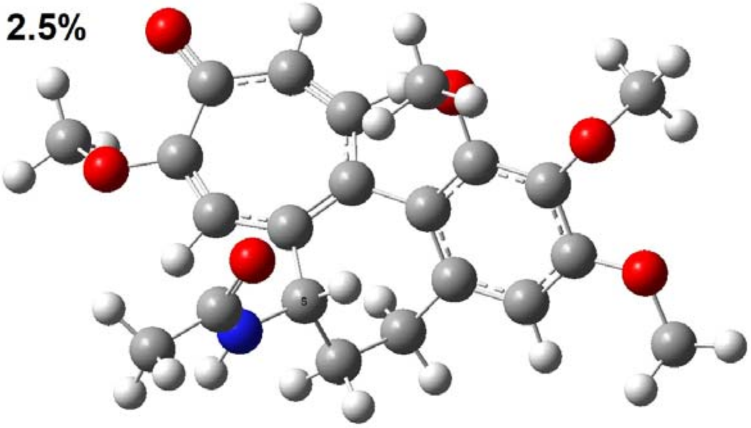

8.3%

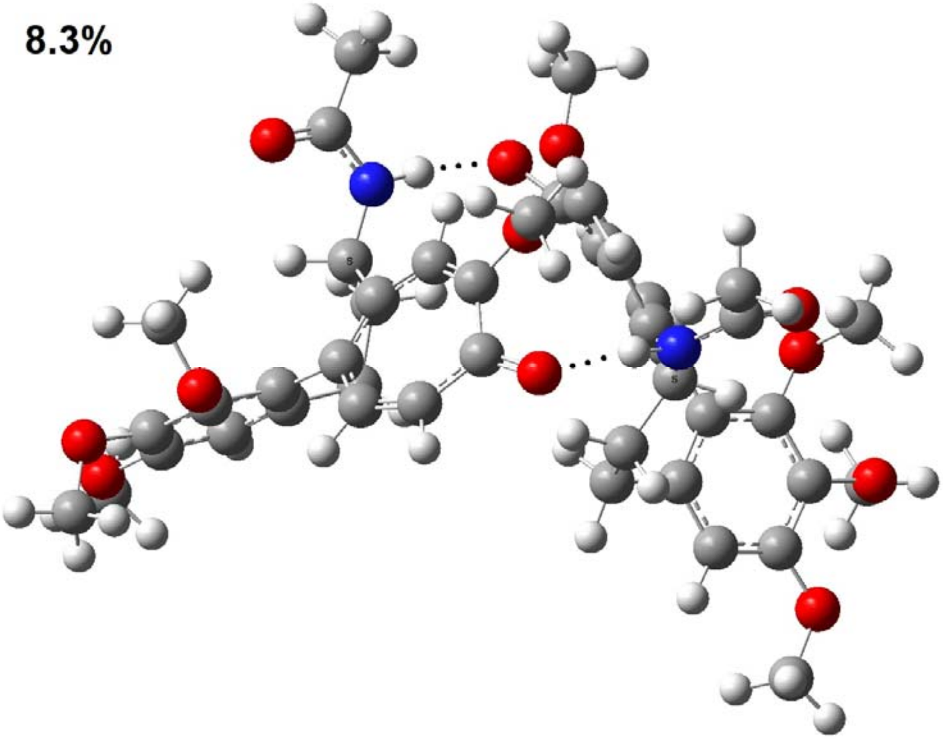

17546 Bee Line Hwy │ Jupiter, Florida 33478, USA
ph. 561 625.0133 │ fax. 561 625.0717 │ www.biotools.us

Title:

VCD Absolute Configuration Determination Report

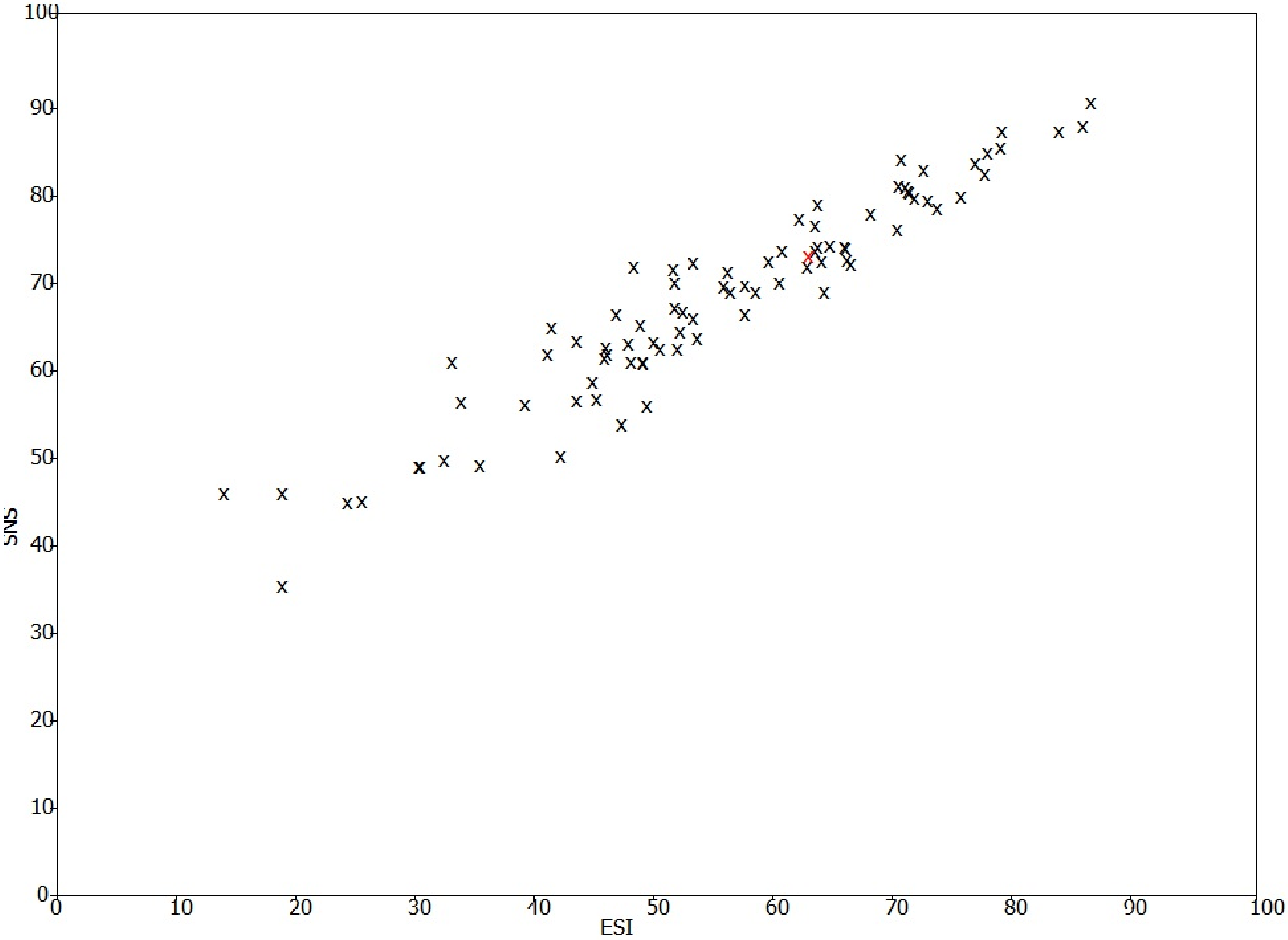

Plot of ESI (similarity of correct enantiomer minus incorrect enantiomer to calculated) vs SNS (overall similarity of correct enantiomer to calculated) for a library of correct assignments verified independently by X-Ray other method (Black X marks). Red X is **(-)-isocolchicine**. Upper right corner is the strongest results, lower left is the weakest.

17546 Bee Line Hwy | Jupiter, Florida 33478, USA
ph. 561 625.0133 | fax. 561 625.0717 | www.biotools.us
