# Supplementary material for: Deconstruction of Desacetamidocolchicine’s B Ring Reveals a Class 3 Atropisomeric AC Ring with Tubulin Binding Properties
Source: J Org Chem. 2025 May 27;90(22):7246–58. doi: 10.1021/acs.joc.5c00284 (PMC12150326; doi:10.1021/acs.joc.5c00284)
Supplement: Supplementary file 3 [file jo5c00284_si_003.zip › VCD Reports/(+) and (-) Colchicine VCD Report.pdf]

Title:

VCD Absolute Configuration Determination Report

GENERAL INFORMATION	
**Customer**	CUNY Brooklyn
**Sales Order Number**	2021-41 LSNC
**Sample code (BT ref.)**	**(-)-colchicine / (+)-colchicine**
**Sample description (Customer ref.)**	**(-)-colchicine / (+)-colchicine**
**VCD-spectrometer**	ChiralIR w/ Dual*PEM*
**Report prepared by: (name / signature as needed)**	Jordan Nafie
**Report validated and signed by**	Rina K Dukor
**Date**	September 12, 2022
**RESULTS**	
Absolute Configuration of (-)-Colchicine is (aR,7S)<br>Absolute Configuration of (+)-Colchicine is (aS,7R)	Confidence Level: **99%**
**MEASUREMENT PARAMETERS**	
**Concentration**	7.4mg / 180uL
**Solvent**	$CDCl_3$
**Instrument Resolution**	4 cm^{-1}
**PEM setting**	1400 cm^{-1}
**Number of scans/Measurement time**	6 hours per enantiomer
**Sample cell**	BaF_2
**Path length**	100 µm
**CALCULATION DETAILS**	
**Molecular Mechanics Force Field**	**MMFF94 (Compute VOA)**
**DFT Software version**	**Gaussian '09**
**Number of conformers used for Boltzmann sum**	**22 Dimer / 17 Mono (6-31G(d) / B3LYP)**
**Methodology and basis sets for DFT calculations**	**6-31G(d / B3LYP / CPCM (Chloroform)**
**Enantiomer used for calculation**	**aR,7S**
**Total calculated conformers**	**138**
**Number of low-energy conformations shown in report**	**4 Dimer / 4 Mono**

COMMENTS

The confidence level is a measure of the degree of congruence between a calculated and measured spectrum. If identical spectra are being compared the confidence level is 100%. The confidence level (CL) is not the likelihood that the assignment is correct. Rather it's a measure of quality or degree of agreement between calculated and measured spectra. With a CL of 99% for this molecule, the visual agreement between measured and calculated spectra is excellent – this is a very high confidence assignment. Due to the possibility of dimerization occurring in deuterochloroform solution, both the monomer and the dimer forms of (-)-colchicine were calculated. Based on the experimental IR and VCD, it was clear that significant dimerization was occurring. A 1:1 average of the DFT monomer:dimer IR and VCD spectra increased the similarity to the experimental compared to either monomer or dimer alone. This is illustrated both in CompareVOA plots as well as stack plots of the individual and combined spectra. The first DFT method used (6-31G(d) / B3LYP) was sufficient to produce very high neighborhood similarity values for this compound. This is an updated report, which includes the experimental data for (+)-colchicine as well as the DFT calculated spectra for the diastereomer (aS,7S) for which we do not have access to a sample of. This data was constructed in an analogous fashion to the original DFT data, as a 1:1 ratio of monomer : dimer for (aS,7S) in each case. There are vast differences in the diastereomer VCD spectra as was anticipated. Boltzmann weight % shown with conformers later in the report accounts for the 1:1 ratio of monomer : dimer. It should be noted that the dimer form is difficult to work with in molecular modeling, so some conformers were likely missed – however we feel confident that the most important (lowest energy) forms were indeed found since the DFT spectra match very well to the experimental.

Title:

VCD Absolute Configuration Determination Report

Structure of (-)-colchicine:

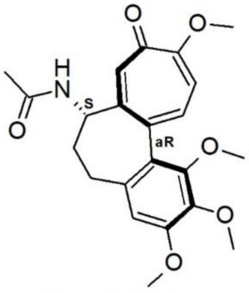

(-)-colchicine

Structure of (+)-colchicine:

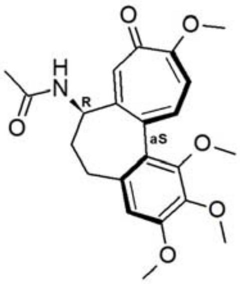

(+)-colchicine

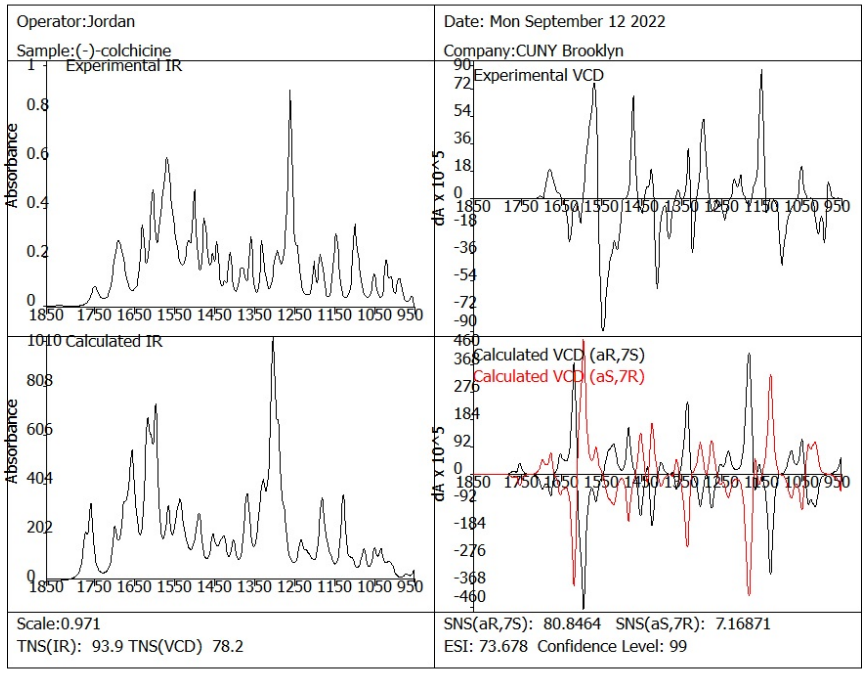

CompareVOA Results.
Please note: In this plot the frequency scaling factor is not applied.
This is using the DFT data of a 1:1 ratio of Dimer : Mono

Table 1. Numerical comparison describing the similarity in the range of 950- 1850 cm^{-1} between the calculated IR and VCD spectra for the **(aR,7S)** enantiomer at the 6-31G(d) / B3LYP w/ CPCM (Chloroform) level (1:1 ratio of dimer : mono) and the observed IR / VCD spectra for **(-)-colchicine**.

Cal. (950-1850cm^{-1})	Numerical comparison	Observed **(-)-colchicine**
**(aR,7S)**	scaling factor	0.971
	IR similarity (%)	93.9
	$^{a}\sum$ (%)	80.8464
	$^{b}\Delta$ (%)	73.678
	Confidence Level (%)	99

$^{a}\sum$: single VCD similarity, gives the similarity between the calculated and observed VCD spectra.
$^{b}\Delta$: enatiomeric similarity index, gives the difference between the values of $\sum$ for both enantiomers of a given diastereoisomer.

Title:

VCD Absolute Configuration Determination Report

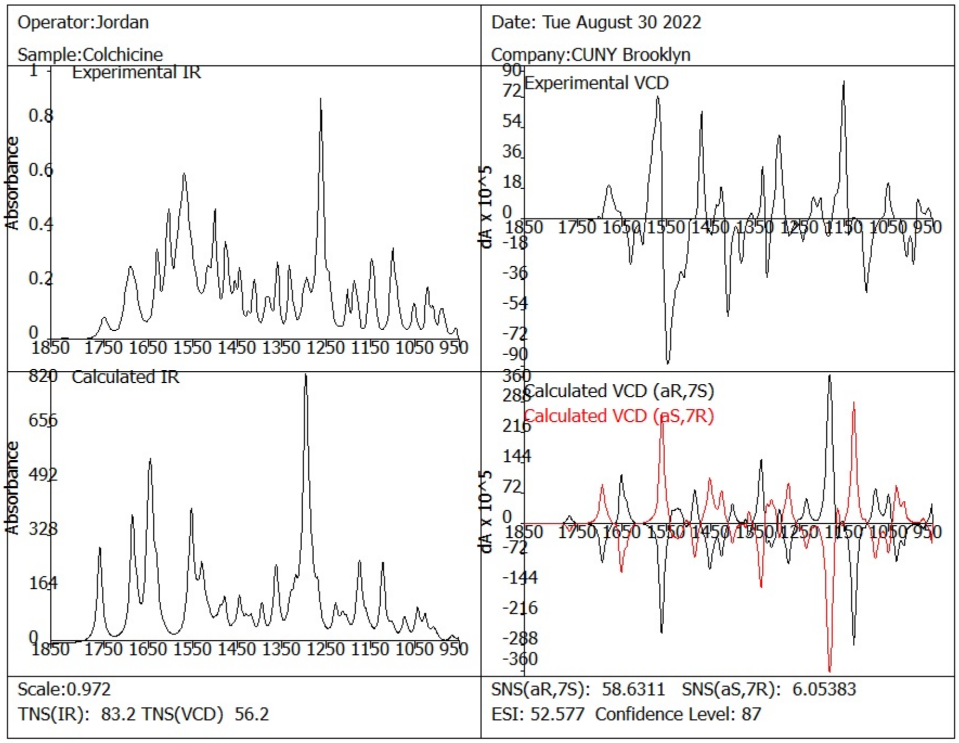

Left: (-)-colchicine experimental vs. DFT of monomer only
Lower numbers across the board.

Right: (-)-colchicine experimental vs. DFT of dimer only
Closer match overall than monomer only but not as good as the 1:1 ratio.

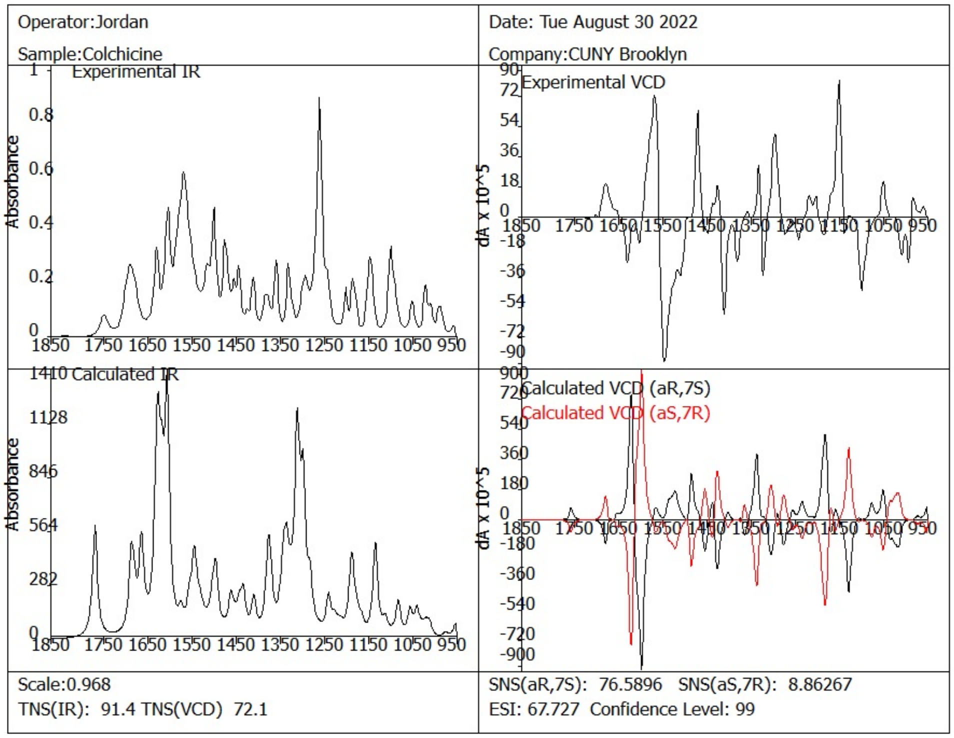

17546 Bee Line Hwy | Jupiter, Florida 33478, USA
ph. 561 625.0133 | fax. 561 625.0717 | www.biotools.us

Title:

VCD Absolute Configuration Determination Report

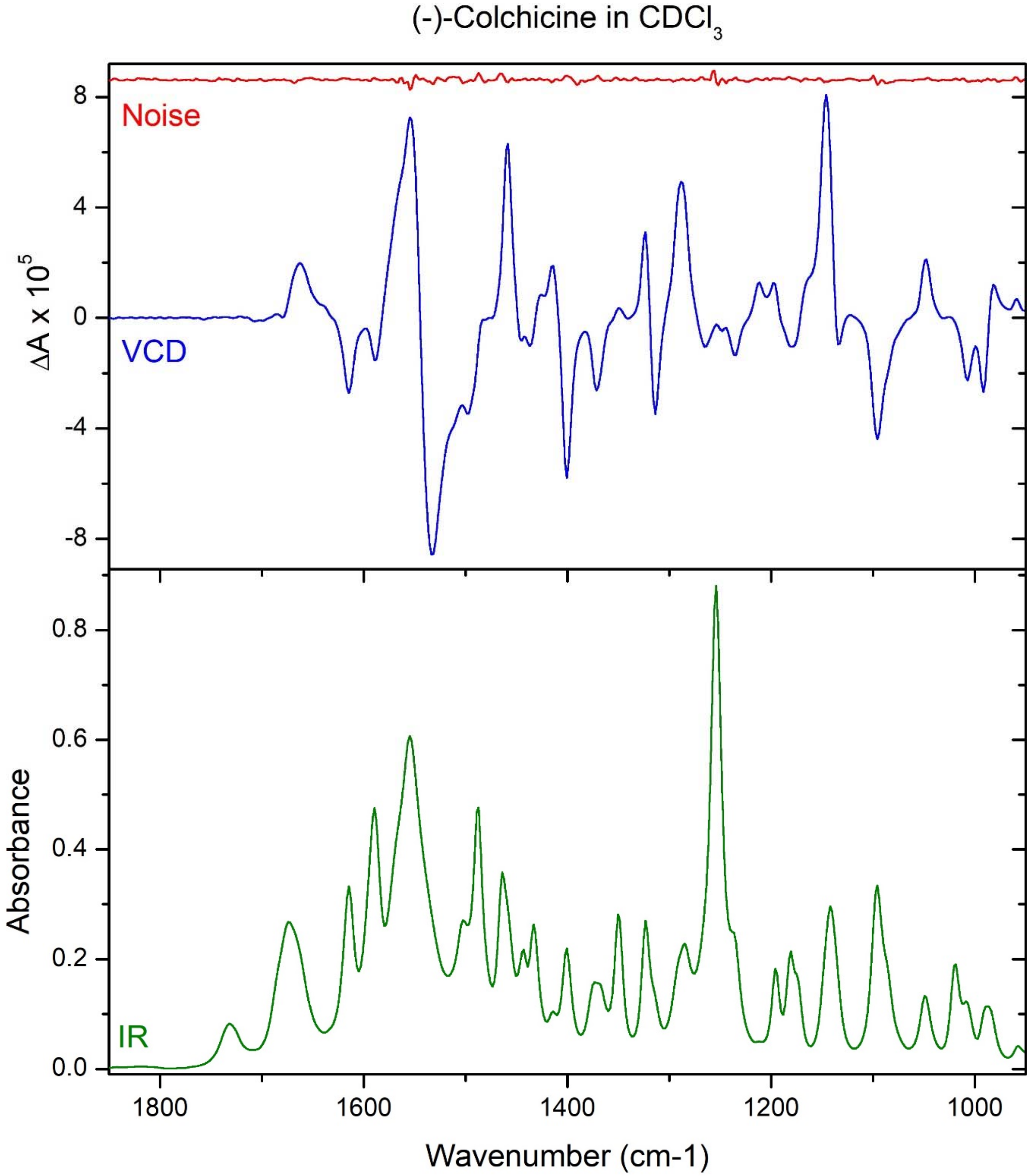

IR (lower frame) and VCD (upper frame) spectra of **(-)-colchicine** in $CDCl_3$; 100um path-length cell with BaF_2 windows; 6 h collection per enantiomer; instrument optimized at 1400 cm^{-1}. Solvent subtracted IR and enantiomer subtracted VCD spectra are shown. Uppermost trace is the VCD noise spectrum.

17546 Bee Line Hwy │ Jupiter, Florida 33478, USA
ph. 561 625.0133 │ fax. 561 625.0717 │ www.biotools.us

Title:

VCD Absolute Configuration Determination Report

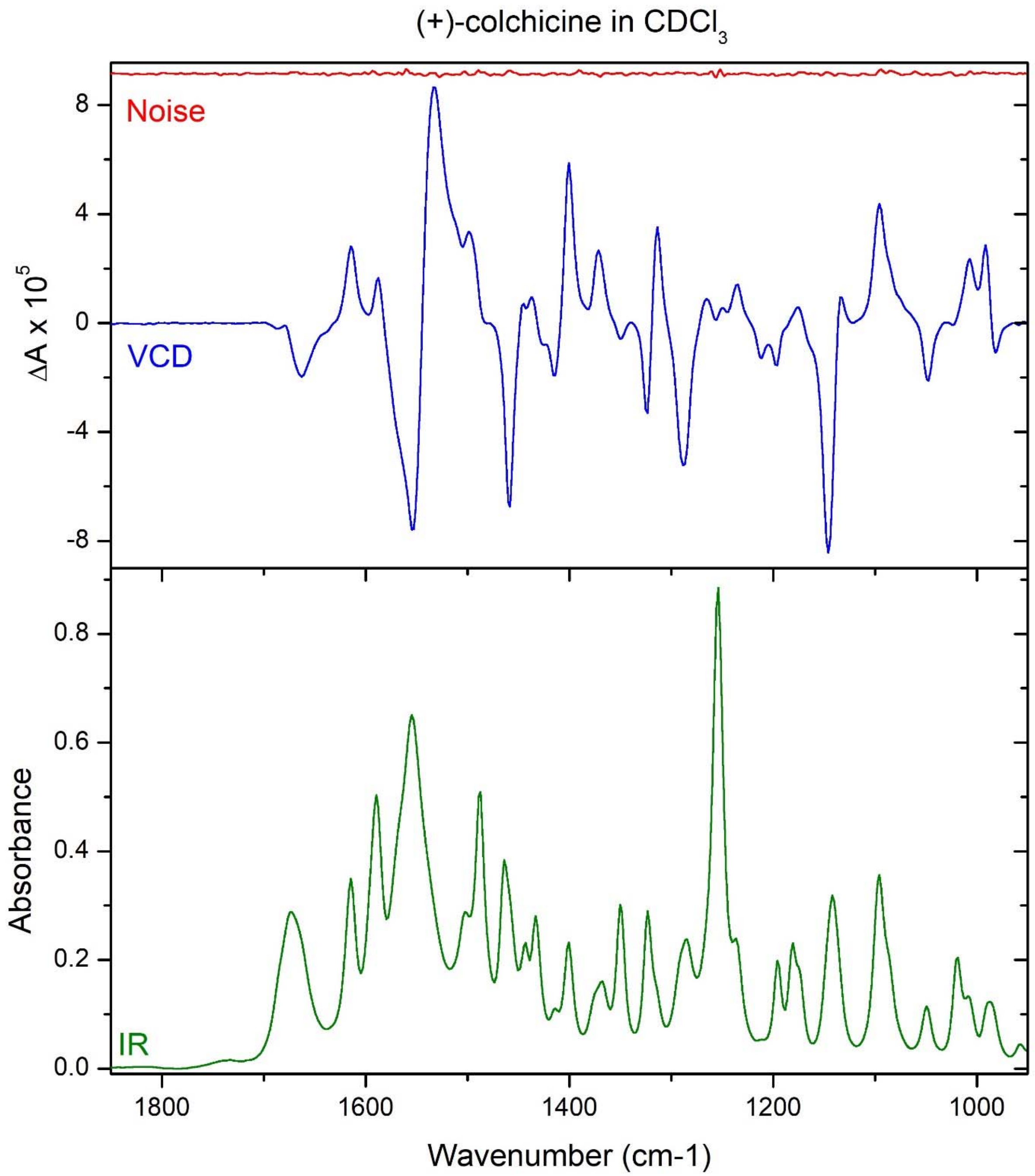

IR (lower frame) and VCD (upper frame) spectra of **(+)-colchicine** in $CDCl_3$; 100um path-length cell with BaF_2 windows; 6 h collection per enantiomer; instrument optimized at 1400 cm^{-1}. Solvent subtracted IR and enantiomer subtracted VCD spectra are shown. Uppermost trace is the VCD noise spectrum.

17546 Bee Line Hwy │ Jupiter, Florida 33478, USA
ph. 561 625.0133 │ fax. 561 625.0717 │ www.biotools.us

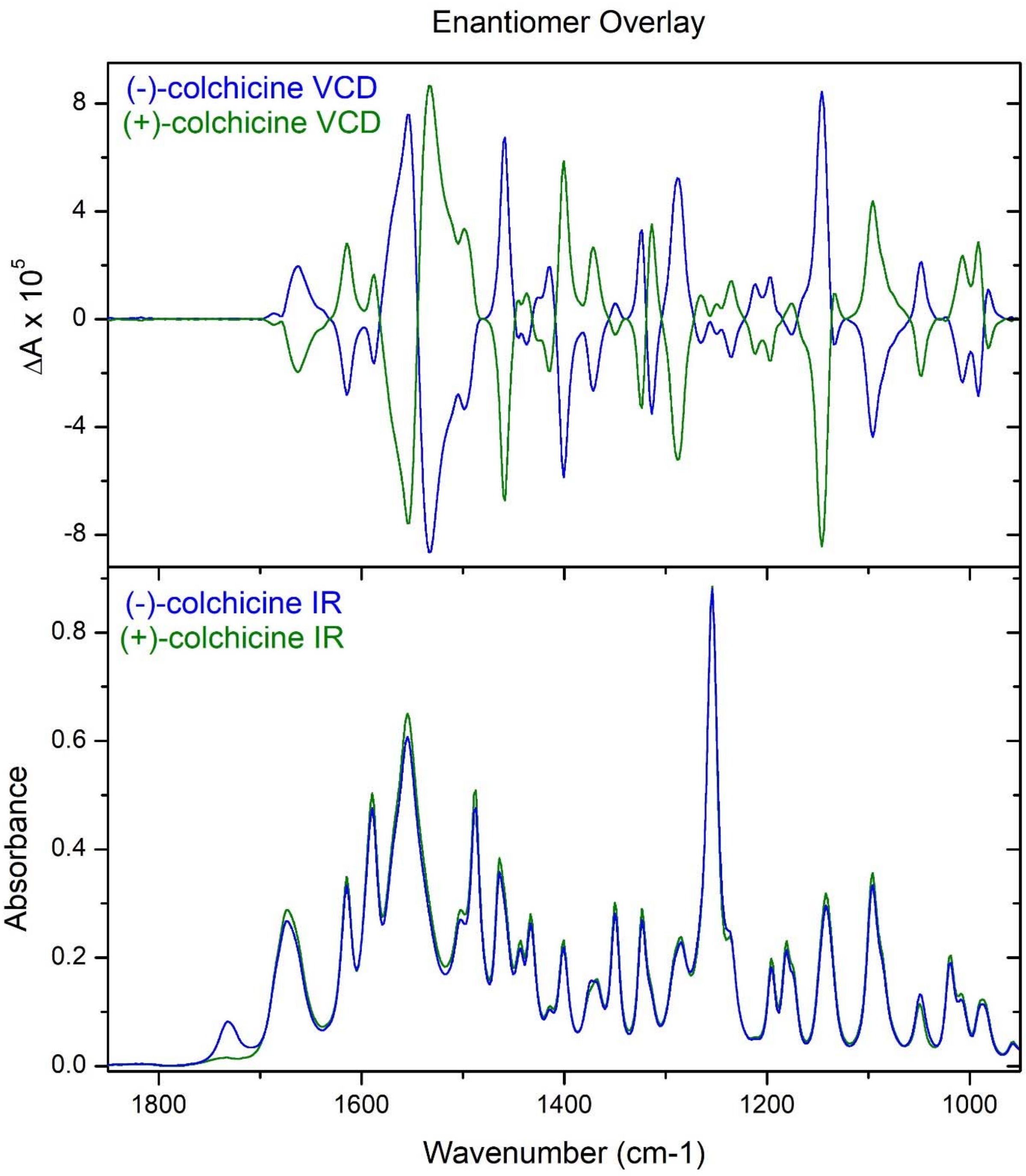

Overlay of both enantiomers, **(-)-colchicine** and **(+)-colchicine**. The IR are nearly identical as expected. The VCD are mirror images due to the half difference processing (E1 – E2) / 2.

Title:

VCD Absolute Configuration Determination Report

(-)-Colchicine Measured vs. Calculated (aR,7S)

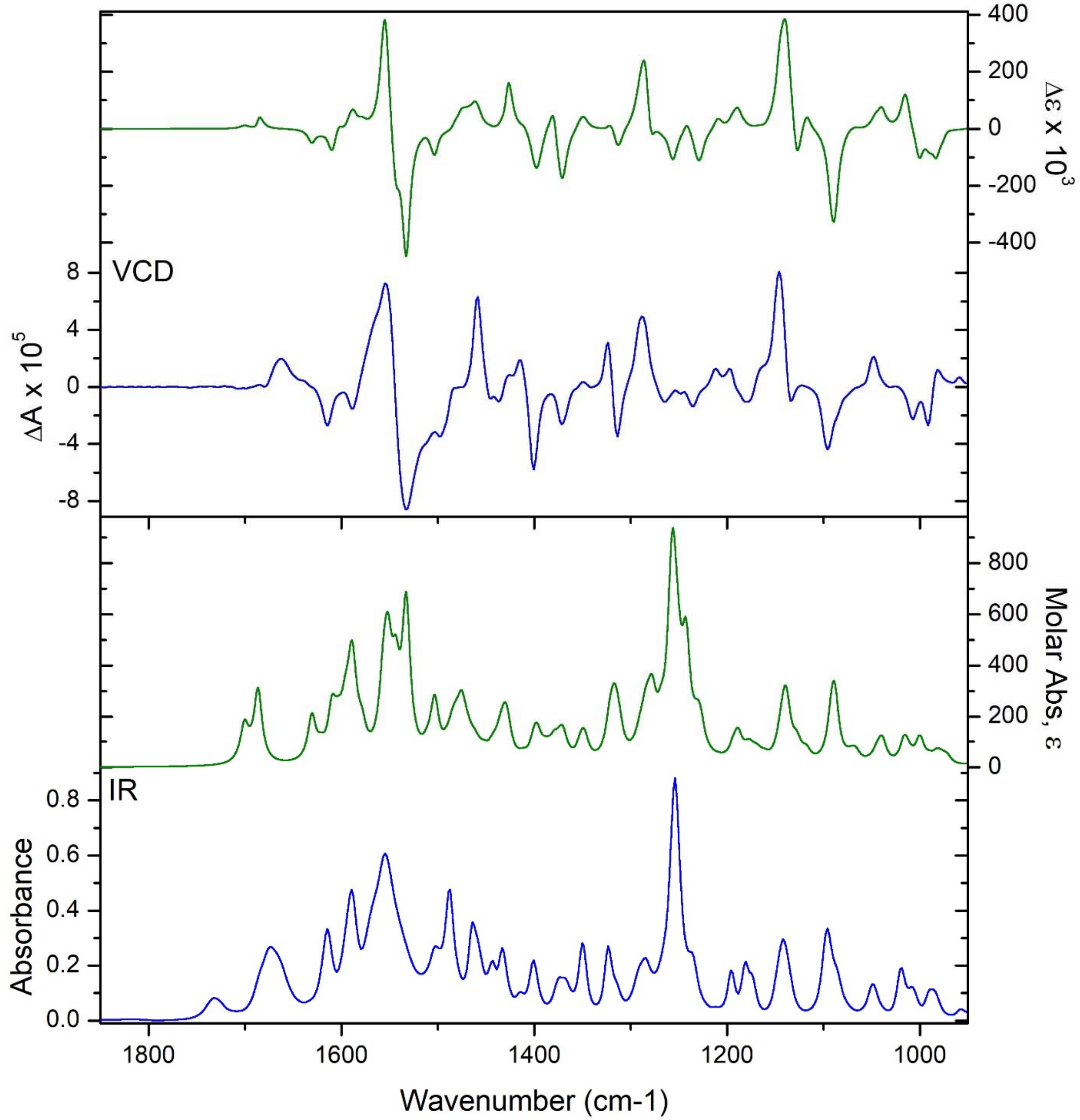

IR (lower frame) and VCD (upper frame) spectra observed for **(-)-colchicine** (left axes) compared with Boltzmann-averaged spectra of the calculated conformations (with a 1:1 ratio of monomer : dimer) for the **(aR,7S)** configuration, (right axes).

Title:

VCD Absolute Configuration Determination Report

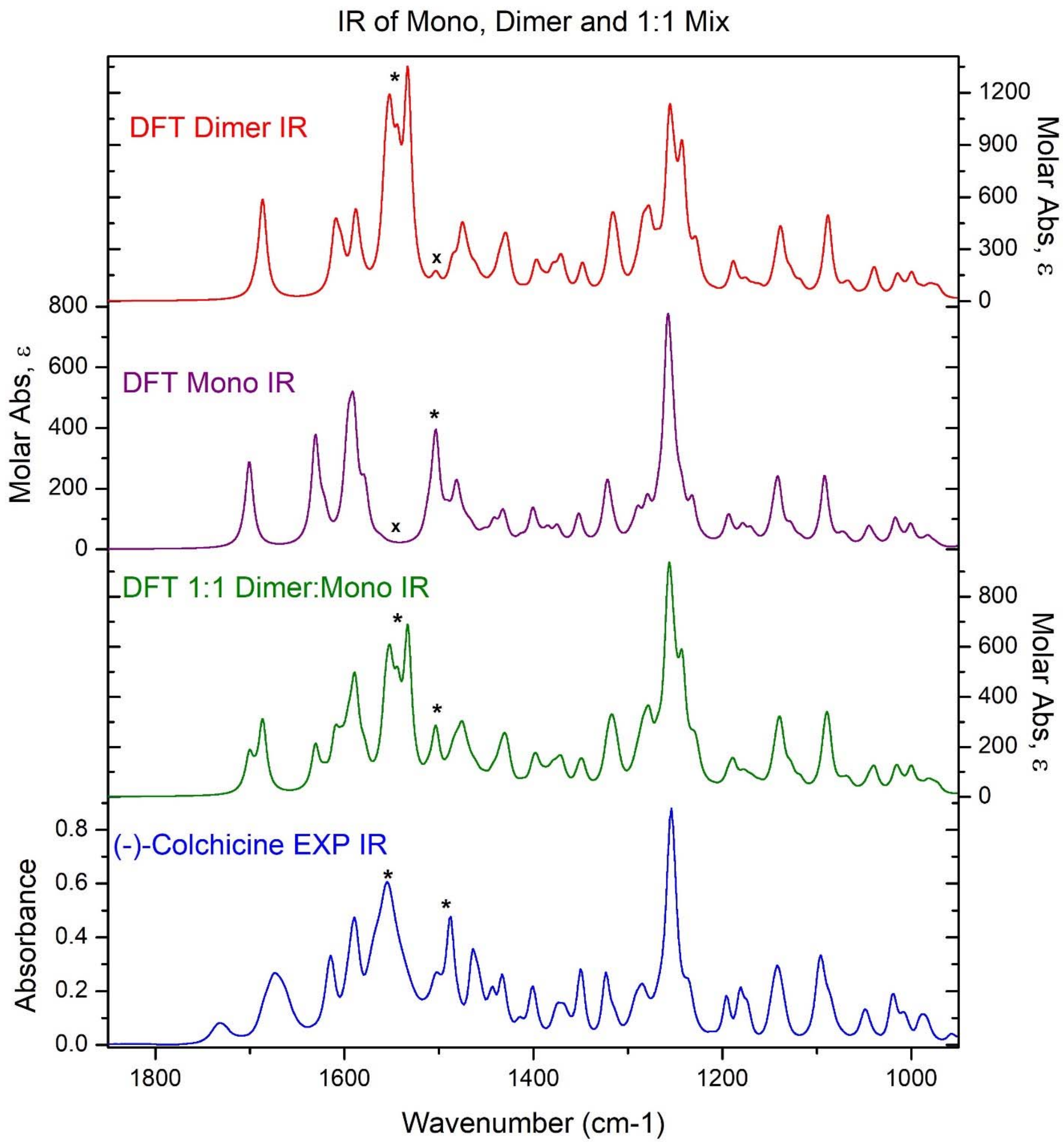

Stack plot of IR spectra for **experimental** (-)-colchicine against DFT spectra of a **1:1 ratio of dimer : monomer**, **monomer only** and **dimer only**. Asterisk indicates peaks which appear in either mono or dimer, but not both and are also found in the experimental. X indicates peaks missing in either mono or dimer spectra.

Title:

VCD Absolute Configuration Determination Report

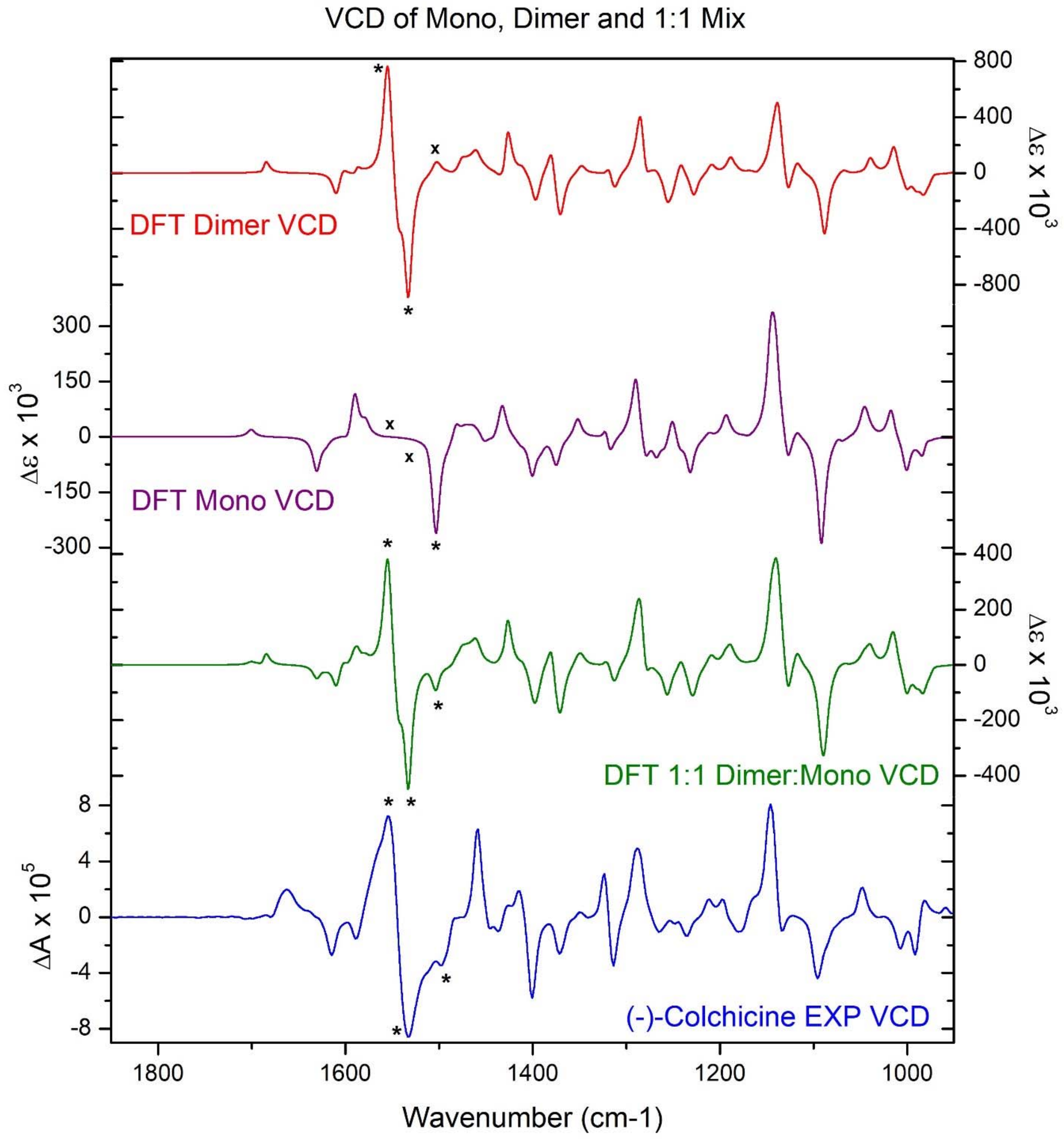

Stack plot of VCD spectra for **experimental** (-)-colchicine against DFT spectra of a **1:1 ratio of dimer** : **monomer**, **monomer only** and **dimer only**. Asterisk indicates peaks which appear in either mono or dimer, but not both and are also found in the experimental. X indicates peaks missing in either mono or dimer spectra.

VCD Absolute Configuration Determination Report

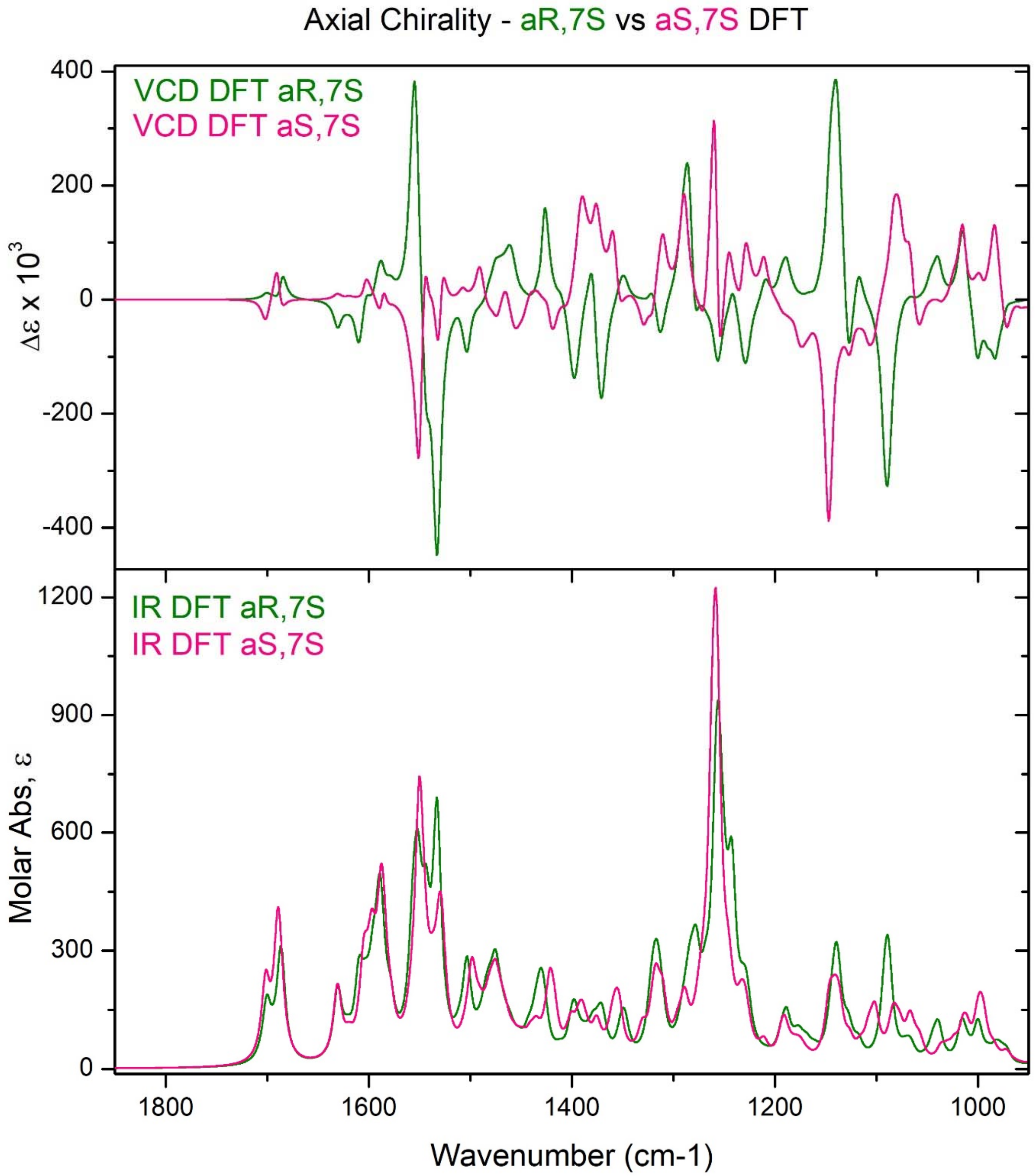

DFT spectra for two diastereomers of colchicine **(aR,7S)** and **(aS,7S)** differing only in axial chirality. The (aS,7S) data was produced using a ratio of 1:1 monomer (aS,7S) to dimer (aS,7S) in an analogous fashion to the original calculated data. The IR have some small differences, with the VCD being vastly different. This shows the strong influence of axial chirality in the overall VCD.

Title:

VCD Absolute Configuration Determination Report

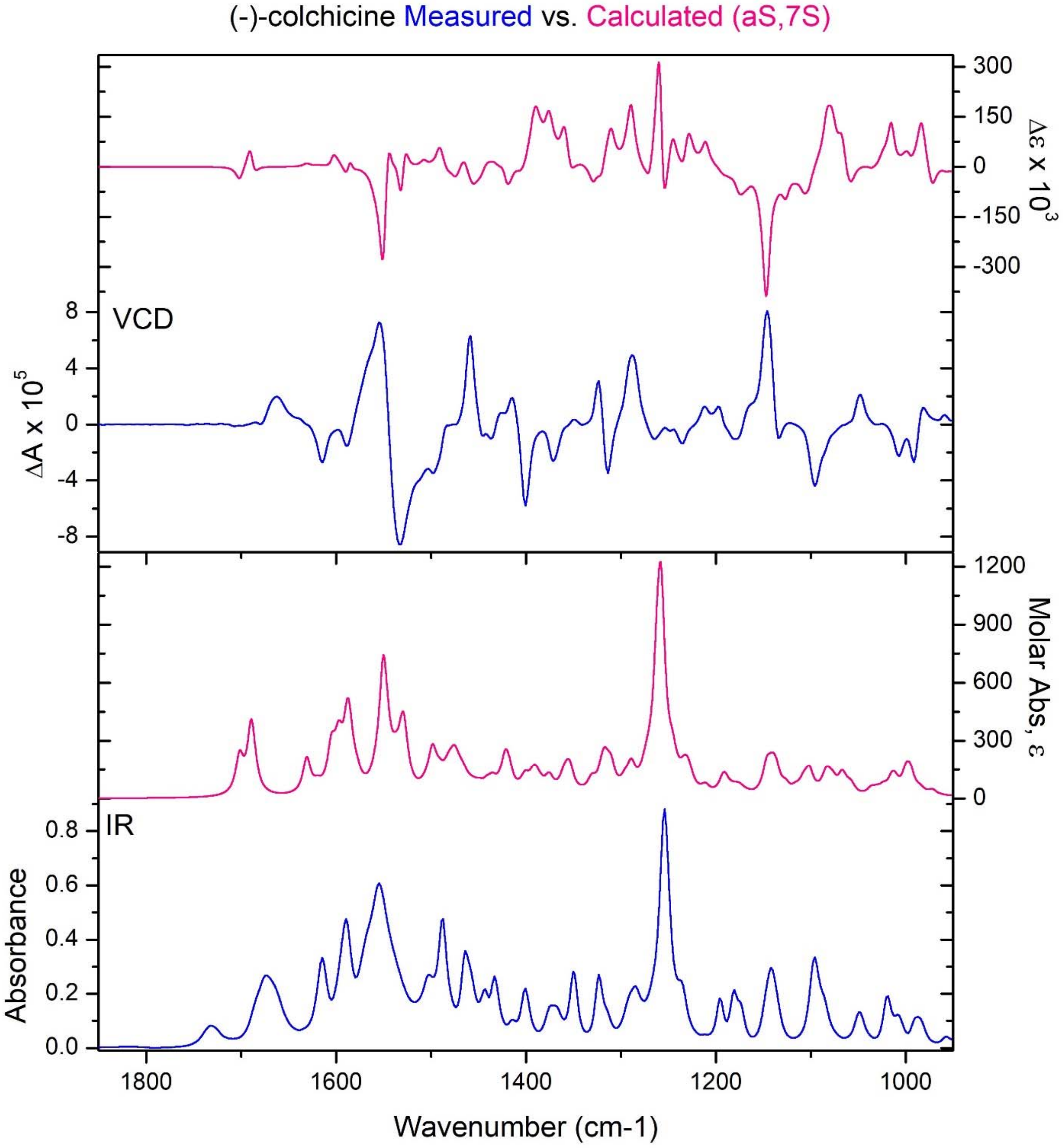

IR (lower frame) and VCD (upper frame) spectra **observed** for **(-)-colchicine** (left axes) compared with Boltzmann-averaged spectra of the **calculated** conformations for the **(aS,7S)** configuration, (right axes). While the IR is reasonable, the VCD is clearly *not* a good match, which is consistent with our assignment.

Four lowest energy conformers (of 17 in Boltzmann average) - (aR,7S) Configuration Mono:

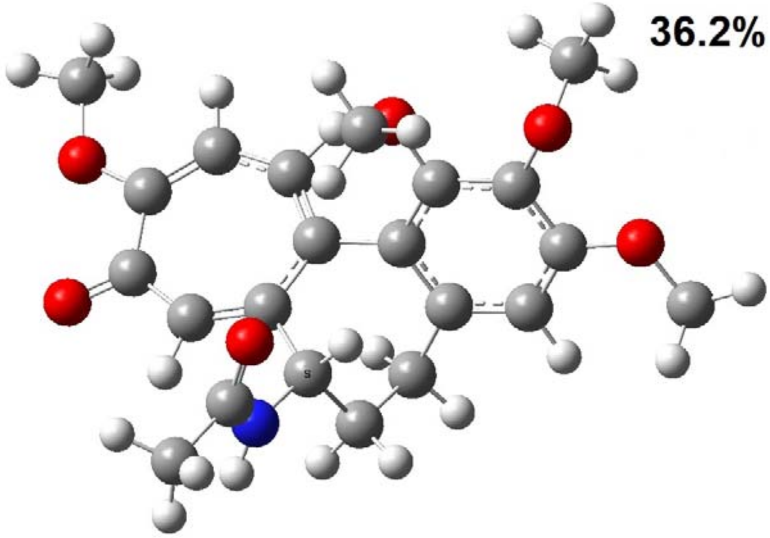

36.2%

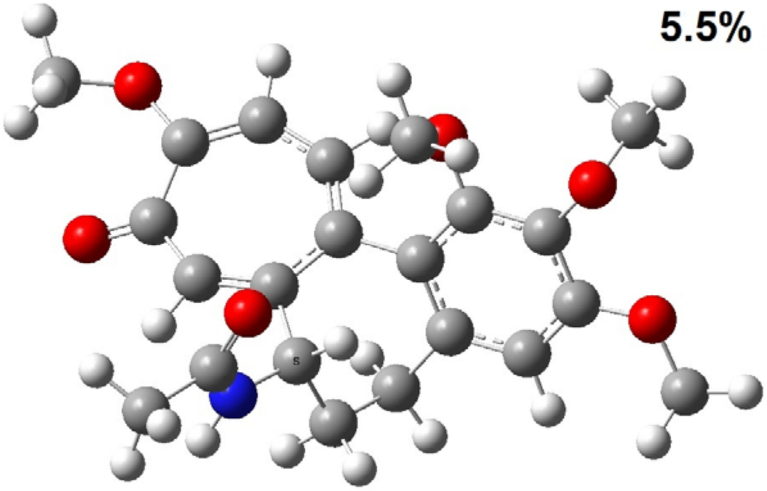

5.5%

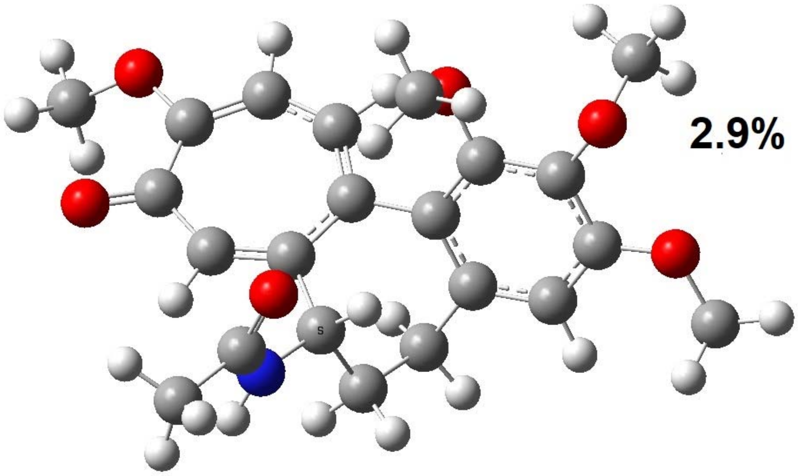

2.9%

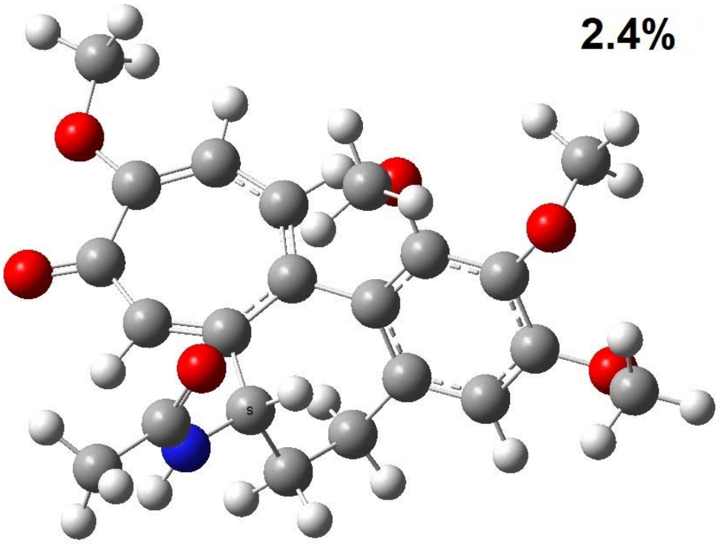

2.4%

17546 Bee Line Hwy │ Jupiter, Florida 33478, USA
ph. 561 625.0133 │ fax. 561 625.0717 │ www.biotools.us

Four lowest energy conformers (of 22 in Boltzmann average) - (aR,7S) Configuration Dimer:

33.1%

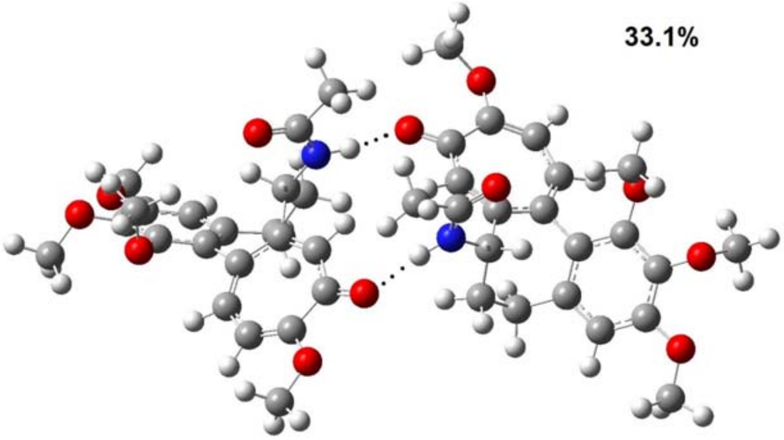

4.6%

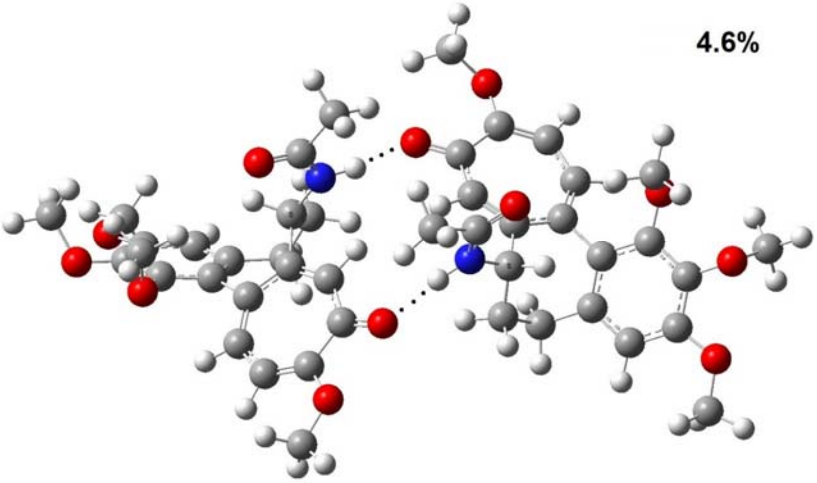

4.1%

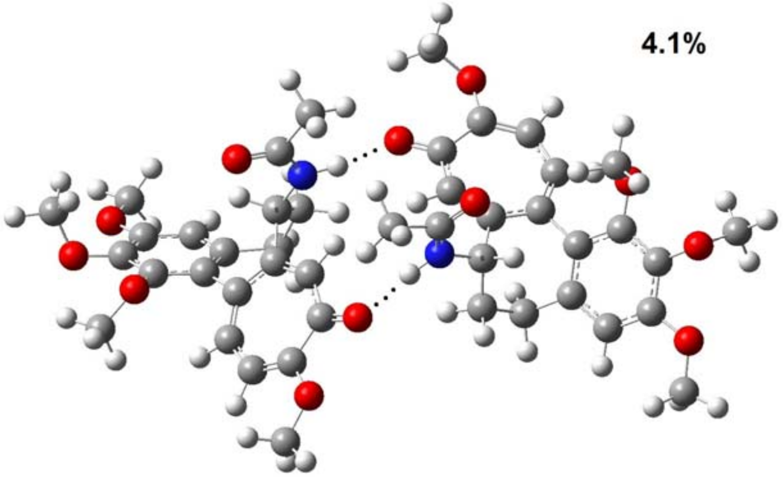

3.9%

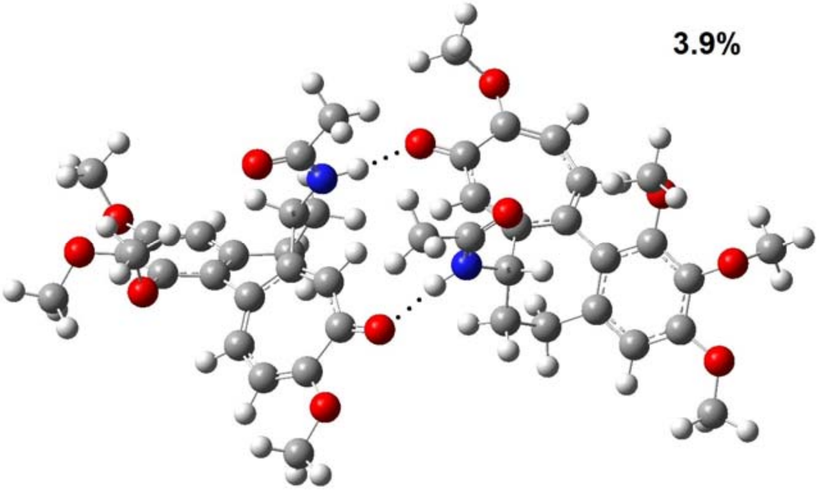

VCD Absolute Configuration Determination Report

Four lowest energy conformers (of 23 in Boltzmann average) - (aS,7S) Configuration Mono:

34.0%

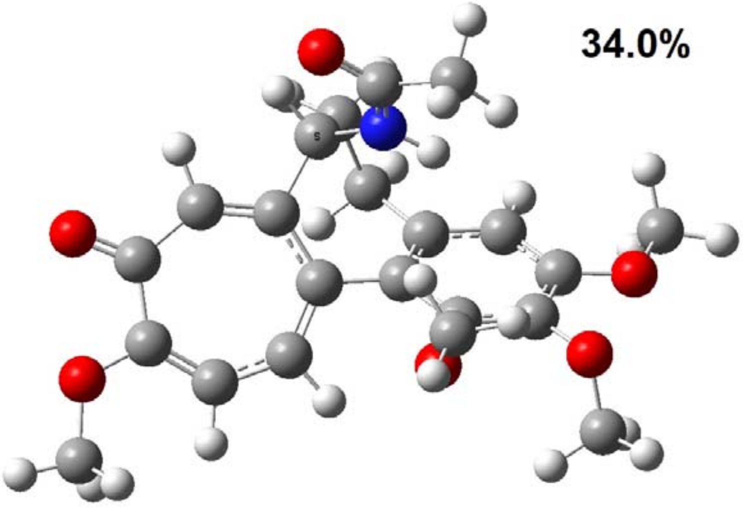

5.6%

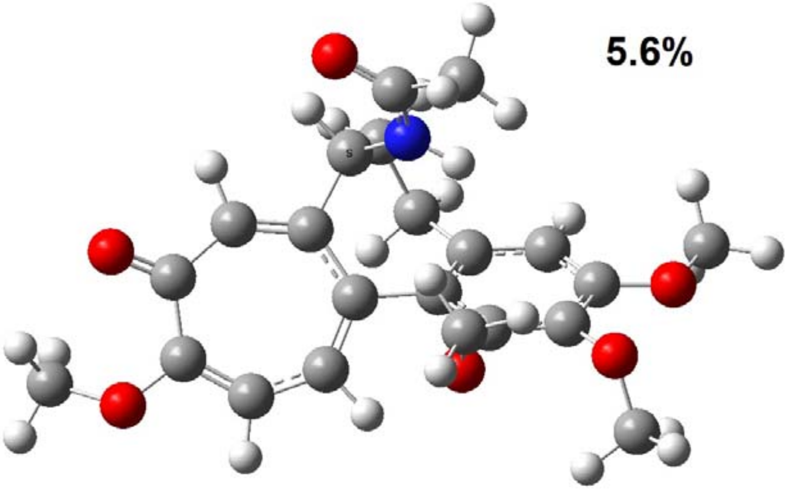

2.5%

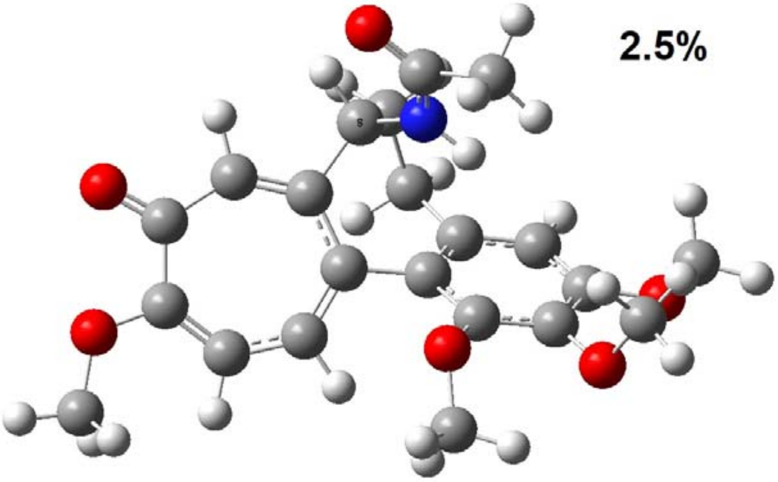

1.9%

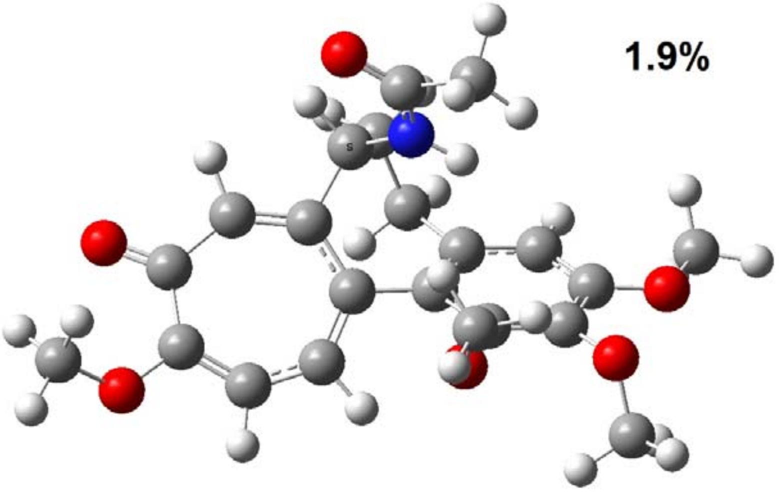

17546 Bee Line Hwy | Jupiter, Florida 33478, USA
ph. 561 625.0133 | fax. 561 625.0717 | www.biotools.us

VCD Absolute Configuration Determination Report

Four lowest energy conformers (of 23 in Boltzmann average) - (aS,7S) Configuration Dimer:

34.2%

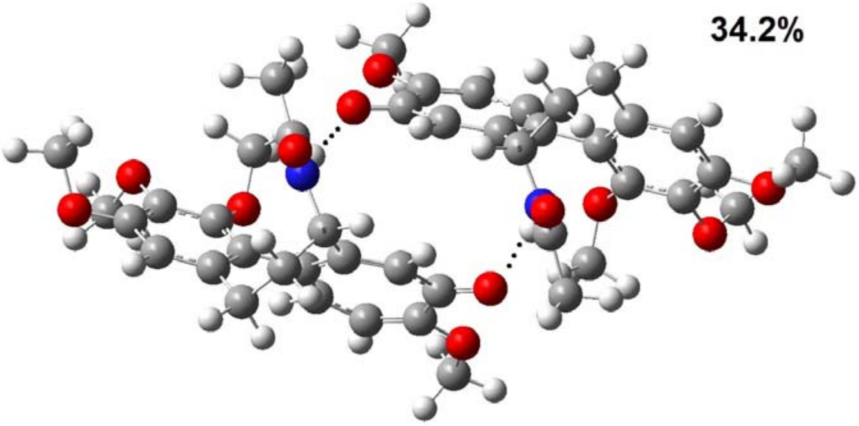

4.6%

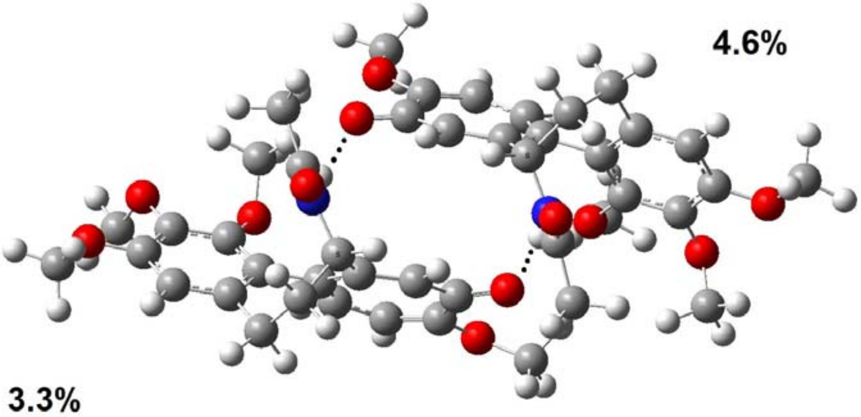

3.3%

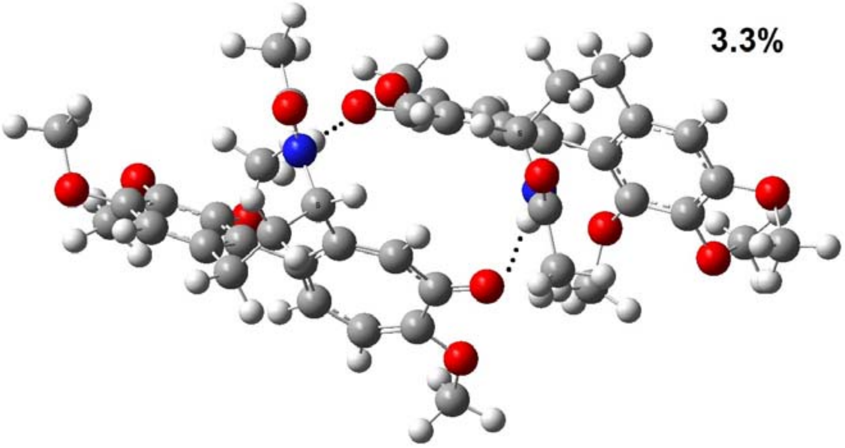

1.7%

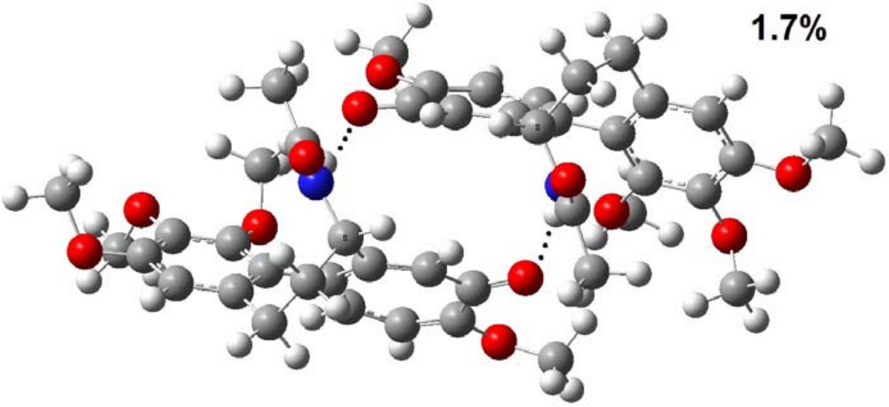

17546 Bee Line Hwy | Jupiter, Florida 33478, USA
ph. 561 625.0133 | fax. 561 625.0717 | www.biotools.us

Title:

VCD Absolute Configuration Determination Report

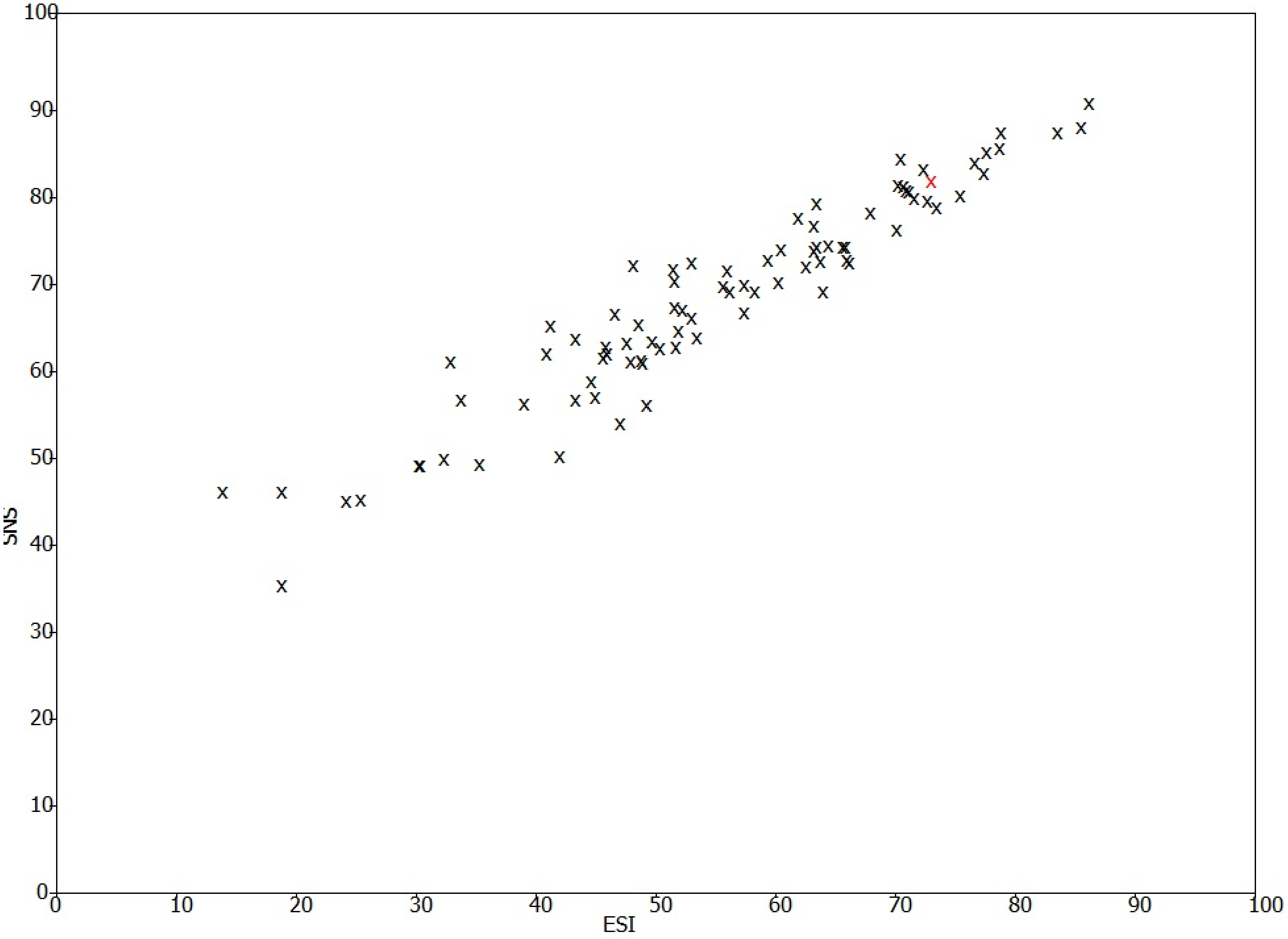

Plot of ESI (similarity of correct enantiomer minus incorrect enantiomer to calculated) vs SNS (overall similarity of correct enantiomer to calculated) for a library of correct assignments verified independently by X-Ray other method (Black X marks). Red X is **(-)-colchicine**. Upper right corner is the strongest results, lower left is the weakest.

17546 Bee Line Hwy | Jupiter, Florida 33478, USA
ph. 561 625.0133 | fax. 561 625.0717 | www.biotools.us
